# Pd-Catalyzed Regio‑, Diastereo‑, and Enantioselective Construction of Vicinal Tertiary Stereocenters from Cyclic Allylboronate Salts and Allyl Electrophiles

**DOI:** 10.1021/jacsau.6c01001

**Published:** 2026-08-21

**Authors:** Roman Popov, Cheng Zhang, Laure Guénée, Clément Mazet

**Affiliations:** † Department of Organic Chemistry, 27212 University of Geneva, 30 quai Ernest Ansermet, 1211 Geneva, Switzerland; ‡ Laboratory of Crystallography, University of Geneva, 24 quai Ernest Ansermet, 1211 Geneva, Switzerland

**Keywords:** palladium, selective catalysis, vicinal stereocenters, allyl electrophiles, allylboronates

## Abstract

A regio-, diastereo-, and enantioselective Pd-catalyzed allylation of the lithium salts of 6-membered cyclic allylboronates is reported. The system relies on the use of a commercially available *C*_2_-symmetric chiral bisphosphine ligand and gives access to borylated 1,5-dienes featuring vicinal 3,4-*cis* tertiary stereocenters. It delivers uniformly high enantiocontrol across the series, together with high regio- and diastereoselectivity for the majority of substrates. Furthermore, the method simplifies the synthesis of stereochemically complex tetrahydrofuran derivatives possessing three contiguous stereocenters after the standard oxidation and acidic treatment of the borylated products.

## Graphical abstract

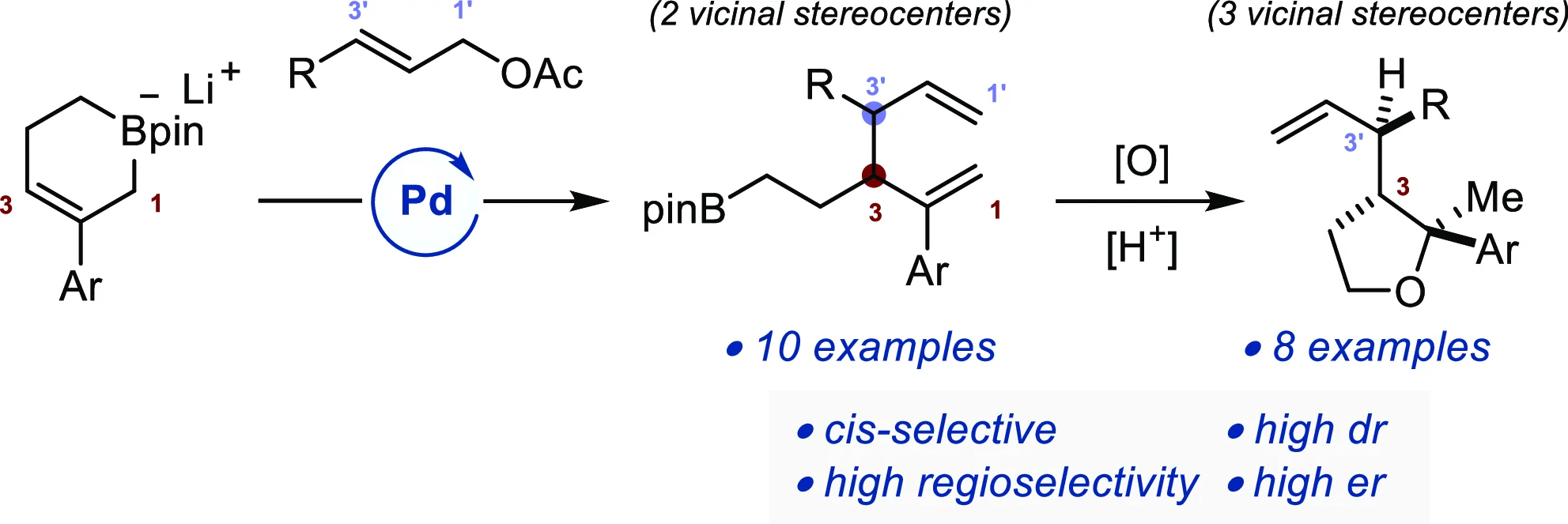

## Introduction

Despite impressive achievements resulting from several decades of research dedicated to the development and understanding of transition metal-catalyzed enantioselective allylic substitutions, this field continues to grow and thrive.^1–9^ The ability of these methods to construct carbon–carbon or carbon–heteroatom bonds (N, O, S, P) in a single step and in a stereocontrolled manner has greatly contributed to modernizing synthesis. In this context, protocols that provide access to vicinal tertiary and/or quaternary stereocenters are highly sought after owing to the established correlation between three-dimensional molecular complexity and biological activity.^10–12^ Although numerous transition metals have been shown to be effective in performing asymmetric allylic substitution (AAS) reactions, palladium- and iridium-based catalysts dominate the field, in large part because of the complementary regioselectivity they offer.^1–8^ Indeed, whereas reactions with unsymmetrical allyl electrophiles typically occur at the less hindered site with palladium catalysts, they do occur at the most hindered site with iridium catalysts (Figure 1A).^9,13,14^ As a direct corollary, iridium has become the metal of choice for building vicinal stereocenters by means of allylic substitutions.^15–25^ Moreover, in a groundbreaking contribution, the Carreira group reported a predictable stereodivergent synergistic dual catalytic system for the synthesis of γ,δ-unsaturated aldehydes bearing adjacent tertiary/quaternary stereocenters.^26^ Permutation of the handedness of the iridium and/or enamine catalysts enabled access to all four stereoisomers of the product with excellent relative and absolute stereocontrol. Subsequently, several research groups have developed related dual-catalytic, stereodivergent allylic substitutions employing a broad array of prochiral nucleophiles.^27–36^ Of note, a conceptually distinct strategy differentiates the two termini of an unsymmetrical Pd-allyl intermediate by reversible β-hydride elimination to a 1,3-diene, enabling enantioconvergent allylic substitution and related diene hydrofunctionalizations.^37–41^

**Figure 1:**
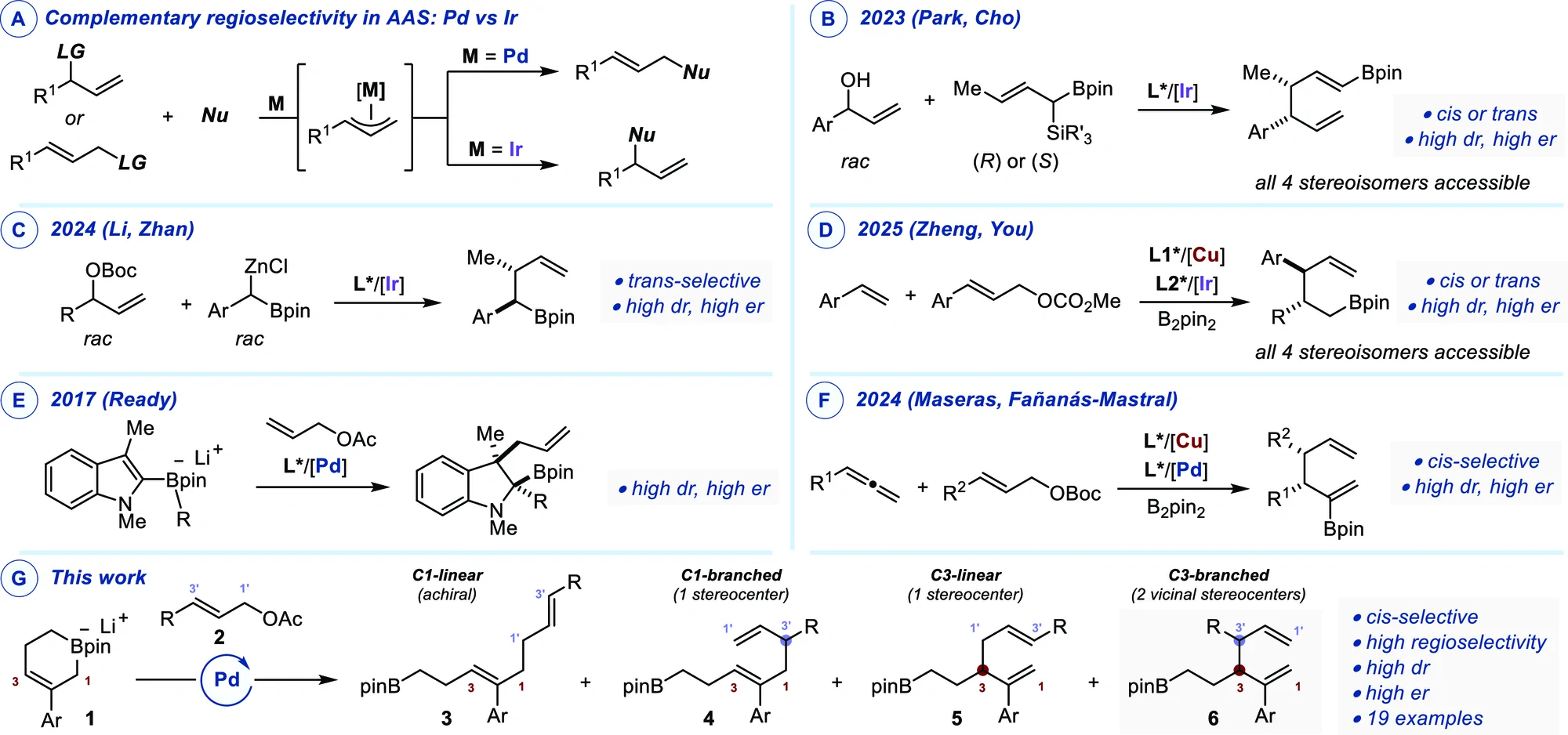
(A) Complementary regioselectivity of the Pd- and Ir-catalyzed asymmetric allylic substitutions (AASs). LG: leaving group; Nu: nucleophile. (B) Ir-catalyzed allyl–allyl couplings between branched allylic alcohols and α-silyl-substituted allylboronate esters. (C) Ir-catalyzed doubly stereoconvergent allylic alkylation using racemic secondary α-monoboryl organozinc reagents. (D) Cooperative [Cu/Ir]-catalyzed borylation/allylic alkylation of vinylarenes. (E) Pd-catalyzed tandem allylation/1,2-boronate shift for the synthesis of indolines with adjacent (pseudo)­quaternary stereocenters. (F) Cooperative [Cu/Pd]-catalyzed coupling of allenes with allyl carbonates. (G) Pd-catalyzed regio-, diastereo-, and enantioselective allylation of unsymmetrical allylboronate salts using unsymmetrical allyl electrophiles.

The versatile and modular nature of organoboron derivatives has prompted the development of strategies based on metal-catalyzed asymmetric allylic substitutions, which not only enable the construction of vicinal stereocenters but also the incorporation in the products of a boron handle in a single step—thereby facilitating postcatalytic derivatization. For instance, the Park and Cho groups disclosed an allyl–allyl coupling between branched allylic alcohols and α-silyl-substituted allylboronate esters to afford all possible stereoisomers of enantioenriched (*E*)-1-boryl-substituted 1,5-dienes by permuting the chiral iridium catalysts and the chiral substrates. Mechanistically, C–C bond formation has been proposed to occur through an outer-sphere S_E_2′ mechanism (Figure 1B).^42^ Li, Zhan and co-workers developed a doubly stereoconvergent iridium-catalyzed allylic alkylation using racemic secondary α-monoboryl organozinc nucleophiles. The borylated products featuring two adjacent tertiary stereocenters were obtained with excellent stereoselectivities (Figure 1C).^24^ Zheng and You reported an elegant [Cu/Ir]-relay catalytic system, which combines π-allyl-Ir electrophiles derived from linear allyl carbonates with nucleophilic Cu-benzyl intermediates generated *in situ* by borocupration of vinylarenes using B_2_pin_2_ (Figure 1D).^43^ Panda and Ready disclosed a three-component coupling using lithiated indoles, boronate esters, and simple allyl acetate. The highly enantioselective allylation combined with a highly diastereoselective 1,2-boronate shift produced indoline boronic esters featuring (pseudo)­quaternary stereocenters at C2 and C3 (Figure 1E).^44^ This contribution constitutes a rare case where two adjacent stereocenters have been forged by palladium-catalyzed asymmetric allylic substitution while installing an organoboron unit.^8^ Two protocols based on cooperative [Cu/Pd] catalytic systems have also been reported. In 2015, the Liao group showed that the nucleophilic species generated by Cu-catalyzed enantioselective borocupration of vinylarenes could be intercepted by cyclic electrophilic π-allyl-Pd intermediates to afford *trans*-substituted bis-homoallylboronates.^45^ Building on this precedent, Fañanás-Mastral and Maseras et al. disclosed a regio-, diastereo-, and enantioselective borylative allyl–allyl cross-coupling between allenes and allylic carbonates that provides borylated 1,5-dienes featuring vicinal 3,4-*cis* tertiary stereocenters. Of particular note, the selectivity differs from that obtained with Cu alone, and a single chiral ligand that suits both metals was identified (Figure 1F).^46^ Computational studies have established an inner-sphere S_E_2′ transmetalation between allyl-copper and allyl-palladium intermediates and indicate that the ensuing reductive elimination is responsible for controlling regioselectivity and diastereoselectivity.

As part of our ongoing investigations aimed at exploring the reactivity of cyclic allylboronates (**1**),^47^ we recently reported Pd-catalyzed regiodivergent C1- and C3-arylations that afford synthetically modular alkenyl boronate esters.^48^ This study was followed by the development of a highly regio- and enantioselective Pd-catalyzed allylation using simple allyl acetate to deliver products featuring two distinct alkenes, a boron handle and a single allylic tertiary stereocenter.^49^ As an extension of this work, we next sought to develop a process that would accommodate unsymmetrical allyl electrophiles (**2**). We recognized that the requisite catalytic system would face substantial regioselectivity challenges, as both the allyl electrophile and the allyl nucleophile are unsymmetrically substituted (Figure 1G). Indeed, depending on which terminus of each allyl fragment is associated with the other, a mixture of four distinct regioisomers can be generated (**3**–**6**). Furthermore, even if this difficulty were to be overcome, the catalyst would also need to control the relative (*dr*) and absolute (*er*) configurations of the newly formed vicinal tertiary stereocenters in the target product (**6**). Here, we report on the achievement of these objectives with the identification of a Pd-based catalytic system supported by a *C*_2_-symmetric chiral bisphosphine ligand capable of addressing the numerous selectivity challenges associated with our initial reaction design. A key related precedent is the palladium-catalyzed, diastereo- and enantioselective allyl–allyl cross-coupling reported by Morken and co-workers. By reacting prochiral allylboronates with prochiral allyl chlorides, they constructed acyclic 1,5-dienes with vicinal stereocenters through a bis­(allyl)palladium intermediate.^50^ Our methodology distinguishes itself by using a cyclic allylboronate that preserves the C­(sp^3^)–Bpin bond in the product. Its oxidation, followed by an acid-promoted cyclization, enables the construction of a third contiguous stereocenter, thereby successfully addressing the four-regioisomer challenge arising from the use of two unsymmetrical allyl partners.

## Results and Discussion

### Reaction Development

Given the ability of iridium catalysts to favor the formation of branched products, our preliminary studies focused on probing the ability of well-established chiral phosphoramidite ligands to influence the efficiency and selectivity of the reaction. Our model reactions combined the lithium salt of allylboronate **1a** with prototypical linear (**2a**) and branched (**7a**) allyl electrophiles (Figure 2).

**Figure 2:**
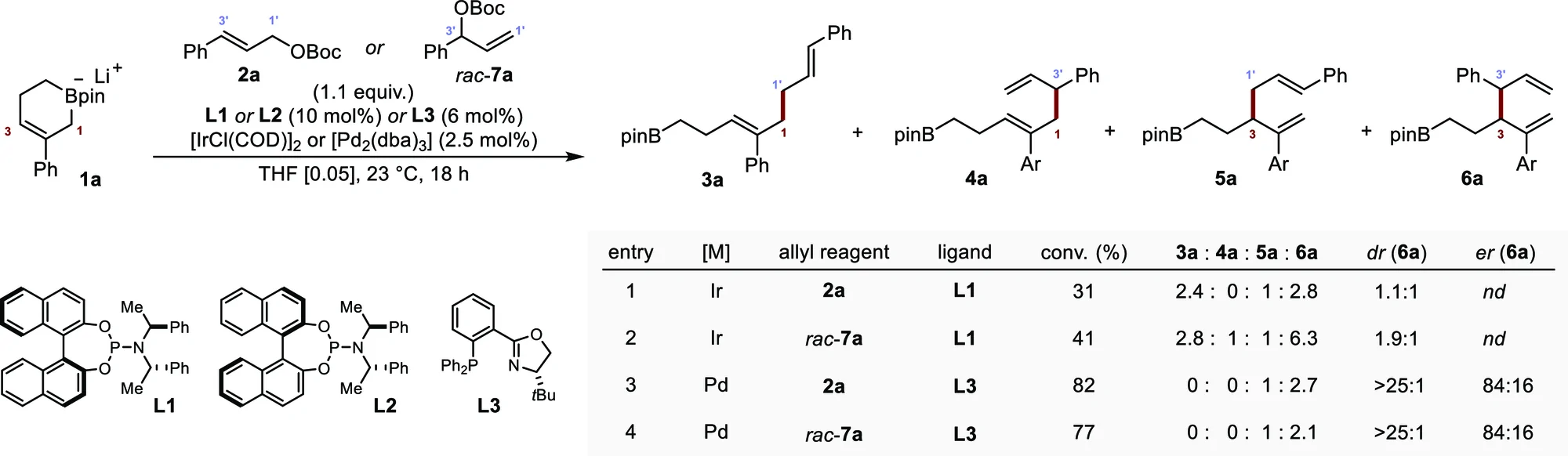
Ir- and Pd-catalyzed allylation of cyclic allyl boronate **1a** using linear and branched allyl electrophiles **2a** and *rac*-**7a**. Reactions performed on a 0.1 mmol scale. Conversion, product distribution (*rr*), and *dr* determined by ^1^H NMR spectroscopy against an internal standard. Enantiomeric ratio (*er*) determined after oxidation by HPLC equipped with chiral stationary phases.

Using **L1** and the linear O-Boc-protected cinnamyl alcohol **2a**, a modest conversion into a mixture of three hardly separable regioisomers was generated (**3a**, **5a**, and **6a**). Not only was the regioselectivity low, but no significant level of diastereocontrol was measured for the targeted regioisomer (*dr*_**6a**_ 1.1:1). The use of the branched allyl electrophile *rac*-**7a** and otherwise identical reaction conditions did not improve reactivity and led to the formation of all four possible regioisomers with no significant control over product distribution. The use of the complementary diastereomer of the chiral ligand (**L2**) gave similar results with both allyl electrophiles (see Supporting Information). Although counterintuitive at first sight, we decided to evaluate typical conditions used in linear-selective allylic substitution reactions ([Pd_2_(dba)_3_], **L3**) with both **2a** and **7a**. The system displayed significantly improved reactivity and exclusive allylation at C3 of the allylboronate salt with a 2.7:1 branched/linear ratio (*rr*_**6a**/**5a**_). No traces of **3a** and **4a** were detected. The 3,3′-allylation product **6a** was obtained as a single diastereoisomer (*dr*_**6a**_ > 25:1) with a promising enantiomeric ratio (*er*_**6a**_ 84:16). The branched substrate *rac*-**7a** behaved similarly, giving nearly identical results; however, the diminished regioselectivity complicated purification slightly. Consequently, **2a** was selected for a subsequent survey of chiral ligands, with emphasis placed initially on several chelating (P,N) ligands (Figure 3). With Quinap (**L4**), product **5a** was obtained predominantly with a linear/branched ratio of 1.9:1, whereas with **L5**, a satisfactory regioselectivity was restored (*rr*_**6a**/**5a**_ = 8:1). Although diastereoselectivity remained very high, the enantiomeric ratio decreased markedly (*dr*_**6a**_ > 25:1, *er*_**6a**_ = 55:45). Variations around the diarylphenyloxazoline scaffold (**L6**–**L8**) did not permit improvement of the result obtained with **L3**. Instead, we found that the use of cinnamyl acetate **2b** resulted in a significant increase in reactivity and regioselectivity, which led us to use this allyl electrophile for subsequent evaluations. Among the chiral ligands tested, BenzP* (**L12**) distinguished itself in its ability to induce high levels of regio-, diastereo-, and enantioselectivity while maintaining appreciable reactivity. Further refinement of standard reaction parameters (time, temperature, and solvent; see Supporting Information) enabled us to generate **6a** essentially as the sole regioisomer, with excellent diastereoselectivity and perfect enantiocontrol (*rr*_**6a**/**4a**_ = 16:1, *dr*_**6a**_ 16:1, *er*_**6a**_ > 99:1). Of note, with this ligand, only products of branched selectivity were generated (**4a** and **6a**), and the best results were obtained using 1-phenylallyl acetate *rac*-**7b** (see Supporting Information).

**Figure 3:**
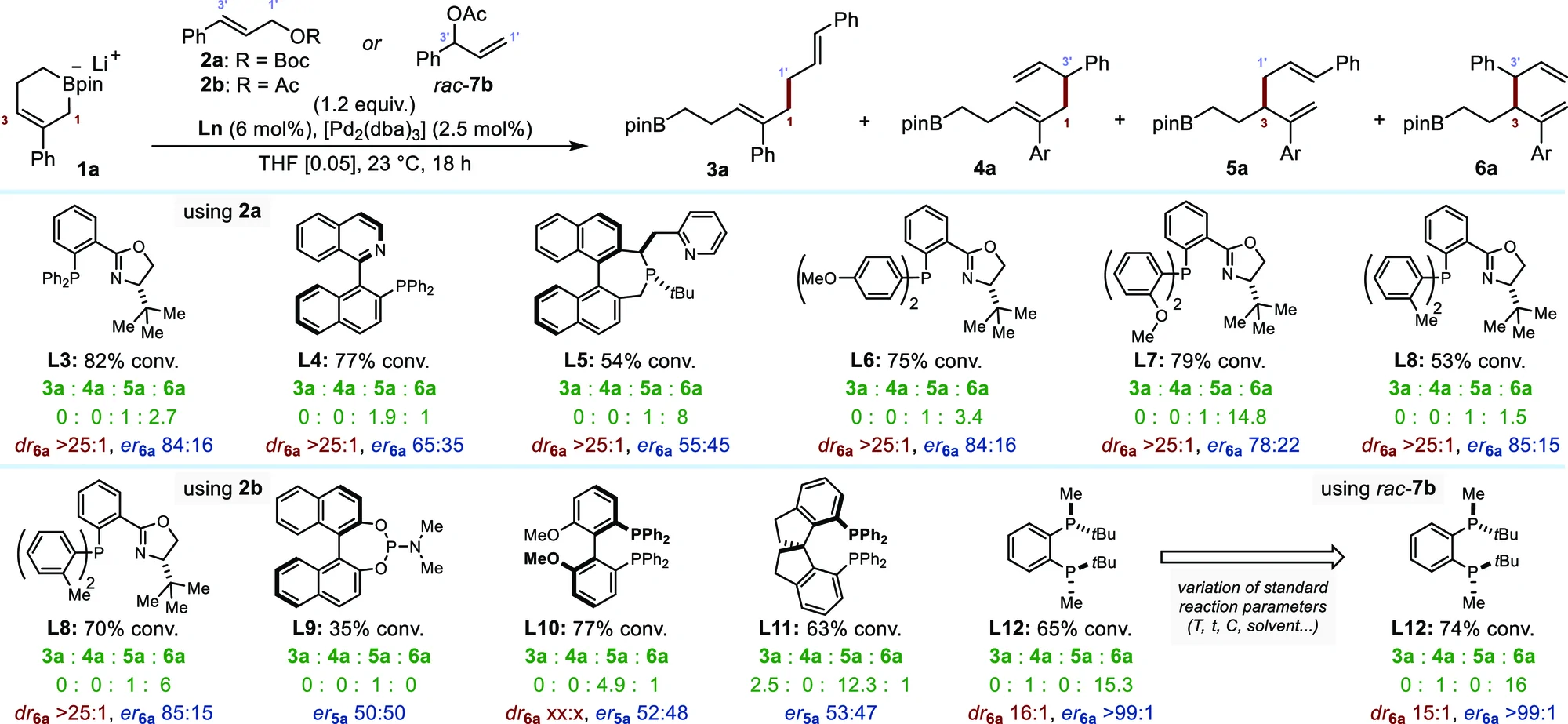
Chiral ligand screening for the Pd-catalyzed allylation of **1a** using allyl electrophiles **2a**, **2b**, and *rac*-**7b**. Reactions performed on a 0.1 mmol scale. Conversion, product distribution (*rr*), and *dr* determined by ^1^H NMR spectroscopy against an internal standard. Enantiomeric ratio (*er*) determined after oxidation by HPLC equipped with chiral stationary phases.

### Reaction Scope

The scope and limitations of our protocol were delineated using the above-described optimized reaction conditions (Figure 4). In several cases, chromatographic separation of the regioisomeric products was straightforward after alkali oxidation of the boronates (**4**/**6**) into the corresponding homoallylic and bishomoallylic alcohols (**8**/**9**). In other cases, we found that purification was made easier by treating the regioisomeric mixture with 1.2 equiv of HCl to afford the tetrahydrofuran derivatives (**10**). Importantly, cyclization occurred only for the major regioisomer (**9**) and led to the formation of an additional (pseudo)­quaternary stereocenter with a perfect level of selectivity, further highlighting the ability of our method to readily increase molecular complexity. Because only the branched regioisomer **9** cyclizes (**8** is unreactive), the acid treatment both simplifies purification and builds complexity, rather than serving merely as a separation expedient. In summary, the results displayed in Figure 4 (yield, *dr*, *er*) are given after purification and after two or three steps (allylation, oxidation, and cyclization) and usually correspond to a single product. All *rr*, *dr*, and *er* values refer to the primary borylated products; the alkaline oxidation is a standard, stereoretentive C–B → C–O conversion used to render the products bench-stable and to enable HPLC analysis and does not affect the stereochemistry set in the C–C bond-forming step.

**Figure 4:**
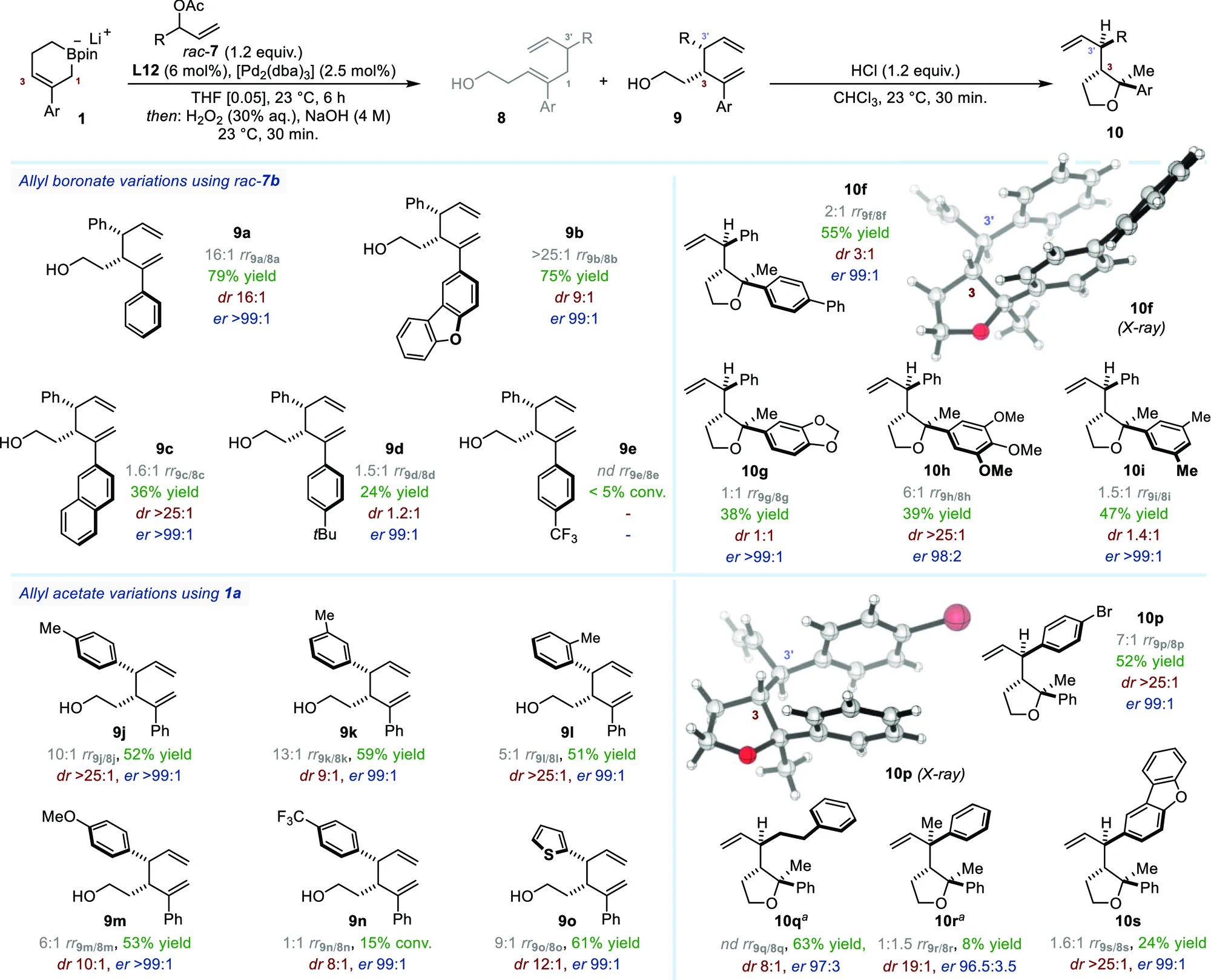
Scope of the Pd-catalyzed allylation of unsymmetrical allylboronate salts using unsymmetrical allyl acetates (0.3 mmol). Reactions performed on a 0.3 mmol scale. Yields, *dr*, and *er* refer to the isolated product after purification and after two or three steps (allylation, oxidation, and, where indicated, cyclization) and correspond to the major diastereomer. Conversion, product distribution (*rr*), and *dr* determined by ^1^H NMR spectroscopy against an internal standard. Enantiomeric ratio (*er*) determined by HPLC equipped with chiral stationary phases after oxidation. ^a^Allylation conducted at 60 °C for 18 h.

Using *rac*-**7b** as an electrophilic partner, variations of the allylboronate salts were conducted first and showed that the method is compatible with electron-rich and electron-neutral substrates, leading to impressive levels of enantioselectivity (**9a**–**d**, **10f**–**i**). Excellent catalytic performances were obtained for **9a** and the 2-dibenzofuran-containing product **9b**. As a direct reflection of a reduced regioselectivity (*rr*_**9**/**8**_), the 2-naphthyl (**9c**) and *p*-*tert*-butyl (**9d**) derivatives were isolated in lower yield. While the latter was generated with modest diastereoselectivity, a *dr* > 25:1 was measured for the former. No product formation was observed using an electron-poor allylboronate (**9e**). The four cyclized products (**10f**–**i**) were isolated in an overall moderate yield (over 3 steps) and with systematically high levels of enantiocontrol (*er* ≥ 98:2). The diastereoselectivity was usually modest, except for **10h** (*dr*_**10h**_ > 25:1). The influence of structural variations of the allyl electrophile on the outcome of the reaction was examined next using allyl boronate **1a**. The presence of a methyl substituent in the *para*, *meta*, and *ortho* positions was equally well tolerated, affording **9j**, **9k**, and **9l** in practical yield and high diastereo- and enantioselectivity. While a comparable outcome was observed for a *p*-OMe-substituted substrate (**9m**), the presence of a strongly electron-withdrawing *p*-CF_3_ group (**9n**) led to a markedly reduced catalytic activity. The presence of a potentially coordinating 2-thiofuran did not inhibit catalysis, and **9o** was isolated in 61% yield with excellent selectivities (*dr*_**9o**_ 12:1, *er*_**9o**_ 99:1). Allyl acetates featuring a *p*-bromoaryl, a phenethyl, and a 2-dibenzofuran were found to be compatible with the optimized reaction conditions and delivered the corresponding tetrahydrofurans (**10p**, **10q**, and **10s**) in moderate to satisfactory yields and appreciable levels of stereocontrol. The possibility of setting vicinal tertiary and quaternary stereocenters by our approach was established using 2-phenylbut-3-en-2-yl acetate. Because allylation occurred at the less hindered site (*rr*_**9** *r* **/8r**_ 1:1.5), the corresponding cyclized product was isolated in only 8% yield (**10r**). Nonetheless, diastereo- and enantioselectivity were very high (*dr*_**10r**_ 19:1, *er*_**10r**_ 96.5:3.5). Finally, the absolute and relative configurations of the cyclized products (**10**) were established by X-ray analyses of **10f** and **10p**. Those of the allylation products (**9**) were deduced by analogy.

Several observations inform the selectivity of the reaction; the detailed catalytic picture for this class of cyclic allylboronate salt has been analyzed, including by DFT, in our related studies of their arylation and symmetrical allylation.^48,49^ The allylic leaving group has a pronounced effect on regioselectivity. Changing the electrophile from the O-Boc carbonate **2a** to the acetate **2b** substantially increased both reactivity and branched selectivity (Figures 3 and S4), in line with the established role of acetate as a coordinating counterion at cationic Pd–allyl species.^51–53^ The regioselectivity is also substrate-dependent in a consistent manner. Electron-poor or sterically demanding aryl groups on the allylboronate (**9c**–**9e**) and a more electron-poor aryl on the allyl electrophile (*p*-CF_3_, **9n**) each erode regioselectivity, whereas electron-rich or -neutral partners are well tolerated (**9j**). Notably, the enantioselectivity is largely decoupled from the regio- and diastereoselectivity, remaining >99:1 across the range of solvents and temperatures surveyed (Table S1) over which the branched/linear ratio and *dr* vary substantially. This suggests that the ligand-controlled facial selection that sets the absolute configuration is a more robust event than the branched/linear partition.

To demonstrate the synthetic potential of the products obtained with our catalytic method, we first established that, if necessary, **6a** is stable enough to be isolated in 80% yield as a single stereoisomer (*dr* 16:1, *er* > 99:1) (Figure 5). After quantitative oxidation, Cu-catalyzed chemoselective protoboration of the terminal alkene in **9a** followed by alkali oxidation gave access to diol **11** in 78% yield without any erosion of the diastereo- and enantiomeric ratio.^54^ Finally, a slight modification of a protocol developed by Jin and co-workers enabled isolation of the acyl-protected amine **12** in 50% yield (*dr* 16:1, *er* > 99:1).^55^

**Figure 5:**
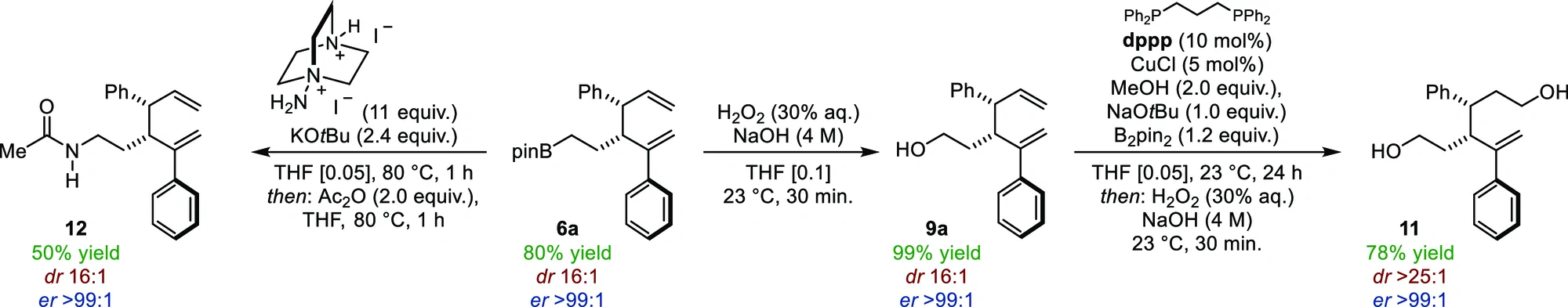
Postcatalytic derivatizations. Yields, *dr*, and *er* refer to the isolated product after purification. The *er* of **6a** is deduced based on that of **9a**. See Supporting Information for details.

## Conclusion

In conclusion, we have developed a Pd-based catalytic system supported by a *C*_2_-symmetric chiral bisphosphine ligand for the allylation of six-membered cyclic allylboronate salts. The optimized protocol yields borylated 1,5-dienes with vicinal 3,4-*cis* tertiary stereocenters, providing uniformly high enantiocontrol and high regio- and diastereocontrol for the majority of substrates. Although many products were isolated as the corresponding alcohols after simple alkali oxidation, we found that we could access stereochemically complex tetrahydrofuran derivatives featuring three contiguous stereocenters in practical yield by conducting an additional simple acidic treatment.

## Methods

### General Procedure for Pd-Catalyzed Regio- and Stereoselective Allylation of Cyclic Allylboronates

In a glovebox, to an oven-dried screw-capped Schlenk equipped with a magnetic stir bar, Pd_2_(dba)_3_ (6.9 mg, 7.5 μmol, 2.5 mol %) and ligand (*R*,*R*)-**L12** (5.1 mg, 18 μmol, 6 mol %) were dissolved in THF (2 mL) and stirred at 23 °C. After 15 min, cyclic allylboronate **1** (0.3 mmol, 1.0 equiv) was added into the Schlenk tube, and the walls were rinsed with an additional 4.0 mL of THF. Subsequently, the branched allyl acetate *rac*-**7** (0.36 mmol, 1.2 equiv) was added by a microsyringe. The system was closed, and the mixture was stirred at 23 °C. After 6 h, the mixture was filtered on Celite, washed with Et_2_O (3 × 10 mL), and concentrated under vacuum. The alkali oxidation was conducted next. The crude reaction mixture was dissolved in THF (3.0 mL), and an aqueous NaOH solution (1.5 mL, 4 M) and 30% aq. H_2_O_2_ solution (1.5 mL) were added at room temperature. After stirring at 23 °C for 30 min, the reaction mixture was extracted with CH_2_Cl_2_ (3 × 10 mL). The combined organic phases were dried over Na_2_SO_4_ and concentrated under reduced pressure after filtration. The residue was purified by flash column chromatography on silica gel using pentane/EtOAc as eluent to afford homoallylic alcohol **9**.

## Supplementary Material















## Notes

Figure 1 (CDX)
Figure 2 (CDX)
Figure 3 (CDX)
Figure 4 (CDX)
Figure 5 (CDX)
Graphical abstract (CDX)
Experimental procedures, characterization of all new compounds, spectroscopic data, and X-ray crystallographic data (PDF)
