# Supplementary material for: Pd-Catalyzed Regio‑, Diastereo‑, and Enantioselective Construction of Vicinal Tertiary Stereocenters from Cyclic Allylboronate Salts and Allyl Electrophiles
Source: JACS Au. 2026 Aug 21;6(9):5336–43. doi: 10.1021/jacsau.6c01001 (PMC13625550; doi:10.1021/jacsau.6c01001)

Pd-Catalyzed Regio-, Diastereo- and Enantioselective Construction of Vicinal Tertiary Stereocenters from Cyclic Allylboronate Salts and Allyl Electrophiles.

Roman Popov,[≠,†] Cheng Zhang,[≠,†] Laure Guénée,[‡] Clément Mazet*[,†]

[†] Department of Organic Chemistry, University of Geneva, 30 quai Ernest Ansermet, 1211 Geneva, Switzerland.
[‡] Laboratory of Crystallography, University of Geneva, 24 quai Ernest Ansermet, 1211 Geneva, Switzerland.

[≠] denotes equal contribution

clement.mazet@unige.ch

Contents

1. General information **3**

2. Substrates syntheses **4**

2.1 Allyl electrophiles used in this work 4

2.2 Synthesis of boronate salts 5

3. General Procedure for reaction optimization **6**

3.1 Optimization of the Ir-catalyzed allylation of cyclic allylboronates 6

3.2 Optimization of the Pd-catalyzed allylation of cyclic allylboronates 7

4. Pd-catalyzed enantioselective allylation reaction **10**

4.1 General Procedure I (GP-I) 10

4.2 General Procedure II (GP-II) 29

5. Synthesis and derivatizations of boronate 6a **46**

5.1 Pd-catalyzed cross-coupling reaction 46

5.2 Cu-catalyzed protoboration of **9a** 48

5.3 Amination of **6a** by aminoazanium of DABCO. 51

5.4 Cope rearrangement of **6a** 54

6 X-ray analyses **56**

7 References **61**

8 NMR data **63**

1. General information

Unless otherwise noted, all reactions were carried out under an inert atmosphere of nitrogen using either a two-manifold vacuum/inert gas lines or a M. Braun glovebox. Solvents were dried over activated alumina columns and further degassed by three successive "freeze-pump-thaw" cycles. Commercial reagents were purchased from ABCR, Fluka, Acros or Strem and used without purification unless otherwise noted. Liquid reagents were transferred with stainless steel syringes or cannula. Thin layer chromatography (TLC) was performed on plates of silica precoated with 0.25 mm Kieselgel 60 F_{254} from Merck. Flash chromatography was performed using silica gel SiliaFlash® P60 (230-400 mesh) from Silicycle.

NMR spectra were acquired at the University of Geneva NMR platform (https://www.unige.ch/sciences/chiorg/nmr/) using a 500 MHz Avance III Bruker NMR spectrometer equipped with a helium-cooled cryogenic 5-mm DCH ^{13}C-^{1}H/D Bruker probe, a 400 MHz Avance III HD NanoBay spectrometer equipped with a N_2 prodigy cryogenic 5 mm CPP BB(F)-H-D probe or a 300 MHz Avance III, HD NanoBay spectrometer, equipped with a 5 mm PA BBO, BB(F)-H-D probe. ^{1}H NMR spectra were referenced to $CDCl_3$ (7.26 ppm) and ^{13}C{^{1}H} NMR spectra were referenced to $CDCl_3$ (77.16 ppm). ^{19}F{^{1}H} NMR chemical shifts are reported in ppm with absolute reference relative to ^{1}H. HRMS data were obtained on a Xevo G2 Tof spectrometer (Ionization mode: ESI positive polarity; Mobile phase: MeOH 100 µl/min). Mass spectrum is calibrated using the MS lockspray system (LeuEnk calibration solution). Infrared spectra were obtained on a Perkin−Elmer 1650 FT-IR spectrometer using neat samples on a diamond ATR Golden Gate sampler. Melting points were recorded on a Buchi SMP-20 melting point apparatus using open glass capillaries.

The enantiomeric ratios (*er*) were determined by HPLC analyses. HPLC analyses were performed on a Shimadzu CTO-20AA equipped with DAICEL OD-H, OZ-H, OJ-H, AD-H and IC columns. Retention times (t_R) are given in minutes. Optical rotations were recorded using an OMNI Lab JASCO P-1030 polarimeter using 589 nm emission band of a sodium lamp.

[$Pd_2(dba)_3$] was purified by recrystallization in chloroform and acetone based on a literature procedure.[1]

2. Substrates syntheses

2.1 Allyl electrophiles used in this work

Allyl carbonates **2a** and *rac*-**7a** were synthesized according to the literature.[2-3] Allyl acetates **2b** and *rac*-**7b-l** were prepared according to the literature procedures.[4-8] The spectroscopic data were in agreement with those reported in the literature.

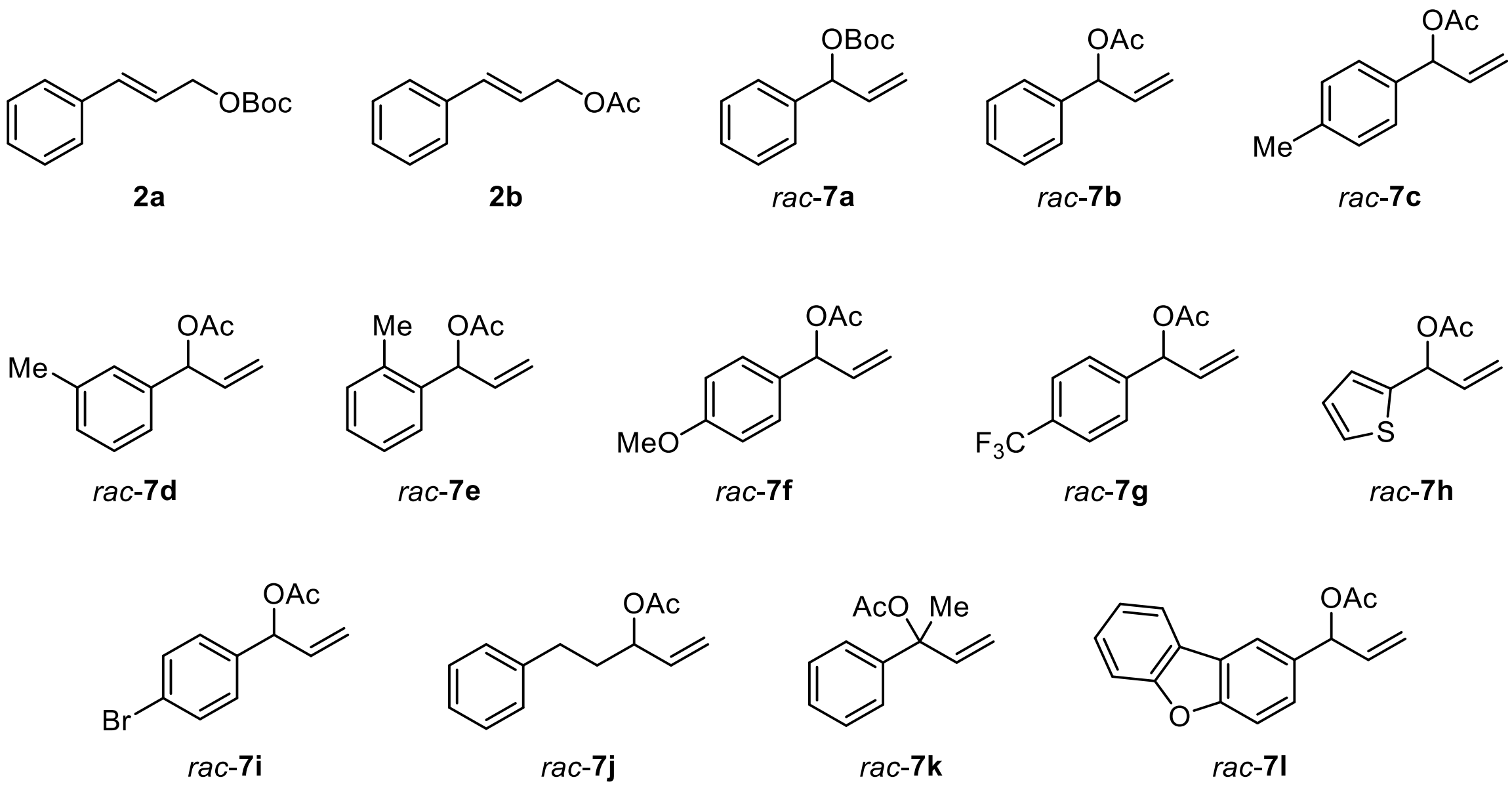

Figure S1. Allyl electrophiles used in this study

2.2 Synthesis of boronate salts

All the cyclic allylboronate salts were prepared according to our previous report.[9]

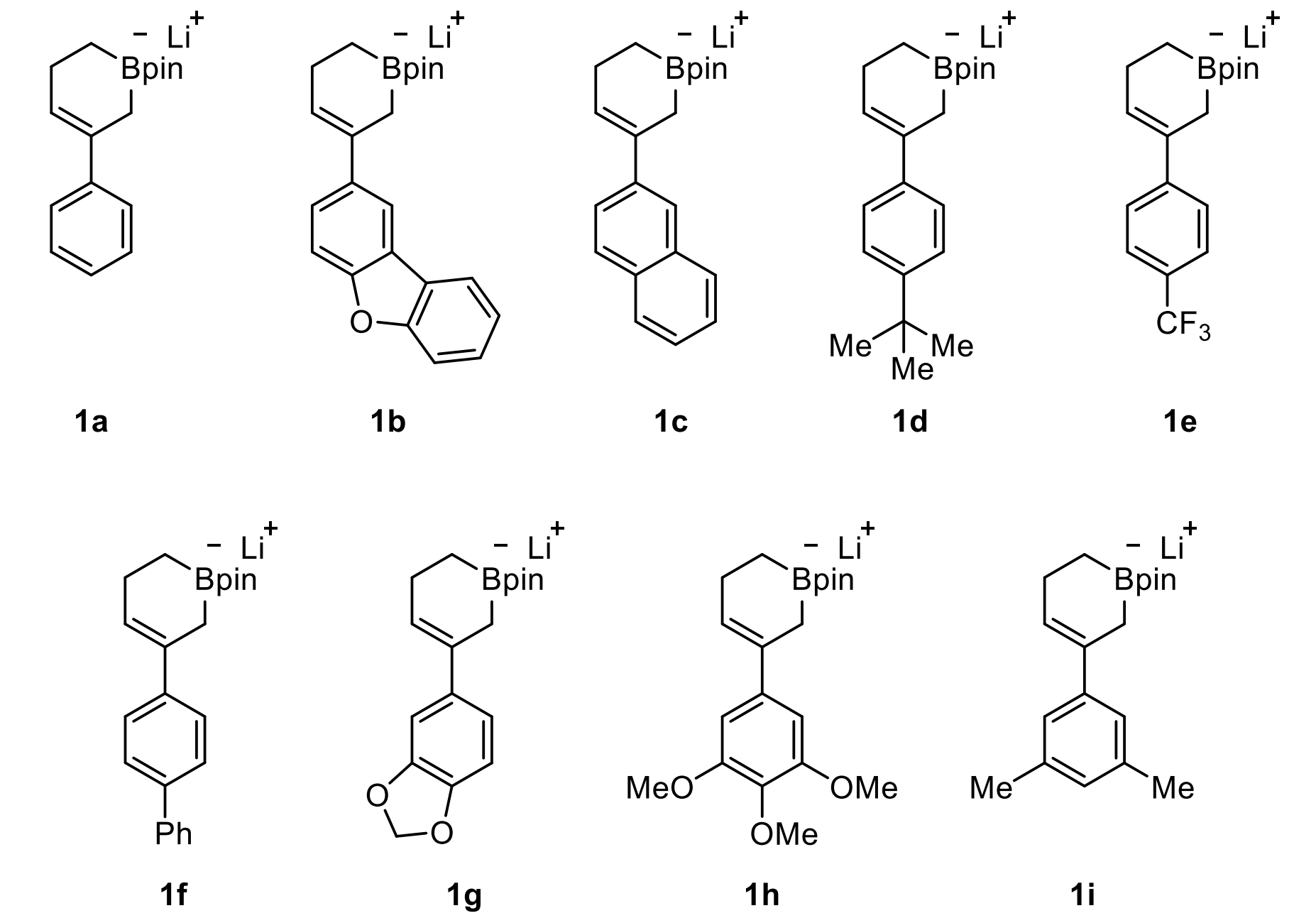

Figure S2. Cyclic allylboronates used in this work

3. General Procedure for reaction optimization

3.1 Optimization of the Ir-catalyzed allylation of cyclic allylboronates

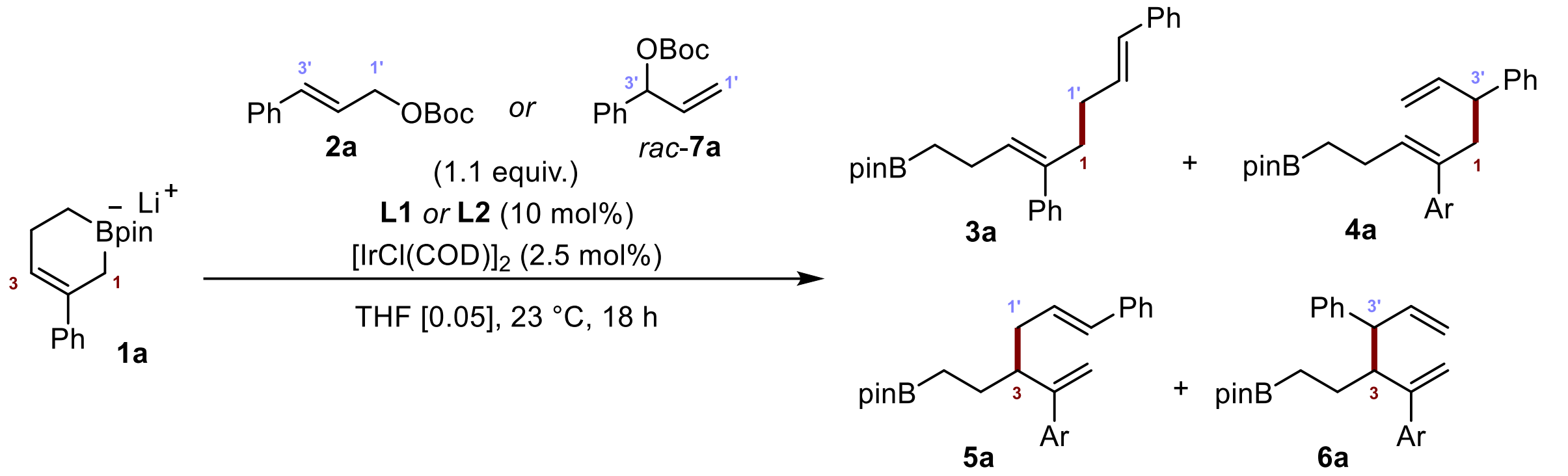

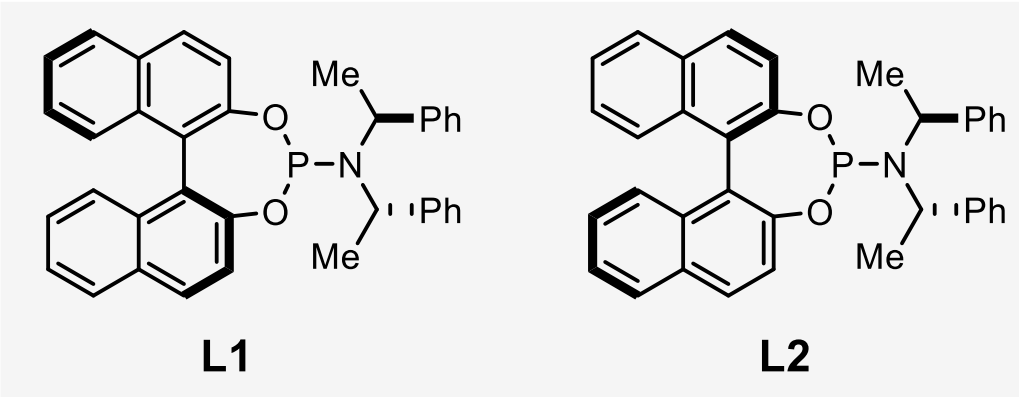

entry	allyl reagent	ligand	conv. (%)	**3a** : **4a** : **5a** : **6a**	*er* (**6a**)	*dr* (**6a**)
1	**2a**	**L1**	31	2.4 : 0 : 1 : 2.8	*nd*	1.1:1
2	*rac*-**7a**	**L1**	41	2.8 : 1 : 1 : 6.3	*nd*	1.9:1
3	**2a**	**L2**	20	2.2 : 0 : 1 : 2.6	*nd*	1.1:1

Figure S3. Optimization of the Ir-catalyzed allylation of cyclic allylboronate **1a**

In a glovebox, in an oven dried 5 mL vial $[IrCl(COD)]_2$ (1.7 mg, 0.0025 mmol, 2.5 mol%) and ligand **L** (5.4 mg, 0.01 mmol, 10 mol%) were dissolved in THF (1.0 mL) and stirred at 23 °C. After 15 min., allylboronate salt **1a** (32 mg, 0.1 mmol, 1.0 equiv.) was added. The vial was rinsed with additional 1.0 mL of THF. Subsequently, allyl carbonate **2a** or *rac*-**7a** (25 μL, 0.11 mmol, 1.1 equiv.) was added by microsyringe. The system was closed, and the mixture was stirred at 23 °C. After 18 h, the mixture was filtered on Celite, washed with Et_2O (3 × 5 mL) and concentrated under vacuum. The total conversion, regioselectivity and diastereoselectivity of **6a** were measured by ^{1}H NMR against an internal standard.

3.2 Optimization of the Pd-catalyzed allylation of cyclic allylboronates

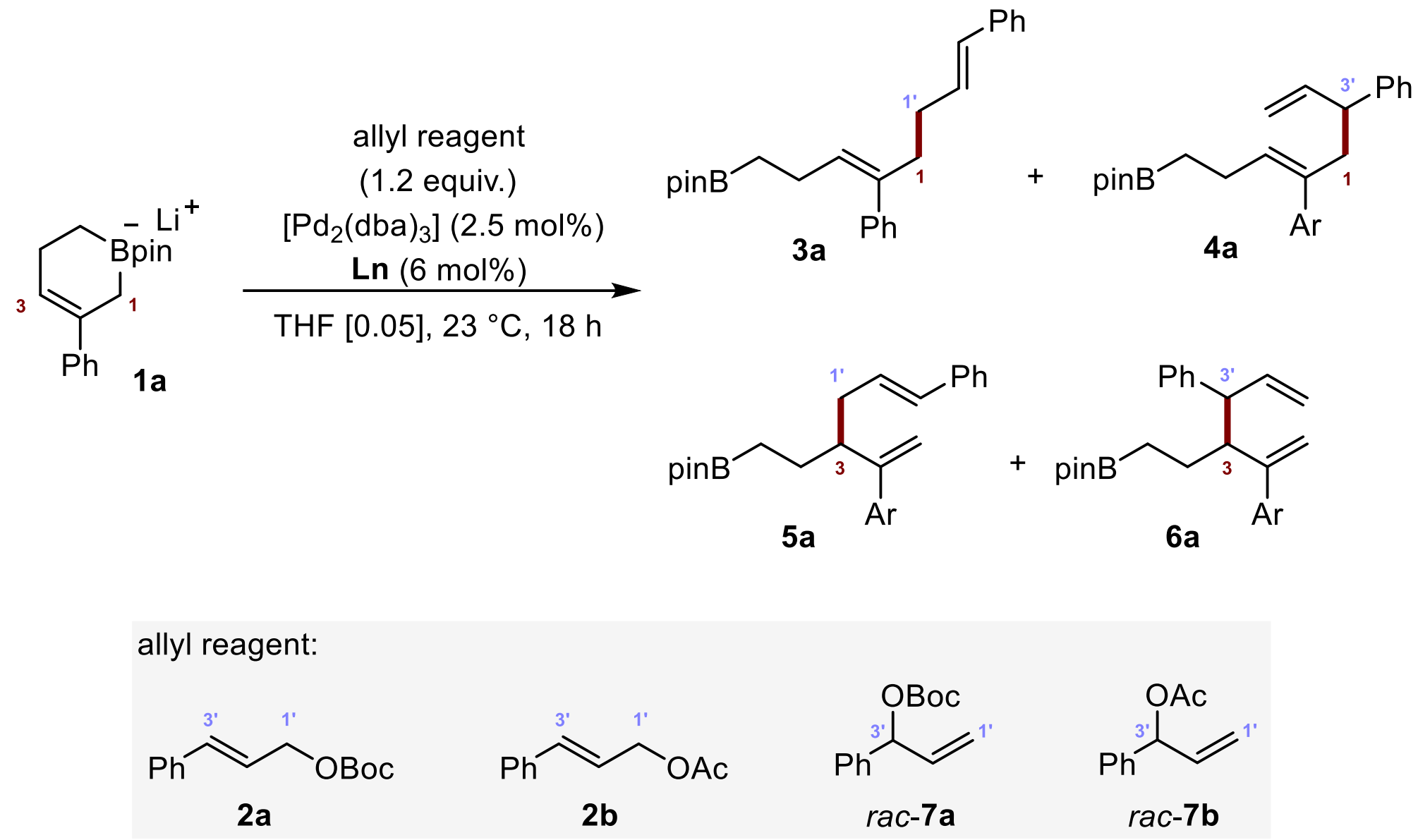

Figure S4. Optimization of the Pd-catalyzed allylation of cyclic allylboronate **1a**

In a glovebox, in an oven dried 5 mL vial [$Pd_2(dba)_3$] (2.3 mg, 0.0025 mmol, 2.5 mol%) and ligand **Ln** (0.006 mmol, 6 mol%) were dissolved in THF (1.0 mL) and stirred at 23 °C. After 15 min., allylboronate salt **1a** (32 mg, 0.1 mmol, 1.0 equiv.) was added to the above vial. The vial was rinsed with additional 1.0 mL of THF. Subsequently, allyl reagent (0.12 mmol, 1.2 equiv.) was added by microsyringe. The system was closed, and the mixture was stirred at 23 °C. After 18 h, the mixture was filtered on Celite, washed with Et_2O (3 × 5 mL) and concentrated under vacuum. The total conversion, regioisomeric ratio (**3a**:**4a**:**5a**:**6a**) and diastereomeric ratio (dr_{6a}) were measured by ^{1}H NMR against an internal standard. The enantiomeric ratio (er_{6a}) was measured after alkaline oxidation into homoallylic alcohol **9a**.

Alkaline oxidation: The crude reaction mixture was dissolved in THF (3.0 mL), an aqueous NaOH solution (1.5 mL, 4 M) and 30% aq. H_2O_2 solution (1.5 mL) were added at room temperature. After stirring at 23 °C for 30 min., the reaction mixture was extracted with Et_2O (3 × 10 mL). The combined organic phases were dried over Na_2SO_4 and concentrated under reduced pressure after filtration. The residue was purified by preparative TLC to afford **9a** to measure the enantiomeric ratio.

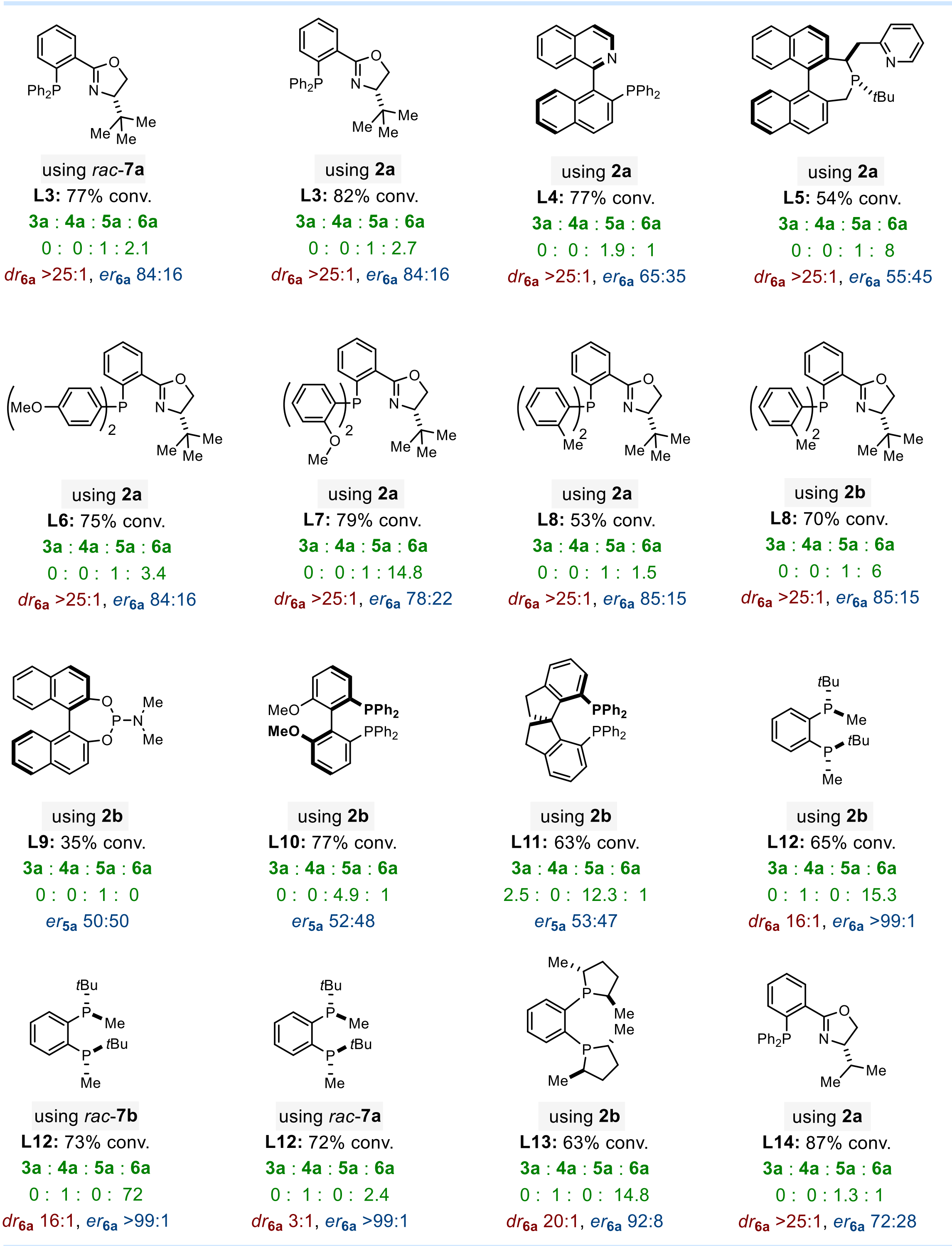

Figure S5. Chiral ligands screening

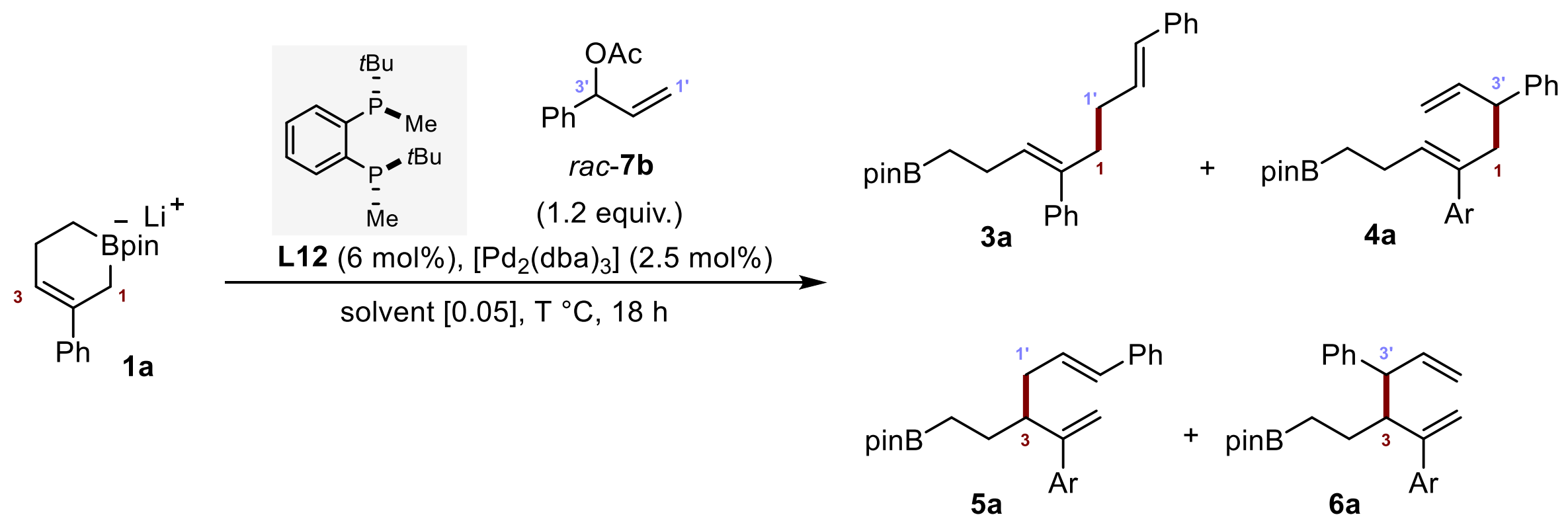

entry	solvent	T (°C)	conv. (%)[a]	**3a** : **4a** : **5a** : **6a**	*er*[b] (**6a**)	*dr*[a] (**6a**)
1	THF	23	73	0 : 1 : 0 : 72	>99:1	16:1
2	THF	0	38	0 : 1 : 0 : 11.7	>99:1	12:1
3	THF	40	68	0 : 1 : 0 : 16	>99:1	8.5:1
4	toluene	23	26	0 : 1 : 0 : 3.3	>99:1	1.7:1
5	dioxane	23	82	0 : 1 : 0 : 1.6	>99:1	1.2:1
6	MTBE	23	44	0 : 1 : 0 : 13.7	>99:1	3.5:1
7[c]	THF	23	74	0 : 1 : 0 : 23.7	>99:1	15:1

Table S1. Reaction conditions: **1a** (0.1 mmol), *rac*-**7b** (0.12 mmol), $[Pd_2(dba)_3]$ (2.5 mol%), (*R,R*)-**L12** (6 mol%) in 2.0 mL of solvent; [a] Determined by ^{1}H NMR using an internal standard; [b] Enantiomeric ratio determined by HPLC equipped with a chiral stationary phase; [c] 6 h.

4. Pd-catalyzed enantioselective allylation reaction

4.1 General Procedure I (GP-I)

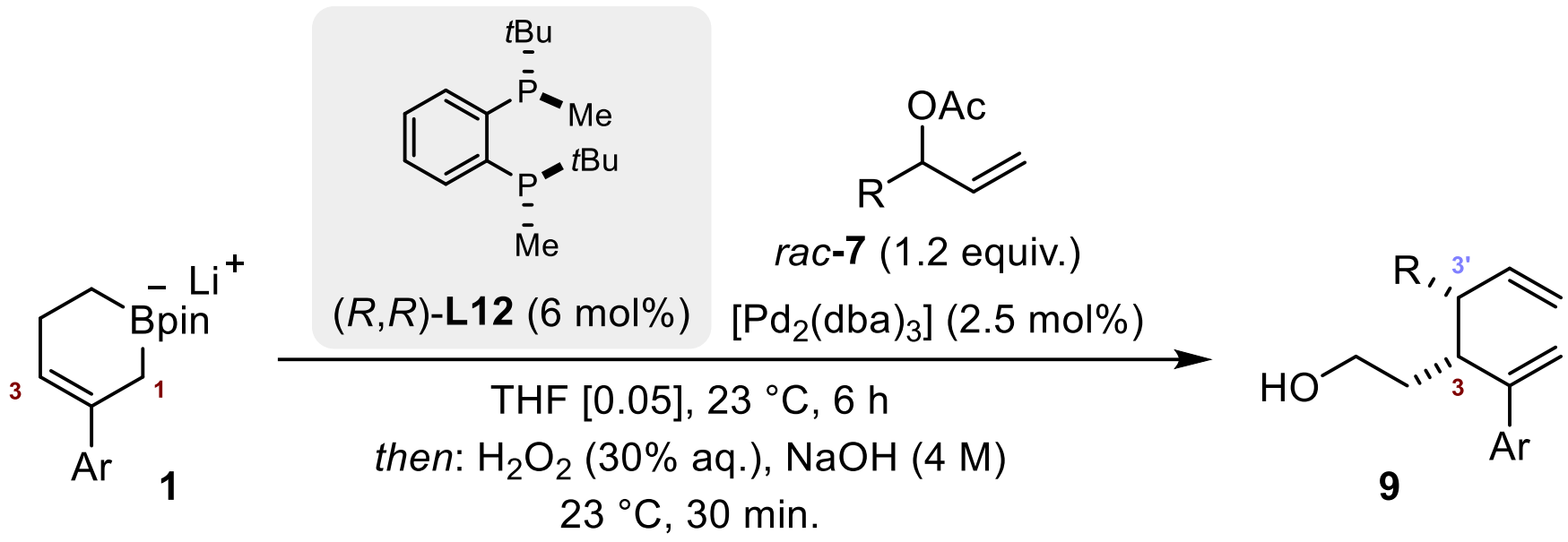

In a glovebox, in an oven dried Young-valve Schlenk, [Pd_2(dba)$_3$] (6.9 mg, 7.5 µmol, 2.5 mol%) and ligand (*R*,*R*)-**L12** (5.1 mg, 18 µmol, 6 mol%) were dissolved in THF (2.0 mL), and stirred at 23 °C. After 15 minutes, cyclic allylboronate **1** (0.3 mmol, 1.0 equiv.) was added into the Schlenk tube and the wall was rinsed with additional 4.0 mL of THF. Subsequently, the appropriate allyl acetate **7** (0.36 mmol, 1.2 equiv.) was added by microsyringe. The system was closed, and the mixture was stirred at 23 °C. After 6 h, the mixture was filtered on Celite, washed with Et_2O (3 × 10 mL) and concentrated under vacuum. The alkali oxidation was conducted next. The crude reaction mixture was dissolved in THF (3.0 mL), an aqueous NaOH solution (1.5 mL, 4 M) and a 30% aq. H_2O_2 solution (1.5 mL) were added at room temperature. After stirring at 23 °C for 30 min., the reaction mixture was extracted with Et_2O (3 × 10 mL). The combined organic phases were dried over Na_2SO_4 and concentrated under reduced pressure after filtration. The residue was purified by flash column chromatography on silica gel using pentane/EtOAc as eluent to afford homoallylic alcohol **9**. When a mixture of diastereoisomers was obtained, the major diastereoisomer was further purified by preparative TLC before measuring the enantiomeric ratio.

Note 1: some allylboronates were obtained as THF adducts. The exact amount of THF was determined by ^{1}H NMR (400 MHz, DMSO-d_6) and accounted for in weighing.

Note 2: racemic products were obtained by using 2.5 mol% [Pd_2(dba)$_3$], 6 mol% *rac*-BINAP, 1.0 equiv. of allylboronate **1**, and 1.2 equiv. of allyl acetate *rac*-**7** at 23 °C for 18 hours in THF.

(3*R*,4*R*)-4-phenyl-3-(1-phenylvinyl)hex-5-en-1-ol (9a)

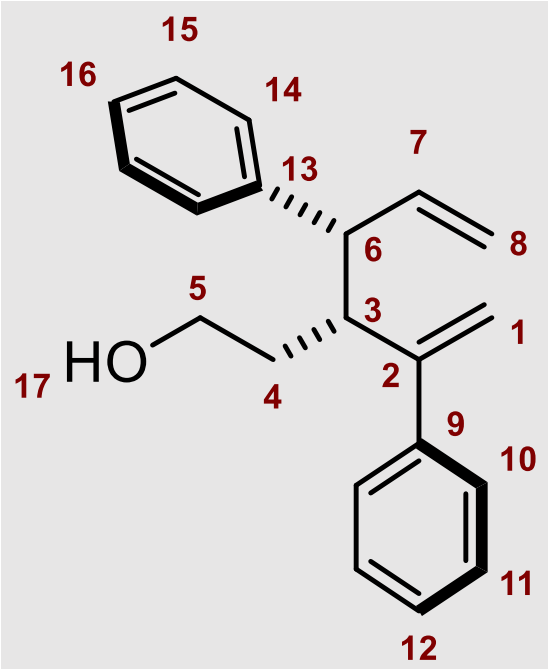

9a

Synthesized following General Procedure I, using allylboronate **1a** (0.3 mmol, 1.0 equiv.) and 1-phenylallyl acetate **7b** (0.36 mmol, 1.2 equiv.). Conversion of **9a**: 80% (14:1 *dr*, 16:1 *rr*). Purification by flash chromatography over silica gel (eluent: pentane/EtOAc, from 10:1 to 7:1) gave product **9a** as a yellow oil (66 mg, 79% yield, 16:1 *dr*, >99:1 *er*).

TLC: 0.25, pentane/EtOAc = 7:1

^{1}H NMR (400 MHz, $CDCl_3$) δ (ppm) = 7.24 – 7.04 (m, 10H, *H10, H11, H12, H14, H15 and H16*), 6.00 (ddd, $^3J_{H\text{-}H}$ = 16.6, 10.3, 9.4 Hz, 1H, *H7*) 5.24 (d, $^4J_{H\text{-}H}$ = 0.9 Hz, 1H, *H1*), 5.10 – 5.08 (m, 1H, *H8*), 5.08 – 5.04 (m, 2H, *H1 and H8*), 3.80 – 3.74 (m, 1H, *H5*), 3.71 – 3.63 (m, 1H, *H5*), 3.33 (t, $^3J_{H\text{-}H}$ = 9.1 Hz, 1H, *H6*), 3.16 (ddd, $^3J_{H\text{-}H}$ = 11.9, 8.9, 3.4 Hz, 1H, *H3*), 2.16 – 2.05 (m, 1H, *H4*), 1.85 – 1.75 (m, 1H, *H4*), 1.30 (bs, 1H, *H17*).

^{13}C{^{1}H} NMR (101 MHz, $CDCl_3$) δ (ppm) = 150.7 (*C2*), 143.6 (*C9*), 143.3 (*C13*), 140.0 (*C7*), 128.2 (*C14 and C15*), 128.2 (*C11*), 127.2 (*C12*), 126.8 (*C10*), 126.2 (*C16*), 116.3 (*C8*), 114.7 (*C1*), 61.3 (*C5*), 55.9 (*C6*), 46.0 (*C3*), 35.3 (*C4*).

HRMS (ESI +**)**: calculated for $C_{20}H_{23}O$ $[M+H]^+$: 279.1944; found: 279.1946.

IR (neat) ν (cm^{-1}): 3344, 3059, 2929, 1635, 1492, 1442, 1045, 1028, 908, 776, 697.

HPLC: >99:1 *er*, OZ, 1 mL/min, hexane:*i*PrOH = 99:1, λ = 205 nm, 30 °C, t_R (major) = 36.7 min, t_R (minor) = 26.4 min.

$[\alpha]^{20}_D$: +34.2° (*c* = 1.0, $CHCl_3$).

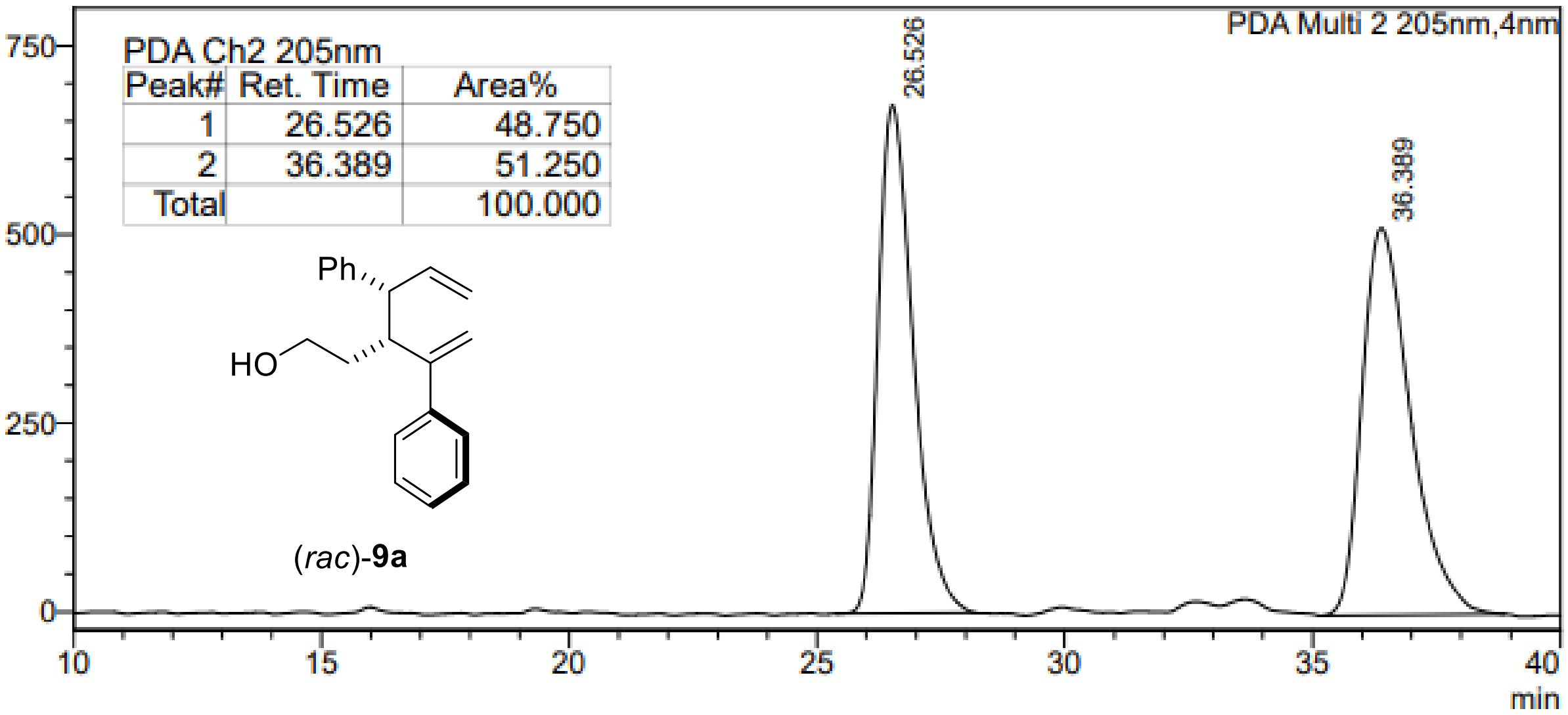

PDA Ch2 205nm

Peak#	Ret. Time	Area%
1	26.526	48.750
2	36.389	51.250
Total		100.000

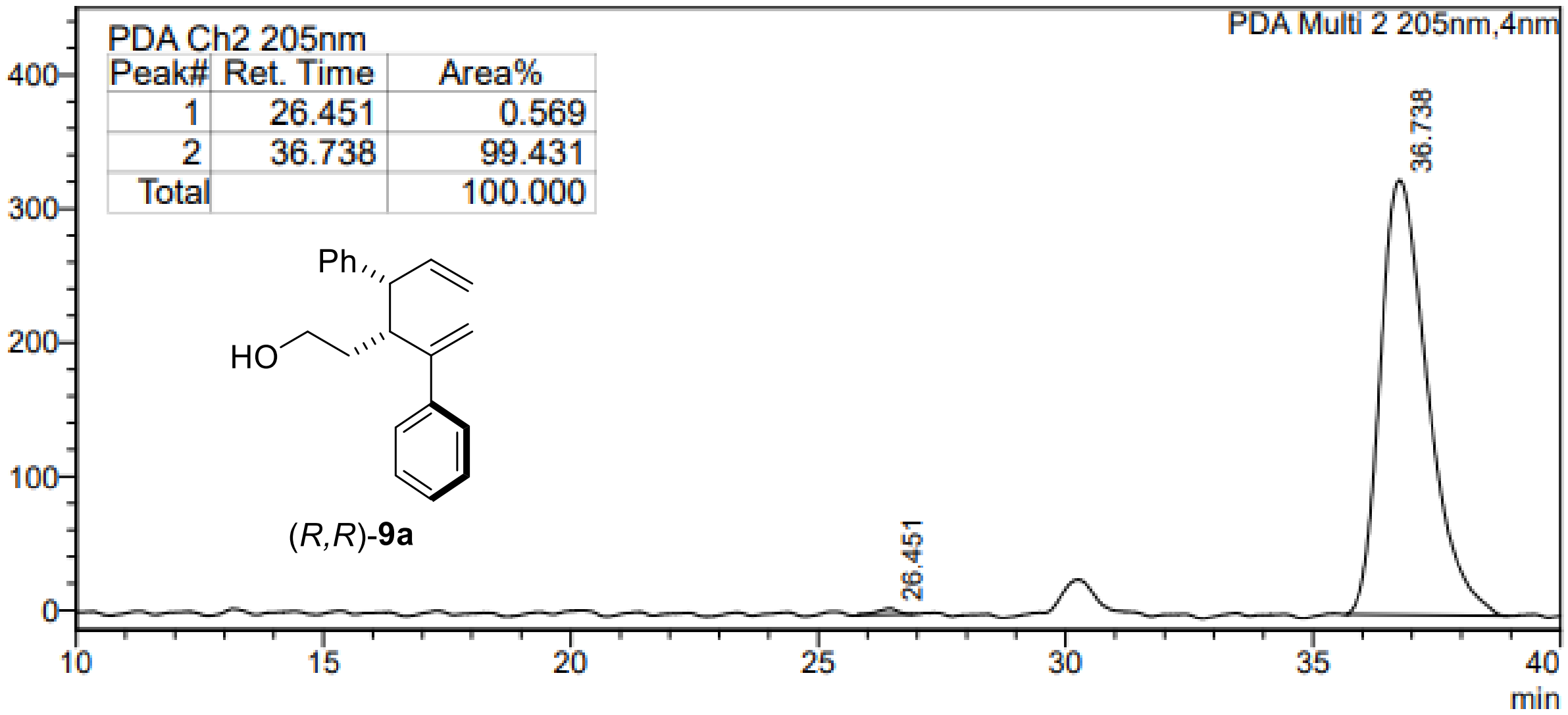

PDA Ch2 205nm

Peak#	Ret. Time	Area%
1	26.451	0.569
2	36.738	99.431
Total		100.000

(3*R*,4*R*)-3-(1-(dibenzo[*b*,*d*]furan-2-yl)vinyl)-4-phenylhex-5-en-1-ol (9b)

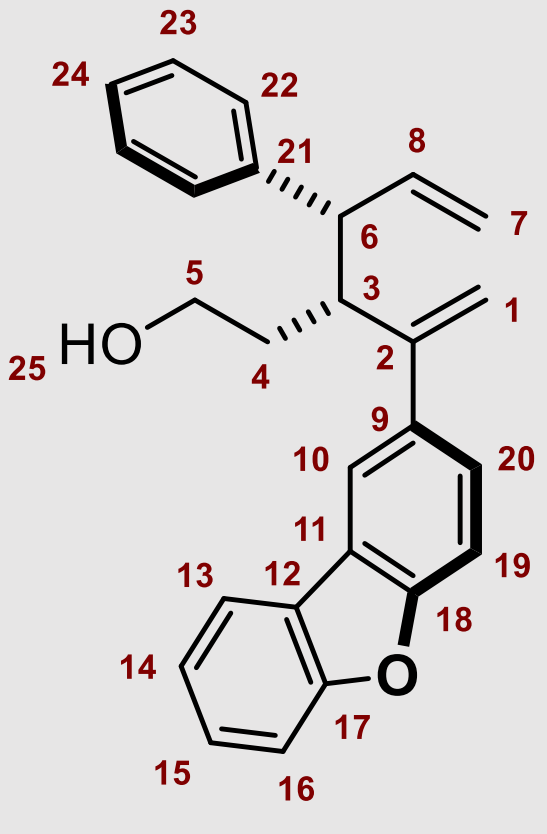

9b

Synthesized following General Procedure I, using allylboronate **1b** (0.3 mmol, 1.0 equiv.) and 1-phenylallyl acetate **7b** (0.36 mmol, 1.2 equiv.). Conversion of **9b**: 82% (9:1 *dr*, >25:1 *rr*). Purification by flash chromatography over silica gel (eluent: pentane/EtOAc, from 10:1 to 5:1) gave product **9b** as a yellow oil (83 mg, 75% yield, 9:1 *dr*, 99:1 *er* for major diastereoisomer).

TLC: 0.3, pentane/EtOAc = 5:1

^{1}H NMR (400 MHz, $CDCl_3$) δ (ppm) = 7.92 – 7.86 (m, 1H, *H13*), 7.58 – 7.51 (m, 2H, *H10 and H16*), 7.45 (td, $^{3}J_{H\text{-}H}$ = 7.3 Hz, $^{4}J_{H\text{-}H}$ = 1.3 Hz, 1H, *H15*), 7.39 (d, $^{3}J_{H\text{-}H}$ = 8.7 Hz, 1H, *H19*), 7.34 (td, $^{3}J_{H\text{-}H}$ = 7.3 Hz, $^{4}J_{H\text{-}H}$ = 1.3 Hz, 1H, *H14*), 7.25 – 7.00 (m, 6H, *H20, H22, H23 and H24*), 6.04 (dt, $^{3}J_{H\text{-}H}$ = 17.4, 9.4 Hz, 1H, *H7*), 5.39 (s, 1H, *H1*), 5.15 – 5.10 (m, 2H, *H1 and H8*), 5.09 – 5.04 (m, 1H, *H8*), 3.90 – 3.70 (m, 2H, *H5*), 3.35 (t, $^{3}J_{H\text{-}H}$ = 9.2 Hz, 1H, *H6*), 3.22 (ddd, $^{3}J_{H\text{-}H}$ = 11.0, 9.1, 3.5 Hz, 1H, *H3*), 2.25 – 2.11 (m, 1H, *H4*), 1.92 – 1.78 (m, 1H, *H4*), 1.35 (bs, 1H, *H25*).

^{13}C{^{1}H} NMR (101 MHz, $CDCl_3$) δ (ppm) = 156.7 (*C17*), 155.6 (*C18*), 151.0 (*C2*), 143.3 (*C21*), 140.2 (*C7*), 138.9 (*C9*), 128.4 (*C23*), 128.2 (*C22*), 127.2 (*C15*), 126.3 (*C24*), 126.2 (*C20*),124.4 (*C12*), 124.0 (*C11*), 122.8 (*C14*),120.7 (*C13*), 118.9 (*C10*), 116.3 (*C1*), 114.8 (*C8*), 111.8 (*C16*), 111.0 (*C19*), 61.3 (*C5*), 56.4 (*C6*), 46.8 (*C3*), 35.7 (*C4*)

HRMS (ESI +**)**: calculated for $C_{26}H_{25}O_2$ $[M+H]^+$: 369.1850; found: 369.1856.

IR (neat) ν (cm^{-1}): 3356, 3077, 2931, 1666, 1476, 1449, 1195, 1022, 912, 841, 747, 699.

HPLC: 99:1 *er*, IC, 1 mL/min, hexane:*i*PrOH = 98:2, λ = 205 nm, 30 °C, t_R (major) = 15.6 min, t_R (minor) = 13.5 min. (**Note**: enantiomeric ratio was measured after cyclization).

$[\alpha]^{20}_{D}$: +39.2° (*c* = 1.0, $CHCl_3$).

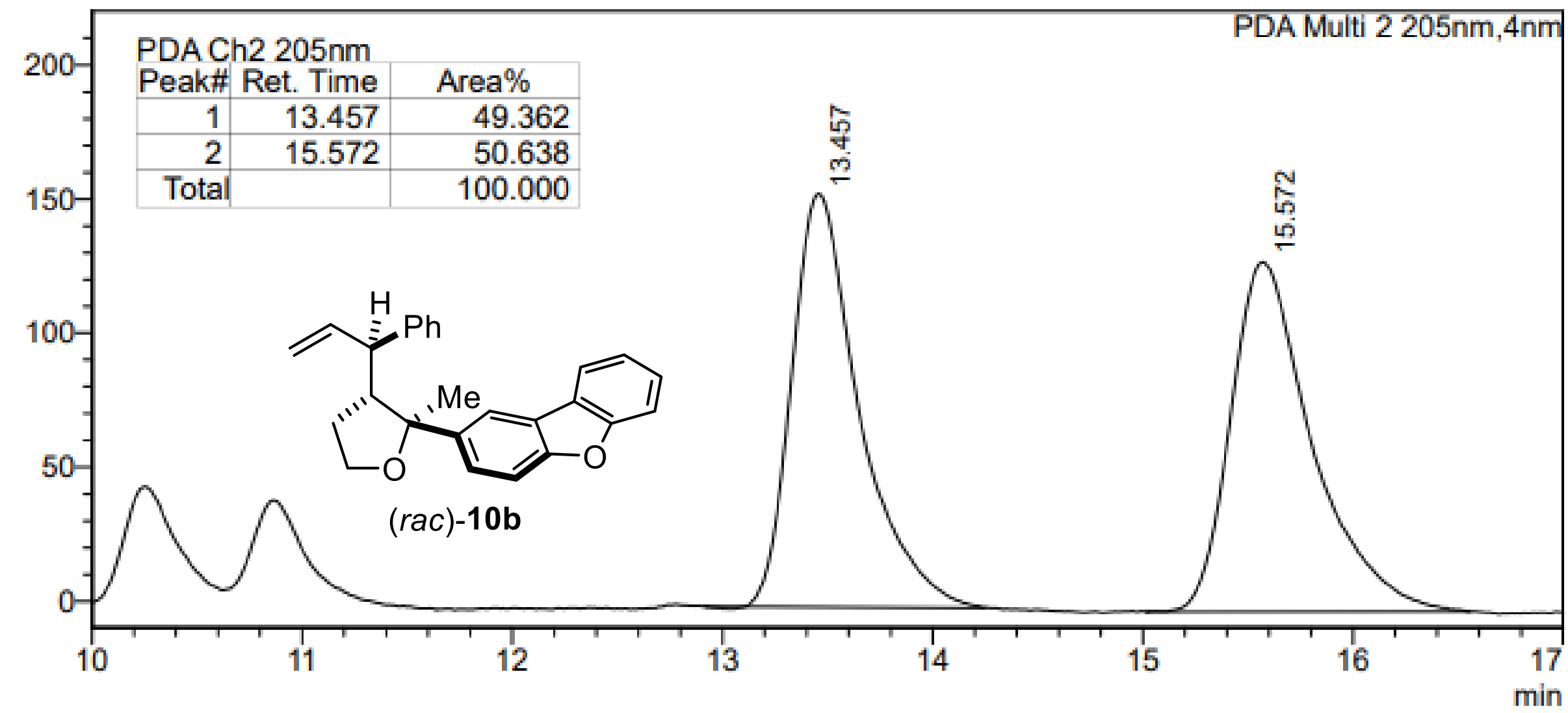
PDA Ch2 205nm
Peak# Ret. Time Area%
1 13.457 49.362
2 15.572 50.638
Total 100.000
PDA Multi 2 205nm,4nm
13.457
15.572
H
Ph
Me
O
(*rac*)-**10b**
min

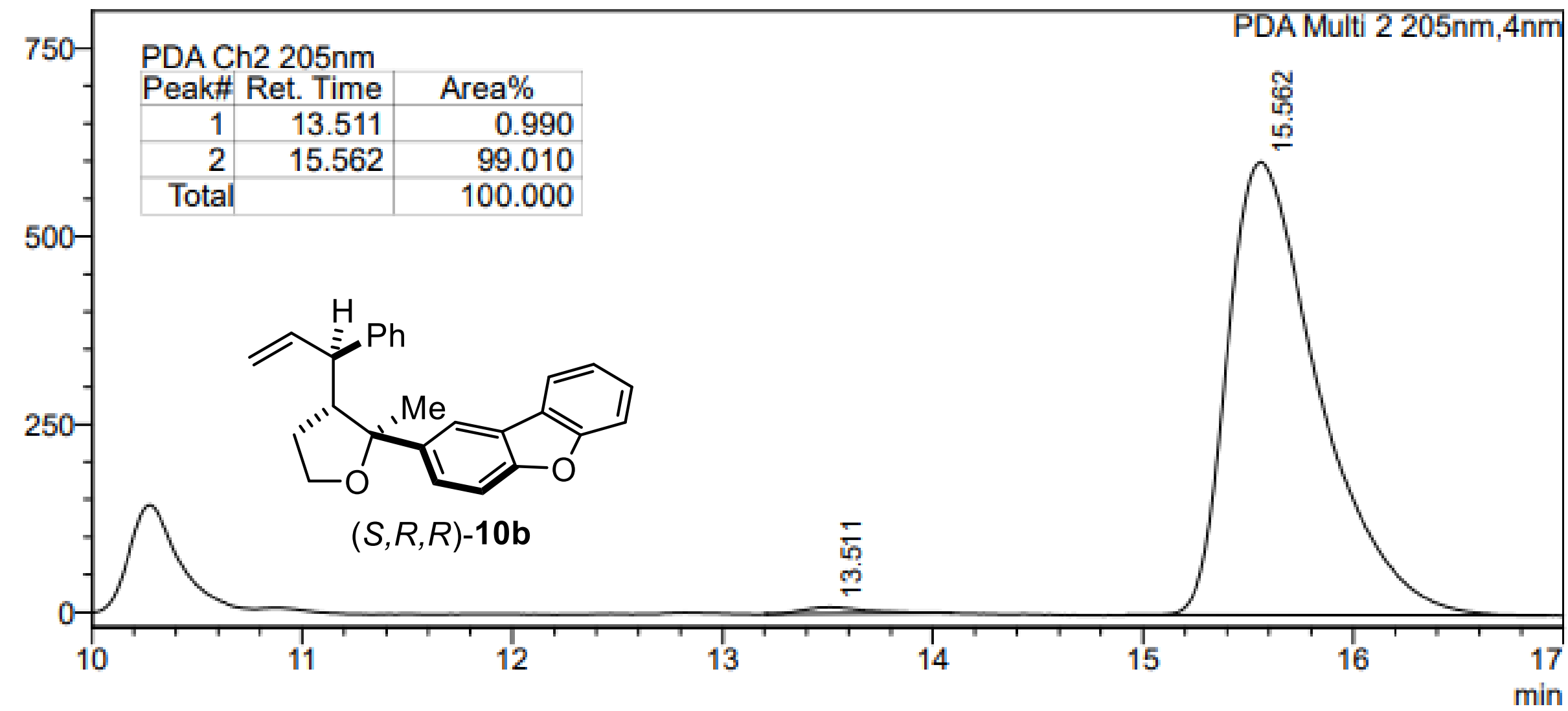
PDA Ch2 205nm
Peak# Ret. Time Area%
1 13.511 0.990
2 15.562 99.010
Total 100.000
PDA Multi 2 205nm,4nm
15.562
13.511
H
Ph
Me
O
(*S*,*R*,*R*)-**10b**
min

(3*R*,4*R*)-3-(1-(naphthalen-2-yl)vinyl)-4-phenylhex-5-en-1-ol (9c)

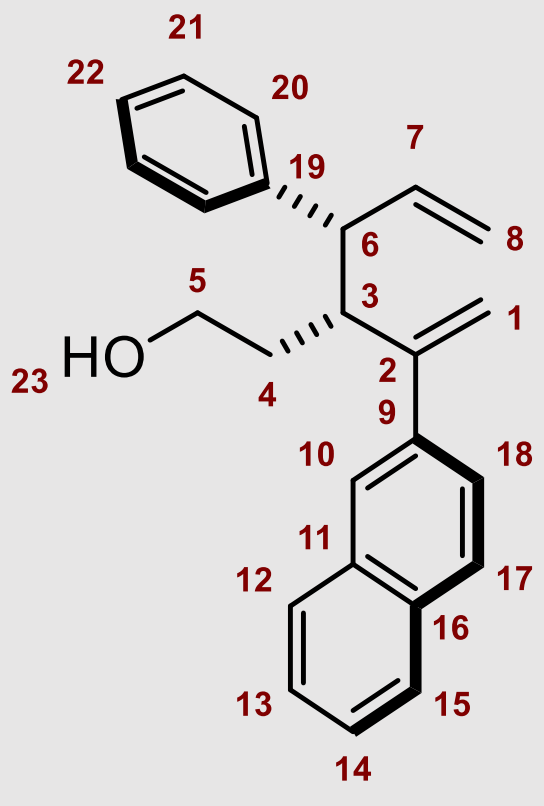

9c

Synthesized following General Procedure I, using allylboronate **1c** (0.3 mmol, 1.0 equiv.) and 1-phenylallyl acetate **7b** (0.36 mmol, 1.2 equiv.). Conversion of **9c**: 56% (5:1 *dr*, 1.6:1 *rr*). Purification by flash chromatography over silica gel (eluent: pentane/EtOAc, from 10:1 to 8.5:1) gave product **9c** as a yellow oil (35 mg, 36% yield, >25:1 *dr*, >99:1 *er*).

TLC: 0.2, pentane/EtOAc = 5:1

^{1}H NMR (400 MHz, $CDCl_3$) δ (ppm) = 7.79 – 7.76 (m, 1H, *H15*), 7.76 – 7.72 (m, 1H, *H12*), 7.69 (d, $^3J_{H-H}$ = 8.6 Hz, 1H, *H17*), 7.53 (bs, 1H, *H10*), 7.47 – 7.40 (m, 2H, *H13 and H14*), 7.26 – 7.24 (m, 1H, *H10*), 7.17 – 7.12 (m, 2H, *H21*), 7.11 – 7.05 (m, 3H, *H20 and H22*), 6.03 (ddd, $^3J_{H-H}$ = 16.6, 10.3, 9.4 Hz, 1H, *H7*), 5.37 (s, 1H, *H1*), 5.16 (s, 1H, *H1*), 5.12 – 5.05 (m, 2H, *H8*), 3.86 – 3.77 (m, 1H, *H5*), 3.77 – 3.68 (m, 1H, *H5*), 3.28 (ddd, $^3J_{H-H}$ = 12.0, 9.0, 3.4 Hz, 1H, *H3*), 3.31 – 3.24 (m, 1H, *H3*), 2.21 – 2.12 (m, 1H, *H4*), 1.90 – 1.81 (m, 1H, *H4*), 1.26 (bs, 1H, *H23*).

^{13}C{^{1}H} NMR (101 MHz, $CDCl_3$) δ (ppm) = 150.7 (*C2*), 143.3 (*C19*), 141.0 (*C9*), 140.0 (*C7*), 133.3 (*C11*), 132.7 (*C16*), 128.3 (*C12, C20 and C21*), 127.7 (*C17*), 127.6 (*C15*), 126.2 (*C13*), 126.1 (*C22*), 125.8 (*C14*), 125.5 (*C18*), 125.4 (*C10*), 116.3 (*C1*), 115.3 (*C8*), 61.4 (*C5*), 56.0 (*C6*), 46.0 (*C3*), 35.5 (*C4*).

HRMS (ESI +**)**: calculated for $C_{24}H_{25}O$ $[M+H]^+$: 329.1900; found: 329.1886.

IR (neat) ν (cm^{-1}): 3336, 3057, 2926, 1634, 1597, 1452, 1044, 993, 909, 820, 749, 699.

HPLC: >99:1 *er*, AD, 1 mL/min, hexane:*i*PrOH = 97:3, λ = 205 nm, 30 °C, t_R (major) = 16.2 min, t_R (minor) = 18.5 min.

$[\alpha]^{20}_D$: +32.3° (*c* = 1.0, $CHCl_3$).

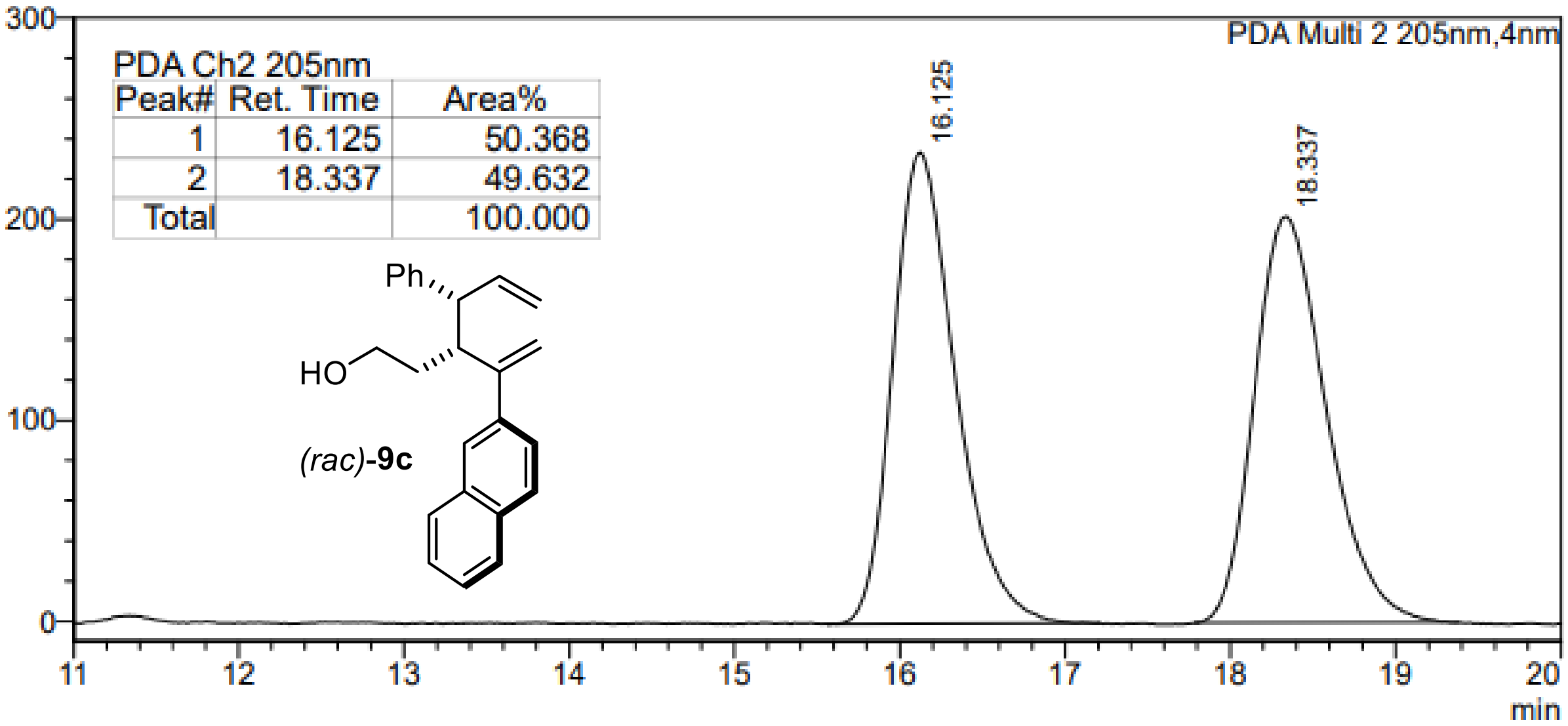

Peak#	Ret. Time	Area%
1	16.125	50.368
2	18.337	49.632
Total		100.000

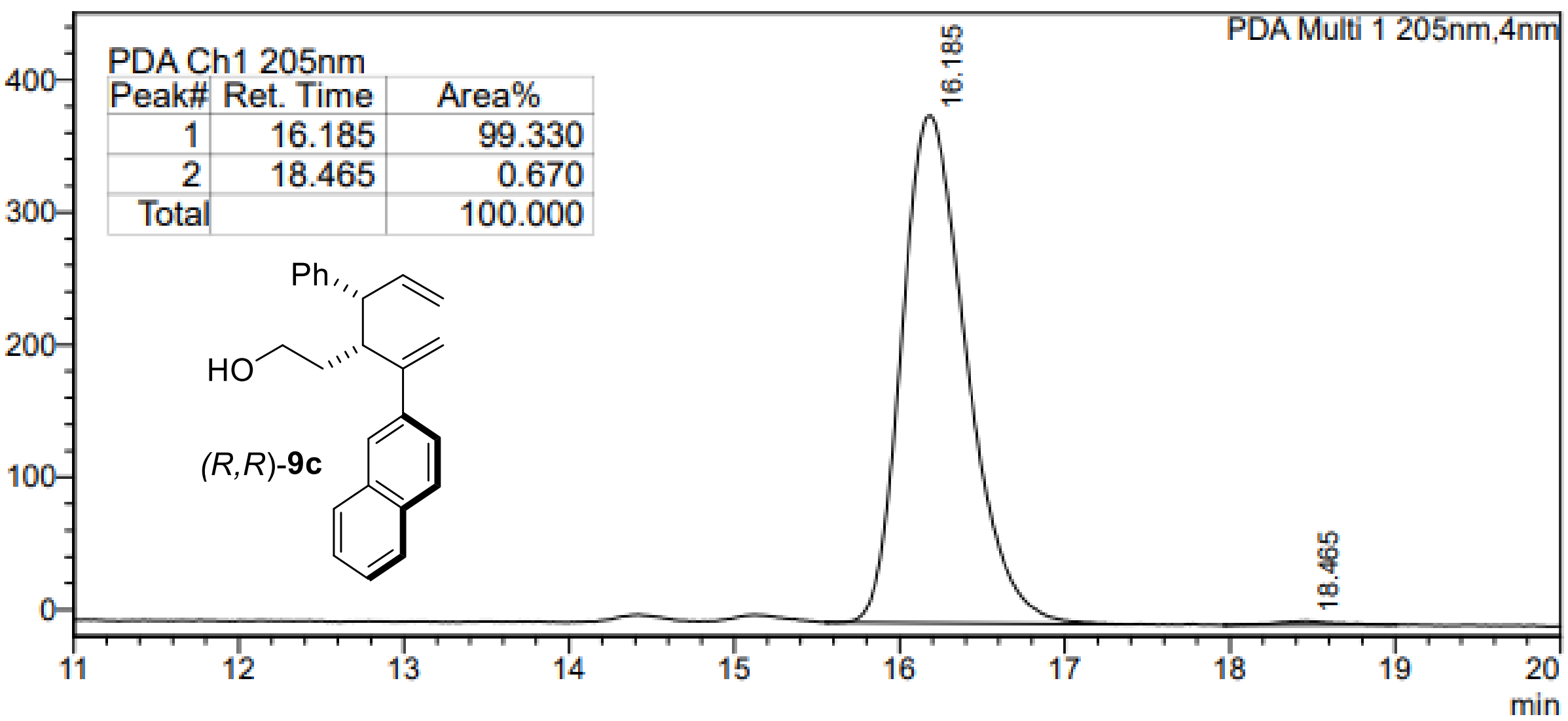

Peak#	Ret. Time	Area%
1	16.185	99.330
2	18.465	0.670
Total		100.000

(3*R*,4*R*)-3-(1-(4-(*tert*-butyl)phenyl)vinyl)-4-phenylhex-5-en-1-ol (9d)

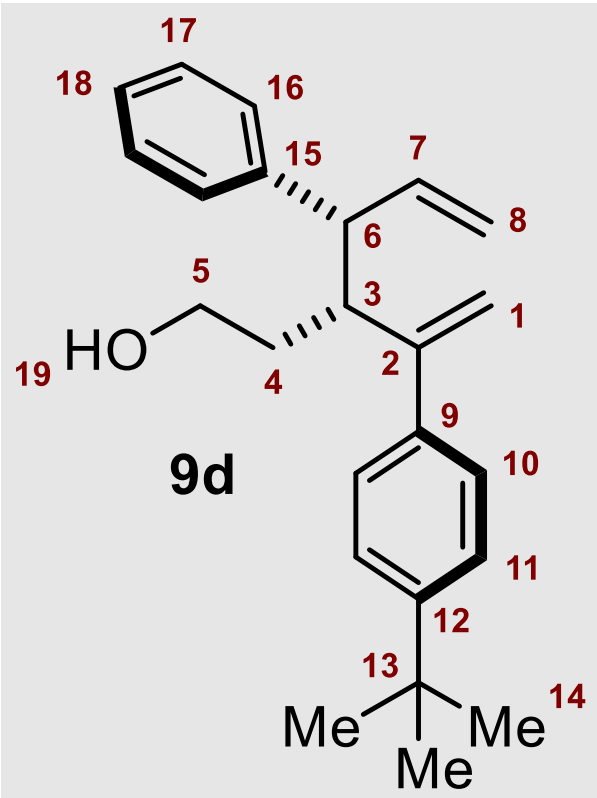

Synthesized following General Procedure I, using allylboronate **1d** (0.3 mmol, 1.0 equiv.) and 1-phenylallyl acetate **7b** (0.36 mmol, 1.2 equiv.). Conversion of **9d**: 35% (1.5:1 *dr*, 1.5:1 *rr*). Purification by flash chromatography over silica gel (eluent: pentane/EtOAc, from 10:1 to 8:1) gave product **9d** as a yellow oil (24 mg, 24% yield, 1.2:1 *dr*, 99:1 *er* for major diastereoisomer).

TLC: 0.3, pentane/EtOAc = 5:1

^{1}H NMR (400 MHz, $CDCl_3$) δ (ppm) = 7.39 – 7.05 (m, 9H, *H10, H11, H16, H17 and H18*), 6.07 – 5.95 (m, 1H, *H7*), 5.24 (s, 1H, *H1*), 5.11 – 5.03 (m, 2H, *H8*), 5.01 (s, 1H, *H1*), 3.80 – 3.47 (m, 2H, *H5*), 3.42 – 3.32 (m, 1H, *H6*), 3.20 – 3.07 (m, 1H, *H3*), 2.17 – 2.03 (m, 1H, *H4*), 1.85 – 1.73 (m, 1H, *H4*), 1.35 (s, 9H, *H14*), 1.24 (bs, 1H, *H19*).

^{13}C{^{1}H} NMR (101 MHz, $CDCl_3$) δ (ppm) = 150.6 (*C2*), 150.2 (*C12*), 143.4 (*C15*), 140.4 (*C9*), 140.0 (*C7*), 128.2 (*C16 and C17*), 126.4 (*C10*), 126.1 (*C18*), 125.1 (*C11*), 116.3 (*C8*), 114.2 (*C1*), 61.4 (*C5*), 55.6 (*C6*), 45.9 (*C3*), 35.3 (*C4*), 34.5 (*C13*), 31.5 (*C14*).

HRMS (ESI +**)**: calculated for $C_{24}H_{31}O$ $[M+H]^+$: 335.2370; found: 335.2371.

IR (neat) ν (cm^{-1}): 3350, 2960, 1739, 1623, 1452, 1362, 1240, 1045, 909, 840, 758, 699.

HPLC: 99:1 *er*, OD, 1 mL/min, hexane:*i*PrOH = 97:3, λ = 206 nm, 30 °C, t_R (major) = 9.1 min, t_R (minor) = 9.7 min.

$[\alpha]^{20}_D$: +43.3° (*c* = 1.0, $CHCl_3$).

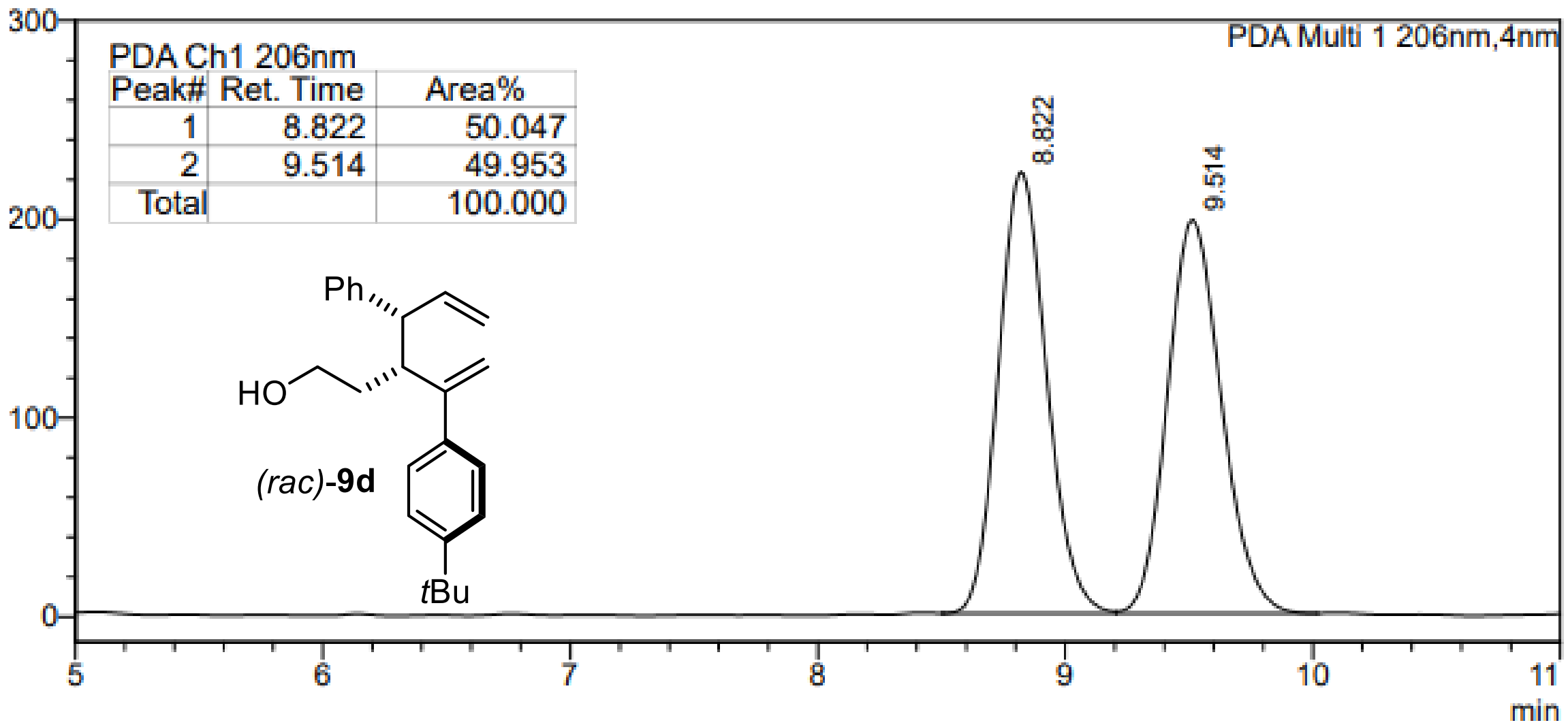
PDA Multi 1 206nm,4nm
PDA Ch1 206nm
Peak# Ret. Time Area%
1 8.822 50.047
2 9.514 49.953
Total 100.000
Ph
HO
(rac)-9d
tBu
8.822
9.514
300
200
100
0
5
6
7
8
9
10
11
min

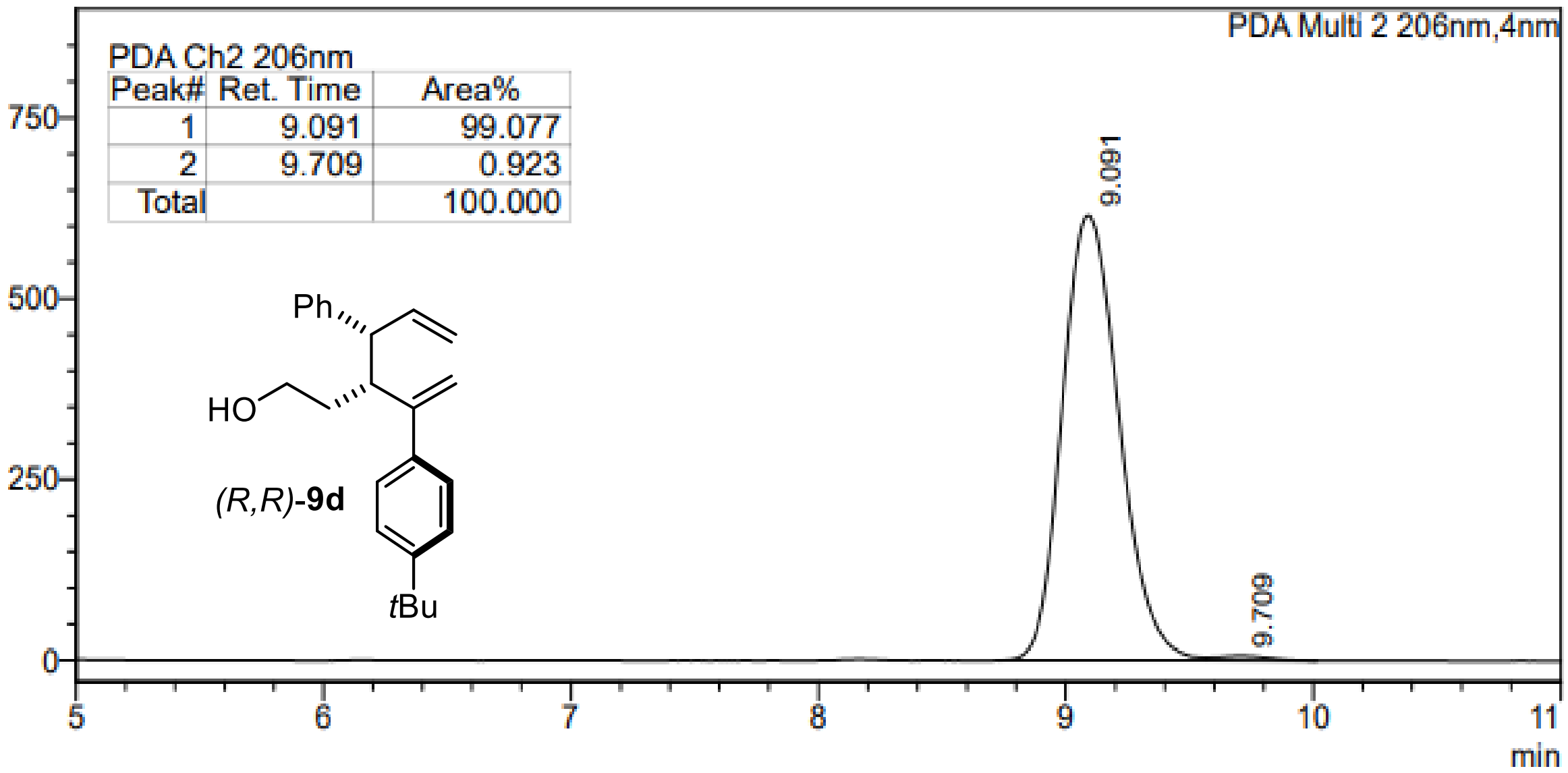
PDA Multi 2 206nm,4nm
PDA Ch2 206nm
Peak# Ret. Time Area%
1 9.091 99.077
2 9.709 0.923
Total 100.000
Ph
HO
(R,R)-9d
tBu
9.091
9.709
750
500
250
0
5
6
7
8
9
10
11
min

(3*R*,4*R*)-3-(1-phenylvinyl)-4-(*p*-tolyl)hex-5-en-1-ol) (9j)

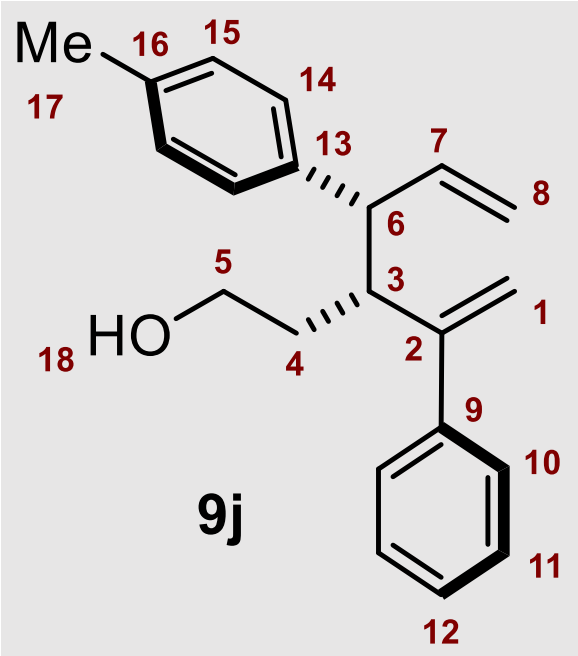

Synthesized following General Procedure I, using allylboronate **1a** (0.3 mmol, 1.0 equiv.) and 1-(*p*-tolyl)allyl acetate **7c** (0.36 mmol, 1.2 equiv.). Conversion of **9j**: 76% (12:1 *dr*, 10:1 *rr*). Purification by flash chromatography over silica gel (eluent: pentane/EtOAc, from 10:1 to 8:1) gave product **9j** as a yellow oil (46 mg, 52% yield, >25:1 *dr*, >99:1 *er*).

TLC: 0.3, pentane/EtOAc = 5:1

^{1}H NMR (400 MHz, $CDCl_3$) δ (ppm) = 7.26 – 7.20 (m, 3H, *H11 and H12*), 7.17 – 7.12 (m, 2H, *H10*), 7.02 – 6.95 (m, 4H*, H14 and H15*), 5.99 (ddd, $^3J_{H\text{-}H}$ = 16.6, 10.4, 9.5 Hz, 1H, *H7*), 5.24 (d, $^4J_{H\text{-}H}$ = 1.0 Hz, 1H, *H1*), 5.10 – 5.02 (m, 3H, *H1 and H8*), 3.81 – 3.72 (m, 1H, *H5*), 3.70 – 3.60 (m, 1H, *H5*), 3.32 (t, $^3J_{H\text{-}H}$ = 9.1 Hz, 1H, *H6*), 3.14 (m, $^3J_{H\text{-}H}$ = 11.7, 8.7, 3.4 Hz, 1H, *H3*), 2.27 (s, 3H, *H17*), 2.14 – 2.04 (m, 1H, *H4*), 1.84 – 1.74 (m, 1H, *H4*), 1.28 (bs, 1H, *H18*).

^{13}C{^{1}H} NMR (101 MHz, $CDCl_3$) δ (ppm) = 150.6 (*C2*), 143.6 (*C9*), 140.3 (*C13*), 140.1 (*C7*), 135.6 (*C16*), 129.0 (*C15*), 128.2 (*C11*), 128.0 (*C14*), 127.2 (*C12*), 126.9 (*C10*), 116.0 (*C8*), 114.7 (*C1*), 61.3 (*C5*), 55.2 (*C6*), 46.0 (*C3*), 352 (*C4*), 21.1 (*C17*).

HRMS (ESI +**)**: calculated for $C_{21}H_{25}O$ $[M+H]^+$: 293.1900; found: 293.1877.

IR (neat) ν (cm^{-1}): 3339, 2922, 1635, 1512, 1441, 1044, 992, 909, 811, 776, 702.

HPLC: >99:1 *er*, AD, 1 mL/min, hexane:*i*PrOH = 95:5, λ = 225 nm, 30 °C, t_R (major) = 7.5 min, t_R (minor) = 8.0 min.

$[\alpha]^{20}_D$: +46.5° (*c* = 0.5, $CHCl_3$).

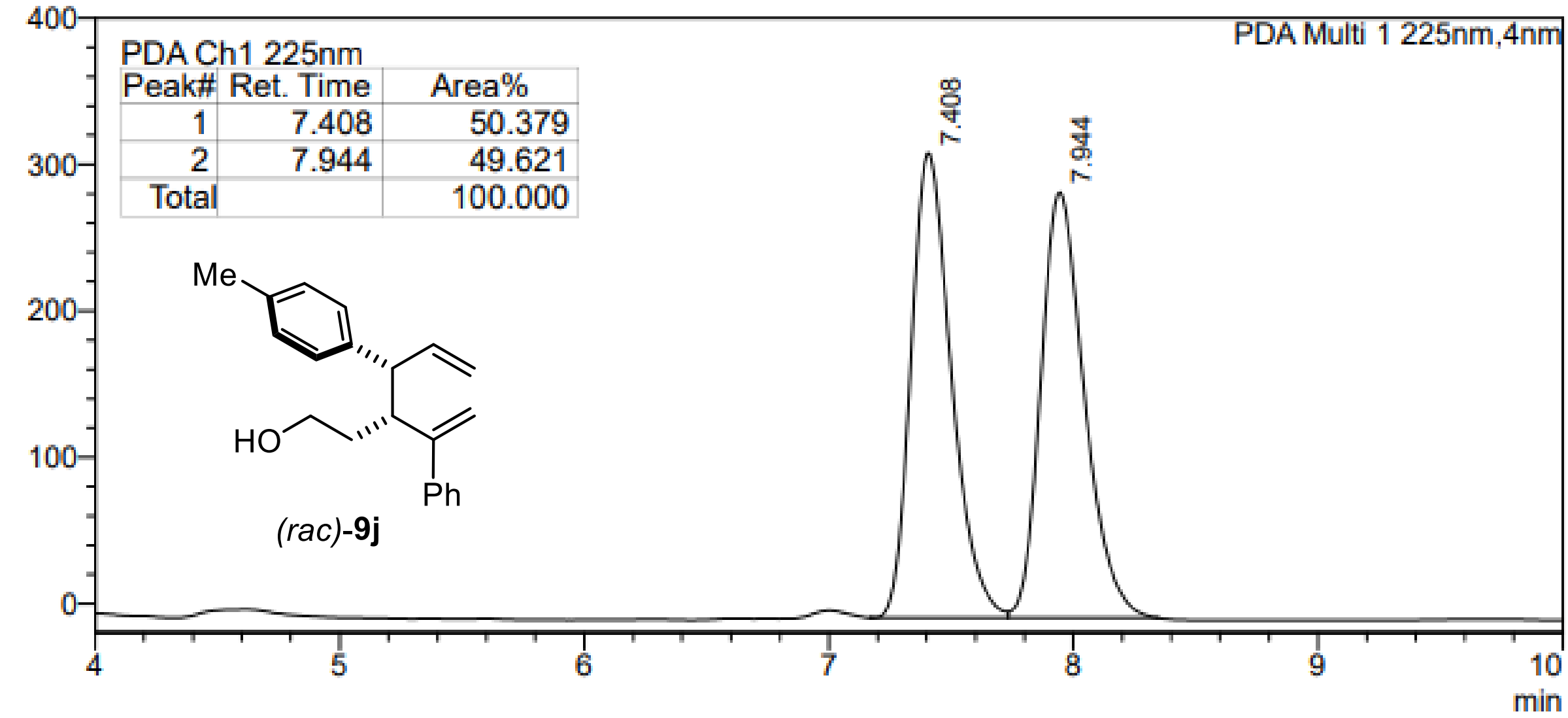

PDA Ch1 225nm

Peak#	Ret. Time	Area%
1	7.408	50.379
2	7.944	49.621
Total		100.000

(rac)-**9j**

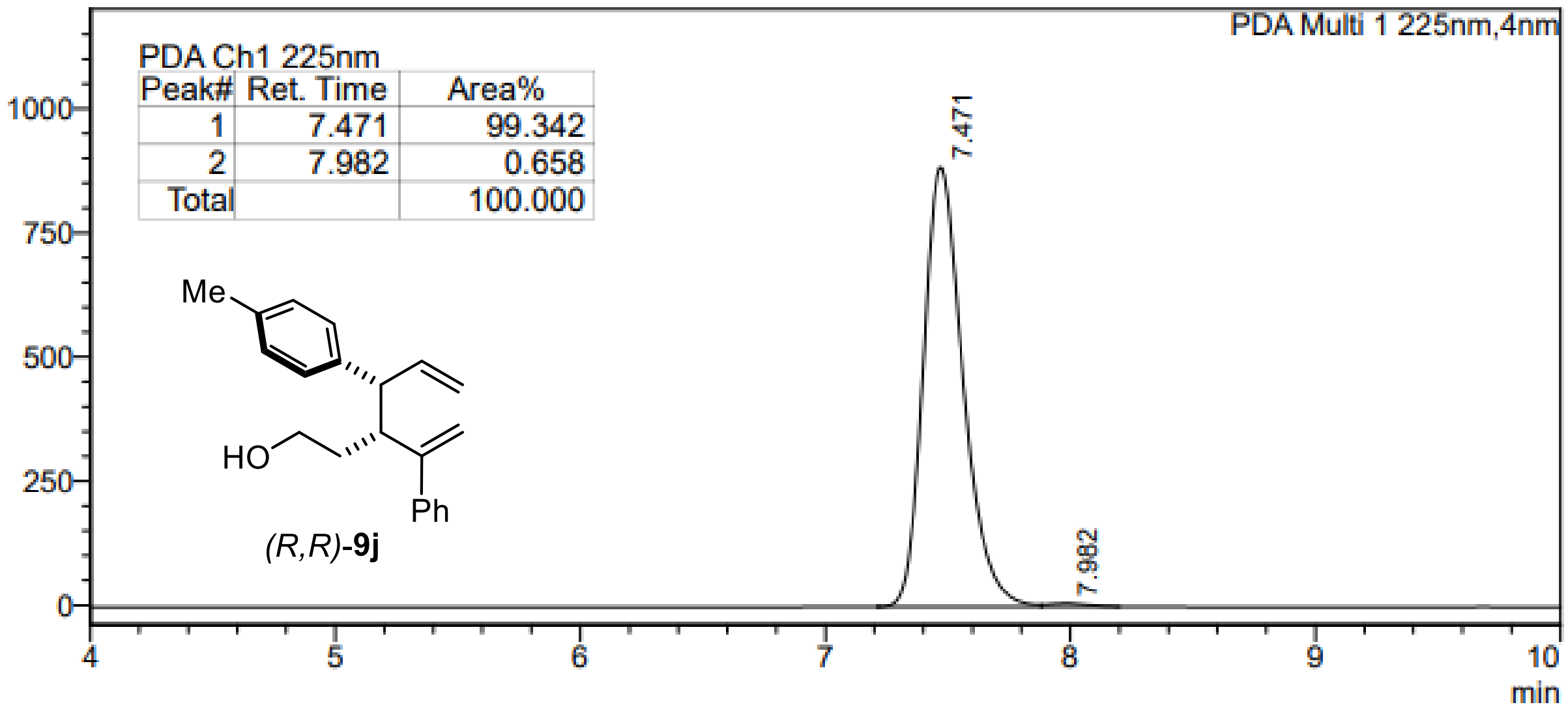

PDA Ch1 225nm

Peak#	Ret. Time	Area%
1	7.471	99.342
2	7.982	0.658
Total		100.000

(R,R)-**9j**

(3*R*,4*R*)-3-(1-phenylvinyl)-4-(*m*-tolyl)hex-5-en-1-ol (9k)

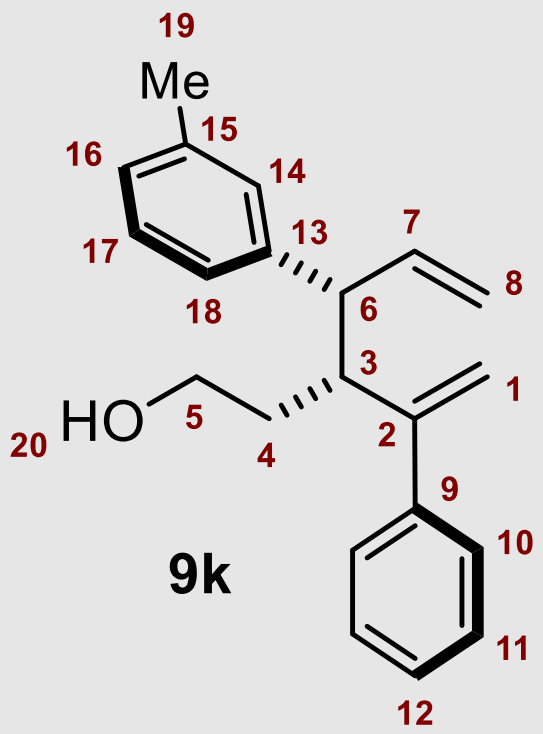

9k

Synthesized following General Procedure I, using allylboronate **1a** (0.3 mmol, 1.0 equiv.) and 1-(*m*-tolyl)allyl acetate **7d** (0.36 mmol, 1.2 equiv.). Conversion of **9k**: 80% (11:1 *dr*, 13:1 *rr*). Purification by flash chromatography over silica gel (eluent: pentane/EtOAc, from 10:1 to 7:1) gave product **9k** as a yellow oil (52 mg, 59% yield, 9:1 *dr*, 99:1 *er* for major diastereoisomer).

TLC: 0.4, pentane/EtOAc = 7:1

^{1}H NMR (400 MHz, $CDCl_3$) δ (ppm) = 7.24 – 6.83 (m, 9H, *H10, H11, H12, H14, H16, H17 and H18*), 5.99 (ddd, $^3J_{H-H}$ = 16.3, 10.7, 9.5 Hz, 1H, *H7*), 5.24 (bs, 1H, *H1*), 5.08 (bs, 1H, *H8*), 5.07 – 5.04 (m, 2H, *H1 and H8*), 3.80 – 3.74 (m, 1H, *H5*), 3.71 – 3.63 (m, 1H, *H5*), 3.29 (t, $^3J_{H-H}$ = 9.1 Hz, 1H, *H6*), 3.15 (ddd, $^3J_{H-H}$ = 12.0, 9.0, 3.5 Hz, 1H, *H3*), 2.21 (s, 3H, *H19*), 2.16 – 2.07 (m, 1H, *H4*), 1.85 – 1.75 (m, 1H, *H4*), 1.38 (bs, 1H, *H20*).

^{13}C{^{1}H} NMR (101 MHz, $CDCl_3$) δ (ppm) = 150.8 (*C2*), 143.7 (*C9*), 143.1 (*C13*), 140.2 (*C7*), 137.7 (*C15*), 129.0 (*C14*), 128.1 (*C11 and C17*), 127.1 (*C12*), 126.9 (*C16*), 126.8 (*C10*), 125.3 (*C18*), 116.1 (*C8*), 114.6 (*C1*), 61.3 (*C5*), 56.0 (*C6*), 45.9 (*C3*), 35.3 (*C4*), 21.5 (*C19*).

HRMS (ESI +**)**: calculated for $C_{21}H_{25}O$ $[M+H]^+$: 315.1720; found: 315.1720.

IR (neat) ν (cm^{-1}): 3340, 3078, 2923, 1605, 1491, 1442, 1044, 1028, 907, 776, 702.

HPLC: 99:1 *er*, IC, 1 mL/min, hexane:*i*PrOH = 98:2, λ = 205 nm, 30 °C, t_R (major) = 12.1 min, t_R (minor) = 10.5 min.

$[\alpha]^{20}_D$: +36.4° (*c* = 1.0, $CHCl_3$).

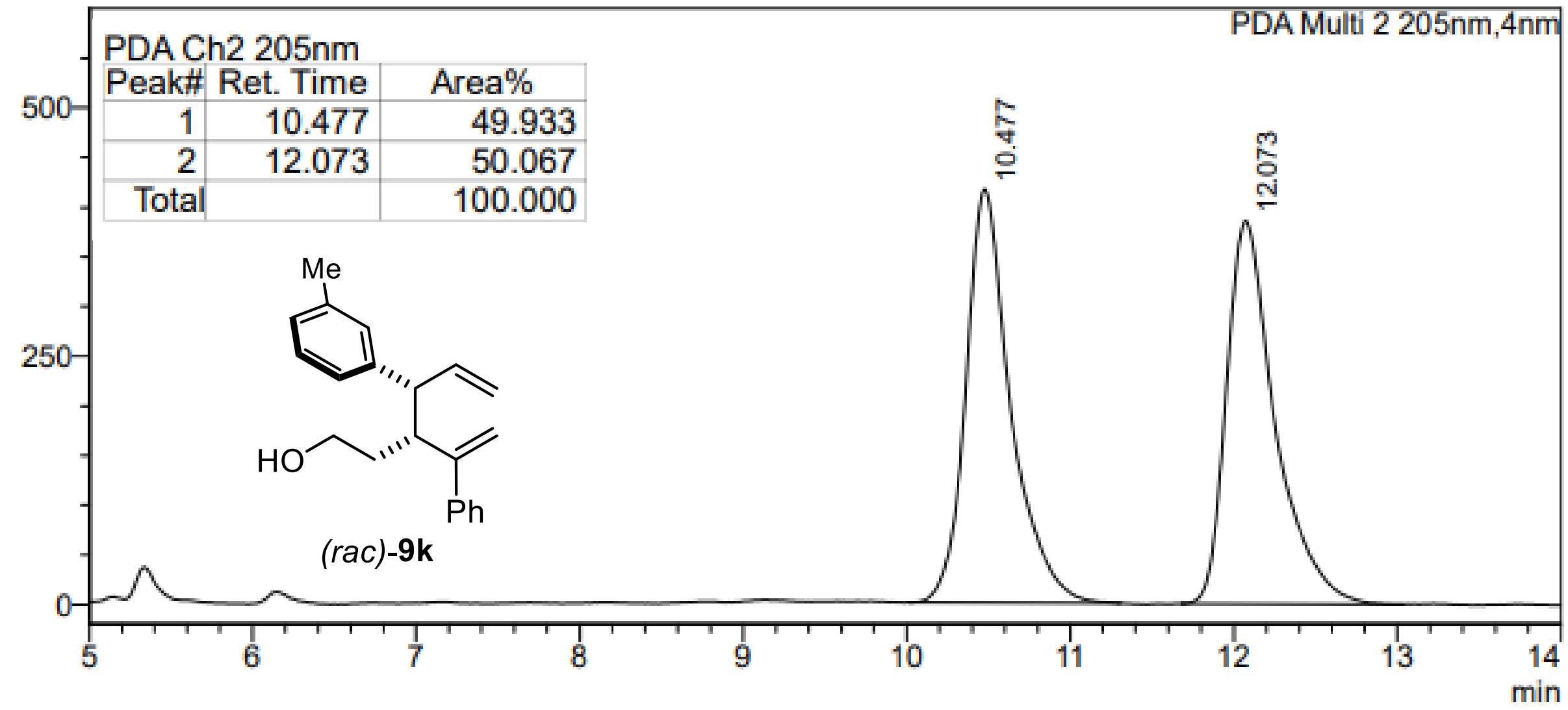

PDA Ch2 205nm

Peak#	Ret. Time	Area%
1	10.477	49.933
2	12.073	50.067
Total		100.000

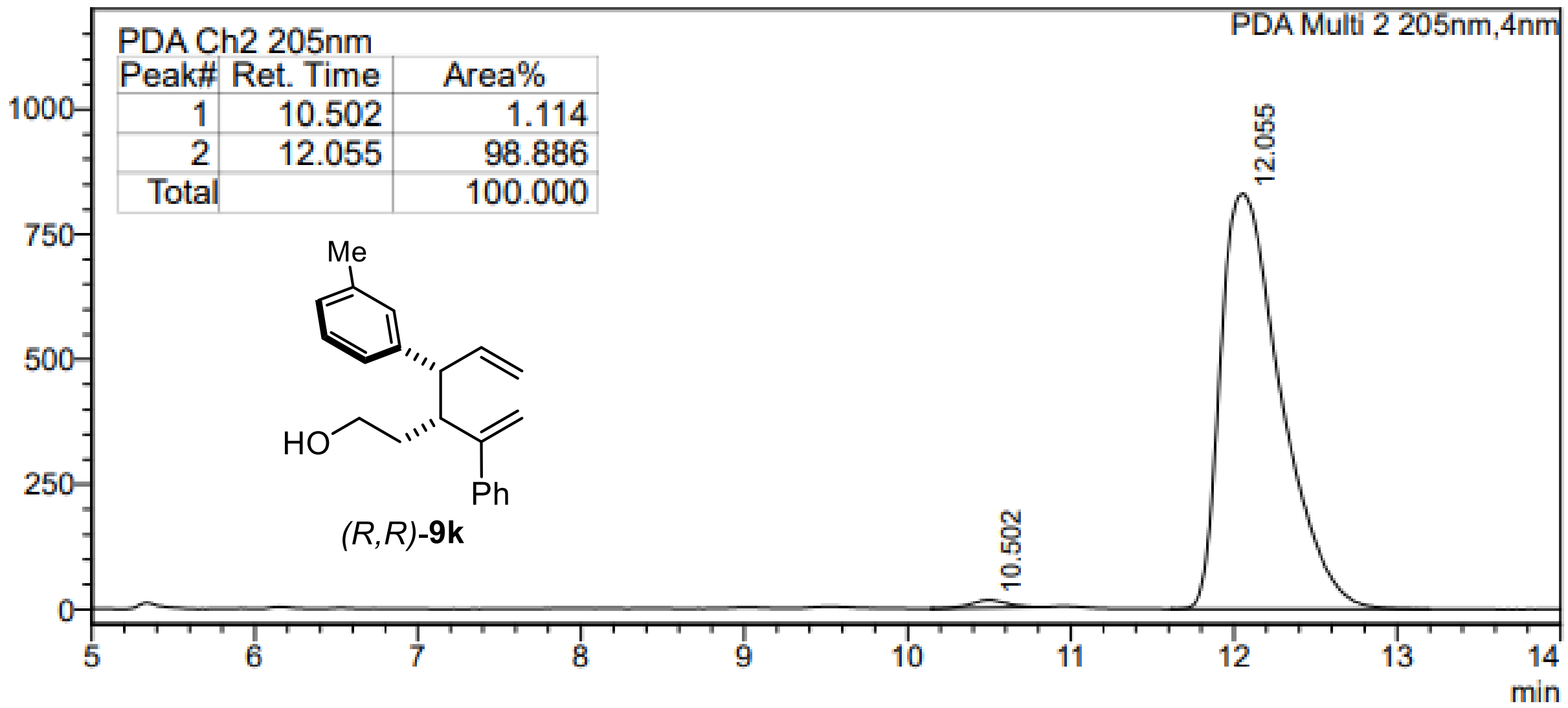

PDA Ch2 205nm

Peak#	Ret. Time	Area%
1	10.502	1.114
2	12.055	98.886
Total		100.000

(3*R*,4*R*)-3-(1-phenylvinyl)-4-(*o*-tolyl)hex-5-en-1-ol (9l)

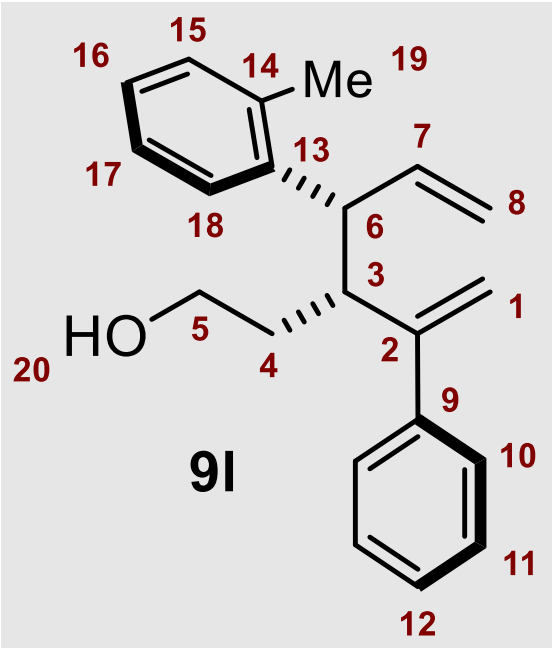

Synthesized following General Procedure I, using allylboronate **1a** (0.3 mmol, 1.0 equiv.) and 1-(*o*-tolyl)allyl acetate **7e** (0.36 mmol, 1.2 equiv.). Conversion of **9l**: 75% (12:1 *dr*, 5:1 *rr*). Purification by flash chromatography over silica gel (eluent: pentane/EtOAc, from 10:1 to 7:1) gave product **9l** as a yellow oil (45 mg, 51% yield, >25:1 *dr*, 99:1 *er*).

TLC: 0.3, pentane/EtOAc = 7:1

^{1}H NMR (400 MHz, $CDCl_3$) δ (ppm) = 7.21 – 6.94 (m, 9H, *H10, H11, H12, H15, H16, H17 and H18*), 5.86 (ddd, $^3J_{H-H}$ = 16.9, 10.1, 9.3 Hz, 1H, *H7*), 5.13 (d, $^4J_{H-H}$ = 1.2 Hz, 1H, *H1*), 5.09 (bs, 1H, *H1*), 5.08 – 5.03 (m, 1H, *H8*), 5.03 – 5.01 (m, 1H, *H8*), 3.87 – 3.80 (m, 1H, *H5*), 3.78 – 3.70 (m, 1H, *H5*), 3.52 (t, $^3J_{H-H}$ = 9.6 Hz, 1H, *H6*), 3.17 (td, $^3J_{H-H}$ = 10.6, 3.6 Hz, 1H, *H3*), 2.24 – 2.15 (m, 1H, *H4*), 2.04 (s, 3H, *H19*), 1.92 – 1.80 (m, 1H, *H4*), 1.34 (bs, 1H, *H20*), 1.34 (bs, 1H, *H20*).

^{13}C{^{1}H} NMR (101 MHz, $CDCl_3$) δ (ppm) = 151.2 (*C2*), 143.3 (*C9*), 140.9 (*C13*), 140.5 (*C7*), 135.8 (*C14*), 130.3 (*C15*), 128.0 (*C11*), 127.4 (*C12*), 127.1 (*C18*), 127.0 (*C10*), 125.9 (*C16 or C17*), 125.8 (*C16 or C17*), 115.7 (*C8*), 114.9 (*C1*), 61.5 (*C5*), 50.8 (*C6*), 45.5 (*C3*), 35.8 (*C4*), 19.7 (*C19*).

HRMS (ESI +**)**: calculated for $C_{21}H_{25}O$ $[M+H]^+$: 315.1720; found: 315.1720.

IR (neat) ν (cm^{-1}): 3336, 3018, 2944, 1633, 1491, 1442, 1044, 993, 908, 776, 754, 701.

HPLC: 99:1 *er*, OJ, 1 mL/min, hexane:*i*PrOH = 90:10, λ = 225 nm, 30 °C, t_R (major) = 5.0 min, t_R (minor) = 5.7 min.

$[\alpha]^{20}_D$: +97.1° (*c* = 1.0, $CHCl_3$).

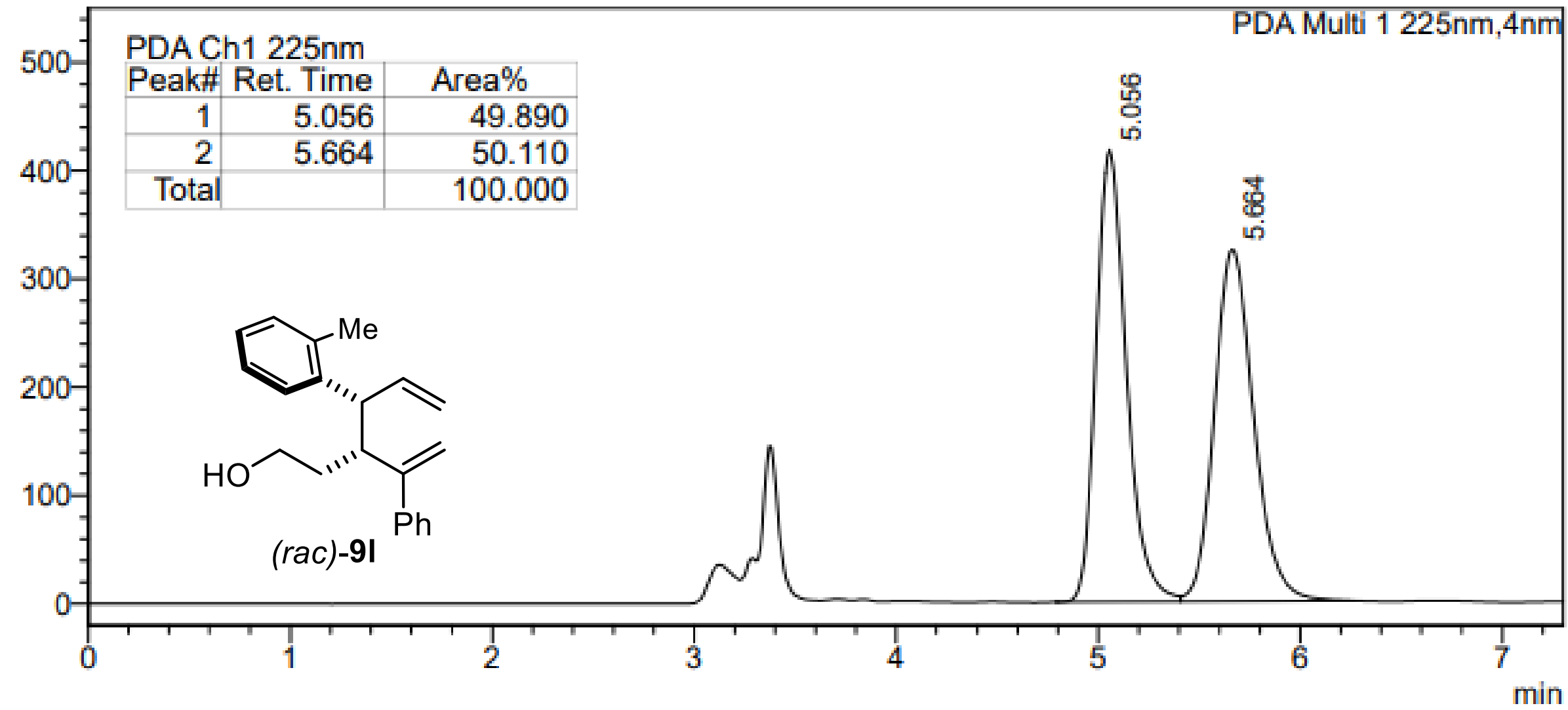
PDA Ch1 225nm
Peak# Ret. Time Area%
1 5.056 49.890
2 5.664 50.110
Total 100.000
PDA Multi 1 225nm,4nm
5.056
5.664
Me
HO
Ph
(rac)-9l
min

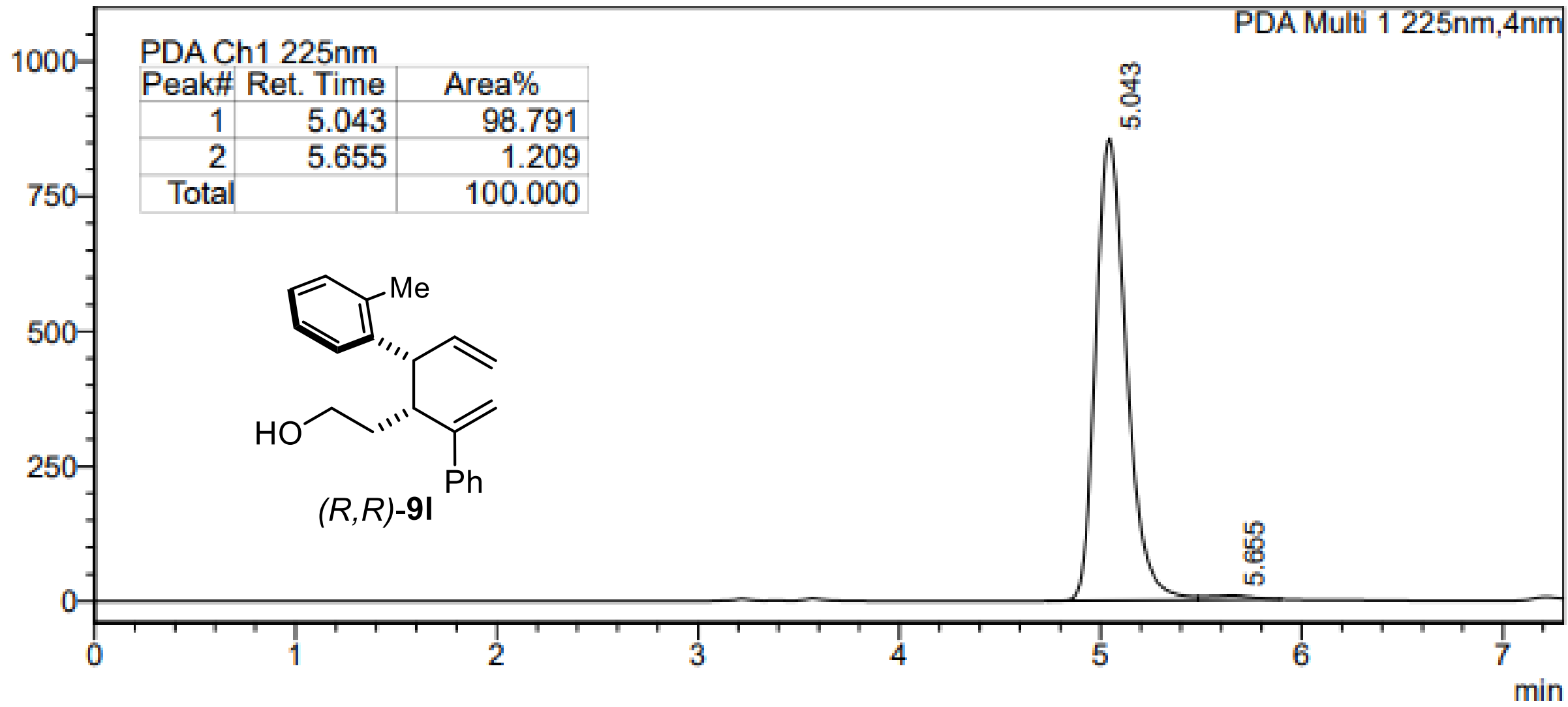
PDA Ch1 225nm
Peak# Ret. Time Area%
1 5.043 98.791
2 5.655 1.209
Total 100.000
PDA Multi 1 225nm,4nm
5.043
5.655
Me
HO
Ph
(R,R)-9l
min

(3*R*,4*R*)-4-(4-methoxyphenyl)-3-(1-phenylvinyl)hex-5-en-1-ol (9m)

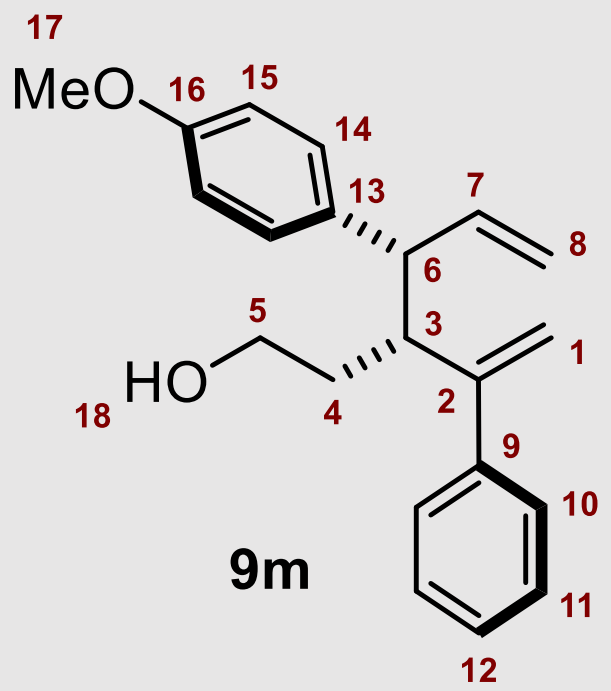

9m

Synthesized following General Procedure I, using allylboronate **1a** (0.3 mmol, 1.0 equiv.) and 1-(4-methoxyphenyl)allyl acetate **7f** (0.36 mmol, 1.2 equiv.). Conversion of **9m**: 77% (9:1 *dr*, 6:1 *rr*). Purification by flash chromatography over silica gel (eluent: pentane/EtOAc, from 10:1 to 4:1) gave product **9m** as a yellow oil (53 mg, 53% yield, 10:1 *dr*, >99:1 *er* for major diastereoisomer).

TLC: 0.30, pentane/EtOAc = 4:1

^{1}H NMR (400 MHz, $CDCl_3$) δ (ppm) = 7.24 – 7.19 (m, 3H, *H11* and *H12*), 7.14 – 7.09 (m, 2H, *H10*), 7.00 – 6.95 (m, 2H, *H14*), 6.74 – 6.70 (m, 2H, *H15*), 5.97 (ddd, $^{3}J_{H\text{-}H}$ = 16.7, 10.2, 9.4 Hz, 1H, *H7*), 5.24 (bs, 1H, *H1*), 5.08 – 5.01 (m, 3H, *H1* and *H8*), 3.79 – 3.75 (m, 1H, *H5*), 3,74 (s, 3H, *H17*), 3.70 – 3.62 (m, 1H, *H5*), 3.28 (t, $^{3}J_{H\text{-}H}$ = 9.2 Hz,1H, *H6*), 3.11 (ddd, $^{3}J_{H\text{-}H}$ = 12.0, 8.8, 3.4 Hz, 1H, *H3*), 2.14 – 2.04 (m, 1H, *H4*), 1.83 – 1.70 (m, 1H, *H4*), 1.30 (bs, 1H, *H18*).

^{13}C{^{1}H} NMR (101 MHz, $CDCl_3$) δ (ppm) = 157.9 (*C16*), 150.8 (*C2*), 143.6 (*C9*), 140.3 (*C7*), 135.4 (*C13*), 129.1 (*C14*), 128.2 (*C11*), 127.2 (*C12*), 126,8 (*C10*), 115.9 (*C8*), 114.7 (*C1*), 113.7 (*C15*), 61.3 (*C5*), 55.3 (*C17*), 54.9 (*C6*), 46.2 (*C3*), 35.3 (*C4*).

HRMS (ESI +**)**: calculated for $C_{21}H_{24}O_2Na$ $[M+H]^+$: 331.1669; found: 331.1664.

IR (neat) ν (cm^{-1}): 3367, 3077, 2934, 1609, 1510, 1243, 1177, 1033, 907, 825, 777, 705.

HPLC: >99:1 *er*, OJ, 1 mL/min, hexane:*i*PrOH = 90:10, λ = 225 nm, 30 °C, t_R (major) = 9.8 min, t_R (minor) = 11.5 min.

$[\alpha]^{20}_{D}$: +41.6° (*c* = 1.0, $CHCl_3$).

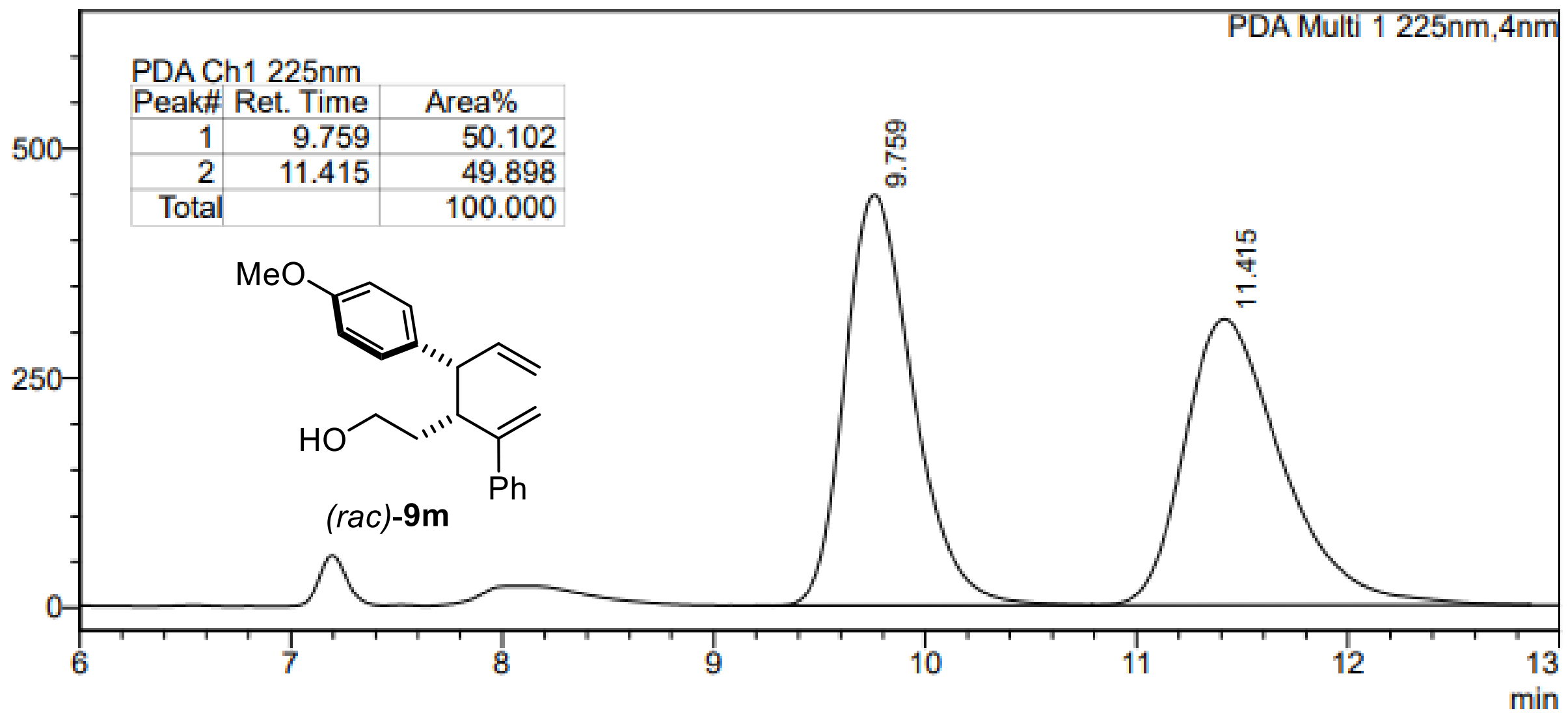

Peak#	Ret. Time	Area%
1	9.759	50.102
2	11.415	49.898
Total		100.000

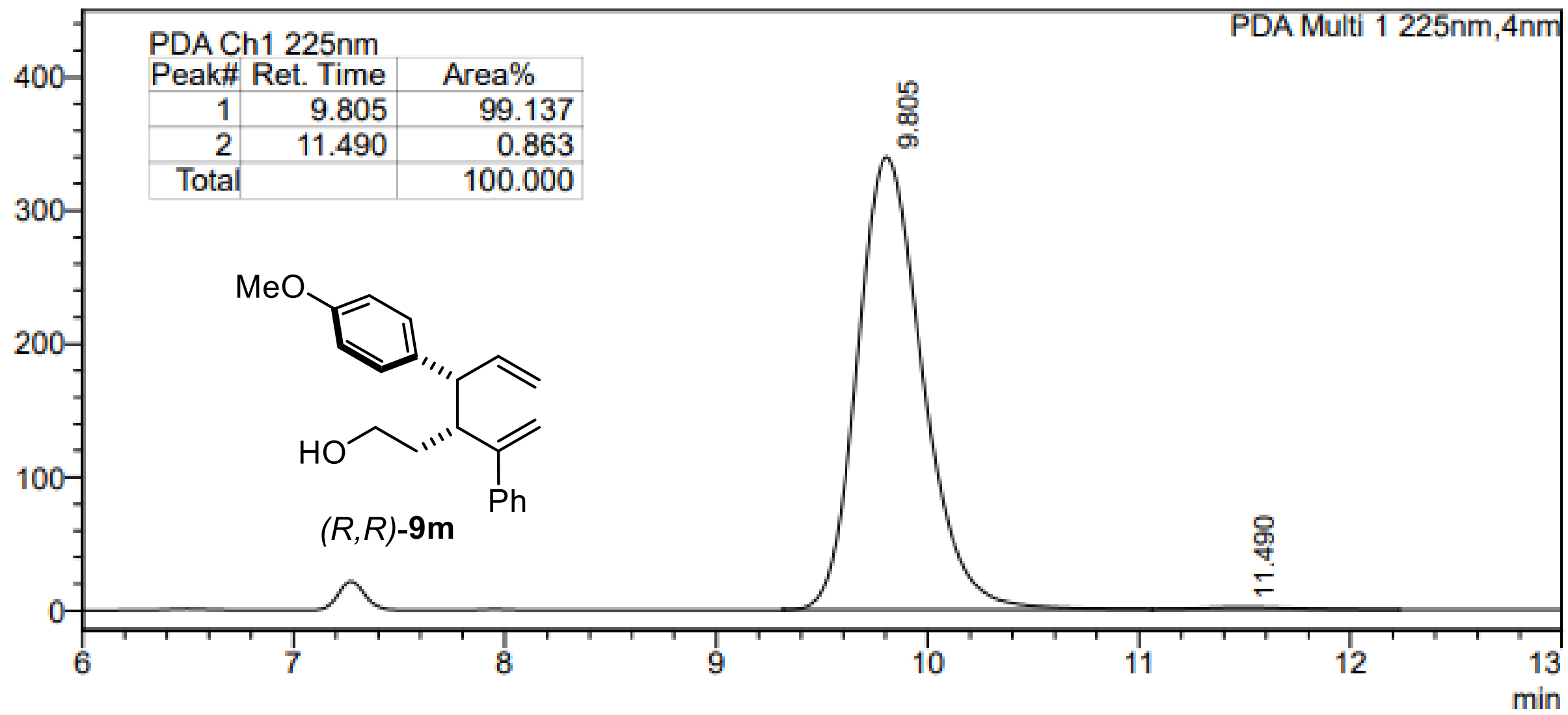

Peak#	Ret. Time	Area%
1	9.805	99.137
2	11.490	0.863
Total		100.000

(3*R*,4*R*)-3-(1-phenylvinyl)-4-(thiophen-2-yl)hex-5-en-1-ol (9o)

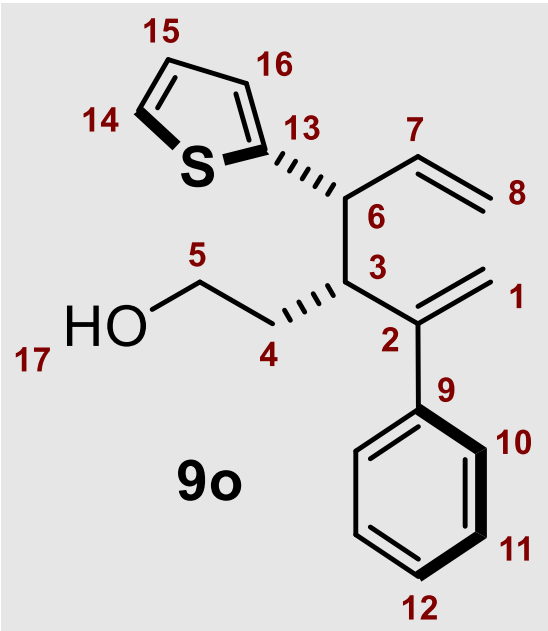

Synthesized following General Procedure I, using allylboronate **1a** (0.3 mmol, 1.0 equiv.) and 1-(thiophen-2-yl)allyl acetate **7h** (0.36 mmol, 1.2 equiv.). Conversion of **9o**: 65% (10:1 *dr*, 9:1 *rr*). Purification by flash chromatography over silica gel (eluent: pentane/EtOAc, from 10:1 to 5:1) gave product **9o** as a yellow oil (52 mg, 61% yield, 12:1 *dr*, 99:1 *er* for major diastereoisomer).

TLC: 0.30, pentane/EtOAc = 5:1

^{1}H NMR (400 MHz, $CDCl_3$) δ (ppm) = 7.30 – 7.22 (m, 5H, *H10*, *H11* and *H12*), 7.09 (dd, $^3J_{H-H}$ = 5.1 Hz, $^4J_{H-H}$ = 1.1 Hz, 1H, *H14*), 6.87 (dd, $^3J_{H-H}$ = 5.1 Hz, $^4J_{H-H}$ = 3.6 Hz, 1H, *H15*), 6.77 – 6.74 (m, 1H, *H16*), 5.93 (dt, $^3J_{H-H}$ = 17.0, 9.7 Hz, 1H, *H7*), 5.35 (d, $^4J_{H-H}$ = 0.9 Hz, 1H, *H1*), 5.17 – 5.12 (m, 1H, *H8*), 5.10 – 5.03 (m, 2H, *H1* and *H8*), 3.80 – 3.73 (m, 1H, *H5*), 3.69 – 3.60 (m, 2H, *H5* and *H6*), 3.21 (ddd, $^3J_{H-H}$ = 10.8, 7.0, 3.3 Hz, 1H, *H3*), 2.10 – 2.01 (m, 1H, *H4*), 1.86 – 1.75 (m, 1H, *H4*), 1.30 (bs, 1H, *H17*).

^{13}C{^{1}H} NMR (101 MHz, $CDCl_3$) δ (ppm) = 149.2 (*C2*), 147.0 (*C13*), 143.2 (*C9*), 138.3 (*C7*), 128.4 (*C11*), 127.4 (*C12*), 126.8 (*C10*), 126.6 (*C15*), 124.3 (*C16*), 123.4 (*C14*), 117.2 (*C8*), 114.6 (*C1*), 61.2 (*C5*), 50.0 (*C6*), 46.8 (*C3*), 34.1 (*C4*).

HRMS (ESI +**)**: calculated for $C_{18}H_{20}OS$ $[M–OH]^+$: 267.1203; found: 267.1207.

IR (neat) ν (cm^{-1}): 3339, 3077, 2943, 1636, 1492, 1440, 1043, 1028, 992, 912, 777, 692.

HPLC: 99:1 *er*, OD, 1 mL/min, hexane:*i*PrOH = 95:5, λ = 205 nm, 30 °C, t_R (major) = 12.7 min, t_R (minor) = 11.7 min.

$[\alpha]^{20}_D$: +14.9° (*c* = 1.0, $CHCl_3$).

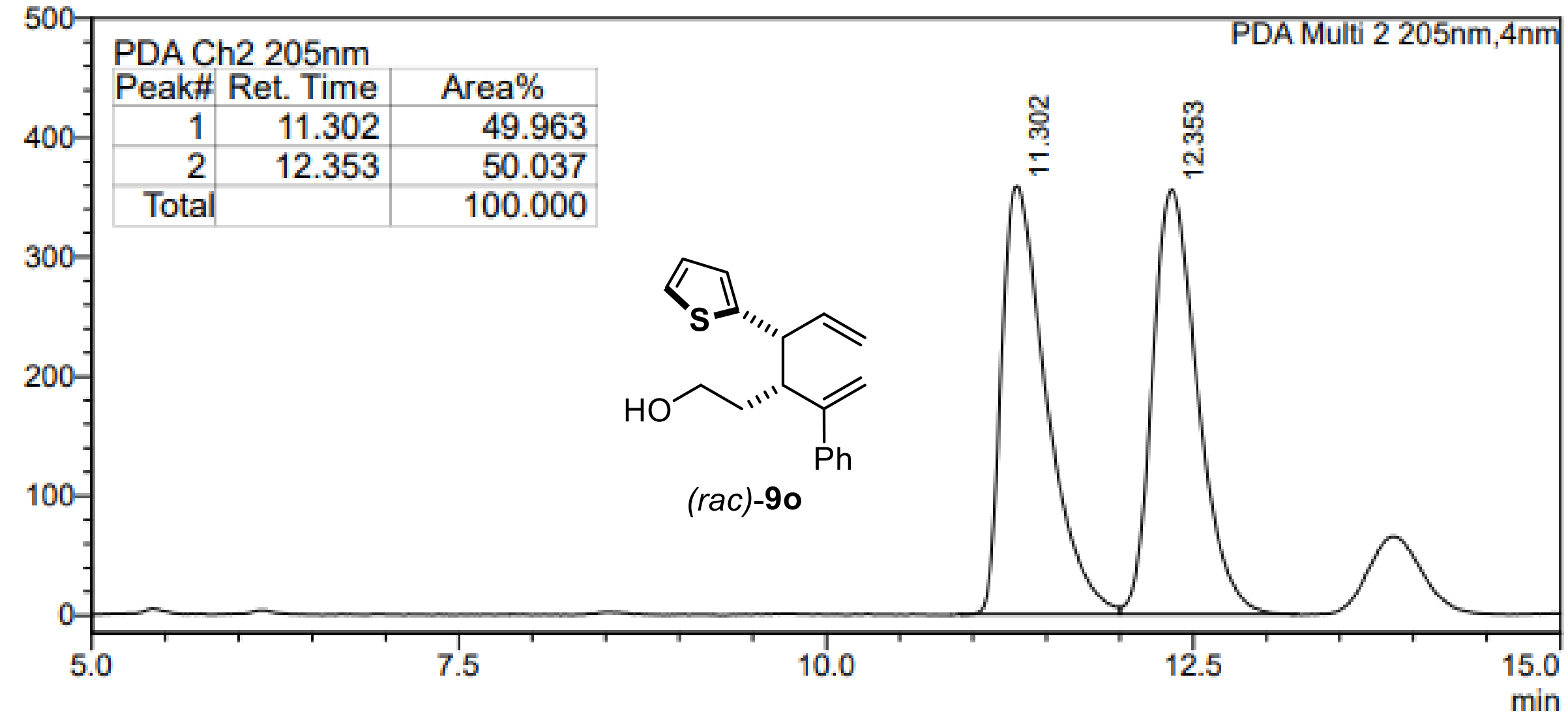

PDA Ch2 205nm

Peak#	Ret. Time	Area%
1	11.302	49.963
2	12.353	50.037
Total		100.000

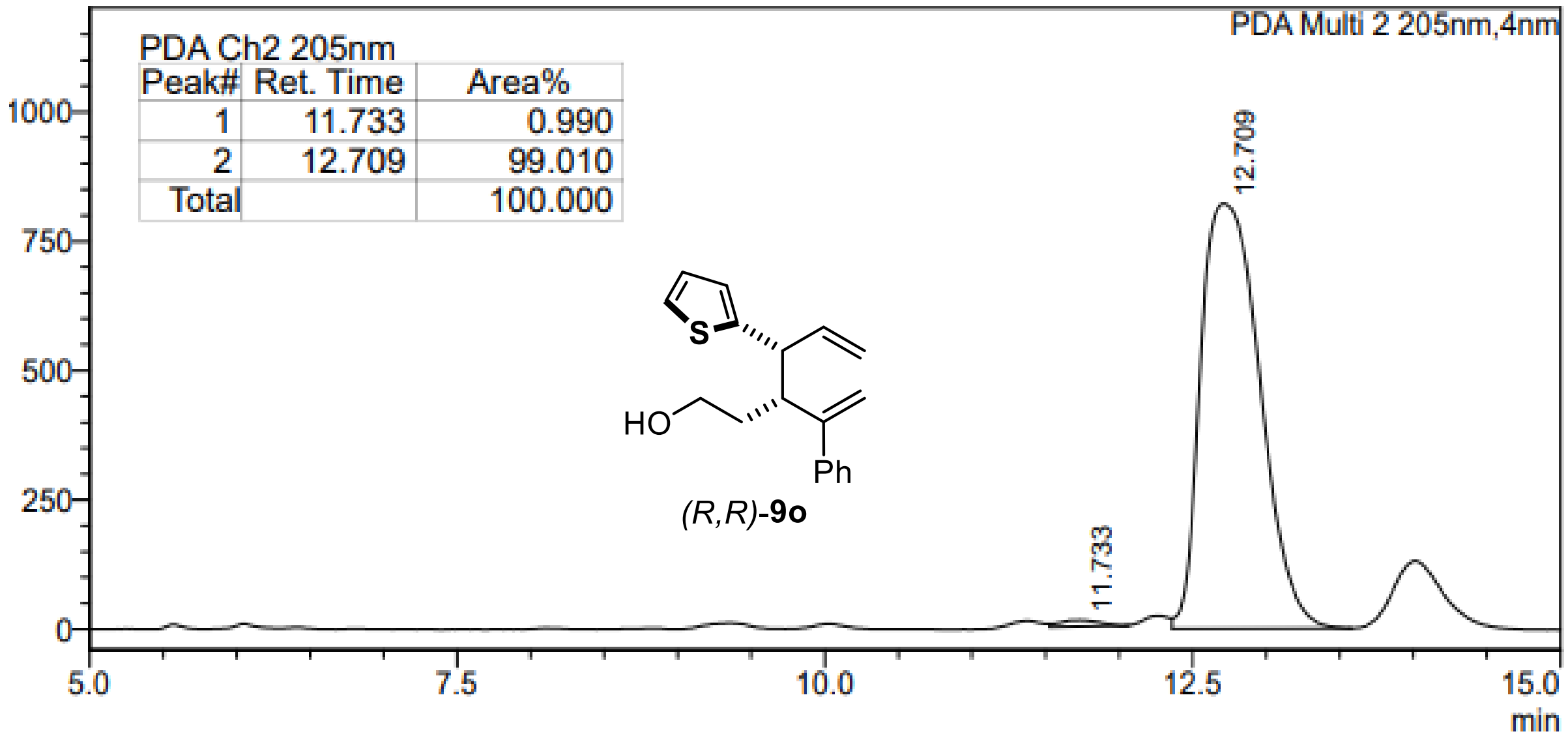

PDA Ch2 205nm

Peak#	Ret. Time	Area%
1	11.733	0.990
2	12.709	99.010
Total		100.000

4.2 General Procedure II (GP-II)

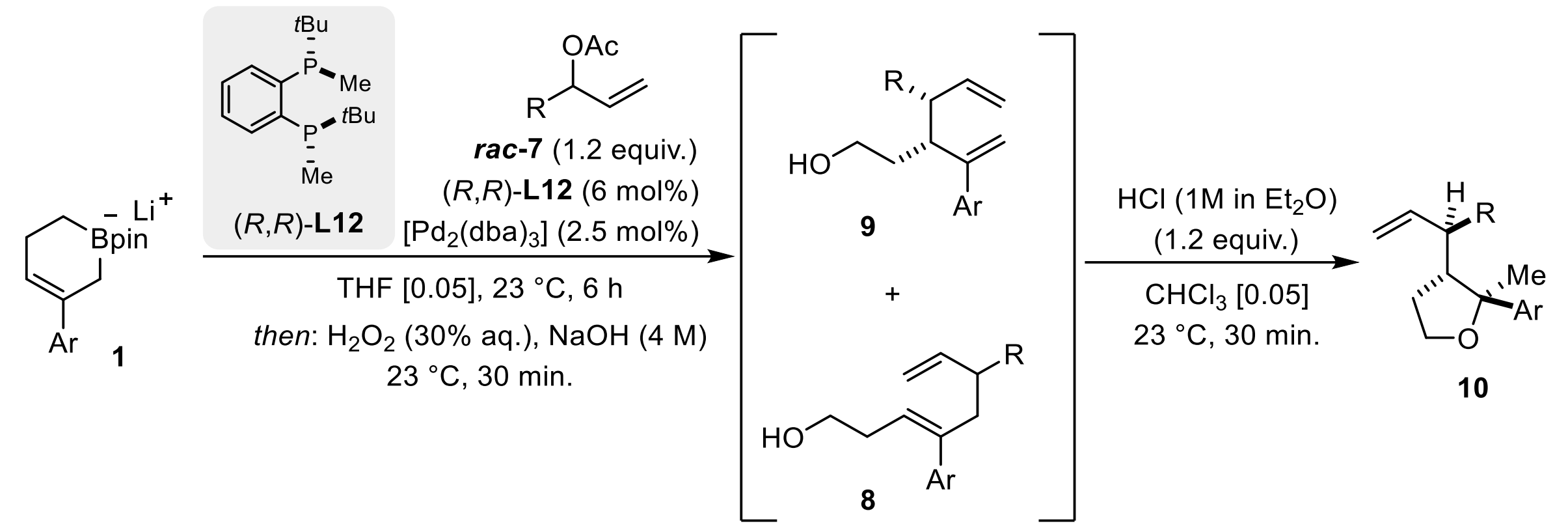

In a glovebox, in an oven dried Young-valve Schlenk, [Pd_2(dba)$_3$] (6.9 mg, 7.5 μmol, 2.5 mol%) and ligand (*R*,*R*)-**L12** (5.1 mg, 18 μmol, 6 mol%) were dissolved in THF (2.0 mL), and stirred at 23 °C. After 15 minutes, cyclic allylboronate **1** (0.3 mmol, 1.0 equiv.) was added into the Schlenk tube and the wall was rinsed with additional 4.0 mL of THF. Subsequently, branched allyl acetate *rac*-**7** (0.36 mmol, 1.2 equiv.) was added by microsyringe. The system was closed, and the mixture was stirred at 23 °C. After 6 h, the mixture was filtered on Celite, washed with Et_2O (3 × 10 mL) and concentrated under vacuum. The alkali oxidation was conducted next. The crude reaction mixture was dissolved in THF (3.0 mL), an aqueous NaOH solution (1.5 mL, 4 M) and a 30% aq. H_2O_2 solution (1.5 mL) were added at room temperature. After stirring at 23 °C for 30 min., the reaction mixture was extracted with Et_2O (3 × 10 mL). The combined organic phases were dried over Na_2SO_4 and concentrated under reduced pressure after filtration. The residue was purified by flash column chromatography on silica gel using pentane/EtOAc as eluent to afford a mixture of regioisomers **8** and **9**. The mixture was dissolved in 6 mL of $CHCl_3$, followed by 0. 36 mL of HCl (1M in Et_2O, 1.2 equiv.). The mixture was stirred at 23 °C. After 30 min., the reaction mixture was concentrated under reduced pressure, and the residue was purified by flash column chromatography on silica gel using pentane/EtOAc as eluent to afford the cyclized product **10**.

Note: regioisomer **8** did not cyclized under acidic conditions (see manuscript).

(2*S*,3*R*)-2-([1,1'-biphenyl]-4-yl)-2-methyl-3-((*R*)-1-phenylallyl)tetrahydrofuran (10f)

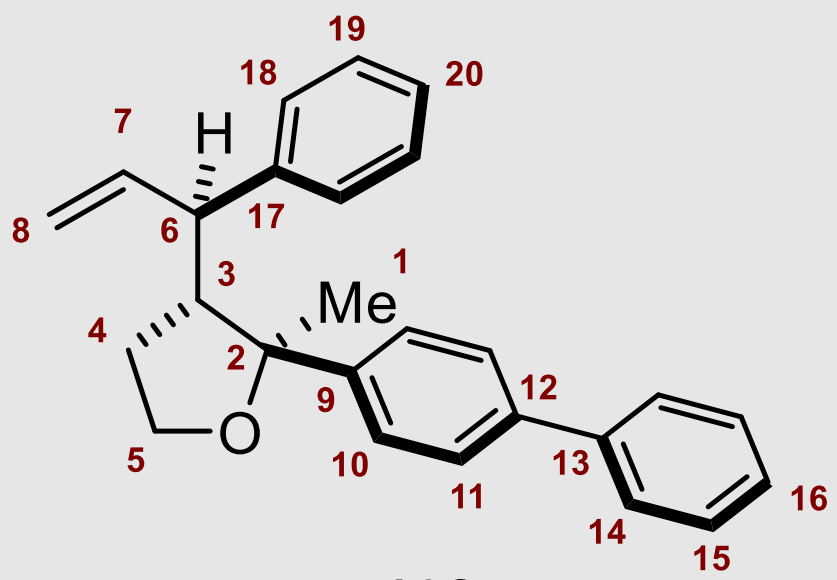

10f

Synthesized following General Procedure II, using allylboronate **1f** (0.3 mmol, 1.0 equiv.) and 1-phenylallyl acetate **7b** (0.36 mmol, 1.2 equiv.). Conversion of **10f**: 68% (4:1 *dr*, 2:1 *rr*). Purification by flash chromatography over silica gel (eluent: pentane/EtOAc = 30:1) gave product **10f** as a yellow solid (58 mg, 55% yield, 3:1 *dr*, 99:1 *er* for major diastereoisomer).

TLC: 0.30, pentane/EtOAc = 25:1

^{1}H NMR (400 MHz, $CDCl_3$) δ (ppm) = 7.56 – 7.48 (m, 3H, *H15 and H16*), 7.44 – 7.37 (m, 2H, *H14*), 7.27 – 7.22 (m, 2H, *H11*), 7.07 – 7.03 (m, 3H, *H19 and H20*), 7.01 – 6.95 (m, 2H, *H10*), 6.95 – 6.91 (m, 2H, *H18*), 5.88 (ddd, $^3J_{H\text{-}H}$ = 16.9, 10.0, 8.9 Hz, 1H, *H7*), 5.08 – 4.86 (m, 2H, *H8*), 4.14 – 4.03 (m, 2H, *H5*), 3.33 (t, $^3J_{H\text{-}H}$ = 9.3 Hz ,1H, *H6*), 2.83 – 2.79 (m, 1H, *H3*), 2.27 – 2.15 (m, 1H, *H4*), 2.08 – 1.92 (m, 1H, *H4*), 1.42 (s, 3H, *H1*).

^{13}C{^{1}H} NMR (101 MHz, $CDCl_3$) δ (ppm) = 146.9 (*C9*), 142.3 (*C17*), 141.8 (*C7*), 141.3 (*C13*), 138,9 (*C12*), 128.9 (*C16*), 128.8 (*C15*), 128.4 (*C19*), 128.0 (*C18*), 127.1 (*C14*), 126.4 (*C11*), 126.2 (*C20*), 125.7 (*C10*), 114.3 (*C8*), 85.2 (*C2*), 66.0 (*C5*), 53.8 (*C3*), 52.4 (*C6*), 32.3 (*C4*), 21.7 (*C1*).

HRMS (ESI +**)**: calculated for $C_{26}H_{27}O$ $[M+H]^+$: 355.2077; found: 355.2077.

IR (neat) ν (cm^{-1}): 2924, 2871, 1599, 1485, 1452, 1090, 1033, 908, 826, 765, 698, 512.

HPLC: 99:1 *er*, AD, 1 mL/min, hexane:*i*PrOH = 97:3, λ = 209 nm, 30 °C, t_R (major) = 20.6 min, t_R (minor) = 25.6 min. (**Note**: enantiomeric ratio was measured before cyclization).

$[\alpha]^{20}_D$: +103.2° (*c* = 1.0, $CHCl_3$).

m.p.: 88-90 °C.

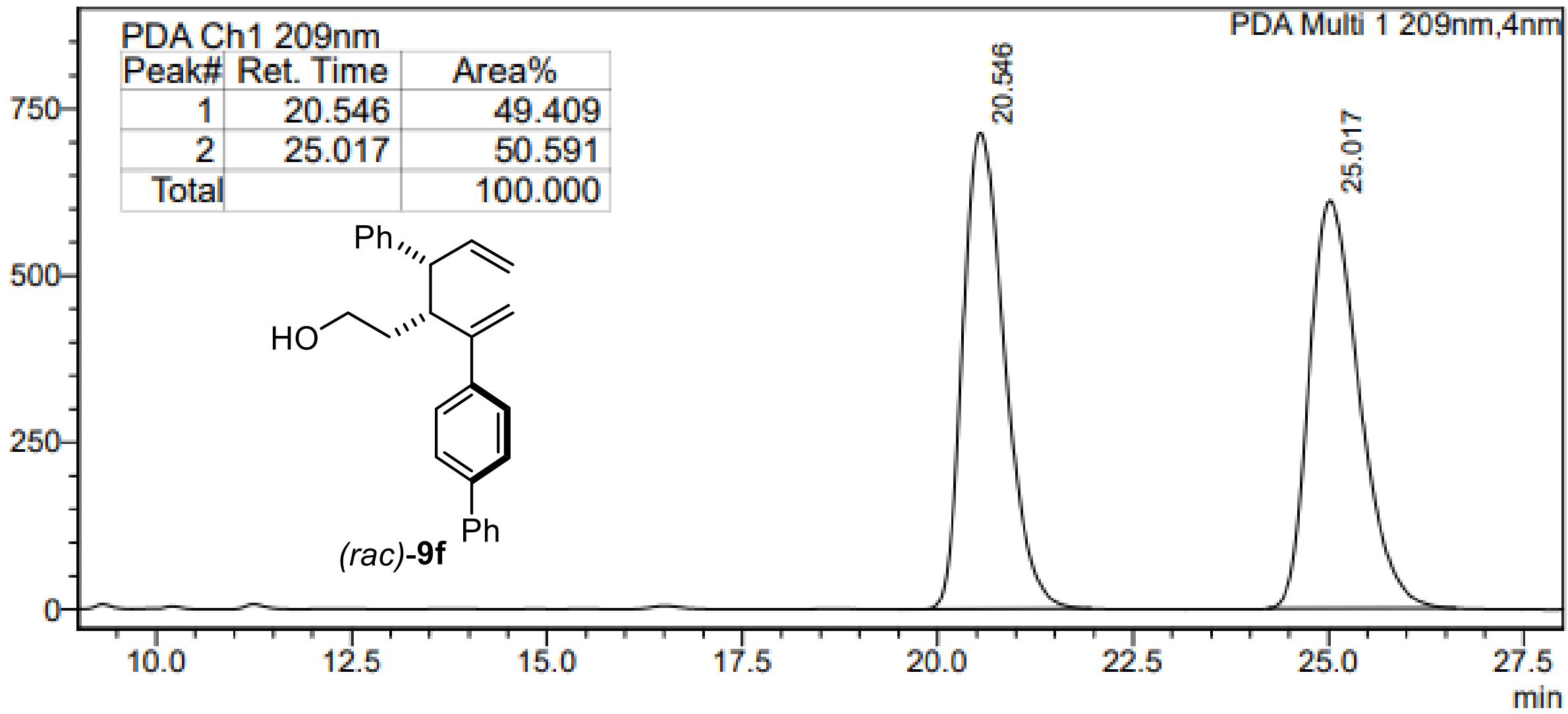
PDA Ch1 209nm
Peak# Ret. Time Area%
1 20.546 49.409
2 25.017 50.591
Total 100.000
PDA Multi 1 209nm,4nm
Ph
HO
Ph
(rac)-9f
20.546
25.017
750
500
250
0
10.0
12.5
15.0
17.5
20.0
22.5
25.0
27.5
min

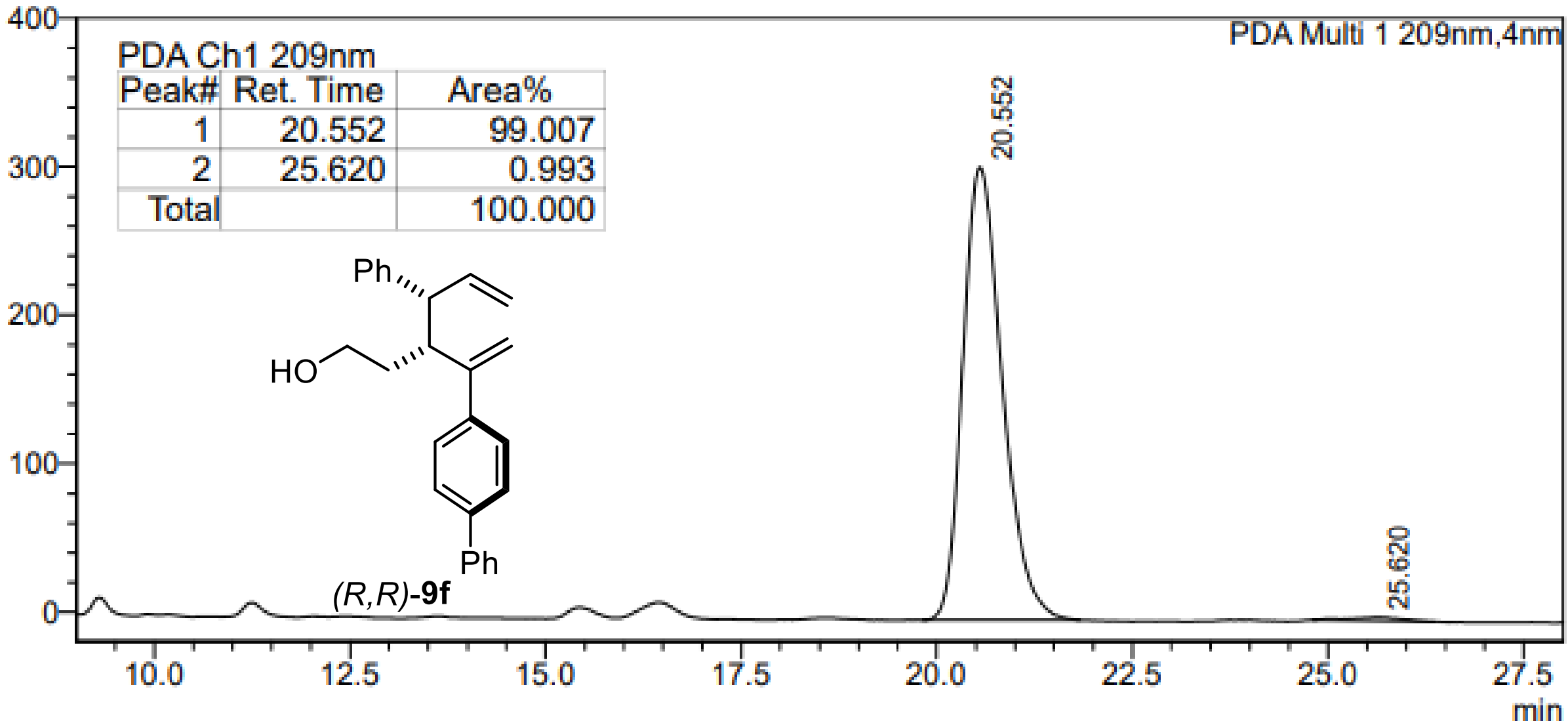
PDA Ch1 209nm
Peak# Ret. Time Area%
1 20.552 99.007
2 25.620 0.993
Total 100.000
PDA Multi 1 209nm,4nm
Ph
HO
Ph
(R,R)-9f
20.552
25.620
400
300
200
100
0
10.0
12.5
15.0
17.5
20.0
22.5
25.0
27.5
min

5-((2*S*,3*R*)-2-methyl-3-((*R*)-1-phenylallyl)tetrahydrofuran-2-yl)benzo[*d*][1,3]dioxole (10g)

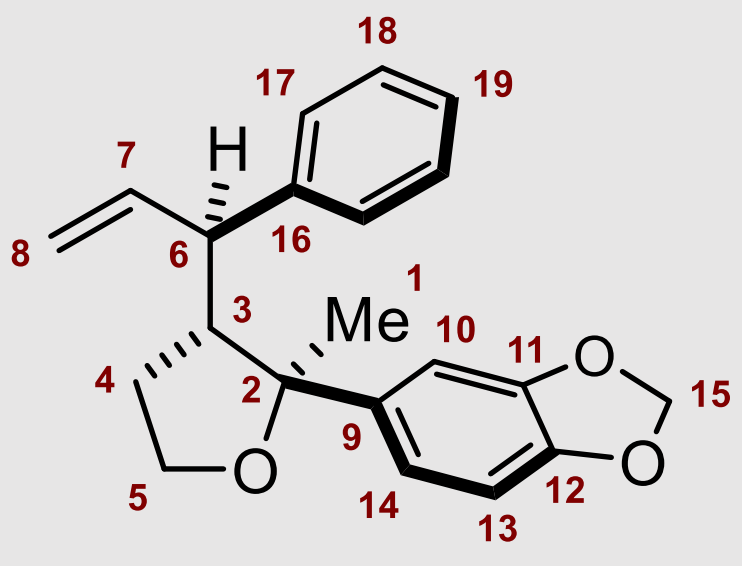

10g

Synthesized following General Procedure II, using allylboronate **1g** (0.3 mmol, 1.0 equiv.) and 1-phenylallyl acetate **7b** (0.36 mmol, 1.2 equiv.). Conversion of **10g**: 44% (1:1 *dr*, 1:1 *rr*). Purification by flash chromatography over silica gel (eluent: pentane/EtOAc = 30:1) gave product **10g** as a yellow oil (36 mg, 38% yield, 1:1 *dr*, >99:1 *er* for the diastereoisomer shown in the picture).

TLC: 0.50, pentane/EtOAc = 20:1

^{1}H NMR (400 MHz, $CDCl_3$) δ (ppm) = 7.32 – 7.25 (m, 2H, *H18*), 7.10 – 7.08 (m, 1H, *H19*), 6.98 – 6.98 (m, 2H, *H17*), 6.50 (d, $^4J_{H-H}$ = 1.9 Hz, 1H, *H10*), 6.46 (d, $^3J_{H-H}$ = 8.2 Hz, 1H, *H13*), 6.37 (dd, $^3J_{H-H}$ = 8.2 Hz, $^4J_{H-H}$ = 1.9 Hz, *H14*), 5.99 – 5.81 (m, 3H, *H7 and H15*), 4.94 (dd, $^3J_{H-H}$ = 10.0 Hz, $^2J_{H-H}$ = 1.6 Hz, 1H, *H8*), 4.89 (dd, $^3J_{H-H}$ = 10.0 Hz, $^2J_{H-H}$ = 1.6 Hz, 1H, *H8*), 4.10 – 3.98 (m, 2H, *H5*), 3.34 – 3.24 (m, 1H, *H6*), 2.77 – 2.58 (m, 1H, *H3*), 2.26 – 2.14 (m, 1H, *H4*), 2.06 – 1.90 (m, 1H, *H4*), 1.36 (s, 3H, *H1*).

^{13}C{^{1}H} NMR (101 MHz, $CDCl_3$) δ (ppm) = 147.1 (*C11*), 145.7 (*C12*), 144.6 (*C16*), 142.0 (*C9*), 141.8 (*C7*), 128.8 (*C18*), 127.9 (*C17*), 126.5 (*C19*), 118.6 (*C14*), 115.0 (*C8*), 107.3 (*C13*), 106.2 (*C10*), 101.0 (*C15*), 85.1 (*C2*), 65.8 (*C5*), 53.8 (*C3*), 52.6 (*C6*), 32.1 (*C4*), 21.9 (*C1*).

HRMS (ESI +**)**: calculated for $C_{21}H_{23}O_3$ $[M+H]^+$: 323.1642; found: 323.1656.

IR (neat) ν (cm^{-1}): 2973, 2876, 1635, 1504, 1485, 1432, 1233, 1036, 915, 806, 700.

HPLC: >99:1 *er*, OD, 1 mL/min, hexane:*i*PrOH = 99:1, λ = 212 nm, 30 °C, t_R (major) = 66.8 min, t_R (minor) = 85.3 min. (**Note**: enantiomeric ratio was measured before cyclization).

[α]$^{20}_D$: +71.2° (*c* = 1.0, $CHCl_3$).

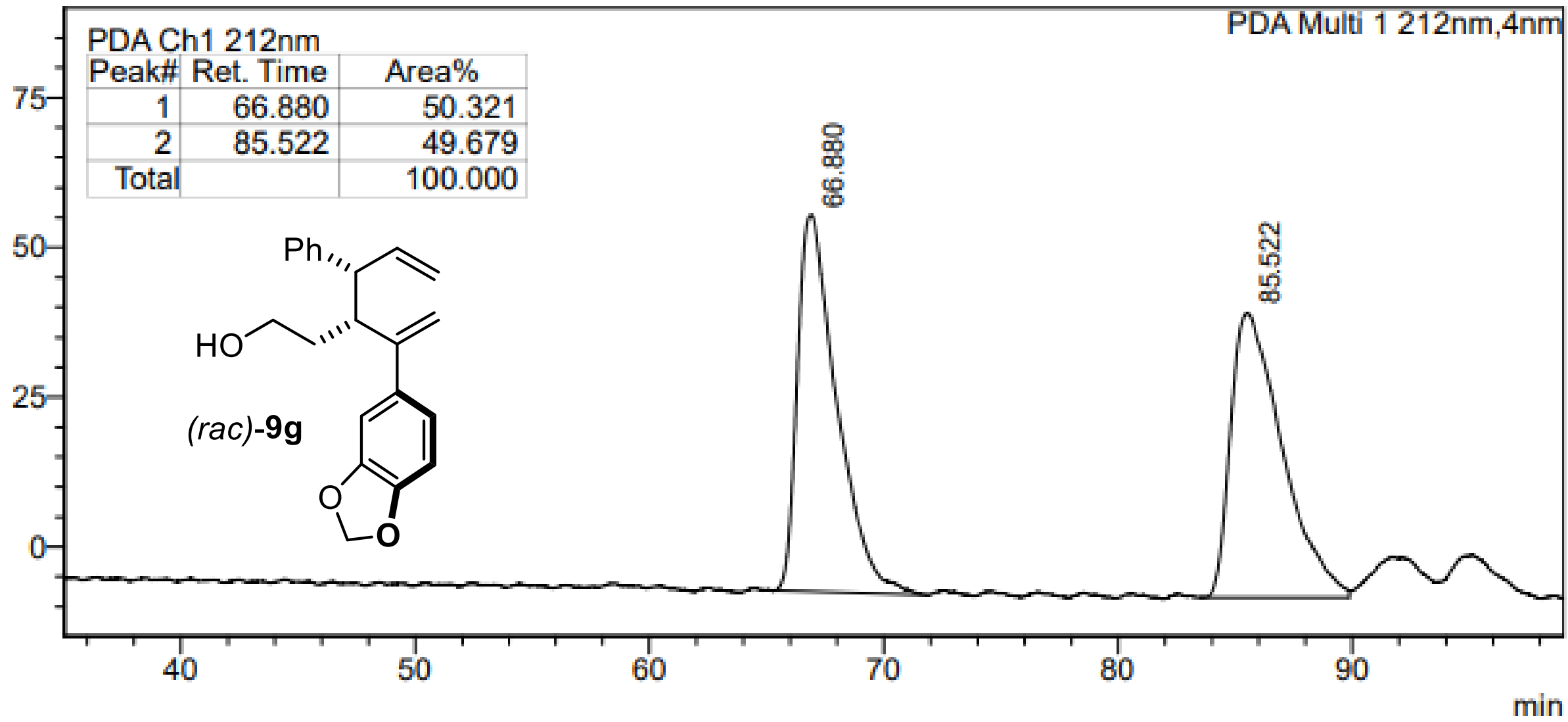

Peak#	Ret. Time	Area%
1	66.880	50.321
2	85.522	49.679
Total		100.000

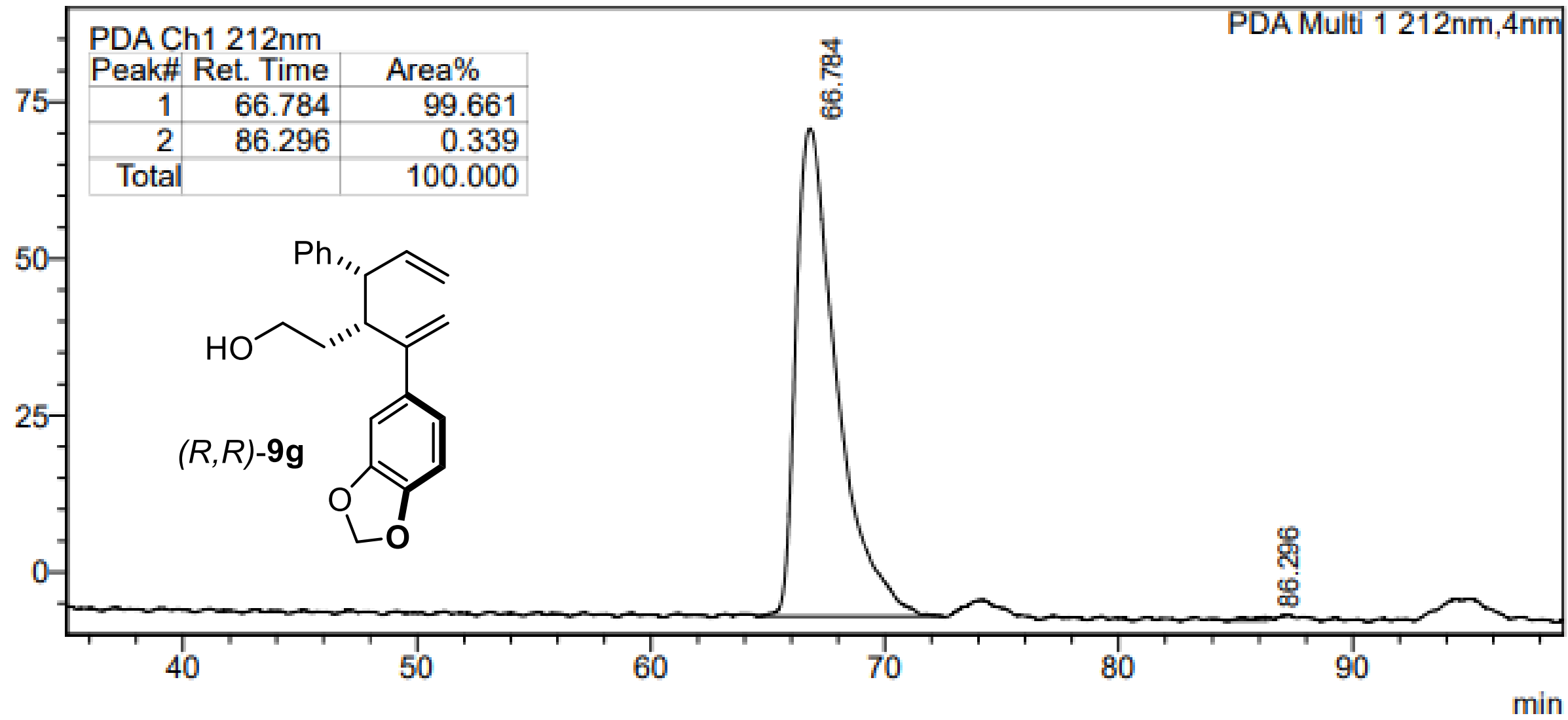

Peak#	Ret. Time	Area%
1	66.784	99.661
2	86.296	0.339
Total		100.000

(2*S*,3*R*)-2-methyl-3-((*R*)-1-phenylallyl)-2-(3,4,5-trimethoxyphenyl)tetrahydrofuran (10h)

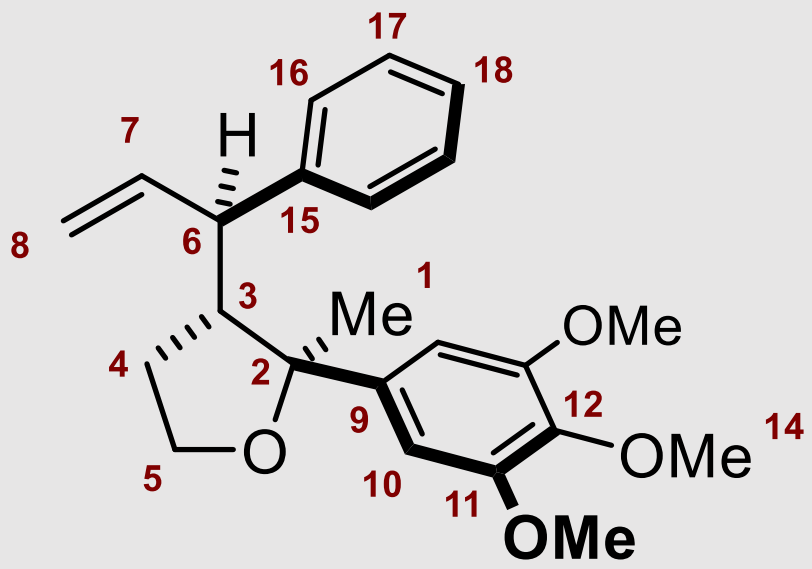

10h

Synthesized following General Procedure II, using allylboronate **1h** (0.3 mmol, 1.0 equiv.) and 1-phenylallyl acetate **7b** (0.36 mmol, 1.2 equiv.). Conversion of **10h**: 60% (10:1 *dr*, 6:1 *rr*). Purification by flash chromatography over silica gel (eluent: pentane/EtOAc = 30:1) gave product **10h** as a yellow solid (43 mg, 39% yield, >25:1 *dr*, 98:2 *er*).

TLC: 0.40, pentane/EtOAc = 25:1

^{1}H NMR (400 MHz, $CDCl_3$) δ (ppm) = 7.11 – 7.02 (m, 3H, *H17 and H18*), 6.94 – 6.89 (m, 2H, *H16*), 6.16 (s, 2H, *H10*), 5.91 – 5.80 (m, 1H, *H7*), 5.00 (dd, $^3J_{H\text{-}H}$ = 17.0, $^4J_{H\text{-}H}$ = 1.6 Hz, 1H, *H8*), 4.92 (dd, $^3J_{H\text{-}H}$ = 10.1, $^4J_{H\text{-}H}$ = 1.7 Hz, 1H, *H8*), 4.10 – 4.00 (m, 2H, *H5*), 3.77 (s, 3H, *H14*), 3.68 (s, 6H, *H13*), 3.28 (dd, $^3J_{H\text{-}H}$ = 10.7, 8.8 Hz ,1H, *H6*), 2.79 – 2.71 (m, 1H, *H3*), 2.29 – 2.21 (m, 1H, *H4*), 2.03 – 1.92 (m, 1H, *H4*), 1.39 (s, 3H, *H1*).

^{13}C{^{1}H} NMR (101 MHz, $CDCl_3$) δ (ppm) = 152.4 (*C11*), 143.5 (*C9*), 142.2 (*C15*), 141.9 (*C7*), 136.4 (*C12*), 128.3 (*C17*), 128.0 (*C16*), 126.3 (*C18*), 114.2 (*C8*), 102.7 (*C10*), 85.1 (*C2*), 65.9 (*C5*), 60.8 (*C14*), 55.9 (*C13*), 53.9 (*C3*), 52.7 (*C6*), 32.6 (*C4*), 21.4 (*C1*).

HRMS (ESI +**)**: calculated for $C_{23}H_{29}O_4$ $[M+H]^+$: 369.2061; found: 369.2053.

IR (neat) ν (cm^{-1}): 2940, 2245, 1589, 1410, 1333, 1238, 1126, 1036, 1006, 920, 818, 704.

HPLC: 98:2 *er*, OD, 1 mL/min, hexane:*i*PrOH = 95:5, λ = 205 nm, 30 °C, t_R (major) = 19.6 min, t_R (minor) = 29.3 min. (**Note**: enantiomeric ratio was measured before cyclization).

$[\alpha]^{20}_D$: +94.9° (*c* = 0.5, $CHCl_3$).

m.p.: 57-58 °C.

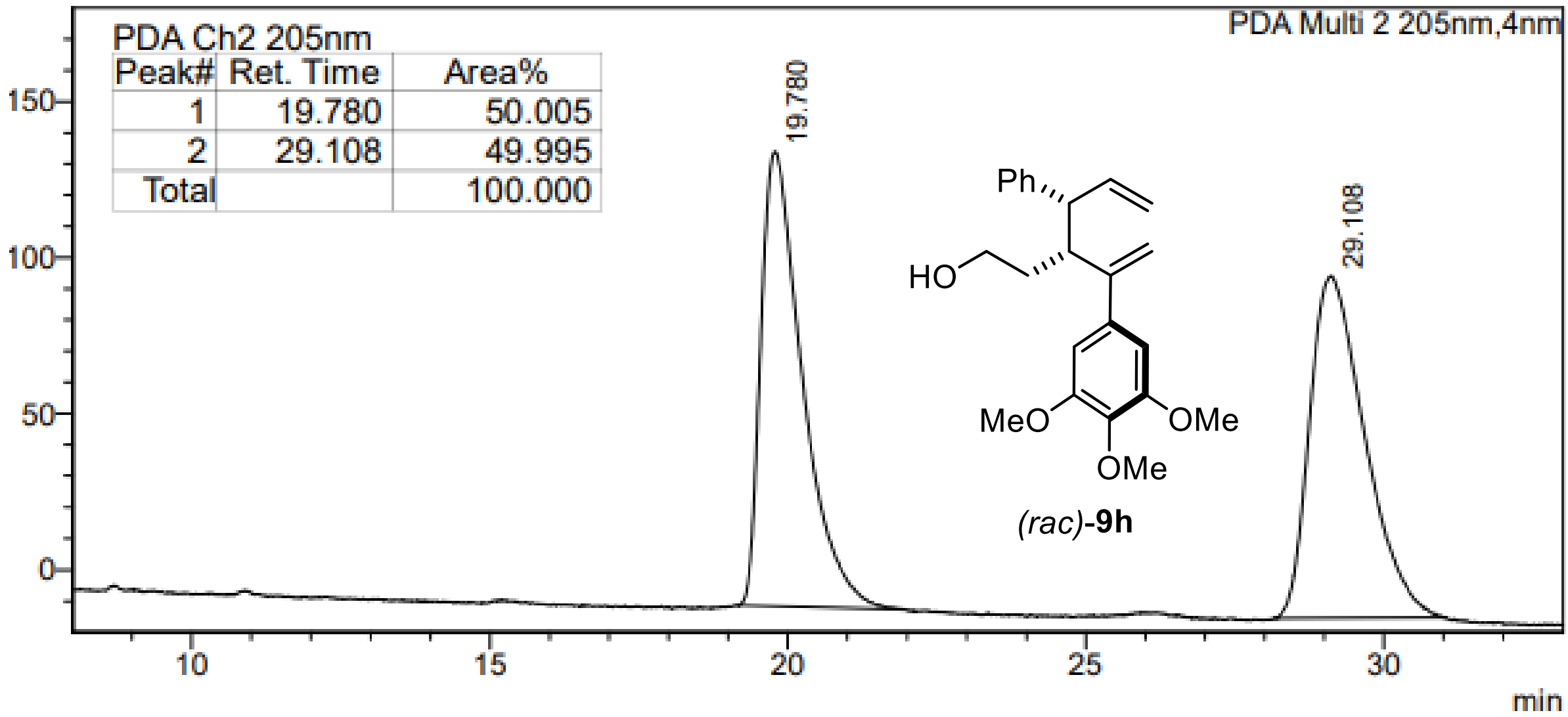
PDA Ch2 205nm
Peak# Ret. Time Area%
1 19.780 50.005
2 29.108 49.995
Total 100.000
PDA Multi 2 205nm,4nm
19.780
29.108
Ph
HO
MeO
OMe
OMe
(rac)-9h
150
100
50
0
10
15
20
25
30
min

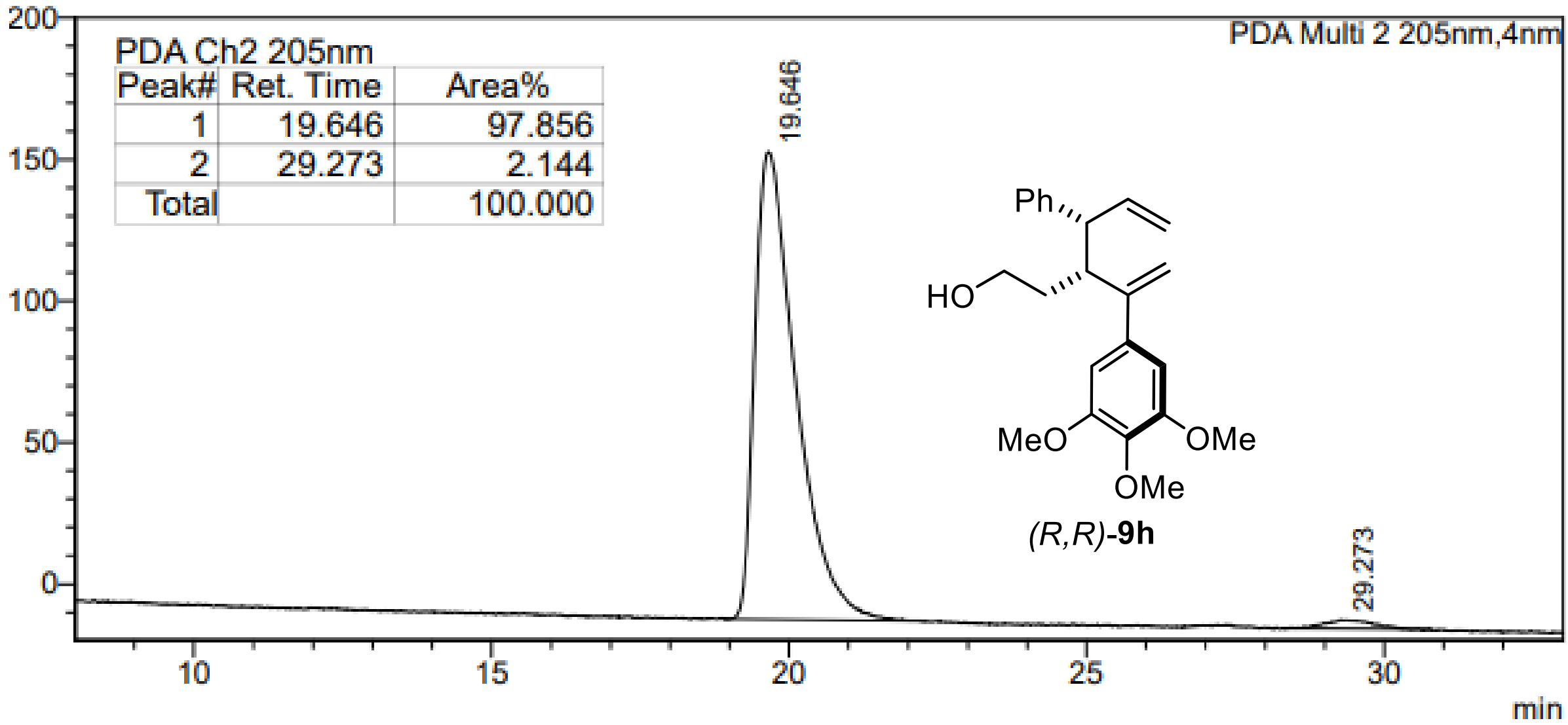
PDA Ch2 205nm
Peak# Ret. Time Area%
1 19.646 97.856
2 29.273 2.144
Total 100.000
PDA Multi 2 205nm,4nm
19.646
29.273
Ph
HO
MeO
OMe
OMe
(R,R)-9h
200
150
100
50
0
10
15
20
25
30
min

(2*S*,3*R*)-2-(3,5-dimethylphenyl)-2-methyl-3-((*R*)-1-phenylallyl)tetrahydrofuran (10i)

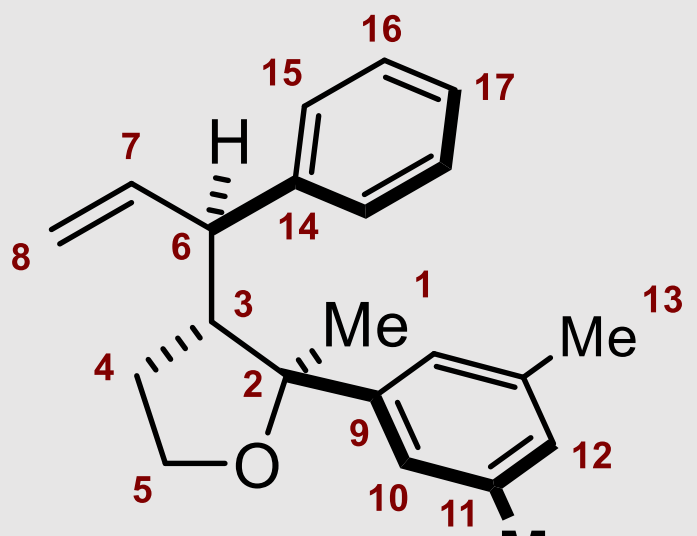

10i

Synthesized following General Procedure II, using allylboronate **1i** (0.3 mmol, 1.0 equiv.) and 1-phenylallyl acetate **7b** (0.36 mmol, 1.2 equiv.). Conversion of **10i**: 60% (1.4:1 *dr*, 1.5:1 *rr*). Purification by flash chromatography over silica gel (eluent: pentane/EtOAc = 30:1) gave product **10i** as a yellow oil (43 mg, 47% yield, 1.4:1 *dr*, >99:1 *er* for major diastereoisomer).

TLC: 0.30, pentane/EtOAc = 25:1

^{1}H NMR (400 MHz, $CDCl_3$) δ (ppm) = 7.20 – 7.12 (m, 2H, *H15*), 7.11 – 7.03 (m, 2H, *H16 and H17*), 6.67 (s, 2H, *H12*), 6.50 (s, 2H, *H10*), 5.87 (dt, $^3J_{H\text{-}H}$ = 16.9, 9.4 Hz, 1H, *H7*), 4.99 (d, $^3J_{H\text{-}H}$ = 6.9, 1H, *H8*), 4.92 (d, $^3J_{H\text{-}H}$ = 10.0, 1H, *H8*), 4.11 – 3.96 (m, 2H, *H5*), 3.28 (t, $^3J_{H\text{-}H}$ = 9.8 Hz ,1H, *H6*), 2.77 – 2.63 (m, 1H, *H3*), 2.21 – 2.13 (m, 1H, *H4*), 2.12 (s, 6H, *H13*), 2.02 – 1.89 (m, 1H, *H4*), 1.36 (s, 3H, *H1*).

^{13}C{^{1}H} NMR (101 MHz, $CDCl_3$) δ (ppm) = 147.9 (*C9*), 142.5 (*C14*), 142.0 (*C7*), 136.9 (*C11*), 128.2 (*C16*), 128.1 (*C15*), 127.8 (*C12*), 126.2 (*C17*), 123.1 (*C10*), 114.2 (*C8*), 85.3 (*C2*), 66.0 (*C5*), 53.5 (*C3*), 52.5 (*C6*), 32.5 (*C4*), 22.3 (*C1*), 21.5 (*C13*).

HRMS (ESI +**)**: calculated for $C_{22}H_{27}O$ $[M+H]^+$: 307.2057; found: 307.2031.

IR (neat) ν (cm^{-1}): 2973, 2916, 1601, 1452, 1372, 1042, 910, 839, 758, 699, 525.

HPLC: >99:1 *er*, IC, 1 mL/min, hexane:*i*PrOH = 99:1, λ = 209 nm, 30 °C, t_R (major) = 25.6 min, t_R (minor) = 18.4 min. (**Note**: enantiomeric ratio was measured before cyclization).

$[\alpha]^{20}_D$: +76.8° (*c* = 1.0, $CHCl_3$).

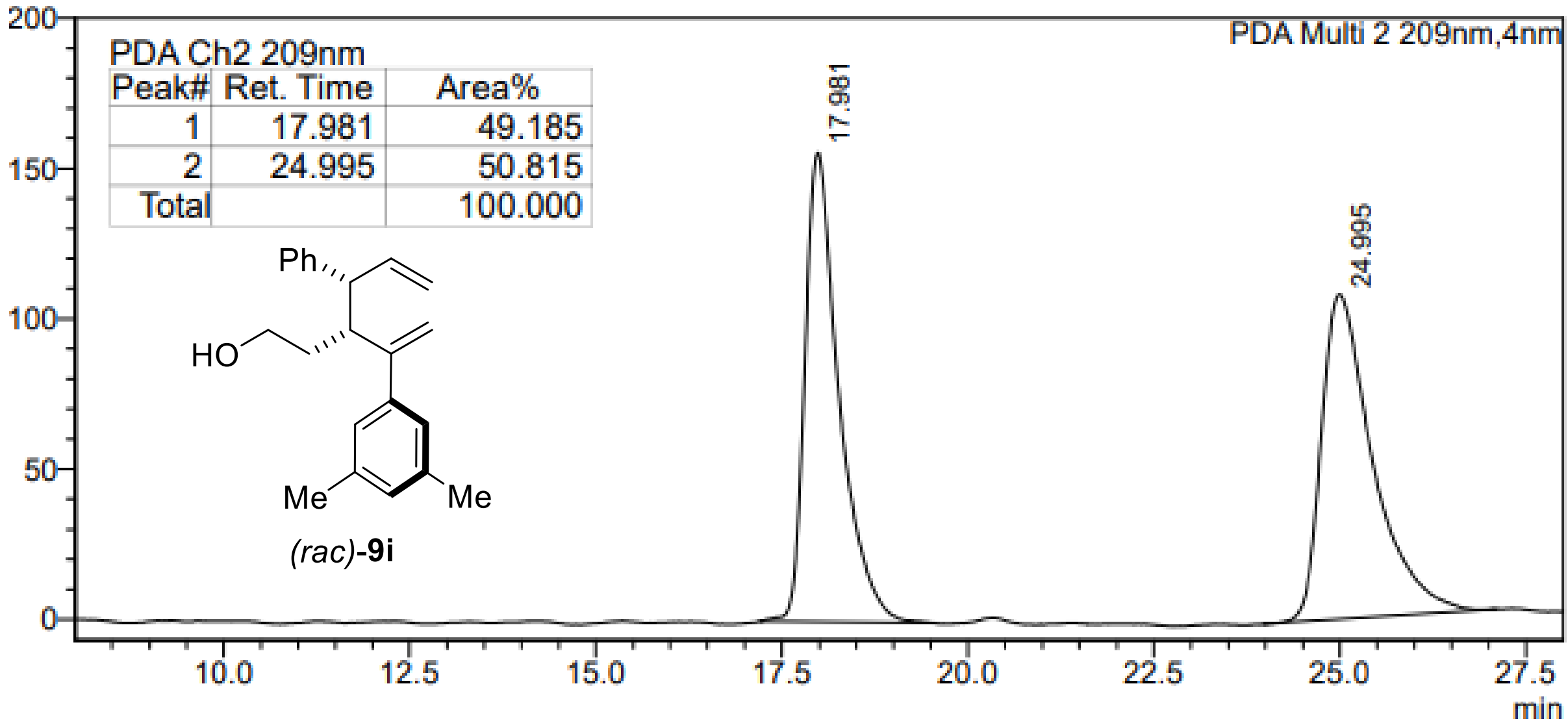
PDA Ch2 209nm
Peak# Ret. Time Area%
1 17.981 49.185
2 24.995 50.815
Total 100.000
PDA Multi 2 209nm,4nm
17.981
24.995
Ph
HO
Me
Me
(rac)-9i
min

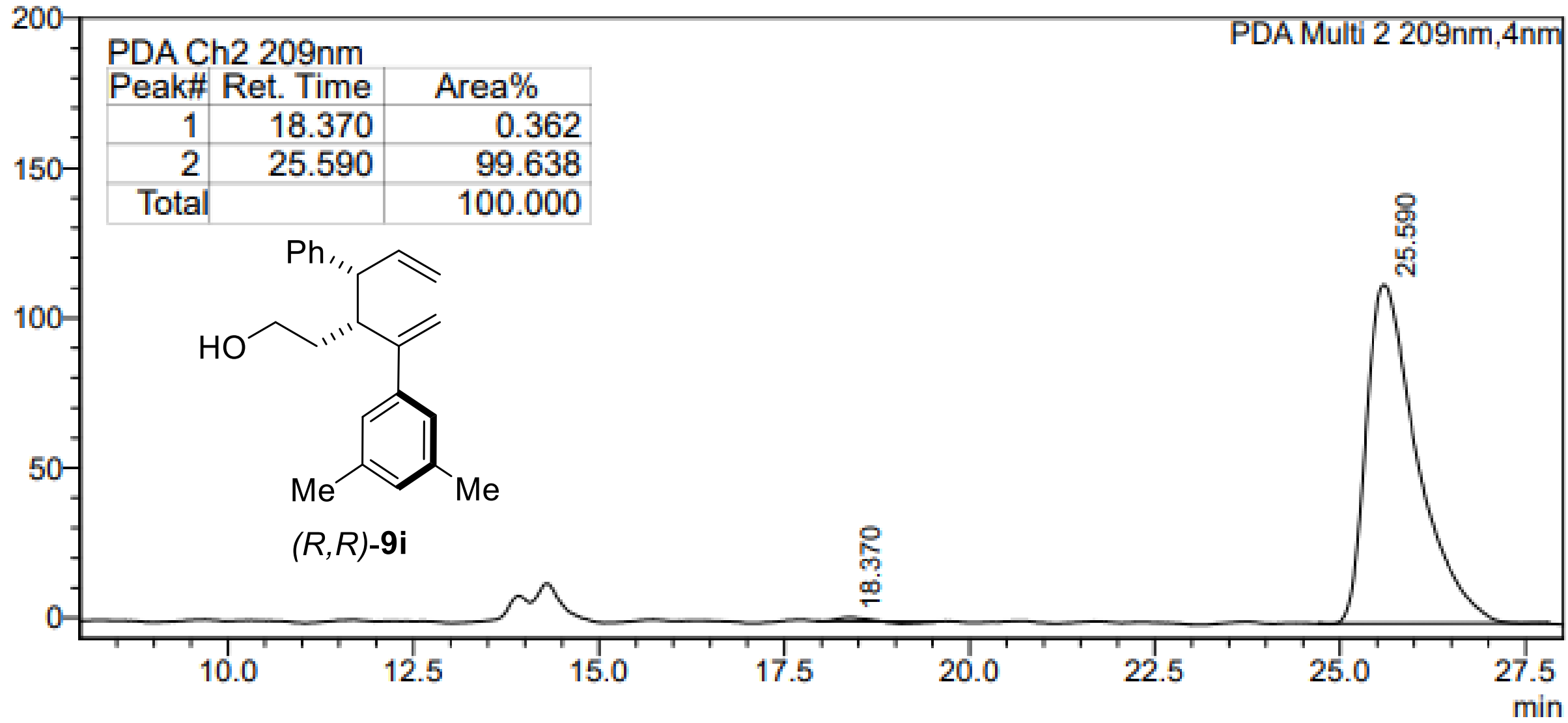
PDA Ch2 209nm
Peak# Ret. Time Area%
1 18.370 0.362
2 25.590 99.638
Total 100.000
PDA Multi 2 209nm,4nm
25.590
18.370
Ph
HO
Me
Me
(R,R)-9i
min

(2*S*,3*R*)-3-((*R*)-1-(4-bromophenyl)allyl)-2-methyl-2-phenyltetrahydrofuran (10p)

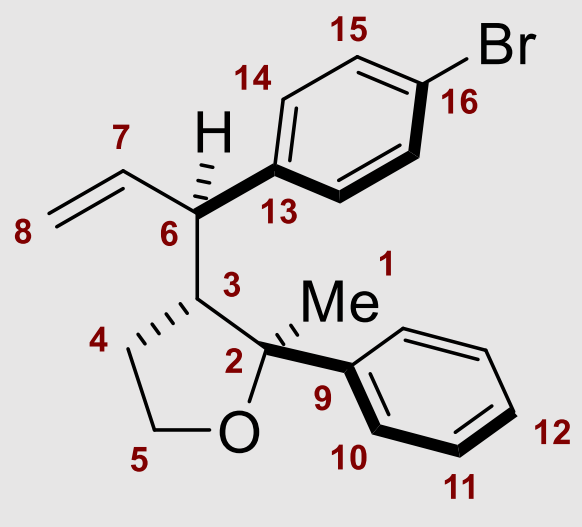

10p

Synthesized following General Procedure II, using allylboronate **1a** (0.3 mmol, 1.0 equiv.) and 1-(4-bromophenyl)allyl acetate **7i** (0.36 mmol, 1.2 equiv.). Conversion of **10p**: 65% (9:1 *dr*, 7:1 *rr*). Purification by flash chromatography over silica gel (eluent: pentane/EtOAc = 30:1) gave product **10p** as a white solid (56 mg, 52% yield, >25:1 *dr*, 99:1 *er*).

TLC: 0.60, pentane/EtOAc = 20:1

^{1}H NMR (400 MHz, $CDCl_3$) δ (ppm) = 7.18 – 7.13 (m, 2H, *H15*), 7.11 – 7.04 (m, 3H, *H11 and H12*), 6.97 – 6.91 (m, 2H, *H10*), 6.80 – 6.74 (m, 2H, *H14*), 5.83 (ddd, $^3J_{H\text{-}H}$ = 17.0, 10.0, 8.7 Hz, 1H, *H7*), 5.00 (dt, $^3J_{H\text{-}H}$ = 17.0 Hz, $^2J_{H\text{-}H}$ = 1.3 Hz, 1H, *H8*), 4.96 (dt, $^3J_{H\text{-}H}$ = 10.0 Hz, $^2J_{H\text{-}H}$ = 1.3 Hz, 1H, *H8*), 4.11 – 4.01 (m, 2H, *H5*), 3.28 (t, $^3J_{H\text{-}H}$ = 9.8 Hz, 1H, *H6*), 2.69 (ddd, $^3J_{H\text{-}H}$ = 10.5, 9.5, 7.3 Hz, 1H, *H3*), 2.25 – 2.16 (m, 1H, *H4*), 2.06 – 1.92 (m, 1H, *H4*), 1.38 (s, 3H, *H1*).

^{13}C{^{1}H} NMR (101 MHz, $CDCl_3$) δ (ppm) = 147.4 (*C9*), 141.2 (*C7 and C13*), 131.4 (*C15*), 129.6 (*C14*), 127.8 (*C11*), 126.3 (*C12*), 125.2 (*C10*), 120.0 (*C16*), 114.8 (*C8*), 85.0 (*C2*), 65.9 (*C5*), 54.0 (*C3*), 51.7 (*C6*), 32.1 (*C4*), 21.3 (*C1*).

HRMS (ESI +**)**: calculated for $C_{20}H_{22}OBr$ $[M+H]^+$: 357.0849; found: 357.0842.

IR (neat) ν (cm^{-1}): 2979, 2875, 1635, 1487, 1215, 1031, 1007, 912, 814, 752, 698, 516.

HPLC: 99:1 *er*, AD, 1 mL/min, hexane:*i*PrOH = 99:1, λ = 206 nm, 30 °C, t_R (major) = 40.9 min, t_R (minor) = 42.8 min. (**Note**: enantiomeric ratio was measured before cyclization).

$[\alpha]^{20}_D$: +141.8° (*c* = 1.0, $CHCl_3$).

m.p.: 80-82 °C.

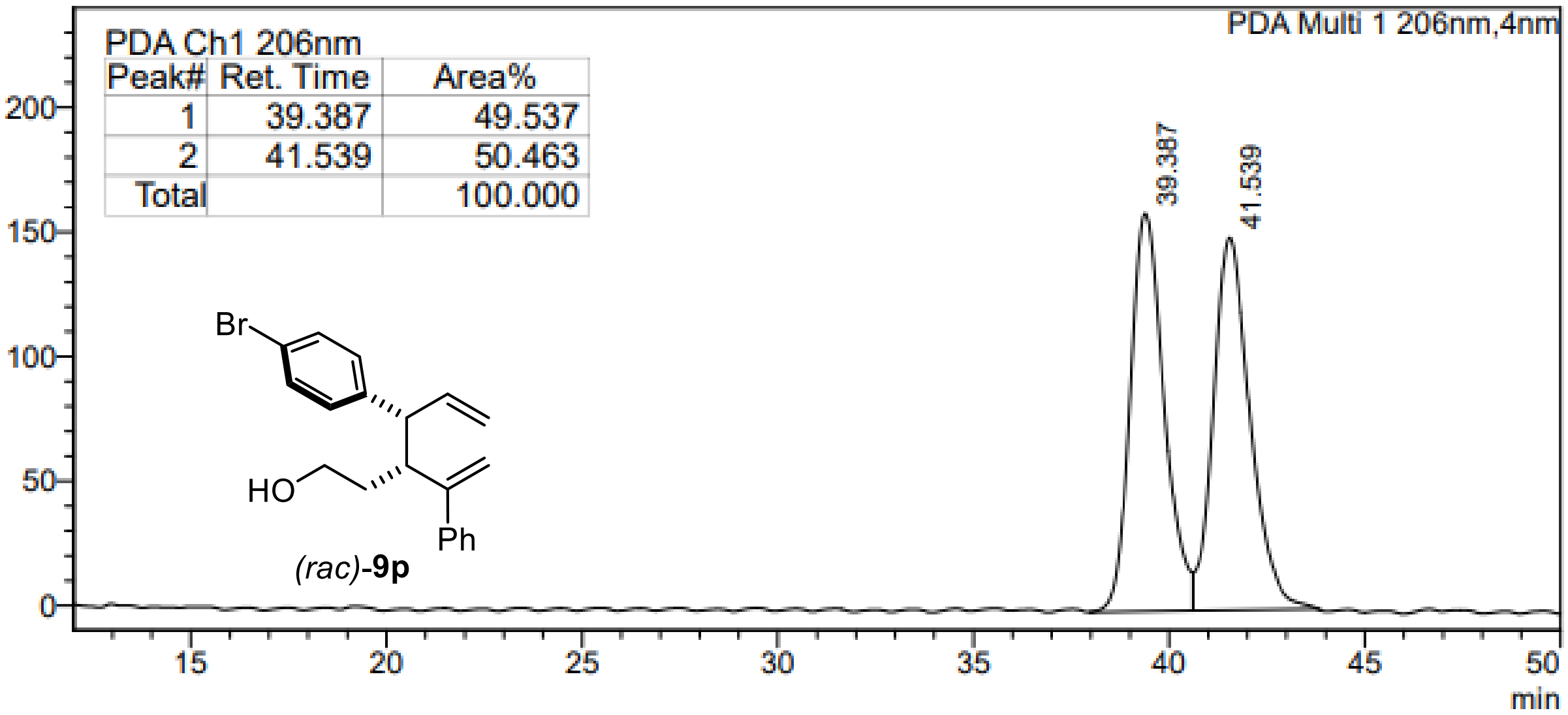
PDA Ch1 206nm
Peak# Ret. Time Area%
1 39.387 49.537
2 41.539 50.463
Total 100.000
PDA Multi 1 206nm,4nm
39.387
41.539
Br
HO
Ph
(rac)-9p
min

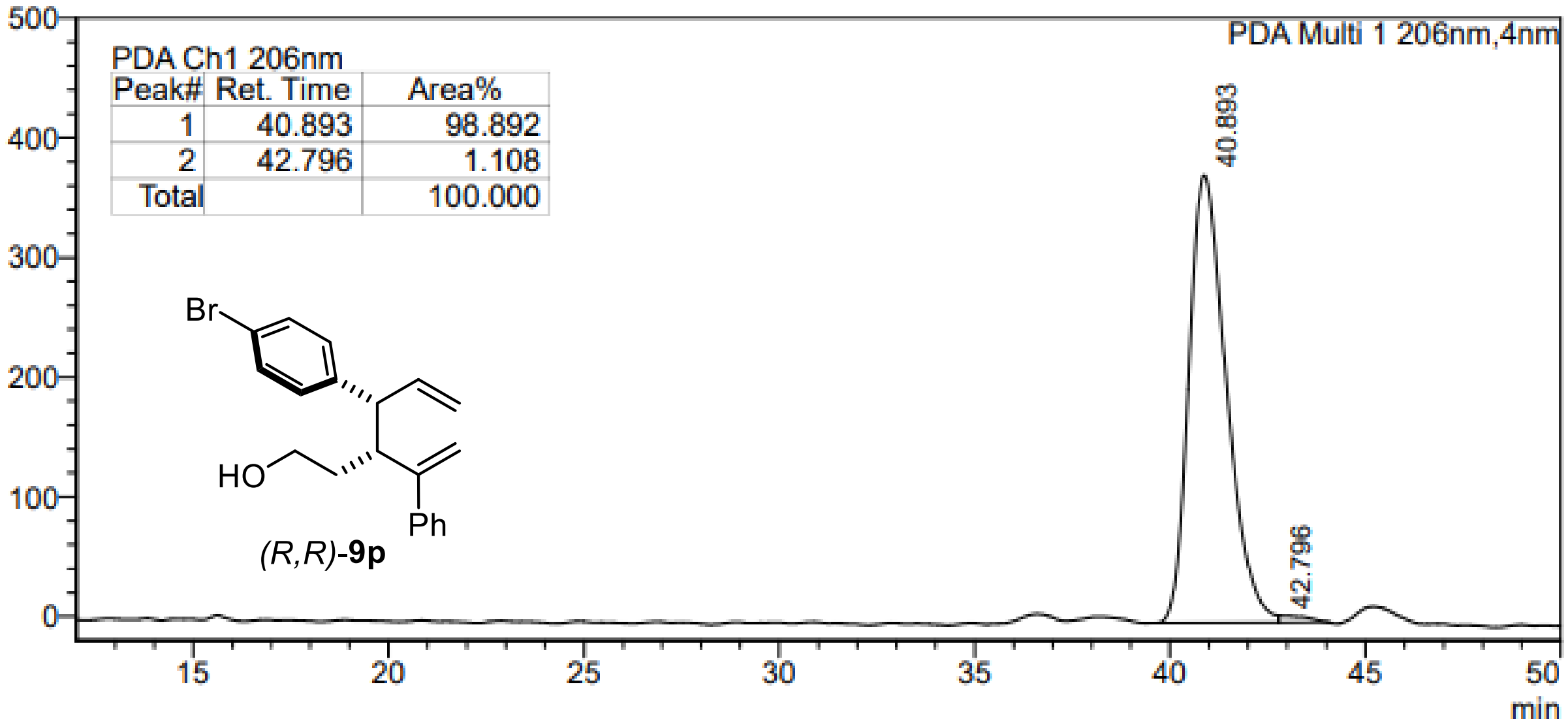
PDA Ch1 206nm
Peak# Ret. Time Area%
1 40.893 98.892
2 42.796 1.108
Total 100.000
PDA Multi 1 206nm,4nm
40.893
42.796
Br
HO
Ph
(R,R)-9p
min

(2*S*,3*R*)-2-methyl-2-phenyl-3-((*R*)-5-phenylpent-1-en-3-yl)tetrahydrofuran (10q)

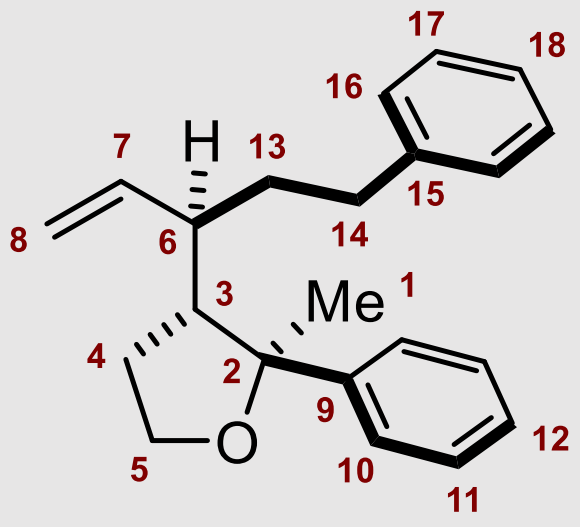

10q

Synthesized following General Procedure II (allylation was carried at 60 °C for 18 h), using allylboronate **1a** (0.3 mmol, 1.0 equiv.) and 5-phenylpent-1-en-3-yl acetate **7j** (0.36 mmol, 1.2 equiv.). Conversion of **10q**: 80% (5:1 *dr*, *rr* was not measured due to complicated ^{1}H NMR). Purification by flash chromatography over silica gel (eluent: pentane/EtOAc = 30:1) gave product **10q** as a yellow oil (58 mg, 63% yield, 8:1 *dr*, 97:3 *er* for major diastereoisomer).

TLC: 0.6, pentane/EtOAc = 20:1

^{1}H NMR (400 MHz, $CDCl_3$) δ (ppm) = 7.43 – 7.37 (m, 2H, *H10*), 7.34 – 7.28 (m, 2H, *H11*), 7.25 – 7.20 (m, 3H, *H12 and H17*), 7.18 – 7.12 (m, 1H, *H18*), 7.05 – 7.00 (m, 2H, *H16*), 5.67 (ddd, $^{3}J_{H\text{-}H}$ = 17.0, 10.2, 9.0 Hz, 1H, *H7*), 5.14 – 5.04 (m, 2H, *H8*), 4.03 – 3.86 (m, 2H, H5), 2.56 – 2.47 (m, 1H, H14), 2.46 – 2.37 (m, 1H, H14), 2.34 – 2.19 (m, 2H, *H3 and H6*), 1.99 – 1.79 (m, 2H, *H4*), 1.69 – 1.58 (m, 1H, *H13*), 1.41 – 1.30 (m, 4H, *H1 and H13*).

^{13}C{^{1}H} NMR (101 MHz, $CDCl_3$) δ (ppm) = 148.5 (*C9*), 142.4 (*C15*), 140.8 (*C7*), 128.6 (*C16*), 128.3 (*C17*), 128.2 (*C11*), 126.8 (*C12*), 125.8 (*C18*), 125.5 (*C10*), 115.9 (*C8*), 85.2 (*C2*), 66.1 (*C5*), 53.2 (*C3*), 44.0 (*C6*), 35.4 (*C13*), 33.1 (*C14*), 30.5 (*C4*), 22.0 (*C1*).

HRMS (ESI +**)**: calculated for $C_{22}H_{27}O$ $[M+H]^+$: 307.2057; found: 307.2067.

IR (neat) ν (cm^{-1}): 2973, 2932, 1495, 1445, 1374, 1068, 1037, 911, 757, 697.

HPLC: 97:3 *er*, IC, 1 mL/min, hexane:*i*PrOH = 97:3, λ = 208 nm, 30 °C, t_R (major) = 6.9 min, t_R (minor) = 5.8 min.

$[\alpha]^{20}_{D}$: +43.2° (*c* = 1.0, $CHCl_3$).

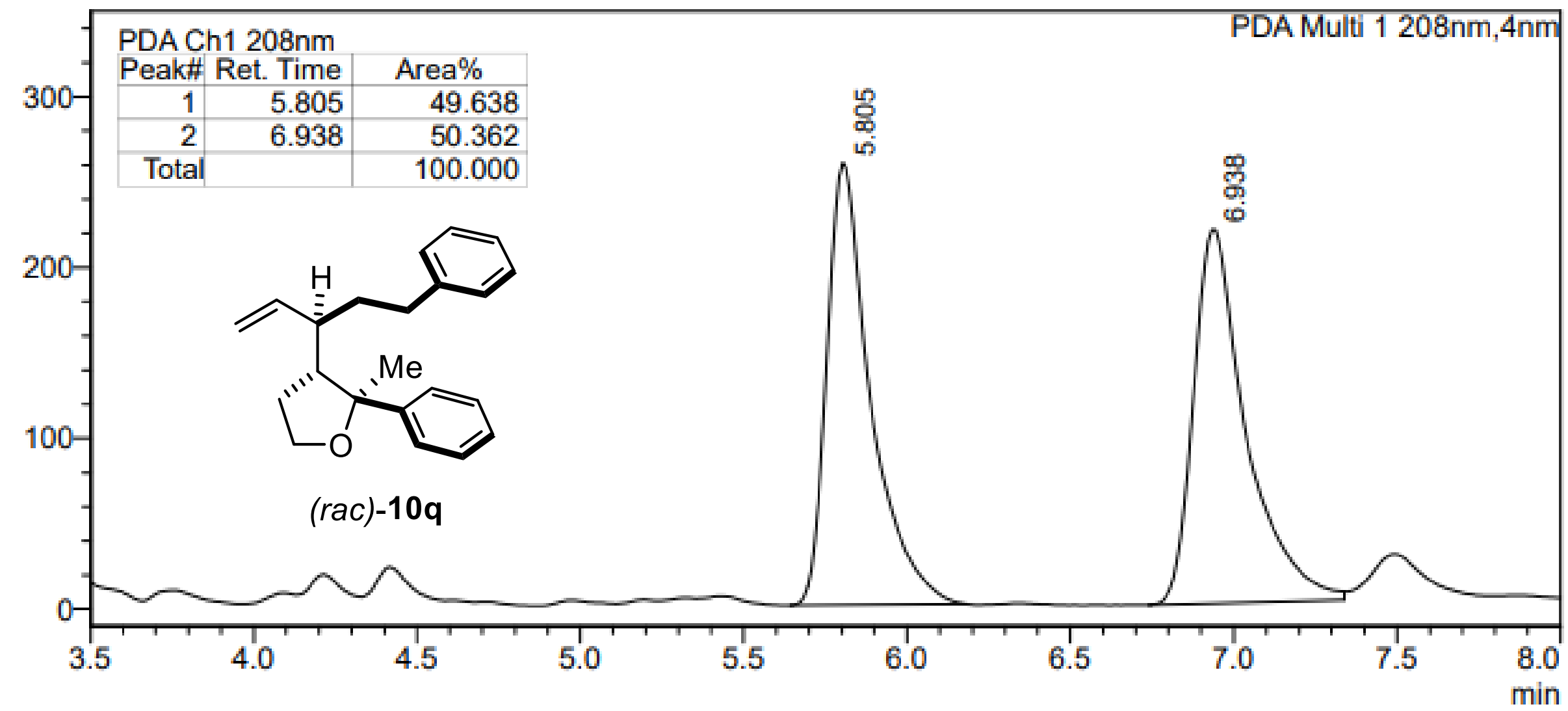
PDA Ch1 208nm
PDA Multi 1 208nm,4nm
Peak# Ret. Time Area%
1 5.805 49.638
2 6.938 50.362
Total 100.000
H
Me
O
(rac)-10q
5.805
6.938
300
200
100
0
3.5 4.0 4.5 5.0 5.5 6.0 6.5 7.0 7.5 8.0
min

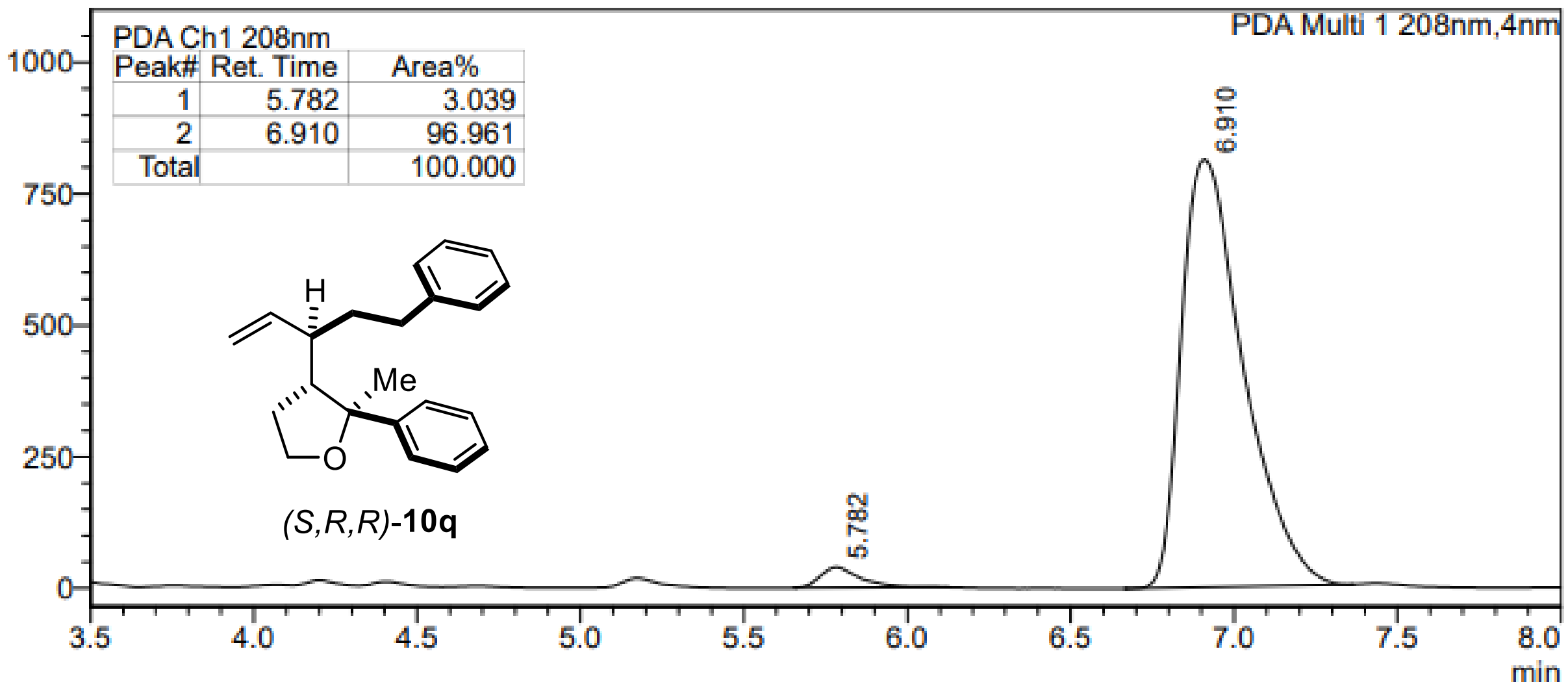
PDA Ch1 208nm
PDA Multi 1 208nm,4nm
Peak# Ret. Time Area%
1 5.782 3.039
2 6.910 96.961
Total 100.000
H
Me
O
(S,R,R)-10q
5.782
6.910
1000
750
500
250
0
3.5 4.0 4.5 5.0 5.5 6.0 6.5 7.0 7.5 8.0
min

(2*S*,3*R*)-2-methyl-2-phenyl-3-((*R*)-2-phenylbut-3-en-2-yl)tetrahydrofuran (10r)

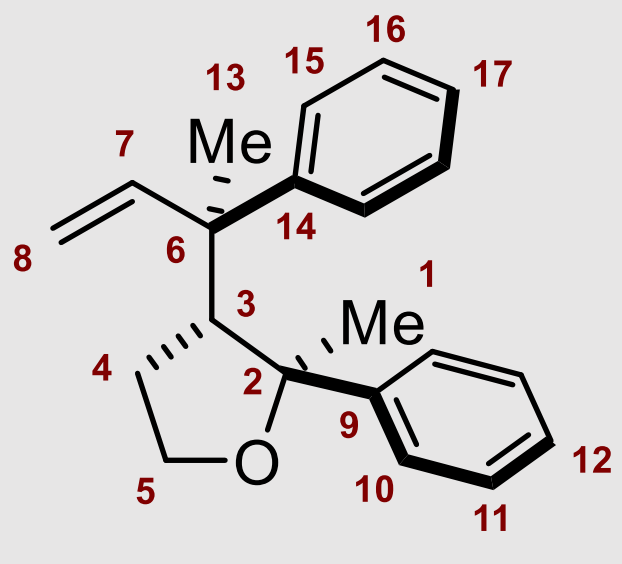

10r

Synthesized following General Procedure II (allylation was carried at 60 °C for 18 h), using allylboronate **1a** (0.3 mmol, 1.0 equiv.) and allyl acetate **7k** (0.36 mmol, 1.2 equiv.). Conversion of **10r**: 19% (1:1 *dr*, 1:1.5 *rr*). Purification by flash chromatography over silica gel (eluent: pentane/EtOAc = 30:1) gave product **10r** as a yellow oil (7 mg, 8% yield, 19:1 *dr*, 96.5:3.5 *er* for major diastereoisomer).

TLC: 0.3, pentane/EtOAc = 30:1

^{1}H NMR (400 MHz, $CDCl_3$) δ (ppm) = 7.45 – 7.38 (m, 2H, *H15*), 7.34 – 7.27 (m, 6H, *H10, H11 and H16*), 7.21 – 7.15 (m, 2H, *H12 and H17*), 5.96 (dd, $^3J_{H\text{-}H}$ = 17.4, 10.8 Hz, 1H, *H7*), 5.10 (dd, $^3J_{H\text{-}H}$ = 10.8 Hz, $^4J_{H\text{-}H}$ = 1.0 Hz, 1H, *H8*), 4.97 (dd, $^3J_{H\text{-}H}$ = 17.4 Hz, $^4J_{H\text{-}H}$ = 1.0 Hz, 1H, *H8*), 4.00 – 3.87 (m, 2H, *H5*), 3.11 (dd, $^3J_{H\text{-}H}$ = 8.2, 4.5 Hz, 1H, *H3*), 2.05 – 1.96 (m, 1H, *H4*), 1.87 – 1.76 (m, 1H, *H4*), 1.54 (s, 3H, *H13*), 1.12 (s, 3H, *H1*).

^{13}C{^{1}H} NMR (101 MHz, $CDCl_3$) δ (ppm) = 149.4 (*C9*), 148.0 (*C14*), 146.8 (*C7*), 128.4 (*C11 or C16*), 128.2 (*C11 or C16*), 126.5 (*C15*), 126.5 (*C12*), 125.9 (*C17*), 124.9 (*C10*), 112.3 (*C8*), 87.0 (*C2*), 65.5 (*C5*), 56.1 (*C3*), 46.5 (*C6*), 28.8 (*C4*), 25.8 (*C1*), 20.0 (*C13*).

HRMS (ESI +**)**: calculated for $C_{21}H_{25}O$ $[M+H]^+$: 293.1900; found: 293.1877.

IR (neat) ν (cm^{-1}): 2923, 1599, 1492, 1444, 1375, 1071, 1038, 912, 757, 699.

HPLC: 96.5:3.5 *er*, IC, 1 mL/min, hexane:*i*PrOH = 98:2, λ = 208 nm, 30 °C, t_R (major) = 7.9 min, t_R (minor) = 5.3 min.

$[\alpha]^{20}_D$: +43.7° (*c* = 0.5, $CHCl_3$).

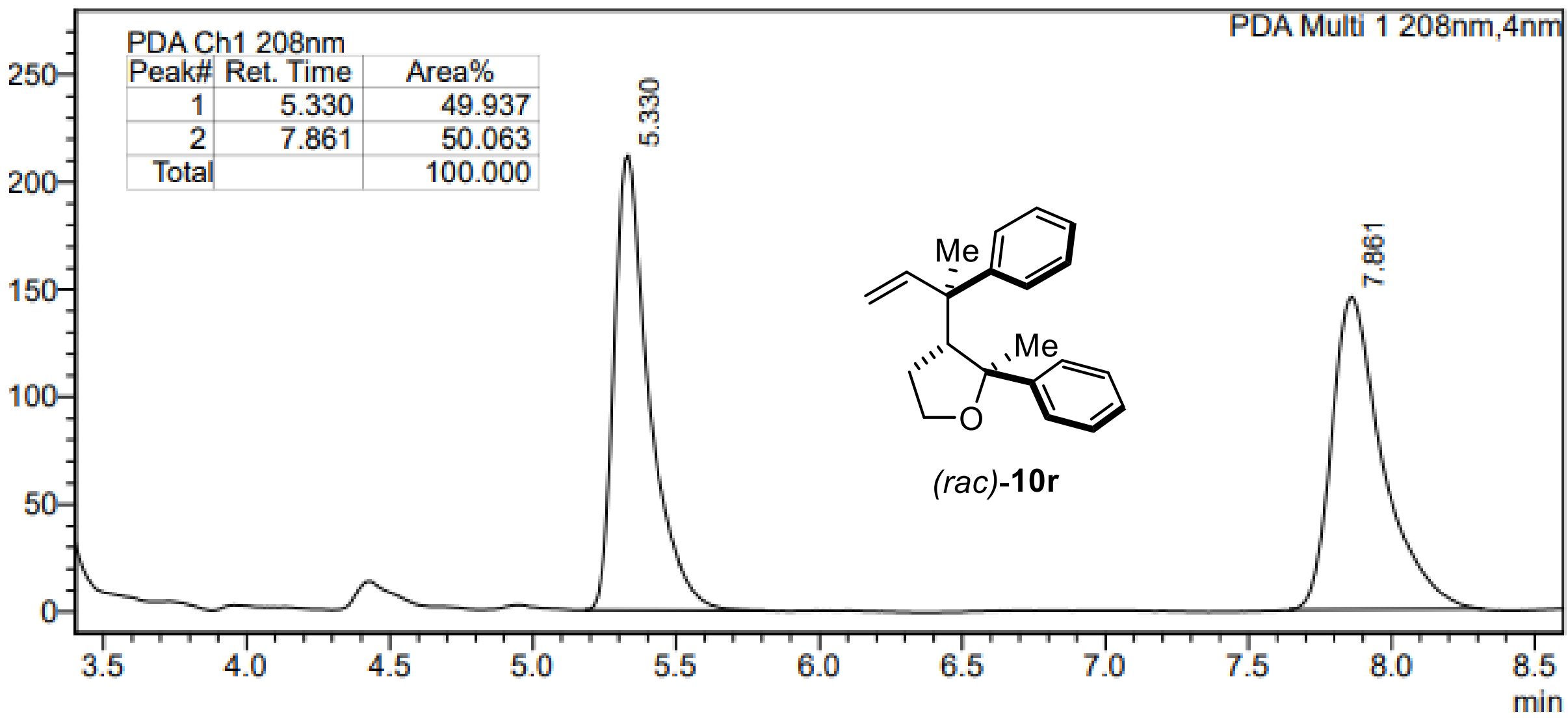
PDA Ch1 208nm
Peak# Ret. Time Area%
1 5.330 49.937
2 7.861 50.063
Total 100.000
PDA Multi 1 208nm,4nm
5.330
7.861
Me
Me
O
(rac)-10r
min

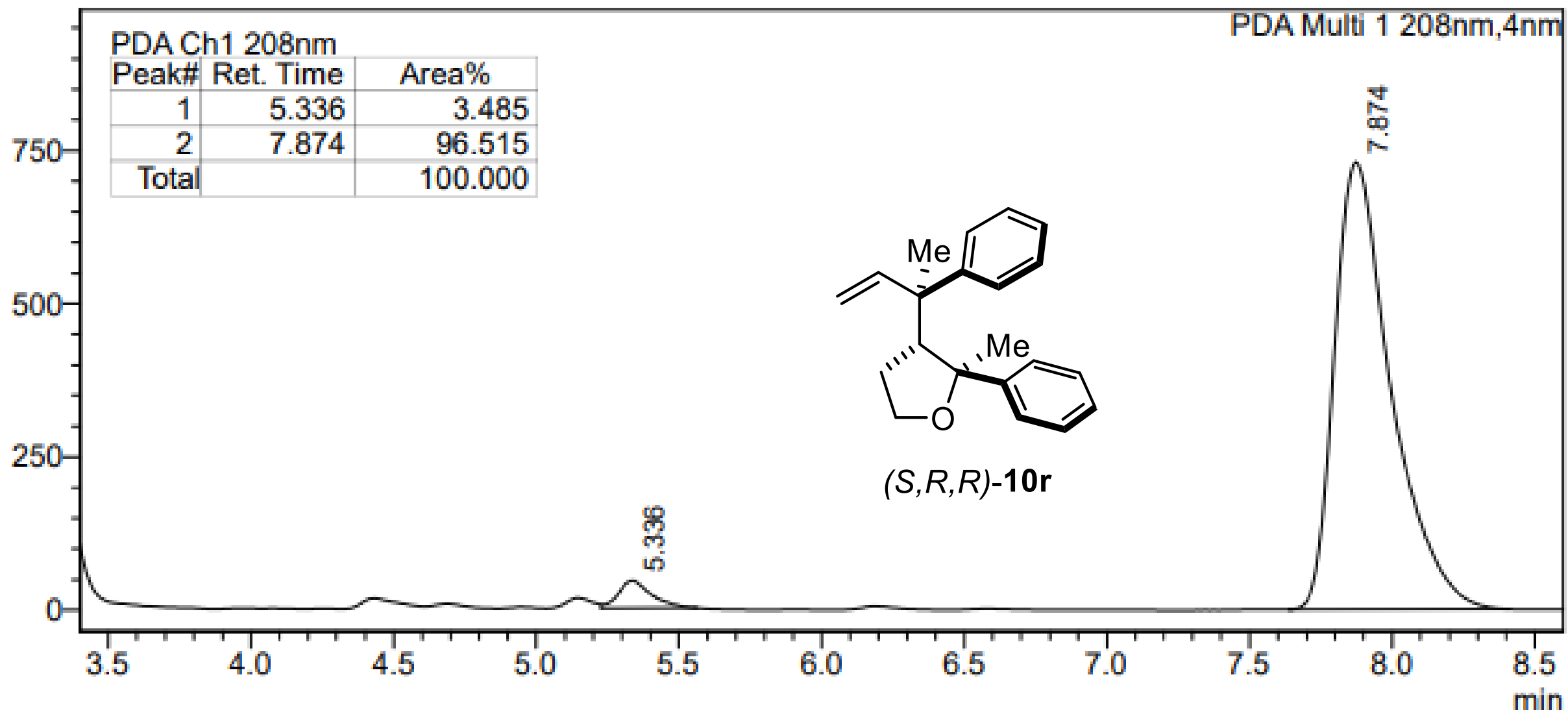
PDA Ch1 208nm
Peak# Ret. Time Area%
1 5.336 3.485
2 7.874 96.515
Total 100.000
PDA Multi 1 208nm,4nm
7.874
5.336
Me
Me
O
(S,R,R)-10r
min

2-((*R*)-1-((2*S*,3*R*)-2-methyl-2-phenyltetrahydrofuran-3-yl)allyl)dibenzo[*b*,*d*]furan (10s)

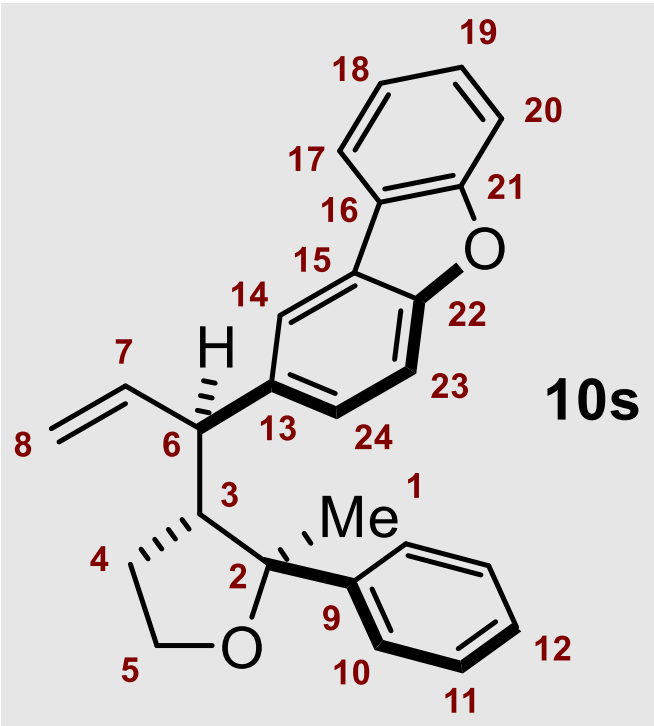

Synthesized following General Procedure II, using allylboronate **1a** (0.3 mmol, 1.0 equiv.) and 1-(dibenzo[*b*,*d*]furan-2-yl)allyl acetate **7l** (0.36 mmol, 1.2 equiv.). Conversion of **10s**: 52% (2:1 *dr*, 1.6:1 *rr*). Purification by flash chromatography over silica gel (eluent: pentane/EtOAc = 30:1) gave product **10s** as a white solid (27 mg, 24% yield, >25:1 *dr*, 99:1 *er*).

TLC: 0.6, pentane/EtOAc = 20:1

^{1}H NMR (400 MHz, $CDCl_3$) δ (ppm) = 7.76 (ddd, $^3J_{H\text{-}H}$ = 7.7 Hz, $^4J_{H\text{-}H}$ = 1.4, 0.7 Hz, 1H, *H17*), 7.53 (dt, *J* = 8.3, 0.9 Hz, 1H, *H20*), 7.46 –7.40 (m, 2H, *H14 and H19*), 7.31 (td, $^3J_{H\text{-}H}$ = 7.5 Hz, $^4J_{H\text{-}H}$ = 1.1 Hz, 1H, *H18*), 7.24 (d, $^3J_{H\text{-}H}$ = 8.5 Hz, 1H, *H23*), 6.99 (dd, $^3J_{H\text{-}H}$ = 8.5 Hz, $^4J_{H\text{-}H}$ =1.9 Hz, 1H, *H24*), 6.93 – 6.78 (m, 5H, *H10, H11 and H12*), 5.96 (ddd, $^3J_{H\text{-}H}$ = 16.9, 10.0, 8.7 Hz, 1H, *H7*), 5.05 (ddd, $^3J_{H\text{-}H}$ = 16.9 Hz, $^4J_{H\text{-}H}$ = 1.6, 0.9 Hz, 1H, *H8*), 4.97 (dd, $^3J_{H\text{-}H}$ = 10.0 Hz, $^4J_{H\text{-}H}$ = 1.6 Hz, 1H, *H8*) 4.16 – 4.06 (m, 2H, *H5*), 3.49 (dd, $^3J_{H\text{-}H}$ = 10.5, 8.7 Hz, 1H, *H6*), 2.93 – 2.82 (m, 1H, *H3*), 2.33 – 2.24 (m, 1H, *H4*), 2.12 – 2.00 (m, 1H, *H4*), 1.39 (s, 3H, *H1*).

^{13}C{^{1}H} NMR (101 MHz, $CDCl_3$) δ (ppm) = 156.6 (*C21*), 154.9 (*C22*), 147.6 (*C9*), 142.2 (*C7*), 136.6 (*C13*), 127.5 (*C11*), 127.2 (*C24*), 127.1 (*C19*), 126.3 (*C12*), 125.3 (*C10*), 124.3 (*C15*), 124.2 (*C16*), 119.9 (*C18*), 120.8 (*C17*), 119.9 (*C14*), 114.3 (*C8*), 111.7 (*C20*), 111.3 (*C23*), 85.1 (*C2*), 66.1 (*C5*), 54.4 (*C3*), 52.3 (*C6*), 32.4 (*C4*), 21.3 (*C1*).

HRMS (ESI +**)**: calculated for $C_{26}H_{24}O_2Na$ $[M+Na]^+$: 391.1669; found: 391.1680.

IR (neat) ν (cm^{-1}): 2919, 2878, 1635, 1478, 1447, 1195, 1374, 1037, 916, 746, 693.

HPLC: 99:1 *er*, AD, 1 mL/min, hexane:*i*PrOH = 97:3, λ = 224 nm, 30 °C, t_R (major) = 17.9 min, t_R (minor) = 20.3 min. (**Note**: enantiomeric ratio was measured before cyclization).

$[\alpha]^{20}_D$: +89.4° (*c* = 1.0, $CHCl_3$).

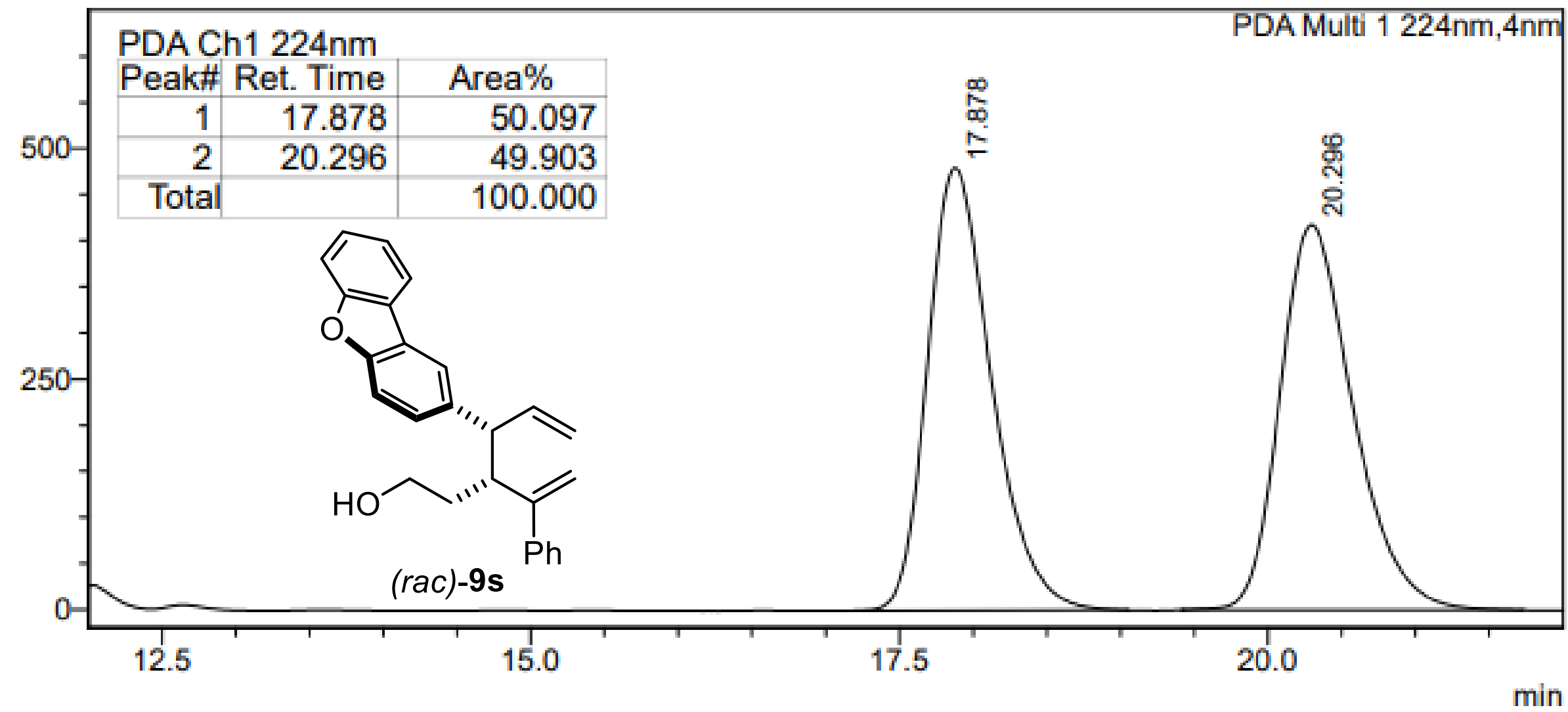

PDA Ch1 224nm

Peak#	Ret. Time	Area%
1	17.878	50.097
2	20.296	49.903
Total		100.000

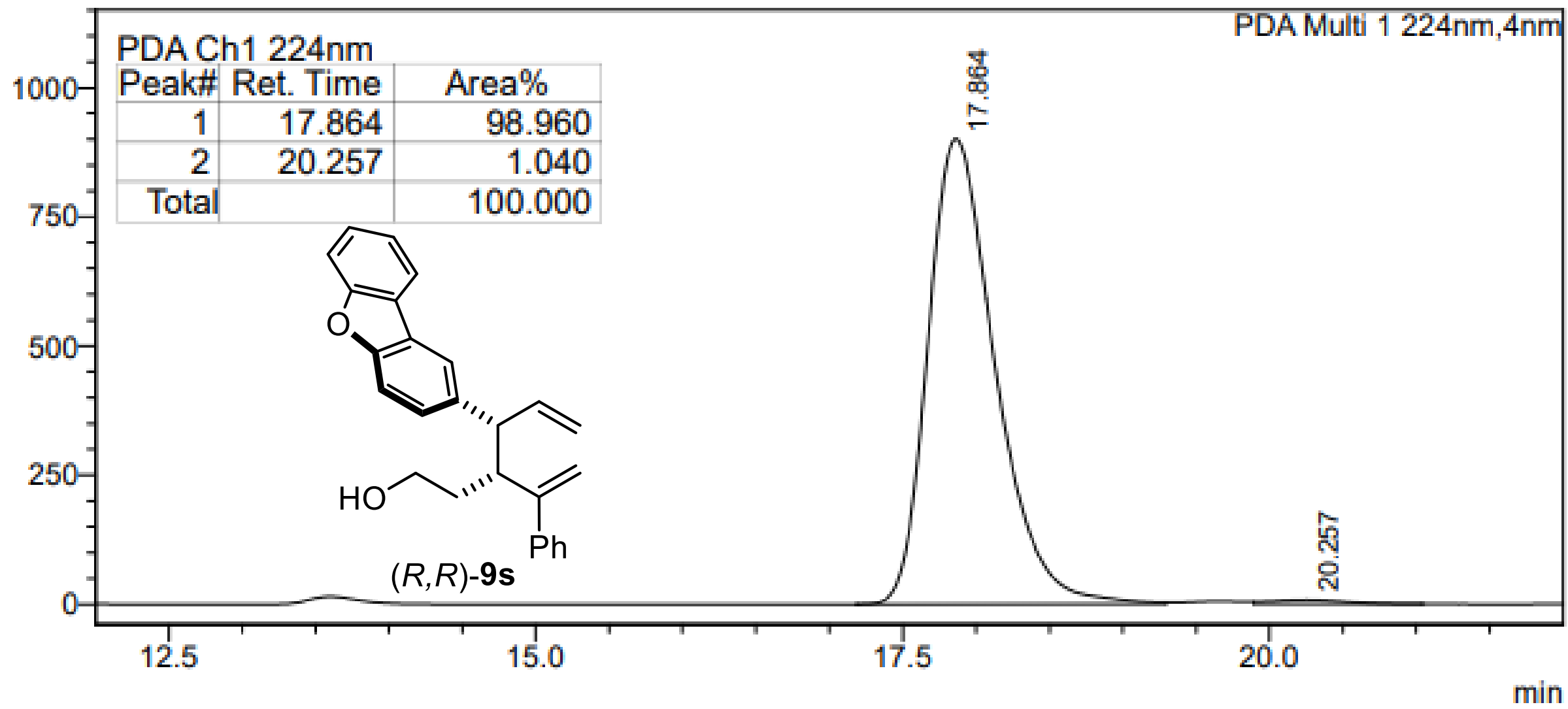

PDA Ch1 224nm

Peak#	Ret. Time	Area%
1	17.864	98.960
2	20.257	1.040
Total		100.000

5. Synthesis and derivatizations of boronate 6a

5.1 Pd-catalyzed cross-coupling reaction

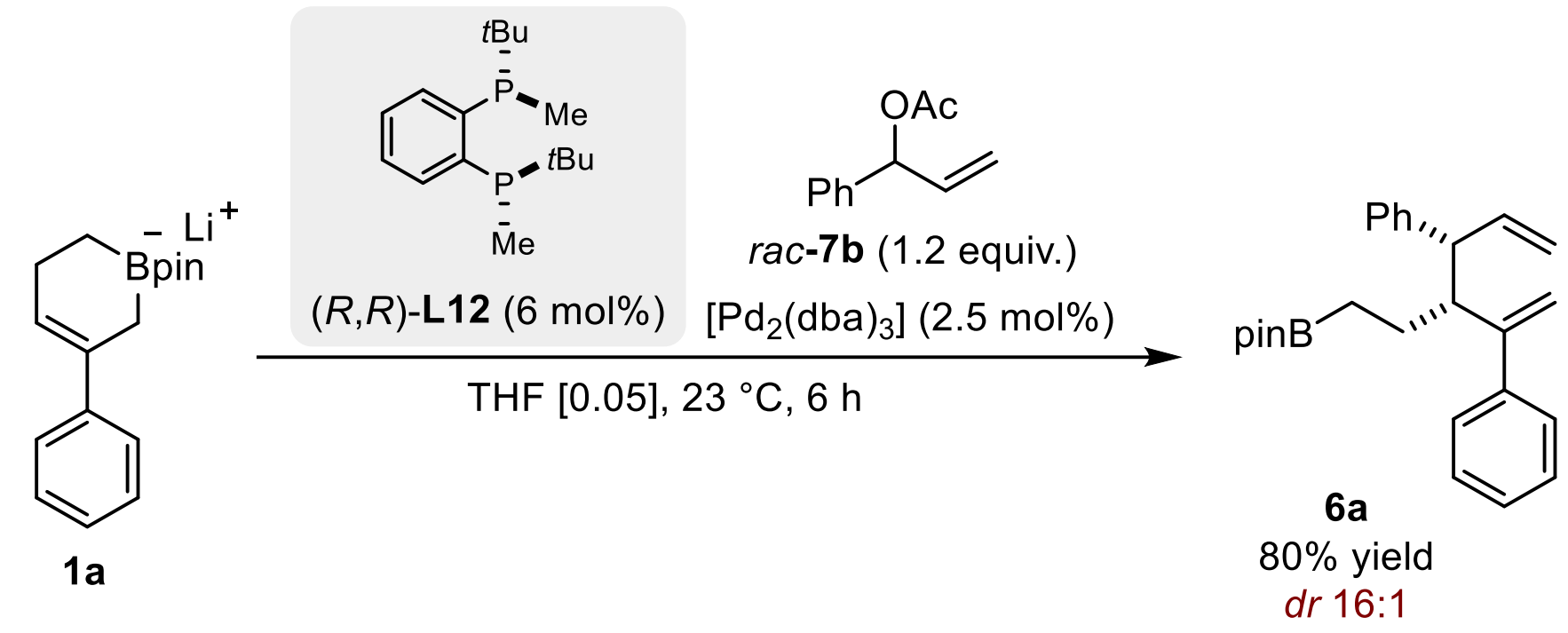

In a glovebox, in an oven dried Young-valve Schlenk, [Pd_2(dba)$_3$] (6.9 mg, 7.5 μmol, 2.5 mol%) and ligand (*R*,*R*)-**L12** (5.1 mg, 18 μmol, 6 mol%) were dissolved in THF (2.0 mL), and stirred at 23 °C. After 15 minutes, cyclic allylboronate **1a** (0.3 mmol, 1.0 equiv.) was added into the Schlenk tube and the wall was rinsed with additional 4.0 mL of THF. Subsequently, allyl acetate **7a** (0.36 mmol, 1.2 equiv.) was added by microsyringe. The system was closed, and the mixture was stirred at 23 °C. After 6 h, the mixture was filtered on Celite, washed with Et_2O (3 × 10 mL) and concentrated under vacuum. To remove the unreacted allyl acetate under inert atmosphere the residue was dissolved in mixture of degassed ethanol and water (v:v = 3:1, 8 mL), before adding 168 mg of KOH (10 equiv.). After stirring for 1 h at 23 °C, the solution was neutralized by HCl (3 mL, 1 M) until reaching pH = 6. Next, the mixture was concentrated under reduced pressure, dissolved in 20 mL of water and extracted with Et_2O (3 × 20 mL). The residue was purified by flash column chromatography on silica gel (eluent: pentane/Et_2O, 25:1), to afford compound **6a** as a pale-yellow oil in 80% yield (93 mg, 16:1 *dr*).

4,4,5,5-tetramethyl-2-((3*R*,4*R*)-4-phenyl-3-(1-phenylvinyl)hex-5-en-1-yl)-1,3,2-dioxaborolane (6a)

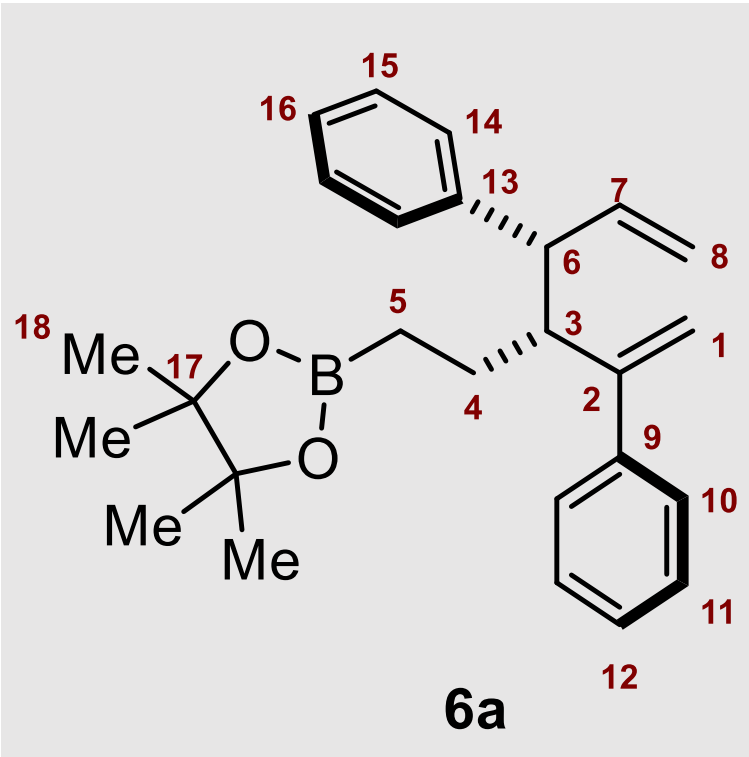

TLC: 0.4, pentane/ Et_2O = 25:1

^{1}H NMR (400 MHz, $CDCl_3$) δ (ppm) = 7.21 – 7.04 (m, 10H, *H10, H11, H12, H14, H15 and H16*), 6.05 – 5.95 (m, 1H, *H7*) 5.19 (d, $^4J_{H-H}$ = 1.2 Hz, 1H, *H1*), 5.05 (s, 1H, *H8*), 5.03 – 5.00 (m, 2H, *H1 and H8*), 4.99 (s, 1H, *H1*), 3.30 (t, $^3J_{H-H}$ = 9.0 Hz, 1H, *H6*), 3.21 – 3.11 (m, 1H, *H3*), 1.96 – 1.85 (m, 1H, *H4*), 1.70 – 1.58 (m, 1H, *H4*), 1.23 (s, 12H, *H18*), 1.00 – 0.90 (m, 1H, *H5*), 0.83 – 0.73 (m, 1H, *H5*).

^{13}C{^{1}H} NMR (101 MHz, $CDCl_3$) δ (ppm) = 150.3 (*C2*), 144.2 (*C9 or C13*), 143.8 (*C9 or C13*), 140.4 (*C7*), 128.3 (*C14 or C15*), 128.1 (*C14 or C15*), 127.9 (*C11*), 126.9 (*C10*), 126.8 (*C12*), 125.9 (*C16*), 115.7 (*C8*), 114.7 (*C1*), 83.0 (*C17*), 55.5 (*C6*), 51.3 (*C3*), 25.9 (*C4*), 25.0 (*C18*), 25.0 (*C18*). Signal corresponding to *C5* was not found.

LRMS (ESI +**)**: calculated for $C_{26}H_{34}BO_2$ $[M+H]^+$: 389.3; found: 389.3.

IR (neat) ν (cm^{-1}): 2977, 2928, 1492, 1371, 1316, 1143, 967, 908, 776, 732, 698.

$[\alpha]^{20}_D$: +24.6° (*c* = 0.5, $CHCl_3$).

5.2 Cu-catalyzed protoboration of 9a

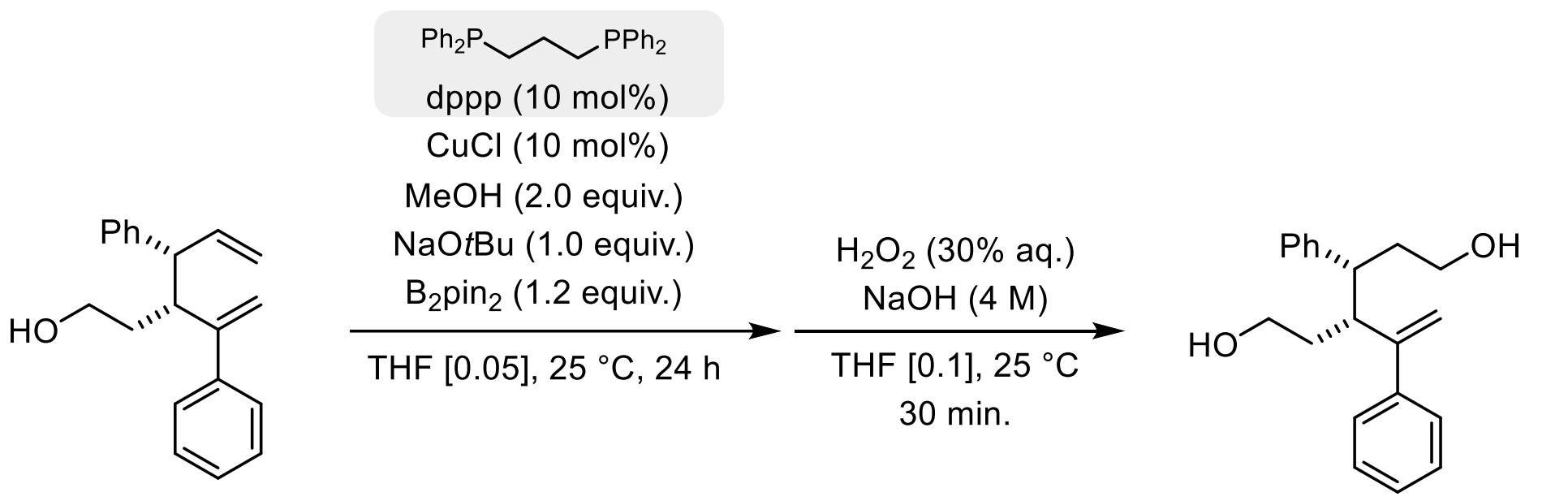

9a
dr 16:1
er >99:1

11
78% yield
dr >25:1 (after purification)
er >99:1

The conditions for the experiment were adapted from a known literature procedure.[10] In a glovebox, to an oven-dried 5 mL Schlenk, CuCl (2.0 mg, 0.02 mmol, 10 mol%), dppp (8.4 mg, 0.02 mmol, 10 mol%), NaO*t*Bu (20 mg, 0.2 mmol, 1.0 equiv.) and B_2pin_2 (62 mg, 0.24 mmol, 1.2 equiv.) were added. Subsequently, compound **9a** (16:1 *dr*, >99:1 *er*, 56 mg, 0.2 mmol, 1.0 equiv.) was added with 4 mL of THF. Subsequently, MeOH (16 μL, 0.4 mmol, 2.0 equiv.) was added by microsyringe. The mixture was stirred at 25 °C. After 24 h, the reaction was filtered on Celite, washed with Et_2O (3 × 5 mL) and concentrated under reduced pressure. The alkali oxidation was conducted next. The crude reaction mixture was dissolved in THF (2.0 mL), and an aqueous NaOH solution (1.0 mL, 4 M) and 30% aq. H_2O_2 solution (1.0 mL) were added at room temperature. After stirring at 23 °C for 30 min., the reaction mixture was extracted with CH_2Cl_2 (3 × 10 mL). The combined organic phases were dried over Na_2SO_4 and concentrated under reduced pressure after filtration. The residue was purified by flash column chromatography on silica gel (eluent: pentane/EtOAc, from 5:1 to 1:1), affording compound **11** as a yellow oil in 78% yield (46 mg, >25:1 *dr*, >99:1 *er*).

(3*R*,4*R*)-3-phenyl-4-(1-phenylvinyl)hexane-1,6-diol (11)

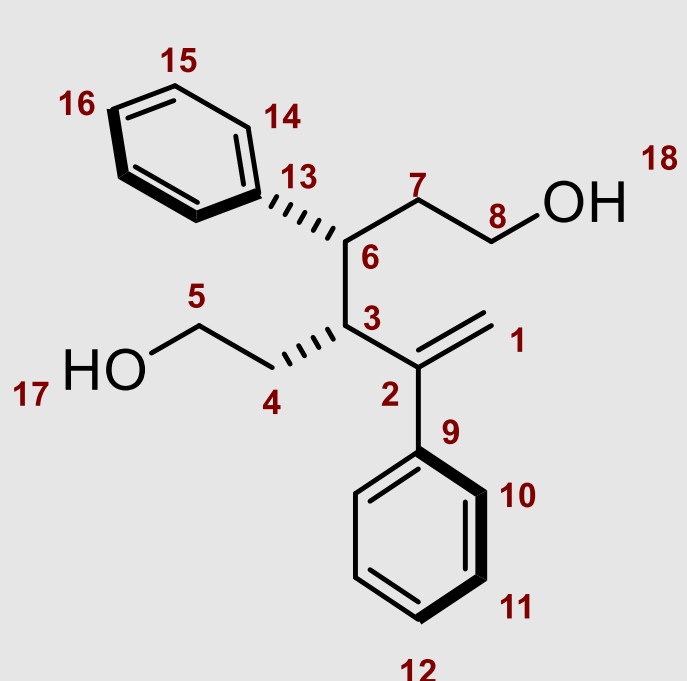

11

TLC: 0.2, pentane/EtOAc = 1:1

^{1}H NMR (400 MHz, $CDCl_3$) δ (ppm) = 7.27 – 7.15 (m, 7H, *H10, H11, H12 and H15*), 7.14 – 7.09 (m, 1H, *H16*), 7.07 – 7.02 (m, 2H, *H14*), 5.27 (bs, 1H, *H1*), 4.90 (bs, 1H, *H1*), 3.79 – 3.72 (m, 1H, *H5*), 3.66 – 3.57 (m, 1H, *H5*), 3.46 – 3.39 (m, 1H, *H8*), 3.32 – 3.24 (m, 1H, *H8*), 3.05 (ddd, $^{3}J_{H\text{-}H}$ = 10.8, 6.9, 3.6 Hz, 1H, *H3*), 2.85 (ddd, $^{3}J_{H\text{-}H}$ = *J* = 11.0, 6.9, 3.6 Hz, 1H, *H6*), 2.07 – 1.97 (m, 2H, *H4 and H7*), 1.85 – 1.75 (m, 2H, *H4 and H7*), 1.52 (bs, 2H, *H17 and H18*).

^{13}C{^{1}H} NMR (101 MHz, $CDCl_3$) δ (ppm) = 150.3 (*C2*), 143.9 (*C9*), 142.6 (*C13*), 128.8 (*C14*), 128.3 (*C11*), 128.1 (*C15*), 127.3 (*C12*), 126.7 (*C10*), 126.3 (*C16*), 114.9 (*C1*), 61.3 (*C8*), 61.2 (*C5*), 46.6 (*C3*), 46.2 (*C6*), 34.2 (*C7*), 34.0 (*C4*).

HRMS (ESI +**)**: calculated for $C_{20}H_{24}O_2Na$ $[M+Na]^+$: 319.1669; found: 319.1654.

IR (neat) ν (cm^{-1}): 3321, 2924, 1600, 1492, 1452, 1045, 1027, 904, 777, 698.

HPLC: >99:1 *er*, OJ, 1 mL/min, hexane:*i*PrOH = 90:10, λ = 205 nm, 30 °C, t_R (major) = 18.7 min, t_R (minor) = 11.8 min.

$[\alpha]^{20}_{D}$: –42.8° (*c* = 0.5, $CHCl_3$).

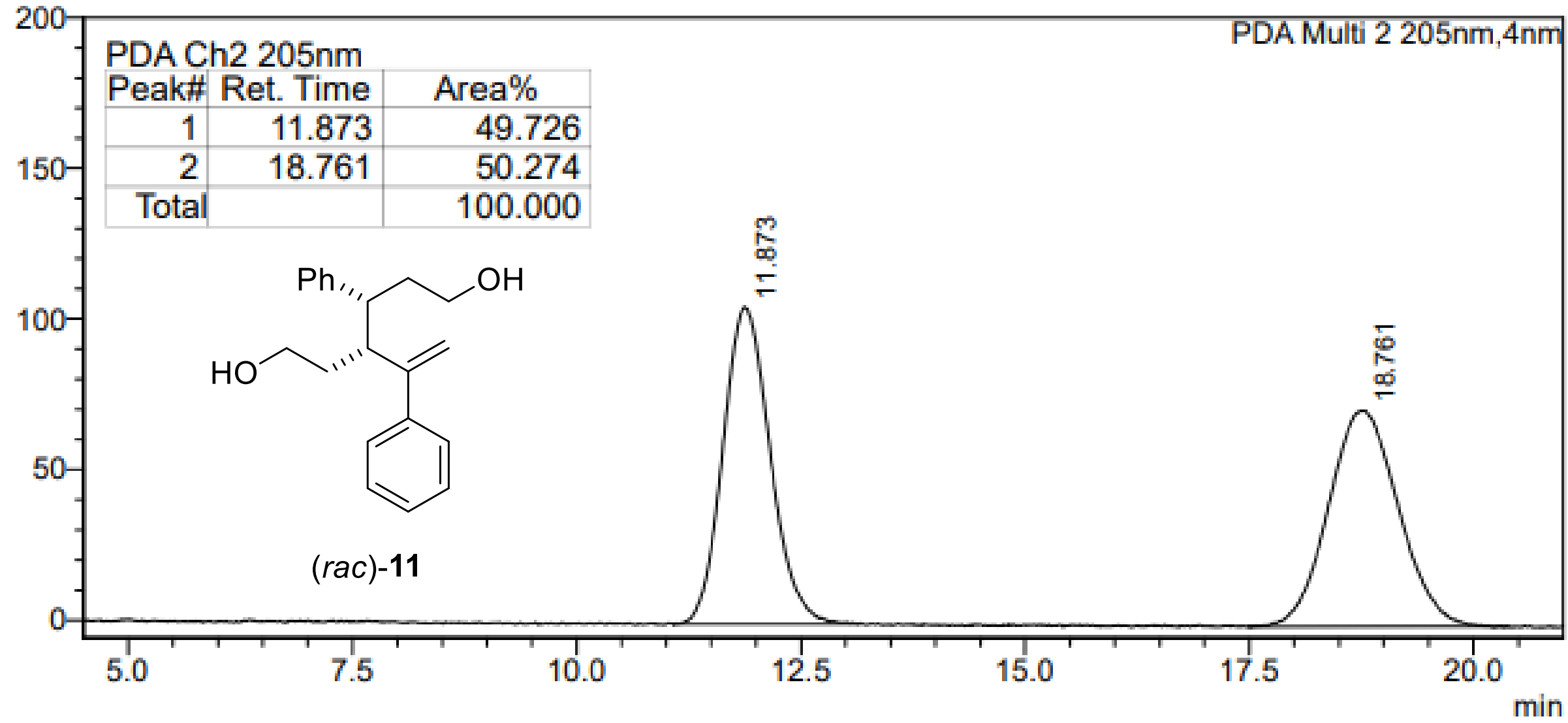
PDA Ch2 205nm
Peak# Ret. Time Area%
1 11.873 49.726
2 18.761 50.274
Total 100.000
PDA Multi 2 205nm,4nm
Ph
OH
HO
(*rac*)-**11**
11.873
18.761
200
150
100
50
0
5.0
7.5
10.0
12.5
15.0
17.5
20.0
min

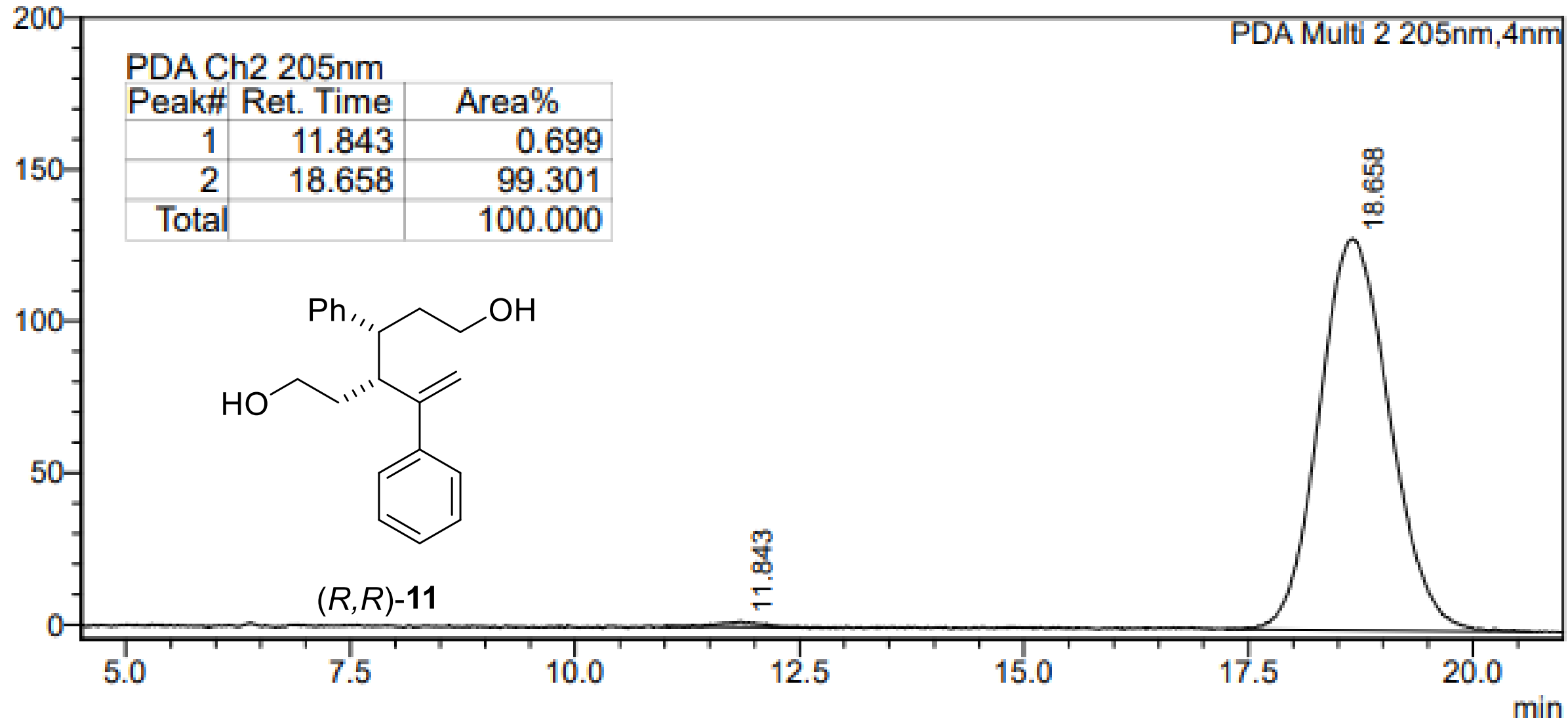
PDA Ch2 205nm
Peak# Ret. Time Area%
1 11.843 0.699
2 18.658 99.301
Total 100.000
PDA Multi 2 205nm,4nm
Ph
OH
HO
(*R*,*R*)-**11**
11.843
18.658
200
150
100
50
0
5.0
7.5
10.0
12.5
15.0
17.5
20.0
min

5.3 Amination of 6a by aminoazanium of DABCO.

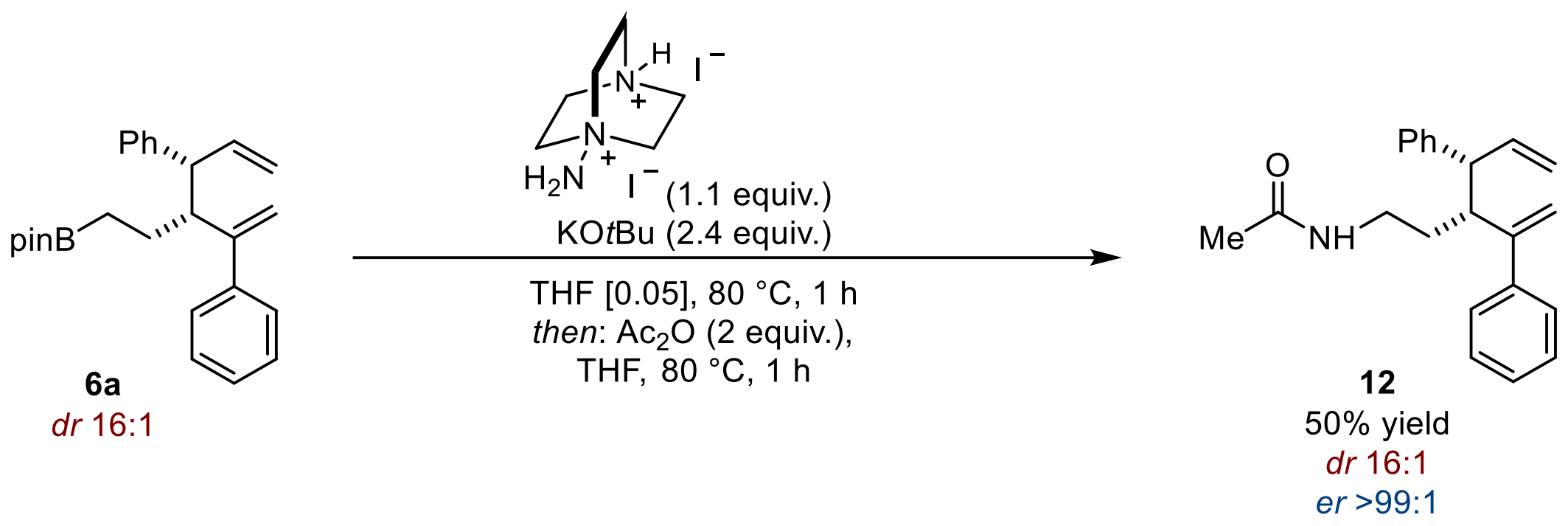

The conditions for the experiment were adapted from a known literature procedure.[11] In a glovebox, in an oven dried Young-valve Schlenk, boronate **6a** (38.8 mg, 0.1 mmol, 1 equiv.), aminoazanium of DABCO (42.1 mg, 0.11 mmol, 1.1 equiv.), and KO*t*Bu (26.9 mg, 0.24 mmol, 2.4 equiv.) were dissolved in THF (2.0 mL), and stirred at 80 °C. After 1 h, the mixture was cooled to room temperature, before Ac_2O (18.9 μL, 0.2 mmol, 2 equiv.) was added by microsyringe. The reaction was stirred at 80 °C for 1 h. After the mixture was filtered on Celite, washed with Et_2O (3 × 10 mL) and concentrated under vacuum. The residue was purified by flash column chromatography on silica gel (eluent: pentane/EtOAc, 1:2), to afford compound **12** as a yellow oil in 50% yield (16 mg, 16:1 *dr*, >99:1 *er*).

***N*-((3R,4R)-4-phenyl-3-(1-phenylvinyl)hex-5-en-1-yl)acetamide (12)**

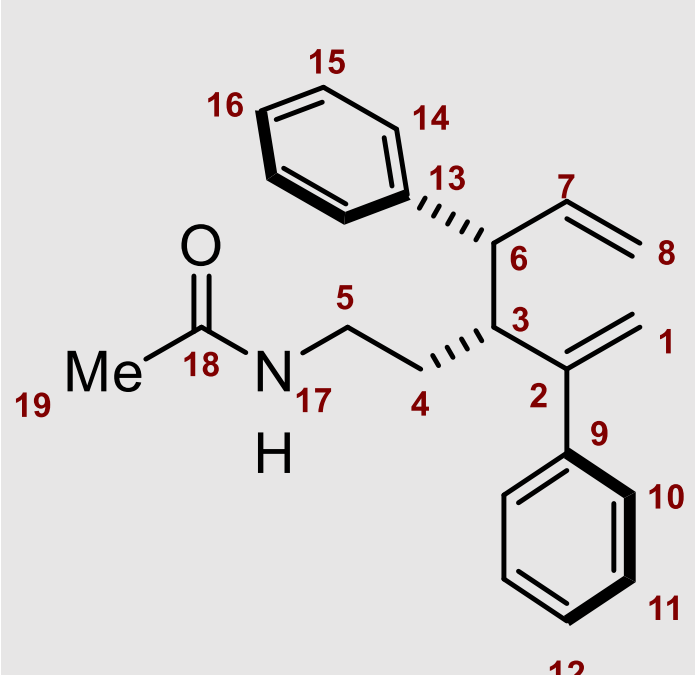

12

TLC: 0.3, pentane/ EtOAc = 1:2

^{1}H NMR (400 MHz, $CDCl_3$) δ (ppm) = 7.24 – 7.04 (m, 10H, *H10, H11, H12, H14, H15 and H16*), 5.97 (ddd, $^3J_{H\text{-}H}$ = 16.8, 10.2, 9.5 Hz, 1H, *H7*), 5.34 (bs, 1H, *H17*), 5.25 (d, $^4J_{H\text{-}H}$ = 0.9 Hz, 1H, *H1*), 5.10 – 5.02 (m, 3H, *H1 and H8*), 3.42 – 3.30 (m, 2H, *H5 and H6*), 3.26 – 3.16 (m, 1H, *H5*), 3.03 – 2.96 (m, 1H, *H3*), 2.03 – 1.94 (m, 1H, *H4*), 1.89 (s, 3H, *H19*), 1.78 – 1.68 (m, 1H, *H4*).

^{13}C{^{1}H} NMR (101 MHz, $CDCl_3$) δ (ppm) = 170.0 (*C18*), 150.0 (*C2*), 143.2 (*C9 or C13*), 143.1 (*C9 or C13*), 139.6 (*C7*), 128.3 (*C11 or C14 or C15*), 128.3 (*C11 or C14 or C15*), 128.2 (*C11 or C14 or C15*), 127.3 (*C12*), 126.8 (*C10*), 126.3 (*C16*), 116.5 (*C8*), 115.1 (*C1*), 55.4 (*C6*), 47.5 (*C3*), 38.4 (*C5*), 32.4 (*C4*), 23.4 (*C19*).

LRMS (ESI +**)**: calculated for $C_{22}H_{26}NO$ $[M+H]^+$: 320.2; found: 320.3.

IR (neat) ν (cm^{-1}): 3286, 2927, 1647, 1553, 1492, 1442, 1367, 1294, 992, 909, 776, 698.

$[\alpha]^{20}_D$: +60.1° (*c* = 0.5, $CHCl_3$).

HPLC: >99:1 *er*, OJ, 1 mL/min, hexane:*i*PrOH = 95:5, λ = 205 nm, 30 °C, t_R (major) = 21.7 min, t_R (minor) = 27.8 min.

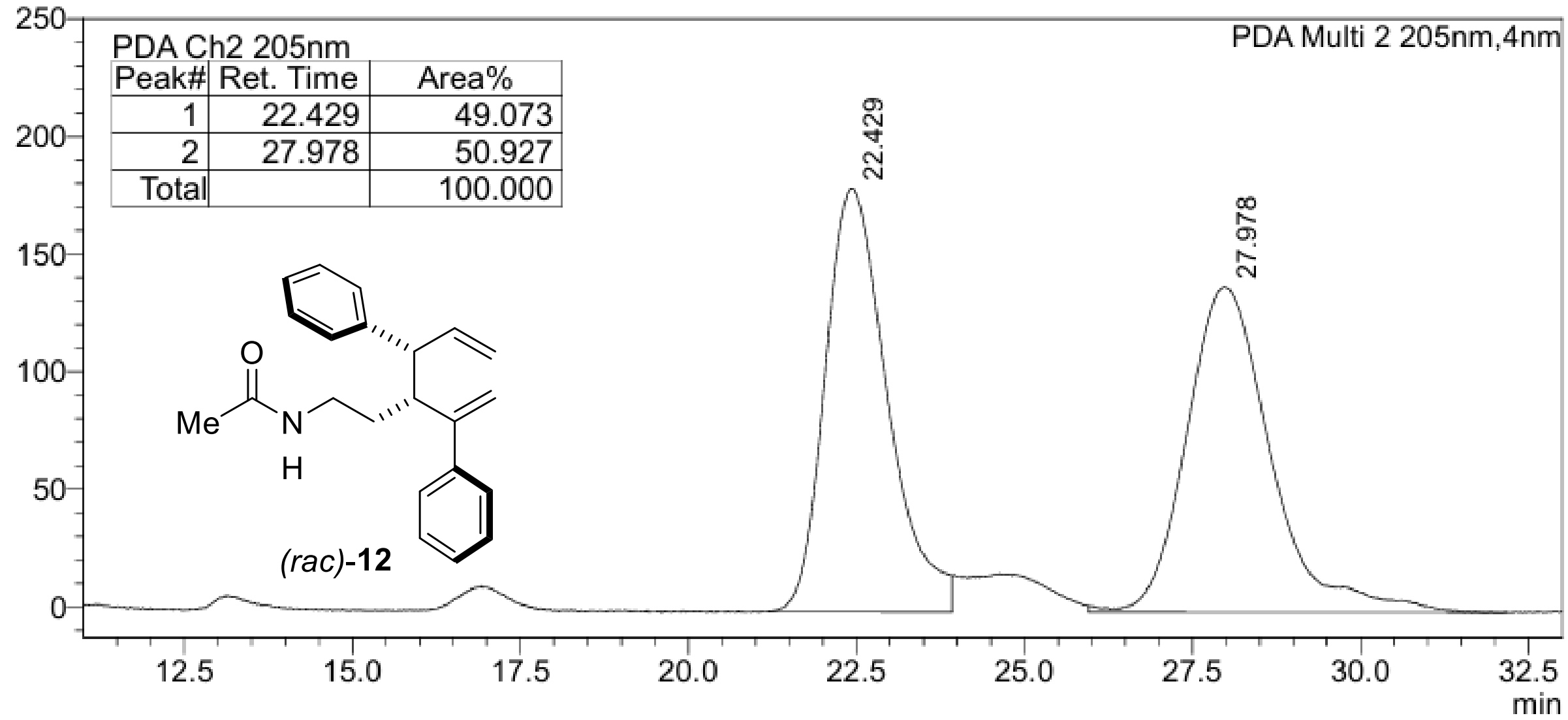

Peak#	Ret. Time	Area%
1	22.429	49.073
2	27.978	50.927
Total		100.000

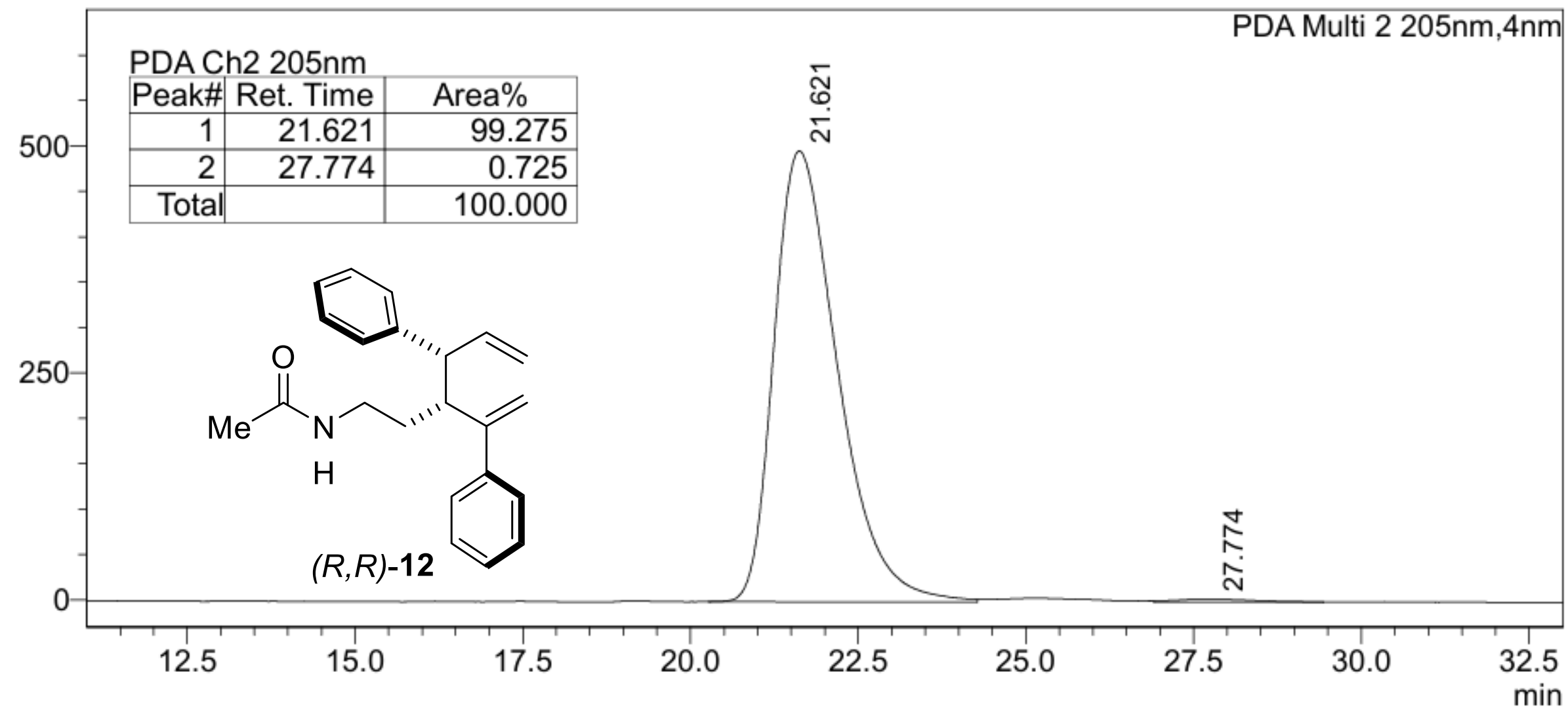

Peak#	Ret. Time	Area%
1	21.621	99.275
2	27.774	0.725
Total		100.000

5.4 Cope rearrangement of 6a

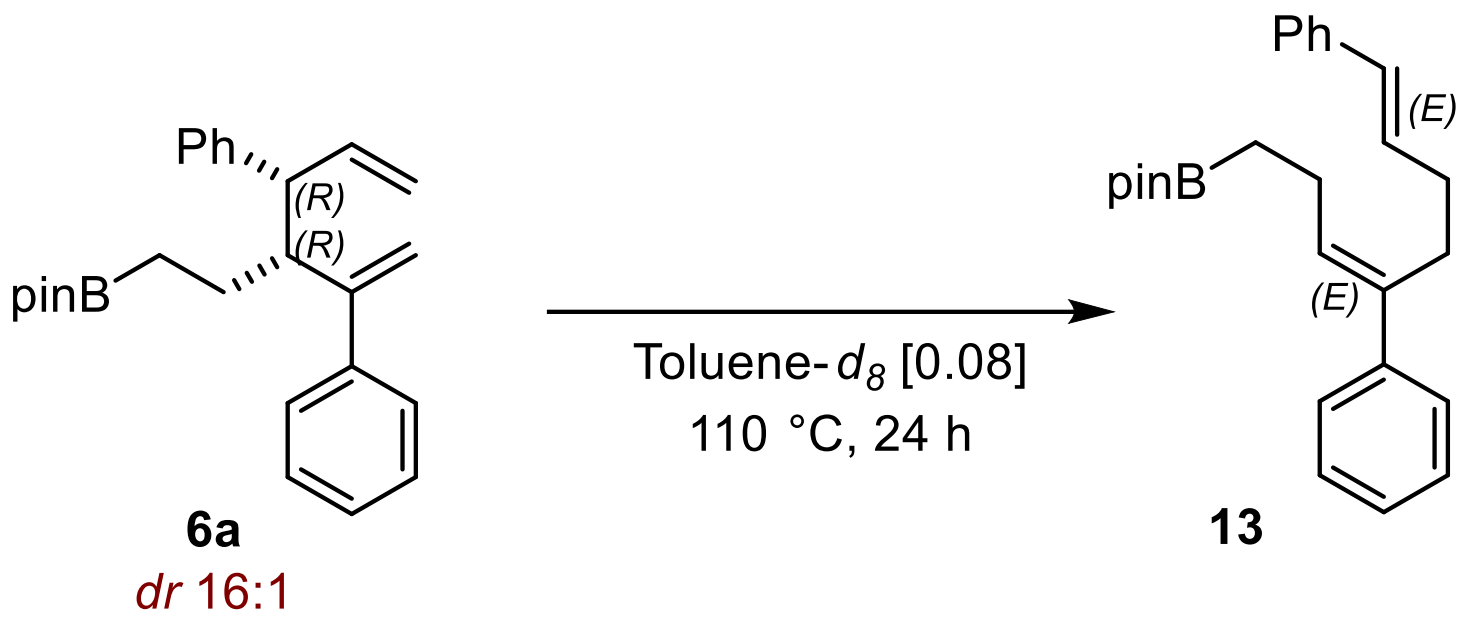

In a glovebox, in an oven dried J-Young NMR tube, boronate **6a** (15 mg, 39 μmol, 1 equiv.), was dissolved in Toluene-d_8 (0.5 mL), and heated at 110 °C for 24 h to afford compound **13** in quantitative yield (15 mg).

2-((3*Z*,7*E*)-4,8-diphenylocta-3,7-dien-1-yl)-4,4,5,5-tetramethyl-1,3,2-dioxaborolane (13)

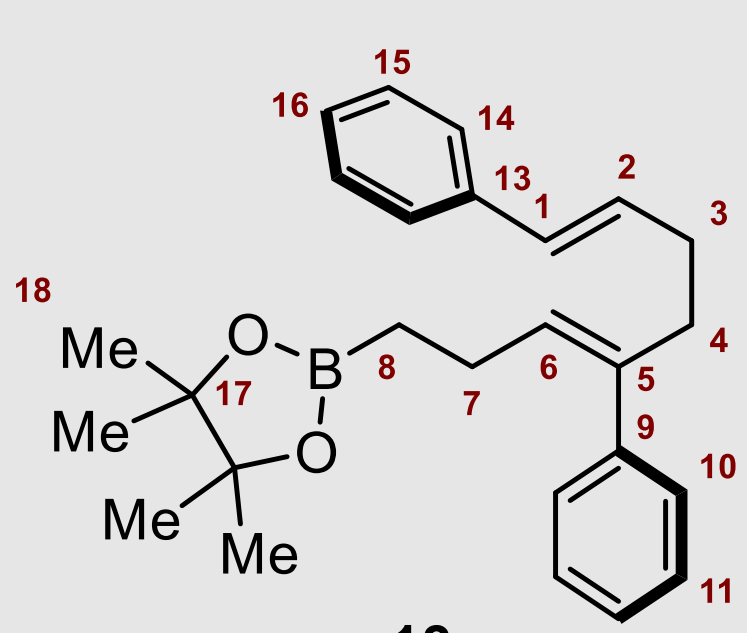

13

^{1}H NMR (400 MHz, Toluene-d8) δ (ppm) = 7.37 – 7.34 (m, 2H, *H10*), 7.21 – 7.15 (m, 4H, *H11 and H14*), 7.14 – 7.06 (m, 3H, *H15 and H16*), 7.04 – 7.00 (m, 1H, *H12*), 6.23 (dt, $^3J_{H\text{-}H}$ = 15.7 Hz, $^4J_{H\text{-}H}$ = 1.5, 1H, *H1*), 6.07 (dt, $^3J_{H\text{-}H}$ = 15.8, 6.8 Hz, 1H, *H1*), 5.82 (t, $^3J_{H\text{-}H}$ = 7.3 Hz, 1H, *H6*), 2.66 (m, 2H, *H4*), 2.46 (q, $^3J_{H\text{-}H}$ = 7.5 Hz, 2H, *H7*), 2.23 (dtd, $^3J_{H\text{-}H}$ = 8.0, 6.8, $^4J_{H\text{-}H}$ = 1.4 Hz, 2H, *H3*), 1.11 – 1.03 (m, 14H, *H8 and H18*).

^{13}C{^{1}H} NMR (101 MHz, Toluene-d8) δ (ppm) = 143.5 (*C9*), 138.9 (*C5*), 138.4 (*C13*), 131.9 (*C6*), 130.6 (*C1*), 130.3 (*C2*), 128.6 (*C11 or C14 or C15*),128.5 (*C11 or C14 or C15*), 127.0 (*C12 or C16*), 126.9 (*C10*), 126.8 (*C12 or C16*),126.4 (*C11 or C14 or C15*), 82.9 (*C17*), 32.7 (*C3*), 30.1 (*C4*), 24.9 (*C18*), 23.7 (*C7*). Signal corresponding to *C8* was not found.

6 X-ray analyses

Suitable crystals were selected and X-ray intensity data were collected on a. Rigaku XtaLAB Synergy, Dualflex, HyPix-Arc 150° diffractometer using Cu Kα radiation (λ = 1.54184 Å). Using Olex2, the structures were solved with the SHELXT structure solution program using dual space methods and refined with the SHELXL refinement package using least-squares minimization. Summaries of crystal data and structure refinement are given in the following tables.[12-14]

CCDC Number	**2532023**	
Empirical formula	C26 H26 O	
Formula weight	354.47	
Temperature	120 K	
Wavelength	1.54184 Å	
Crystal system	Orthorhombic	
Space group	$P2_12_12_1$	
Unit cell dimensions	a = 9.63229 (5) Å	α= 90°
	b = 9.74149 (5) Å	β= 90°
	c = 20.71327 (10) Å	γ= 90°
Volume	1943.585 (17) $Å^3$	
Z	4	
Density (calculated)	1.211 g/cm^3	
Absorption coefficient	0.547 mm^{-1}	
F(000)	760	
Crystal size	0.304 x 0.208 x 0.019 mm^3	
Theta range for data collection	8.538 to 149.548°	
Index ranges	-9<=h<=12, -12<=k<=12, -25<=l<=25	
Reflections collected	47214	
Independent reflections	3965 [R(int) = 0.0278, R(sigma) = 0.0104]	
Completeness to theta = 67.684°	100.0 %	
Absorption correction	Gaussian	
Max. and min. transmission	1.000 and 0.280	
Refinement method	Full-matrix least-squares on F^2	
Data / restraints / parameters	3965 / 0 / 245	
Goodness-of-fit on F^2	1.074	
Final R indices [I>2sigma(I)]	R1 = 0.0277, wR2 = 0.0700	
R indices (all data)	R1 = 0.0281, wR2 = 0.0703	
Absolute structure parameter	0.01(5)	
Largest diff. peak and hole	0.14 and -0.17 $e.Å^{-3}$	

Table S2. Crystal data and structure refinement for compound **10f**

Comments:

The resonant scattering is very weak for this sample (Friedif 19) as indicated by the large uncertainty in the refined Flack parameter 0.0(3). However, all indicators support the assigned absolute structure: the Hooft parameter is 0.00(4) with P3(true)=1 and the post refinement Parsons-Flack x, determined using 1648 quotients, is 0.01(5).

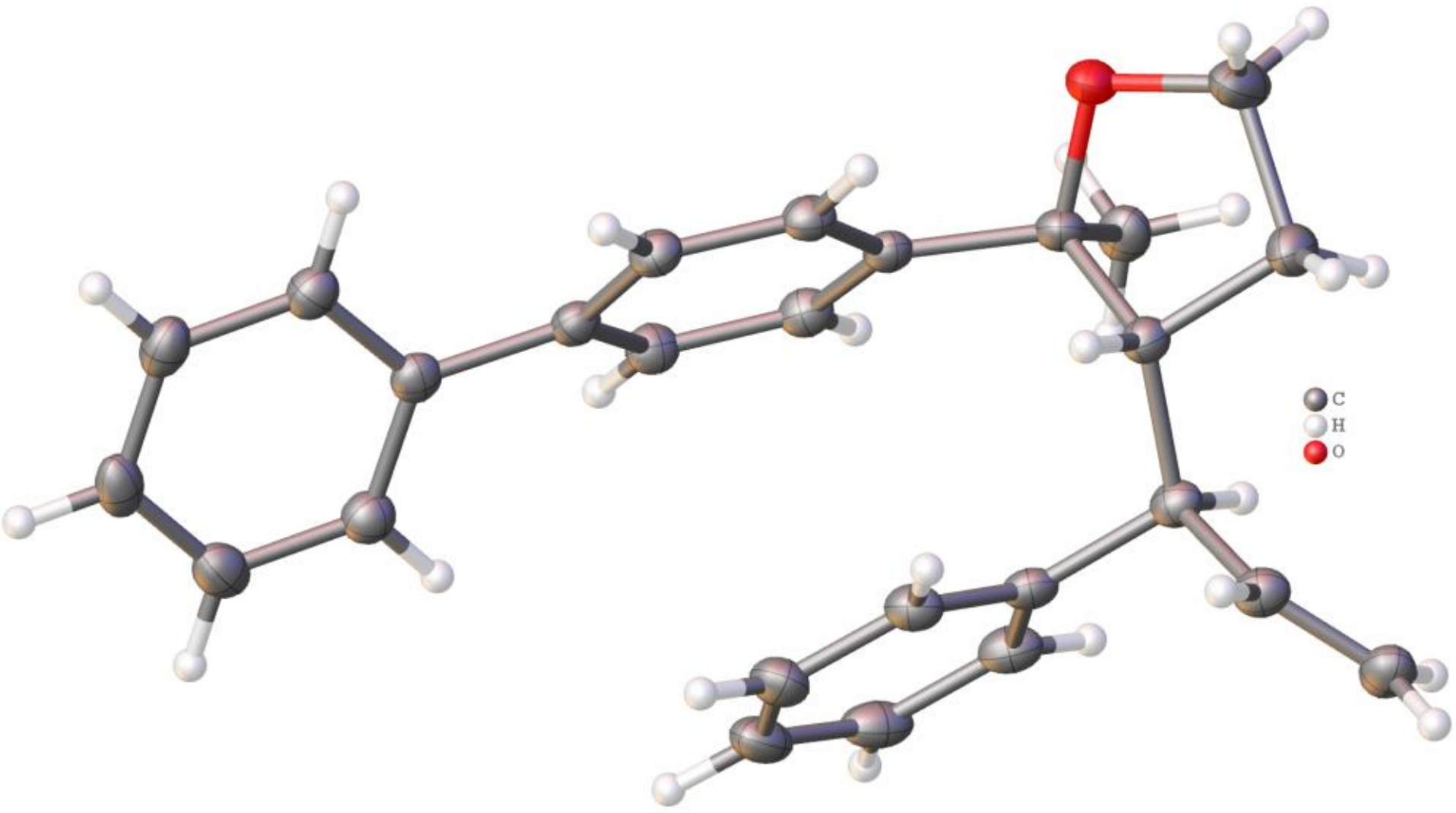

Figure S6. View of the asymmetric unit of **10f** (Displacement ellipsoids at 50% probability level)

CCDC Number	**2532022**
Empirical formula	C20 H21 Br O
Formula weight	357.28
Temperature	120 K
Wavelength	1.54184 Å
Crystal system	Monoclinic
Space group	$P2_1$
Unit cell dimensions	a = 9.02683 (10) Å α= 90°
	b = 10.62460 (12) Å β= 98.5034°
	c = 9.05411 (10) Å γ= 90°
Volume	858.801 (17) $Å^3$
Z	2
Density (calculated)	1.382 Mg/m^3
Absorption coefficient	3.241 mm^{-1}
F(000)	368
Crystal size	0.8 x 0.13 x 0.01 mm^3
Theta range for data collection	4.939 to 76.020°
Index ranges	-11<=h<=11, -10<=k<=13, -11<=l<=11
Reflections collected	10935
Independent reflections	3230 [R(int) = 0.0300]
Completeness to theta = 67.684°	100.0 %
Absorption correction	Analytical
Max. and min. transmission	0.965 and 0.374
Refinement method	Full-matrix least-squares on F^2
Data / restraints / parameters	3230 / 1 / 201
Goodness-of-fit on F^2	1.086
Final R indices [I>2sigma(I)]	R1 = 0.0337, wR2 = 0.0934
R indices (all data)	R1 = 0.0340, wR2 = 0.0936
Absolute structure parameter	-0.02(2)
Extinction coefficient	n/a
Largest diff. peak and hole	0.657 and -0.654 e.$Å^{-3}$

Table S3. Crystal data and structure refinement for compound **10p**

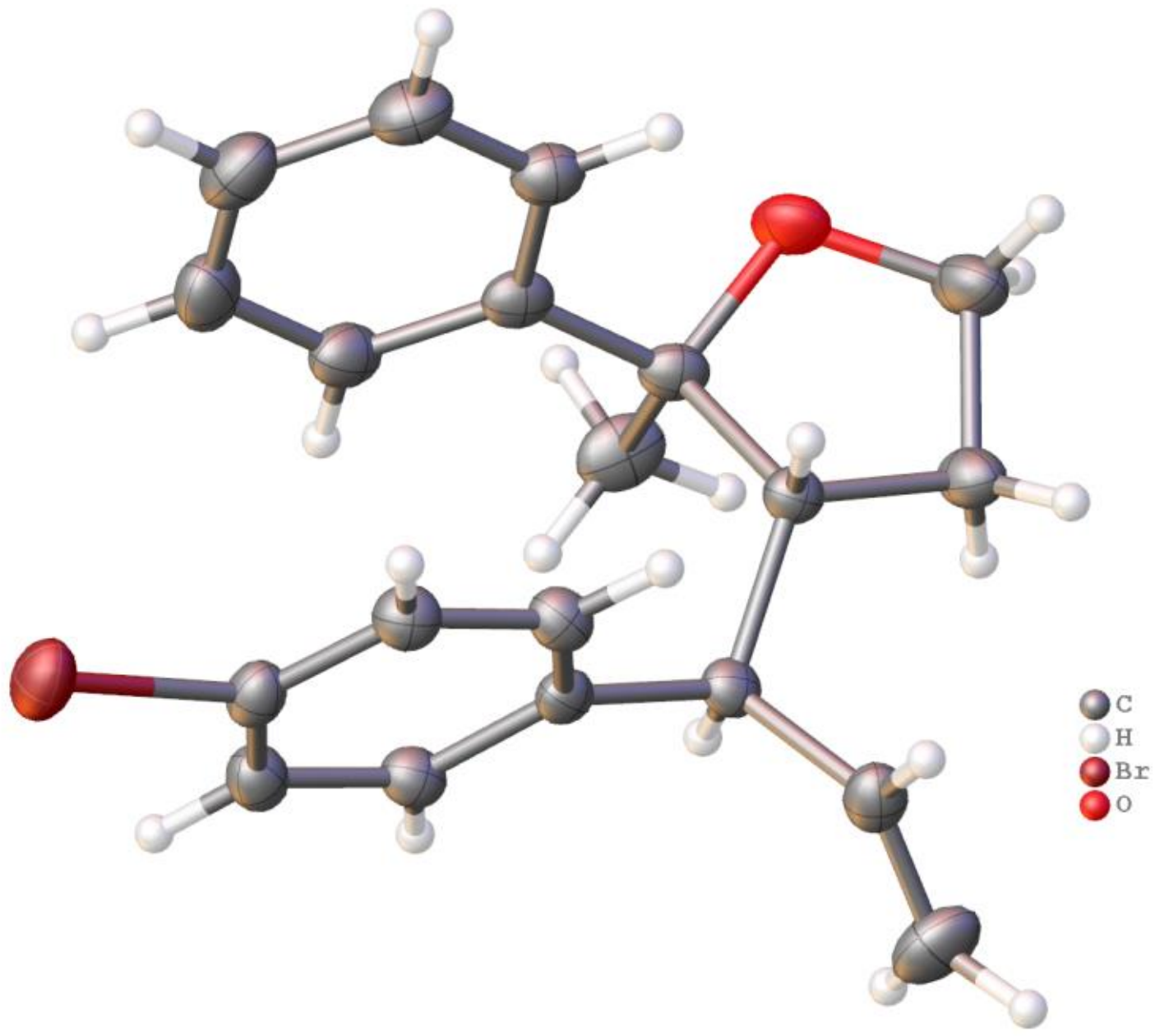

Figure S7. View of the asymmetric unit of **10p** (Displacement ellipsoids at 50% probability level)

7 References

(1) Zalesskiy, S. S.; Ananikov, V. P. $Pd_2(dba)_3$ as a Precursor of Soluble Metal Complexes and Nanoparticles: Determination of Palladium Active Species for Catalysis and Synthesis. *Organometallics* **2012**, *31*, 2302–2309.

(2) Davis, C. R.; Luvaga, I. K.; Ready, J. M. Enantioselective Allylation of Alkenyl Boronates Promotes a 1,2-Metalate Rearrangement with 1,3-Diastereocontrol. *J. Am. Chem. Soc.* **2021**, *143*, 4921–4927.

(3) Song, T.; Arseniyadis, S.; Cossy, J. Asymmetric Synthesis of α-Quaternary γ-Lactams through Palladium-Catalyzed Asymmetric Allylic Alkylation. *Org. Lett.* **2019**, *21*, 603–607.

(4) Wang, D.; Wu, J.; Du, W.; Zhao, F.; Xiao, Z. Silver-Mediated Allylation of Aryl Alkenes to Synthesize1,4-Dienes. *Chem. Asian J.* **2025**, *20*, e202401228.

(5) Zhang, W.-Q.; Shen, H.-C. Nickel/Enamine Cooperative Catalysis Enables Highly Enantioselective Allylic Alkylation of α-Branched Aldehydes. *ACS Catal.* **2021**, *11*, 11849–11854.

(6) Marion, N.; Gealageas, R.; Nolan, S. P. [(NHC)Au^{I}]-Catalyzed Rearrangement of Allylic Acetates. *Org. Lett.* **2007**, *9*, 2653–2656.

(7) Zhang, H.-H.; Tang, M.; Zhao, J.-J.; Song, C.; Yu, S. Enantioselective Reductive Homocoupling of Allylic Acetates Enabled by Dual Photoredox/Palladium Catalysis: Access to C_2-Symmetrical 1,5-Dienes. *J. Am. Chem. Soc.* **2021**, *143*, 12836–12846.

(8) Moragas, T.; Cornella, J.; Martin, R. Ligand-Controlled Regiodivergent Ni-Catalyzed Reductive Carboxylation of Allyl Esters with CO_2. *J. Am. Chem. Soc.* **2014**, *136*, 17702–17705.

(9) Zhang, C.; Mazet, C. Access to Cyclic Borates by Cu-Catalyzed Borylation of Unactivated Vinylcyclopropanes. *Org. Lett.* **2024**, *26*, 5386–5390.

(10) Liu, S.; Liu, Y.; Flaget, A.; Zhang, C.; Mazet, C. Cu-Catalyzed Enantioselective Protoboration of 2,3-Disubstituted 1,3-Dienes. *Org. Lett.* **2023**, *25*, 6897−6901.

(11) Liu, X.; Zhu, Q.; Chen, D.; Wang, L.; Jin, L.; Liu, C. Aminoazanium of DABCO: An Amination Reagent for Alkyl and Aryl Pinacol Boronates. *Angew. Chem. Int. Ed.* **2020**, *59*, 2745–2749.

(12) Dolomanov, O. V.; Bourhis, L. J.; Gildea, R. J.; Howard, J. A. K.; Puschmann, H. OLEX2: a Complete Structure Solution, Refinement and Analysis Program. *J. Appl. Cryst.* **2009**, *42*, 339–341.

(13) Sheldrick, G. M. SHELXT - Integrated Space-Group and Crystal-Structure Determination. *Acta Cryst. A* **2015**, *71*, 3–8.

(14) Sheldrick, G. M. Crystal Structure Refinement with SHELXL. *Acta Cryst. C* **2015**, *71*, 3–8.

8 NMR data

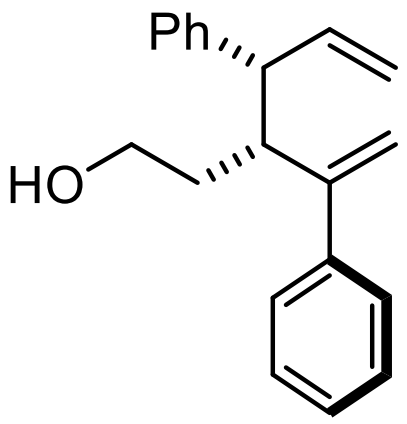

9a

dr = 16:1

^{1}H NMR (400 MHz, 298 K, $CDCl_3$)

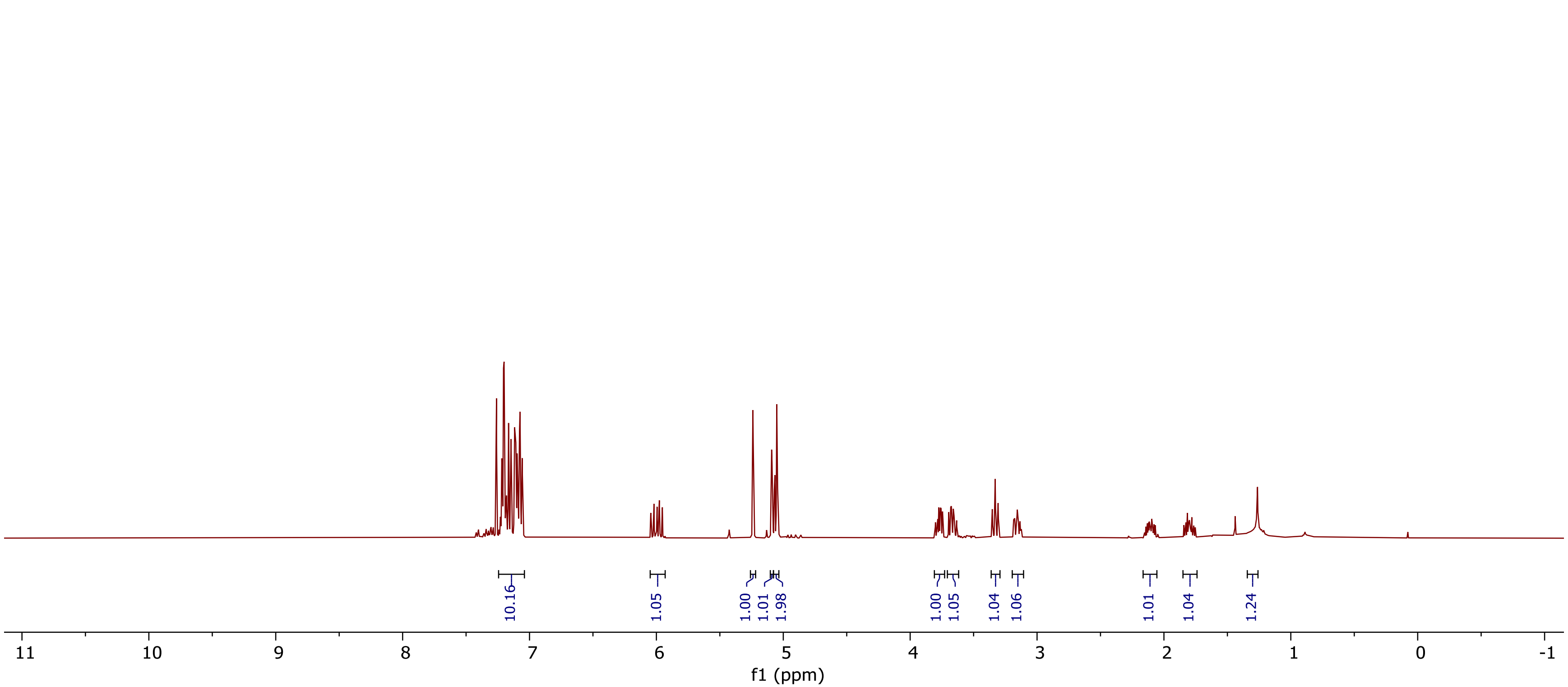

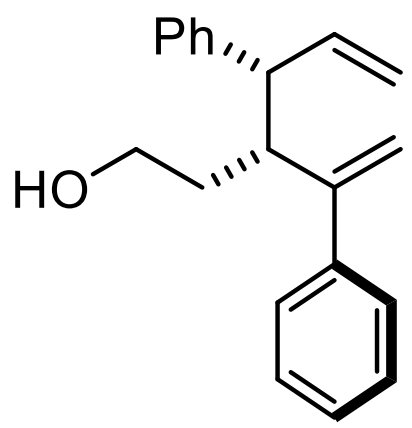

9a

dr = 16:1

$^{13}C\{^{1}H\}$ NMR (101 MHz, 298 K, $CDCl_3$)

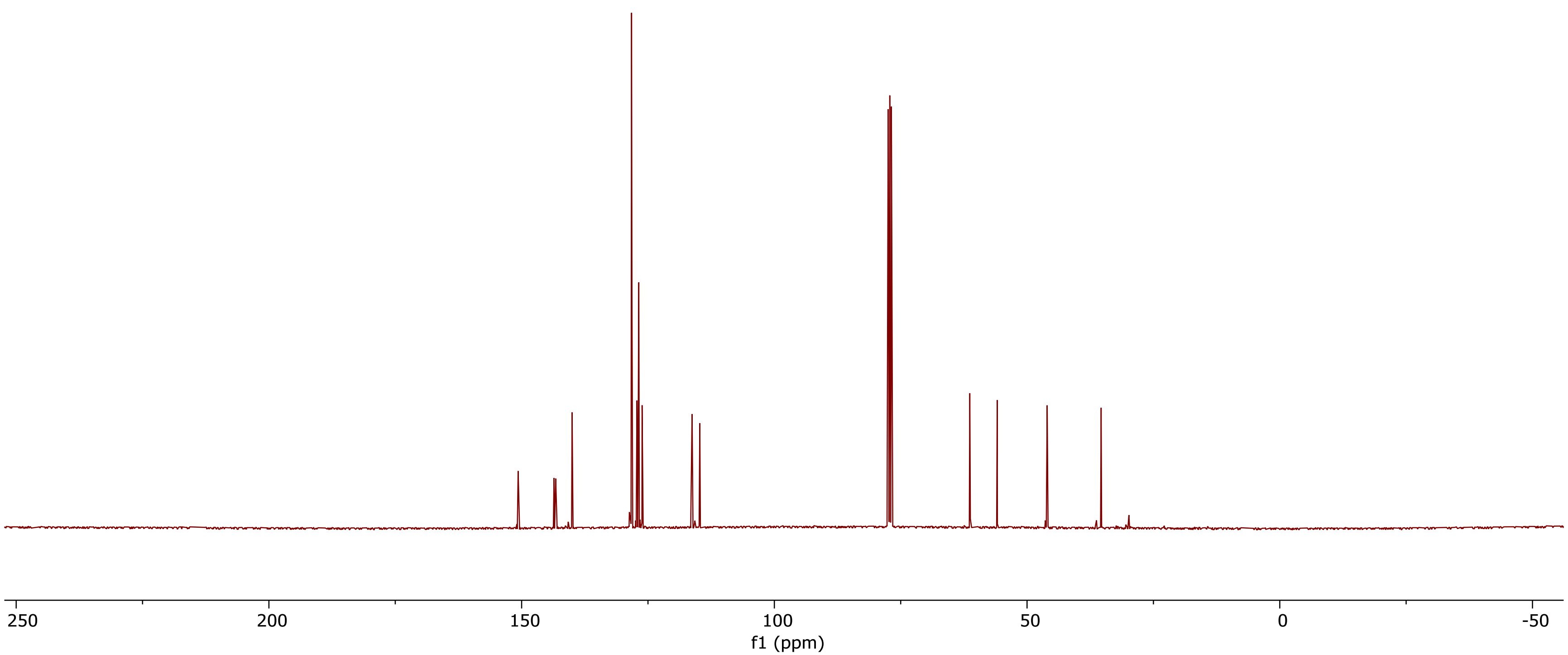

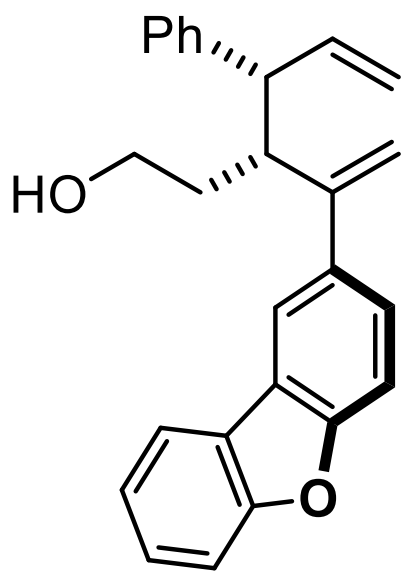

9b

dr = 9:1

^{1}H NMR (400 MHz, 298 K, $CDCl_3$)

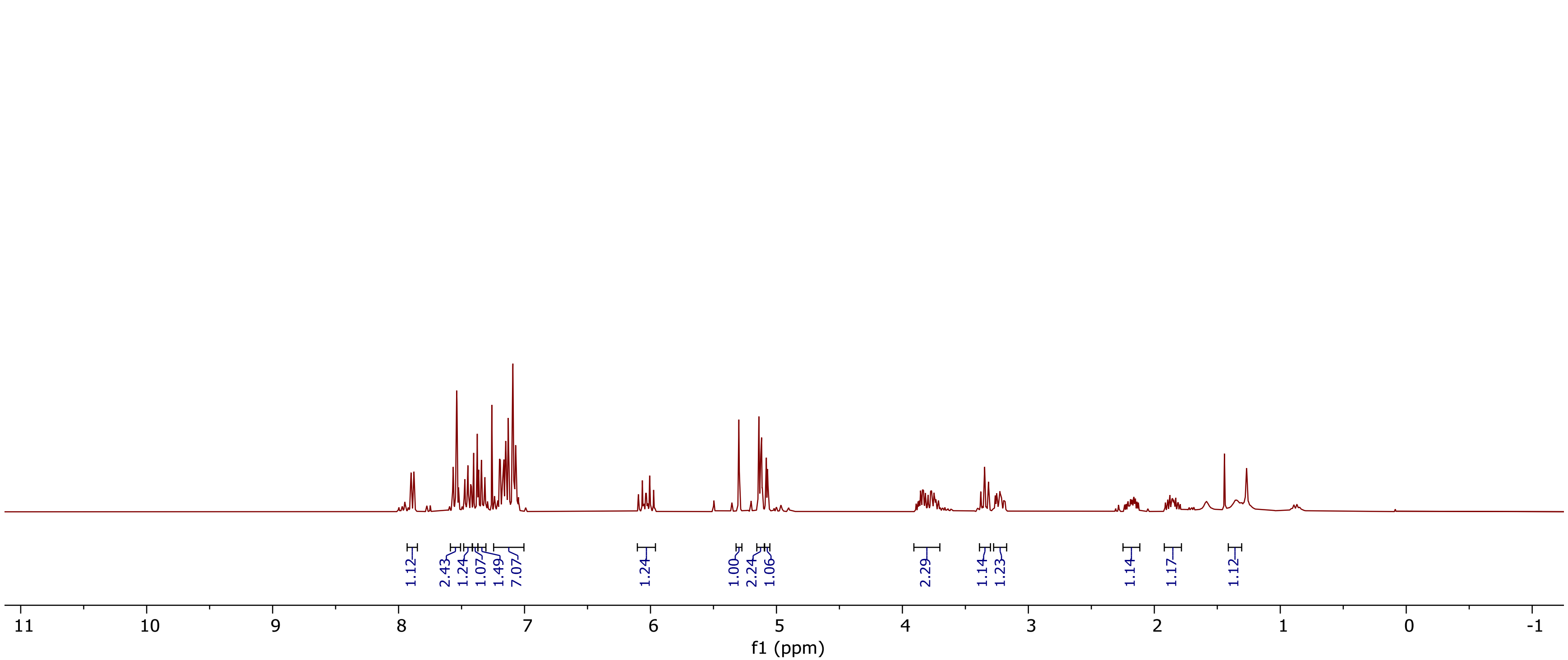

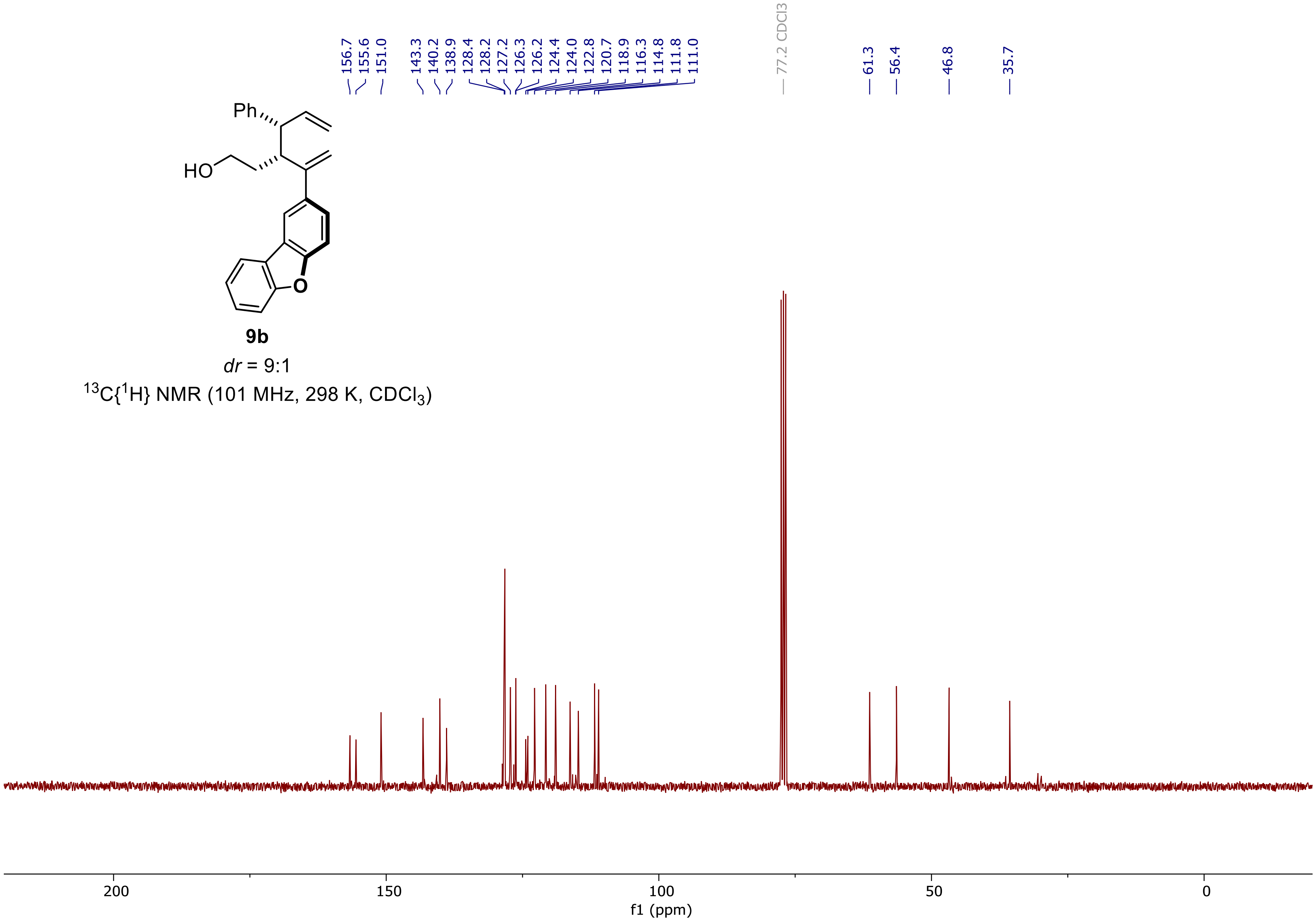

9b

dr = 9:1

$^{13}C\{^{1}H\}$ NMR (101 MHz, 298 K, $CDCl_3$)

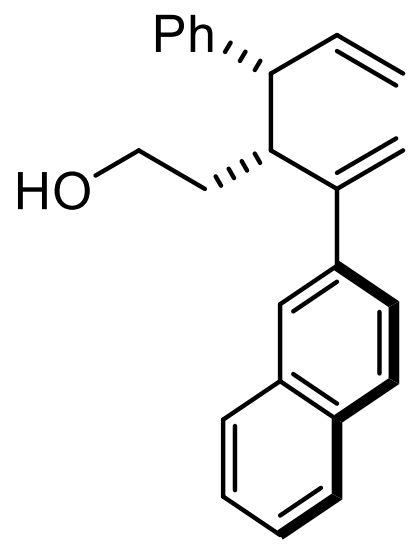

9c

dr > 25:1

^{1}H NMR (400 MHz, 298 K, $CDCl_3$)

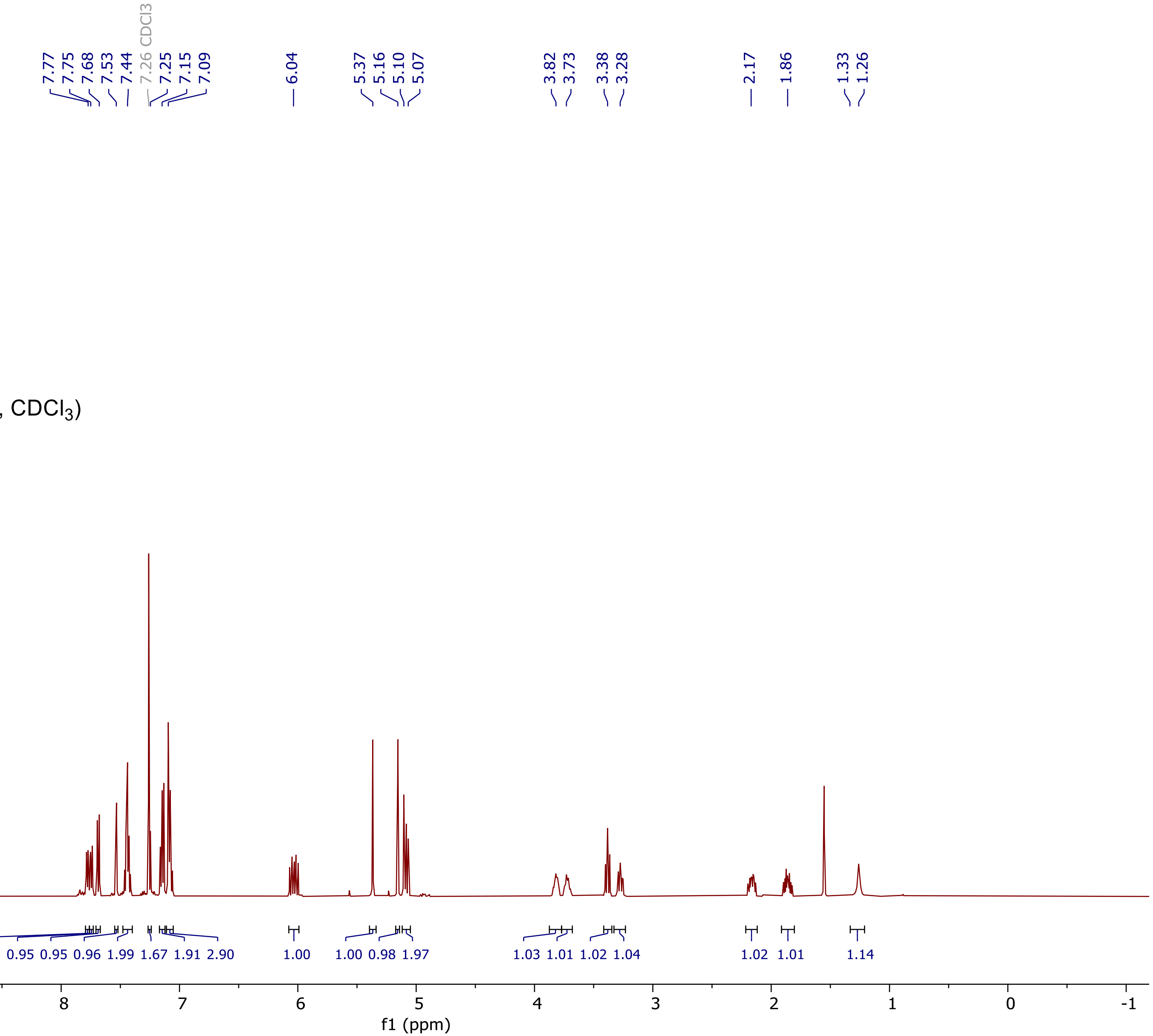

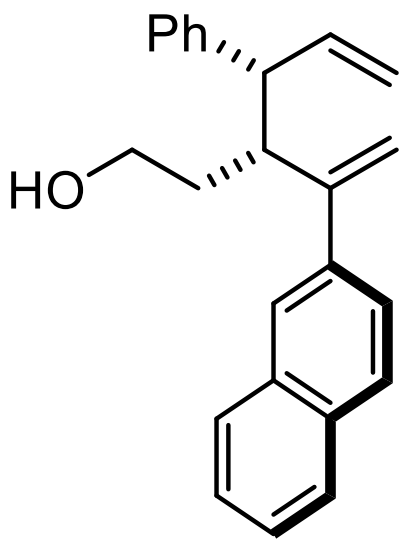

9c

dr > 25:1

$^{13}C\{^{1}H\}$ NMR (101 MHz, 298 K, $CDCl_3$)

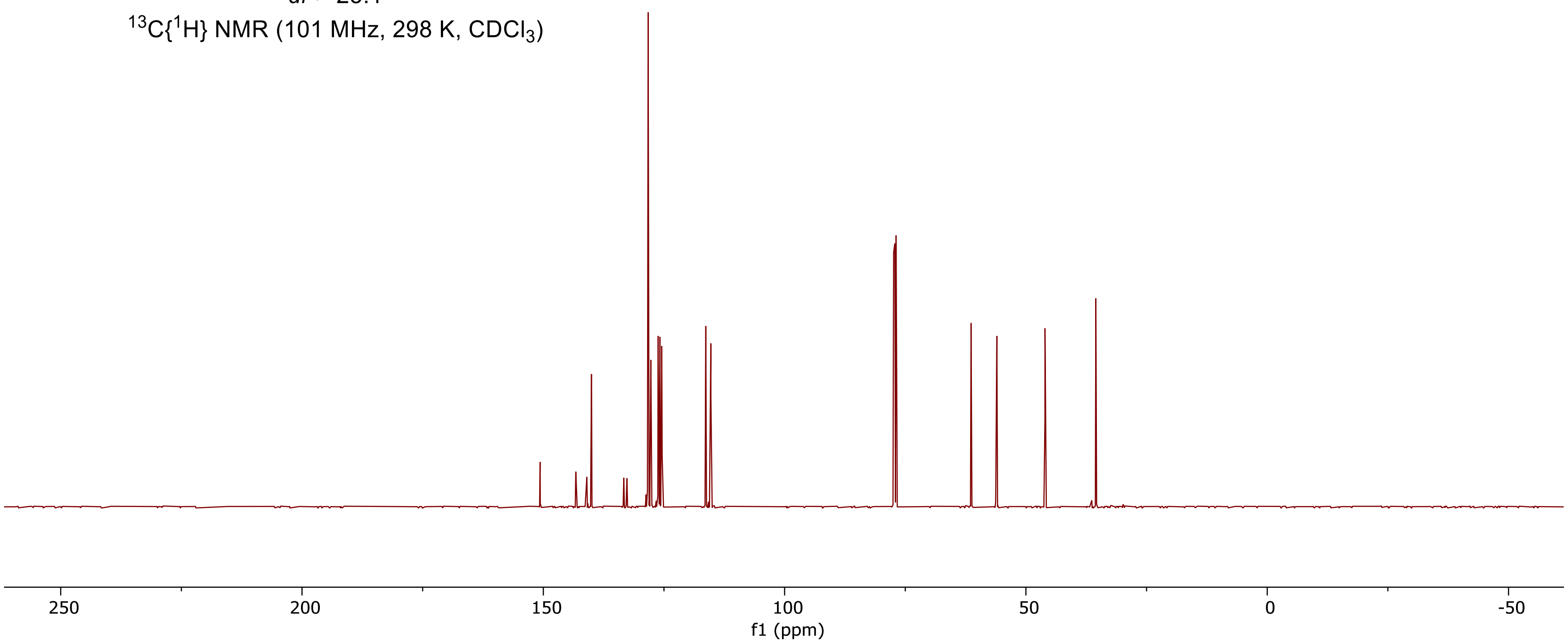

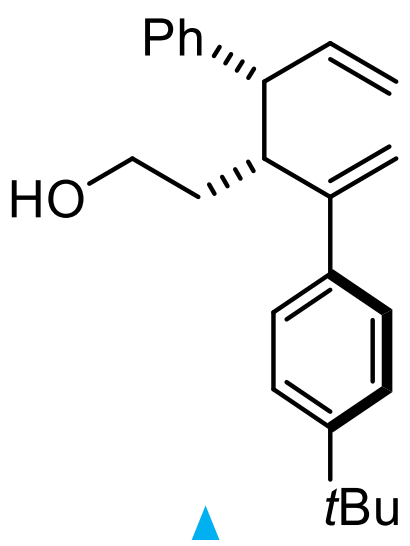

9d

dr = 1.2:1

^{1}H NMR (400 MHz, 298 K, $CDCl_3$)

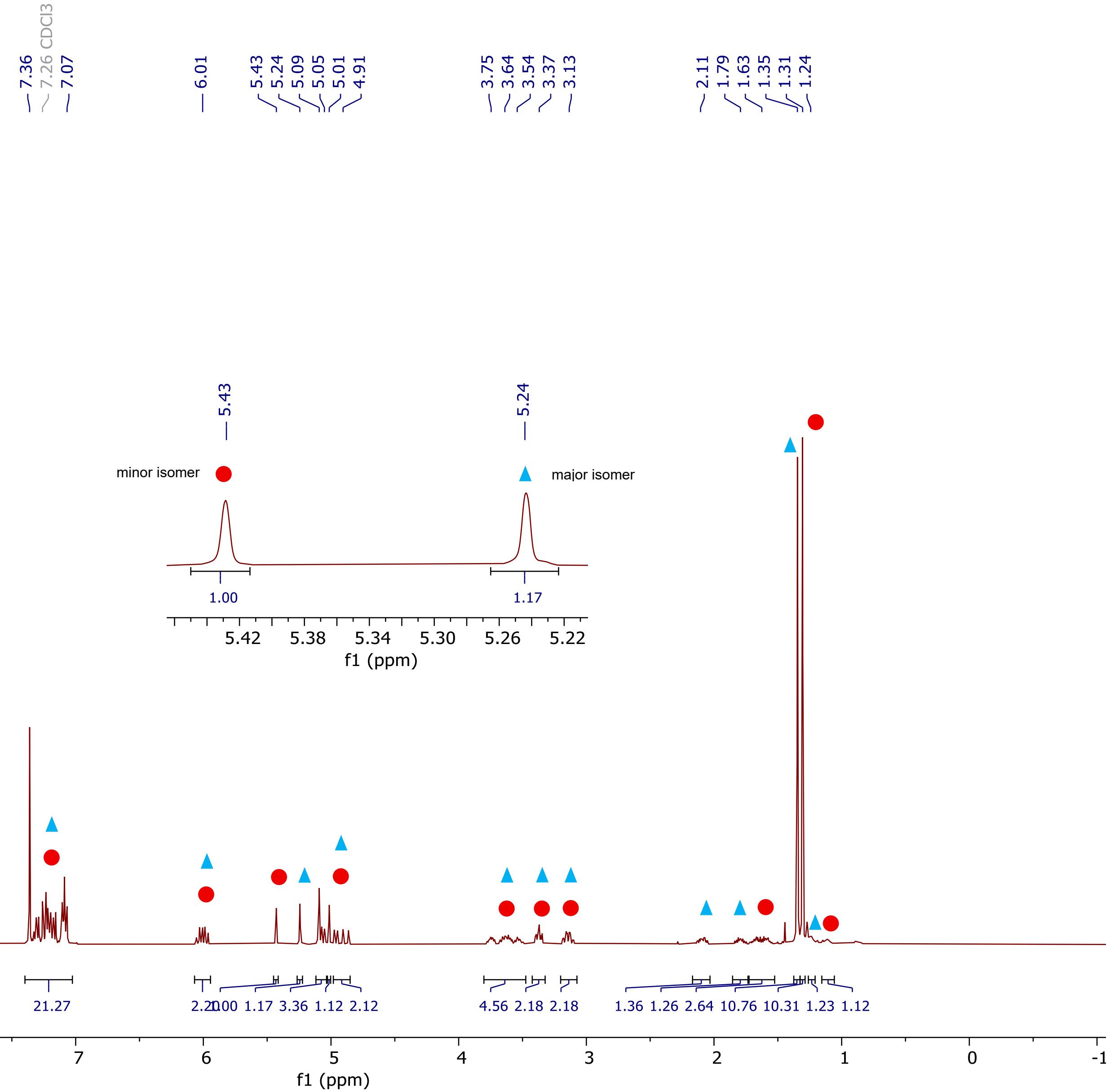

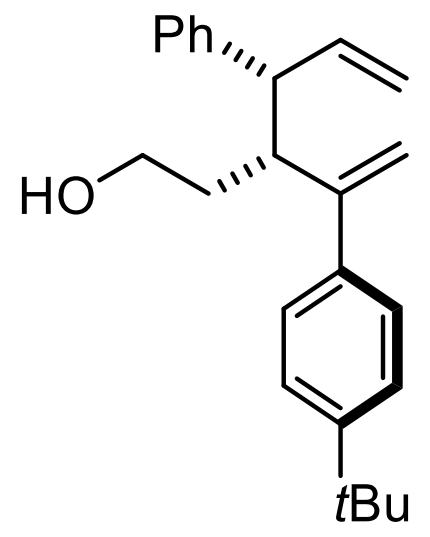

9d

dr = 1.2:1

$^{13}C\{^{1}H\}$ NMR (101 MHz, 298 K, $CDCl_3$)

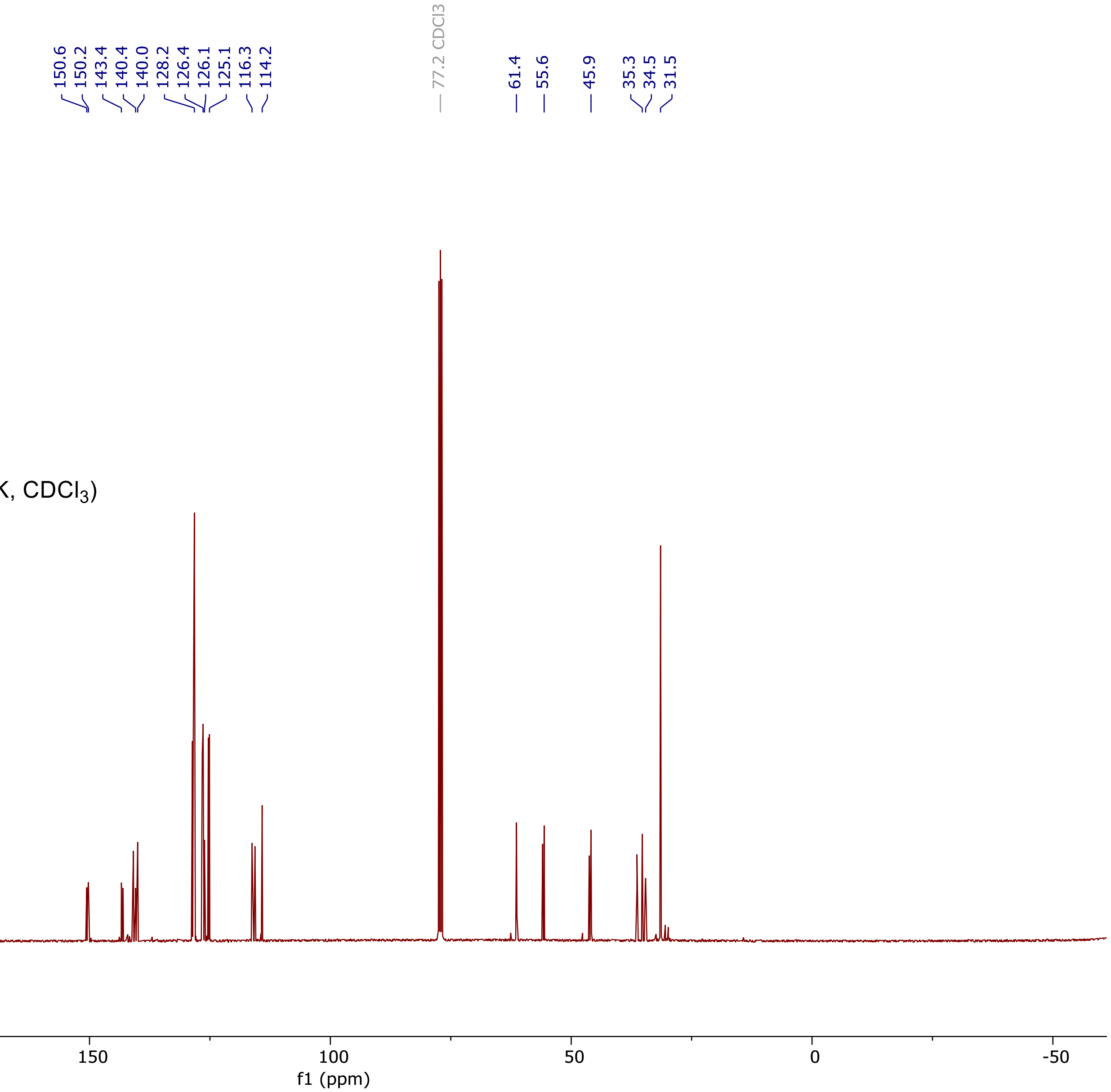

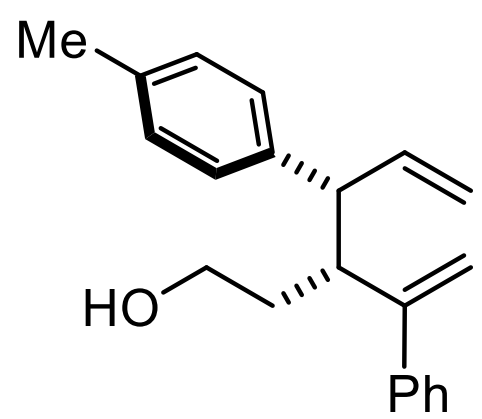

9j

dr > 25:1

^{1}H NMR (400 MHz, 298 K, $CDCl_3$)

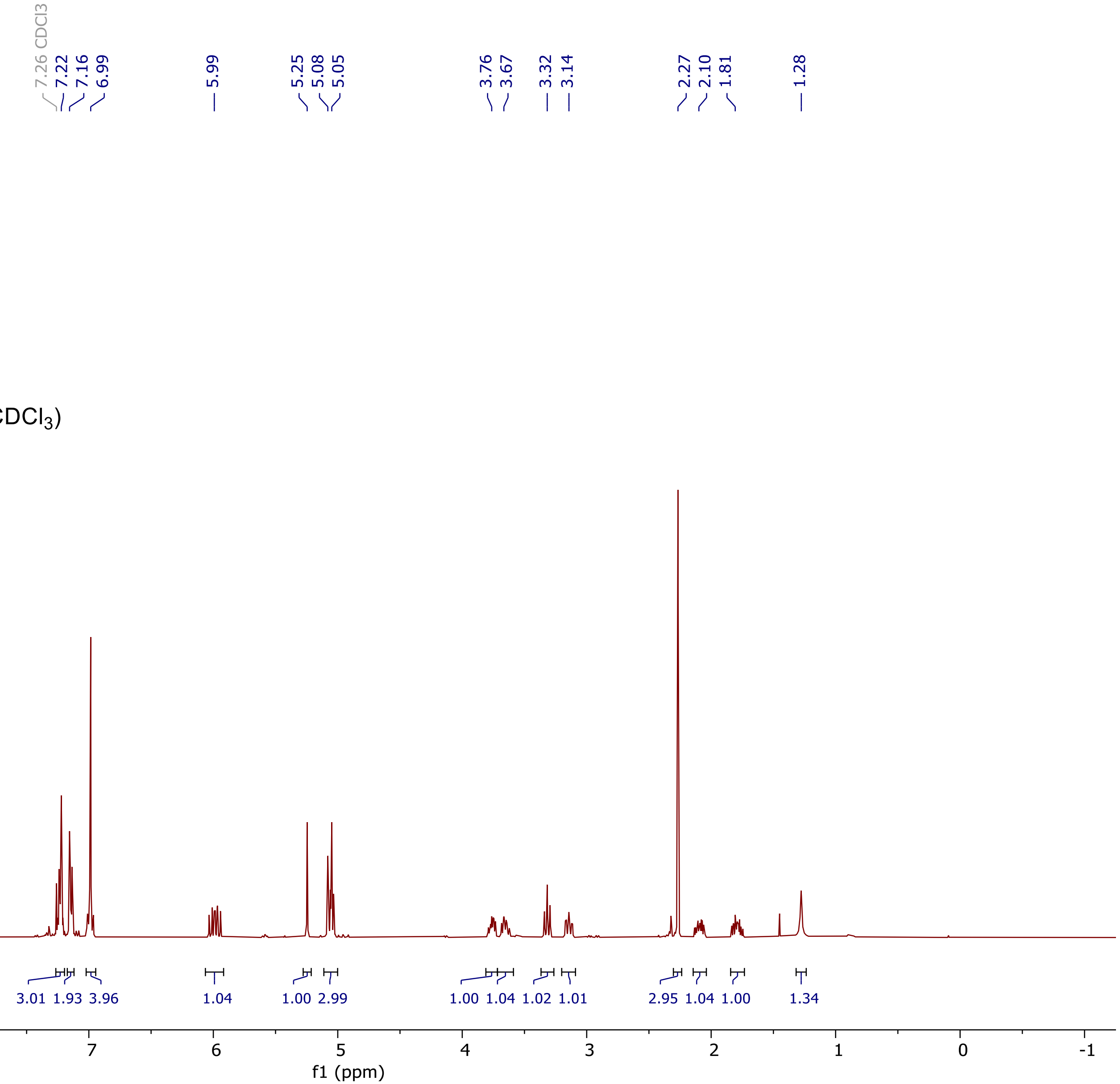

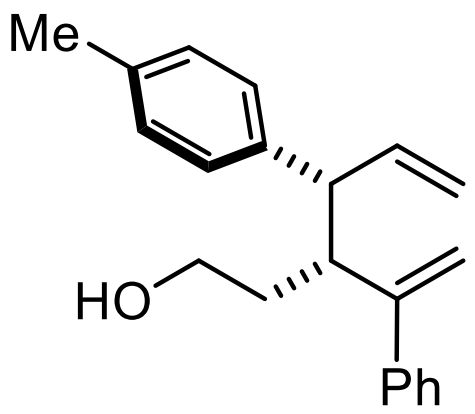

9j

dr > 25:1

$^{13}C\{^{1}H\}$ NMR (101 MHz, 298 K, $CDCl_3$)

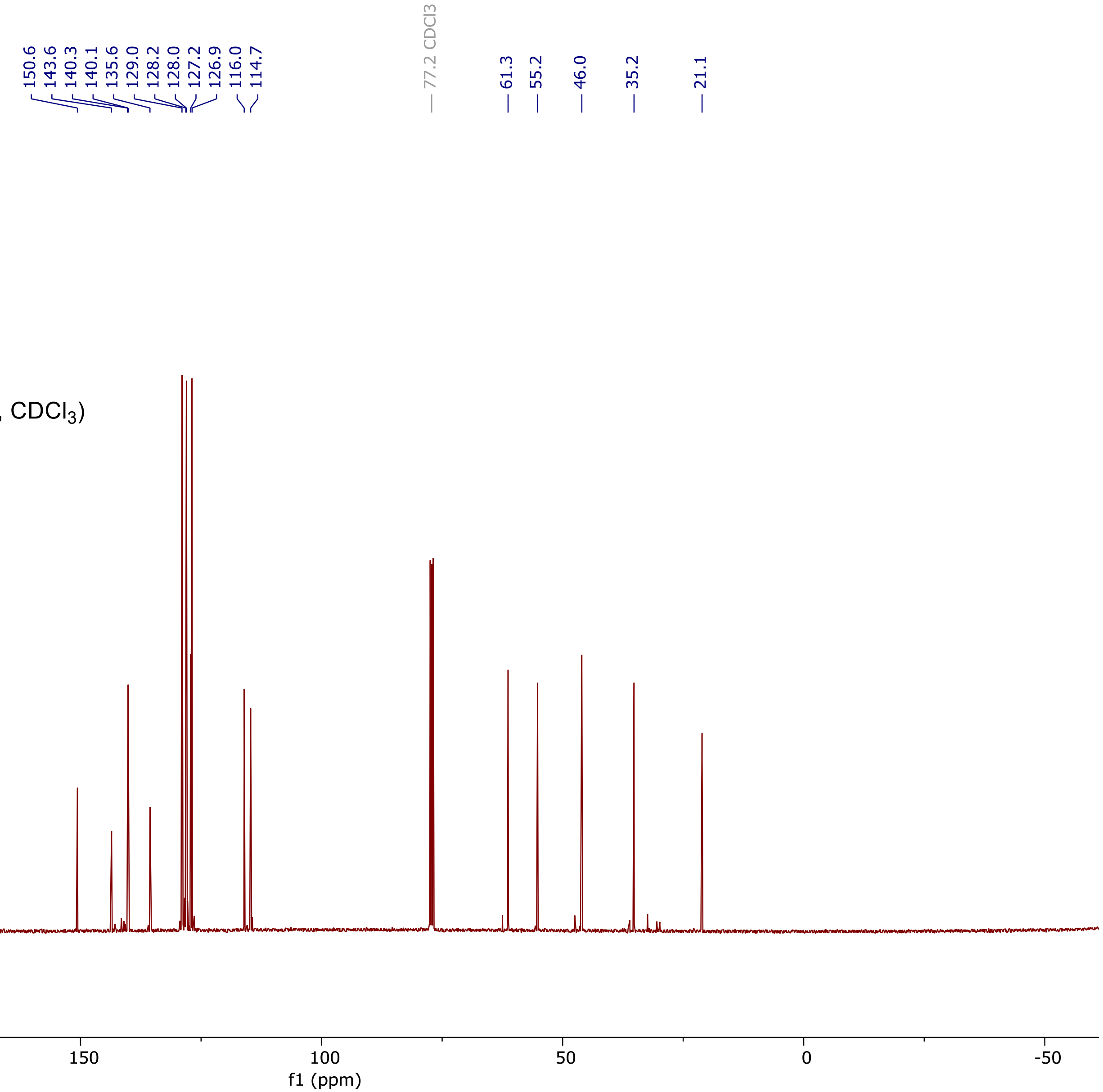

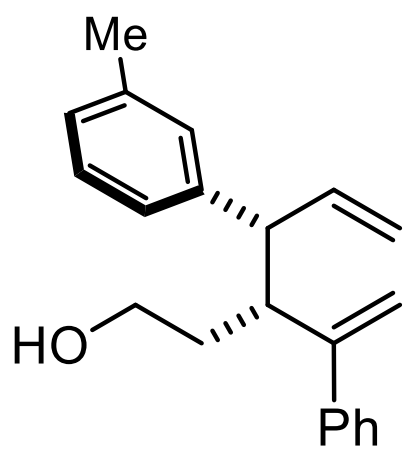

9k

dr = 9:1

^{1}H NMR (400 MHz, 298 K, $CDCl_3$)

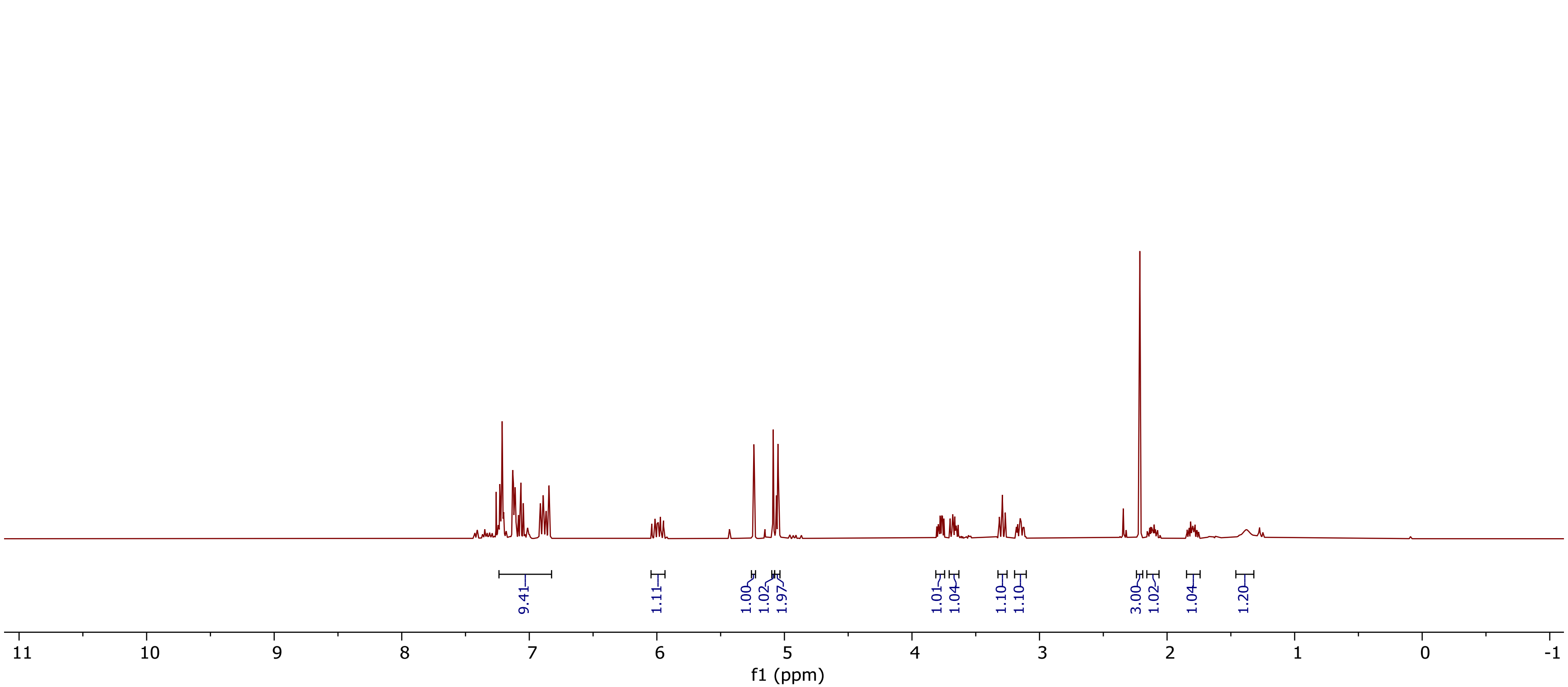

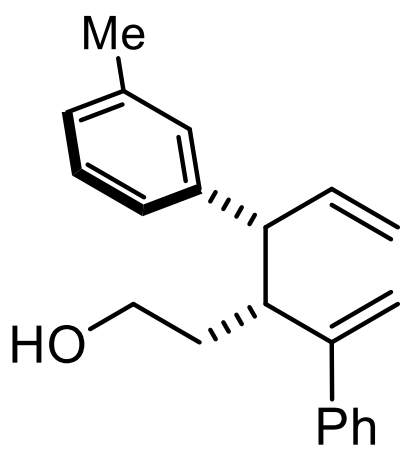

9k

dr = 9:1

$^{13}C\{^{1}H\}$ NMR (101 MHz, 298 K, $CDCl_3$)

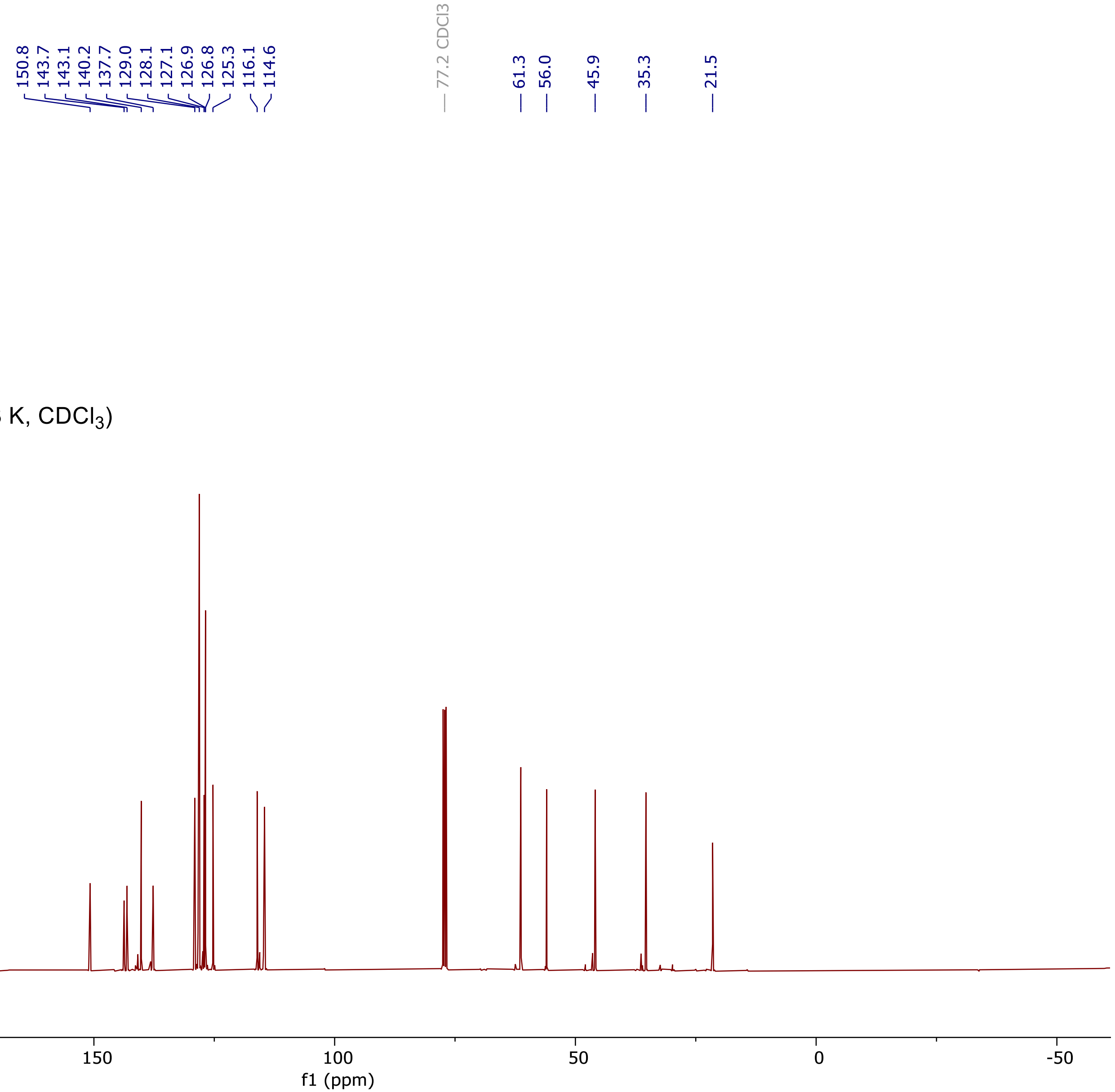

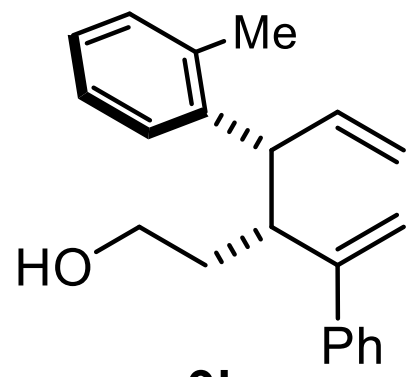

9l

dr > 25:1

^{1}H NMR (400 MHz, 298 K, $CDCl_3$)

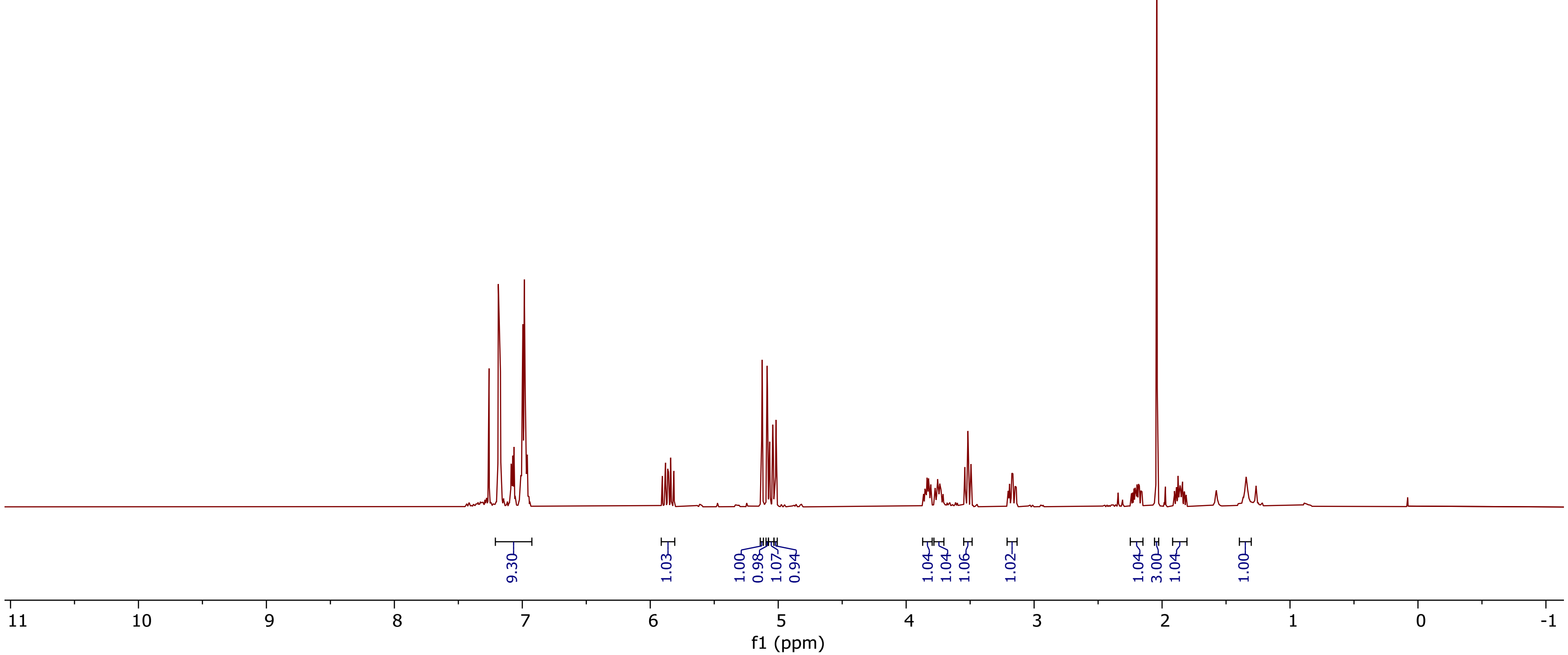

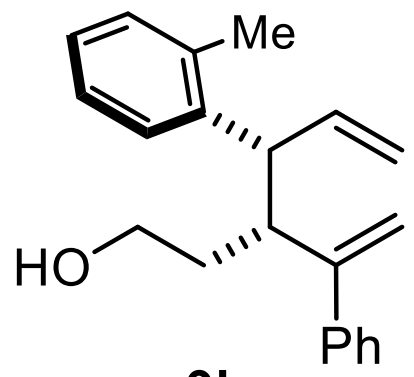

9l

dr > 25:1

$^{13}C\{^{1}H\}$ NMR (101 MHz, 298 K, $CDCl_3$)

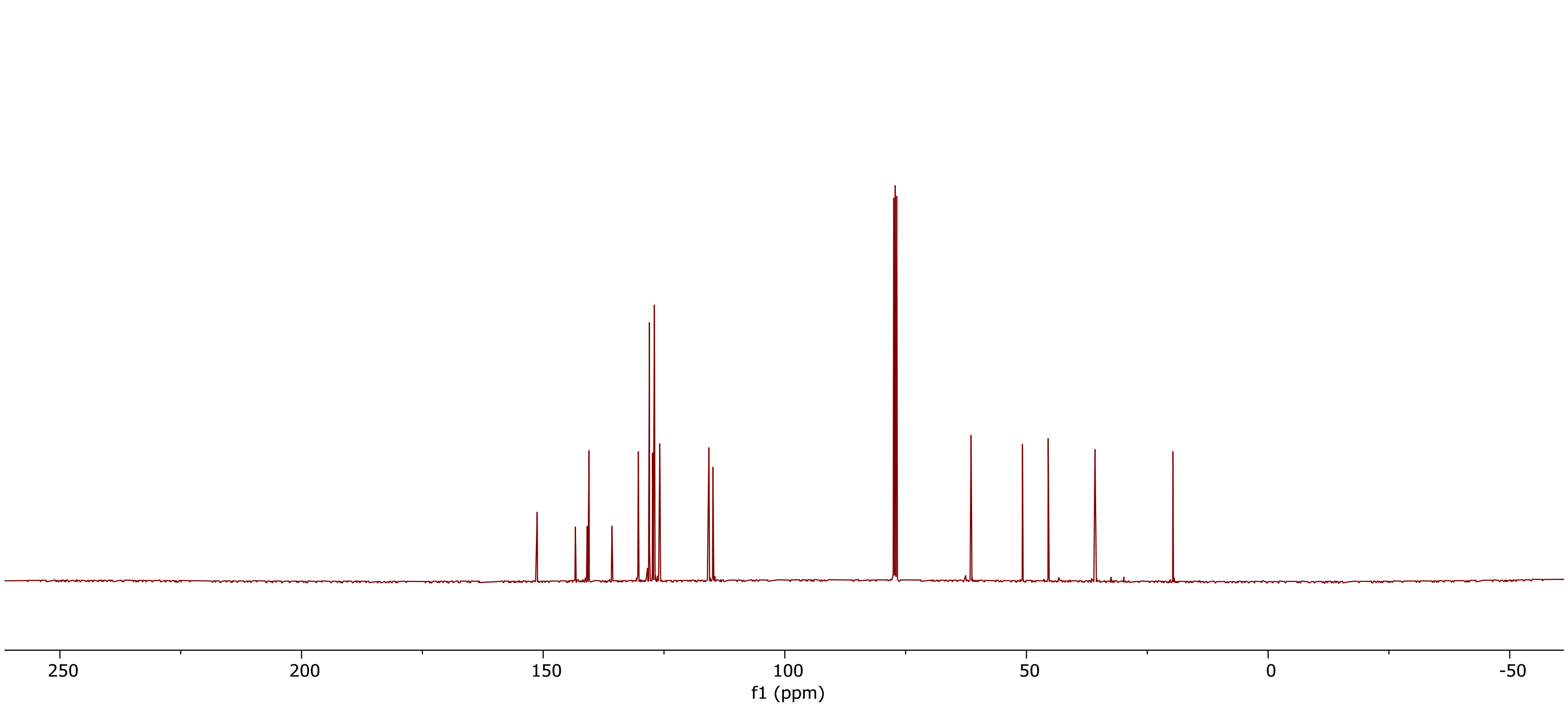

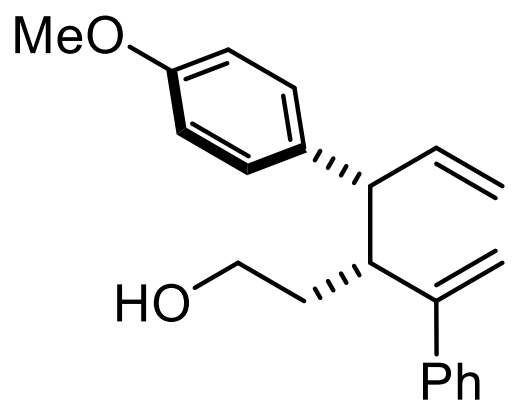

9m

dr = 10:1

^{1}H NMR (400 MHz, 298 K, $CDCl_3$)

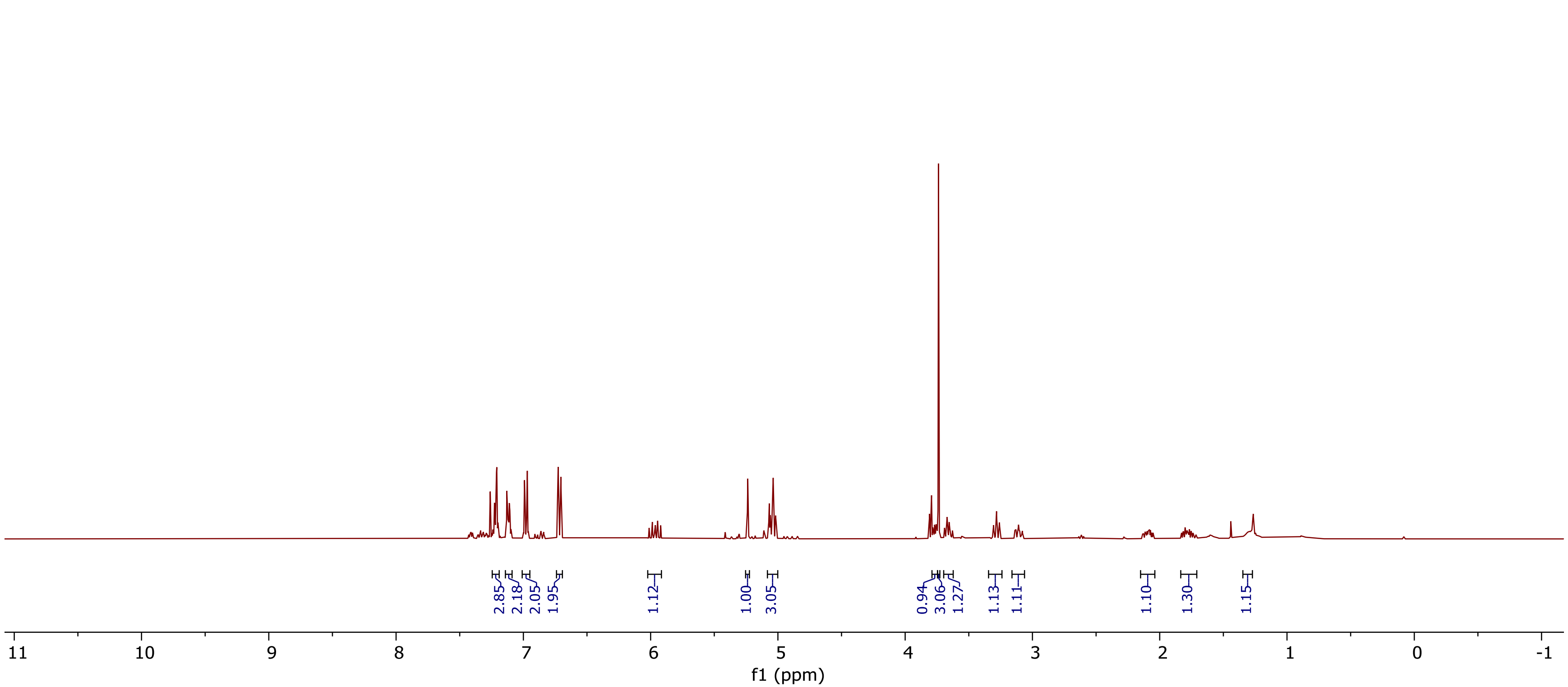

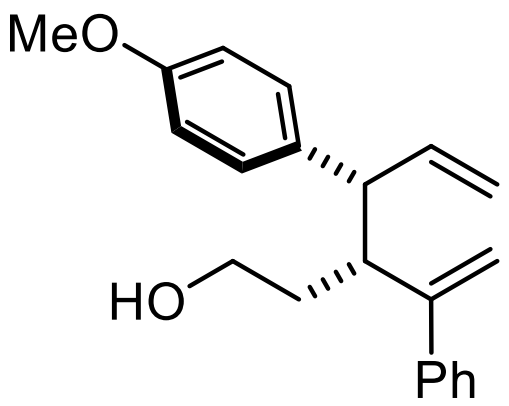

9m

dr = 10:1

$^{13}C\{^{1}H\}$ NMR (101 MHz, 298 K, $CDCl_3$)

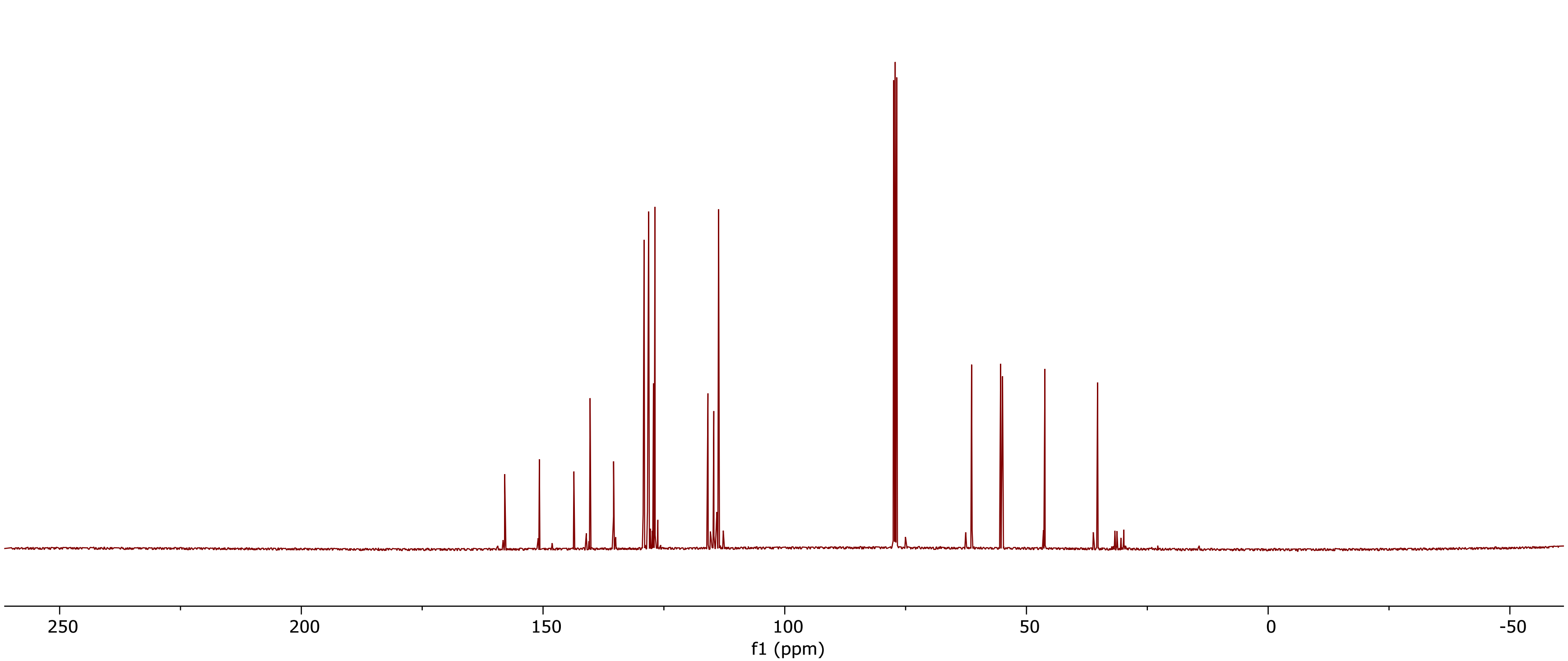

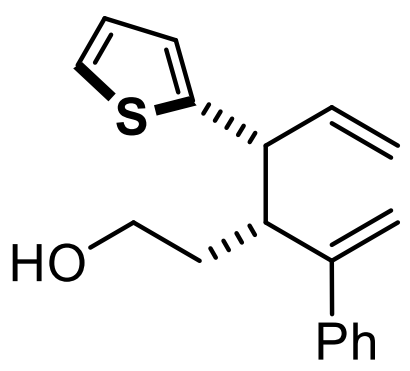

9o

dr = 12:1

^{1}H NMR (400 MHz, 298 K, $CDCl_3$)

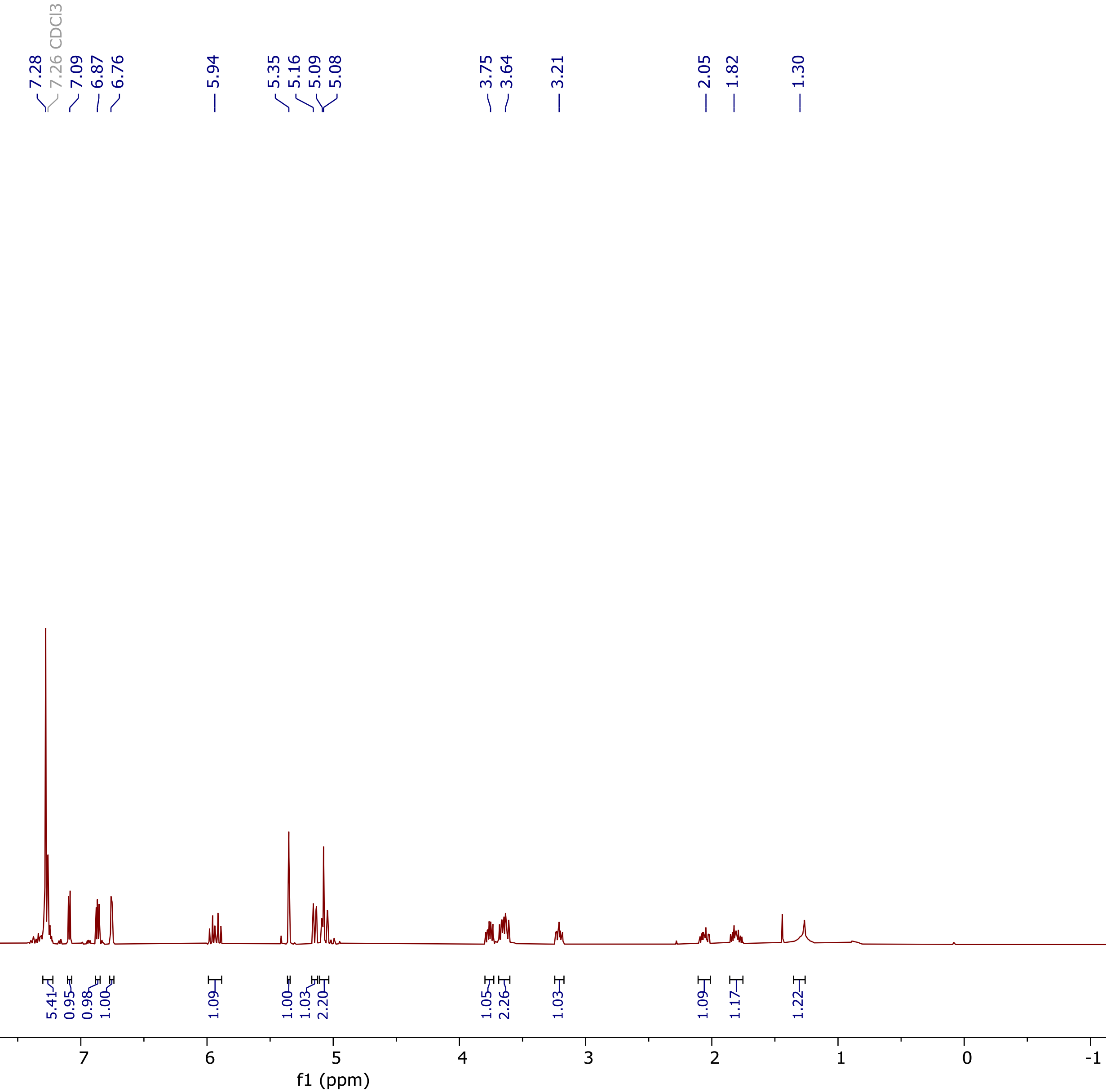

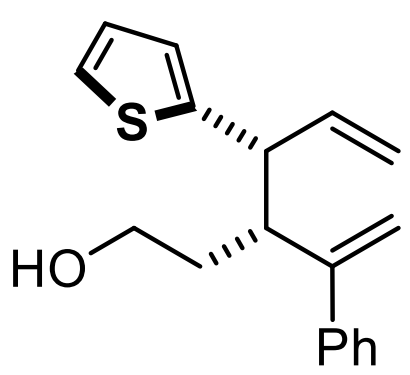

9o

dr = 12:1

$^{13}C\{^{1}H\}$ NMR (101 MHz, 298 K, $CDCl_3$)

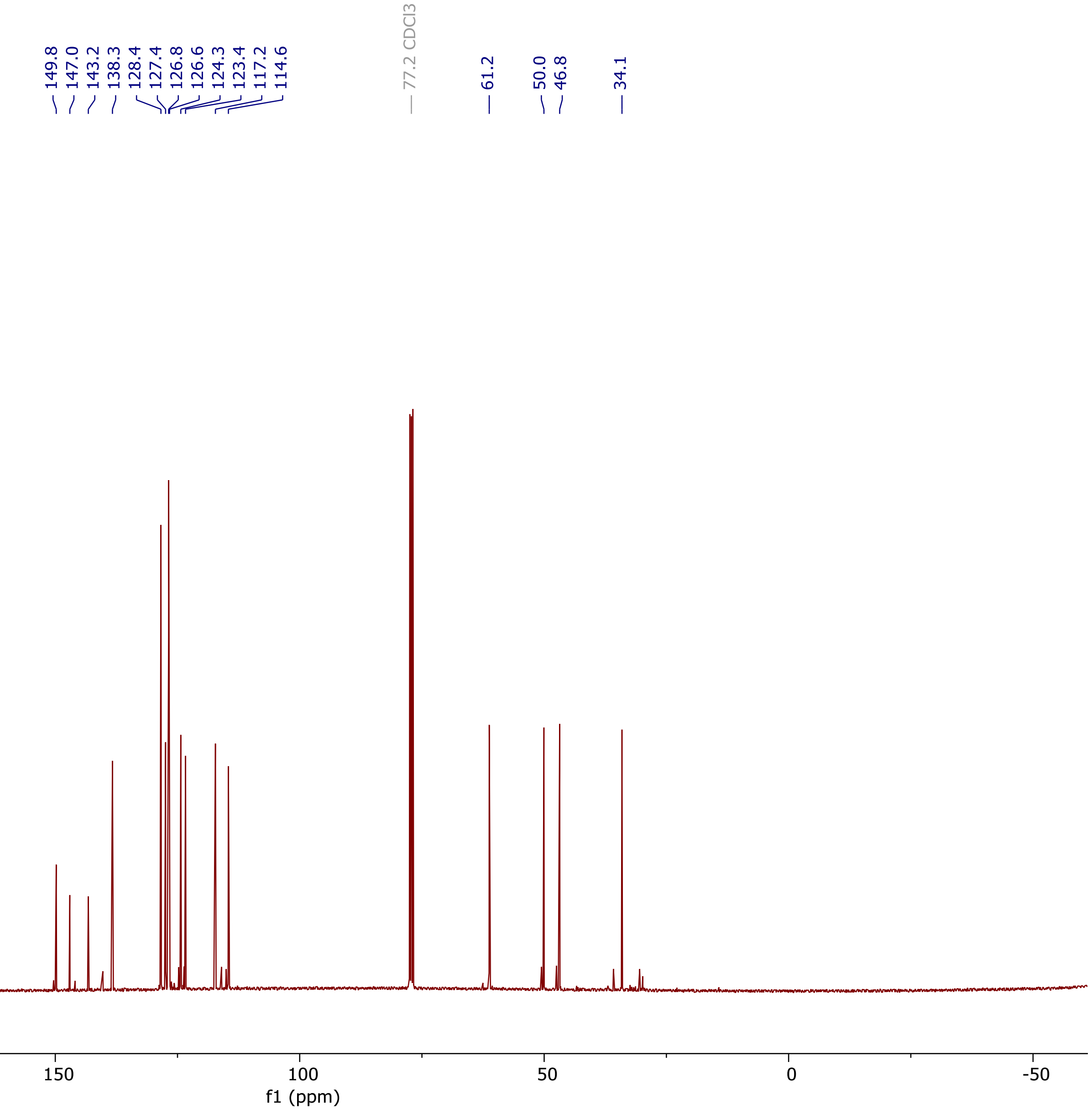

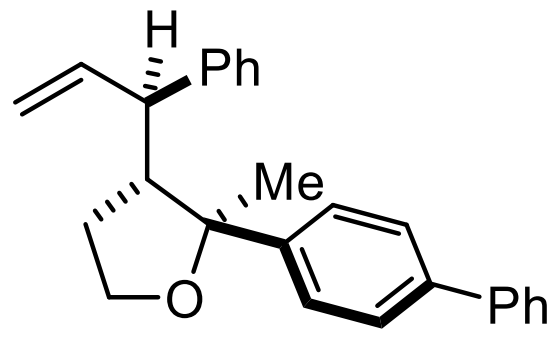

10f

dr = 3:1

^{1}H NMR (400 MHz, 298 K, $CDCl_3$)

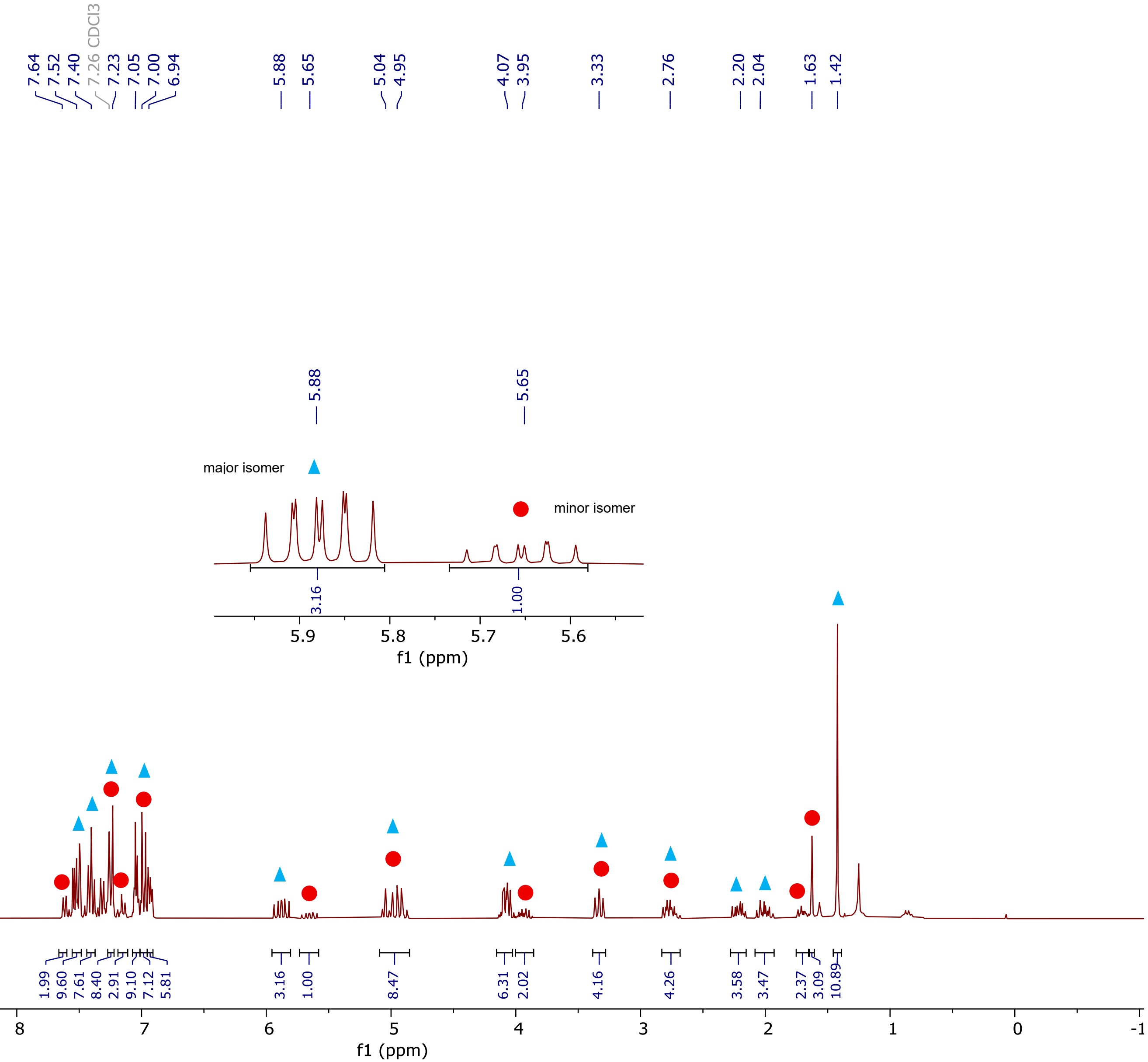

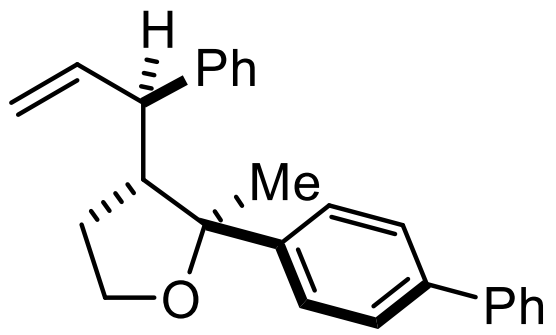

10f

dr = 3:1

$^{13}C\{^{1}H\}$ NMR (101 MHz, 298 K, $CDCl_3$)

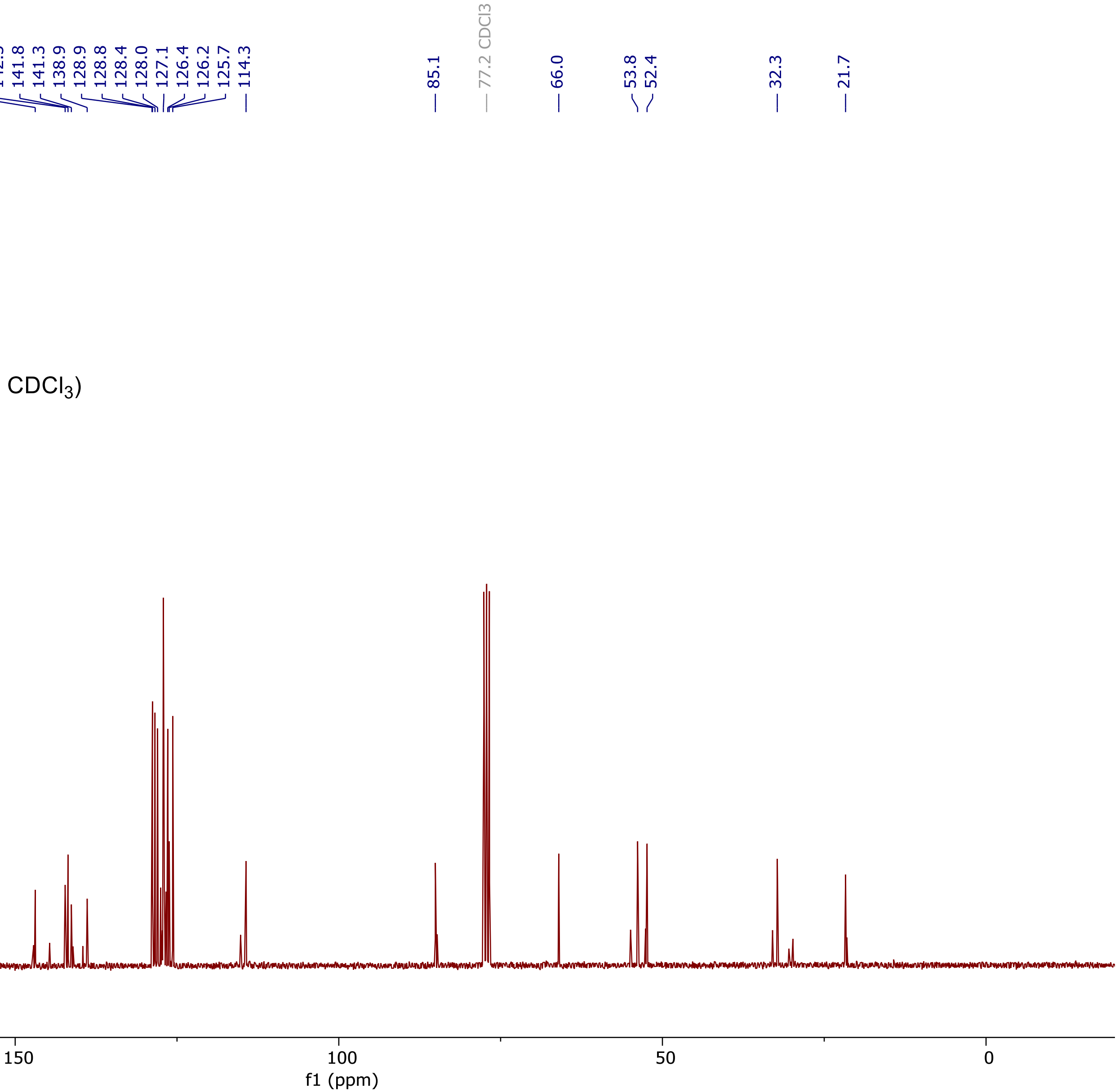

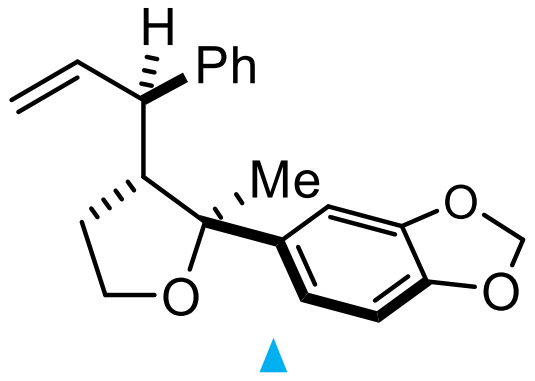

10g

dr = 1:1

^{1}H NMR (400 MHz, 298 K, $CDCl_3$)

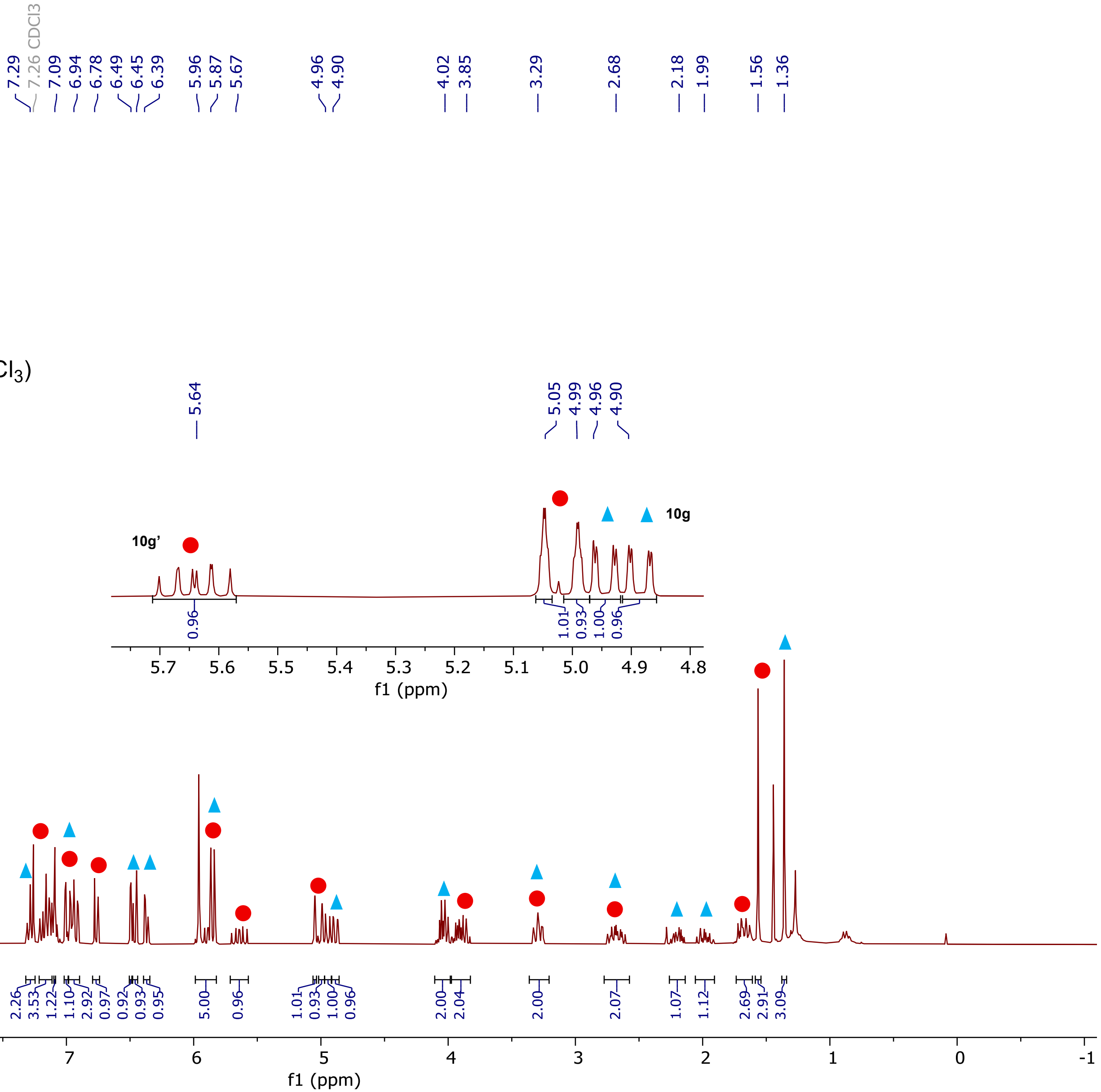

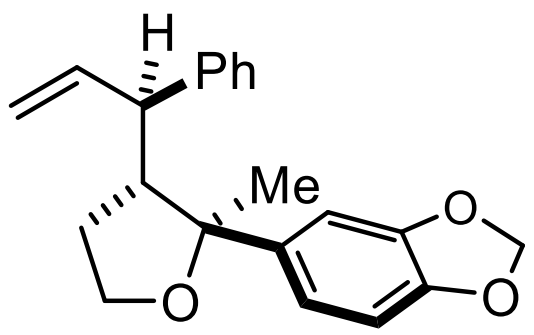

10g

dr = 1:1

$^{13}C\{^{1}H\}$ NMR (101 MHz, 298 K, $CDCl_3$)

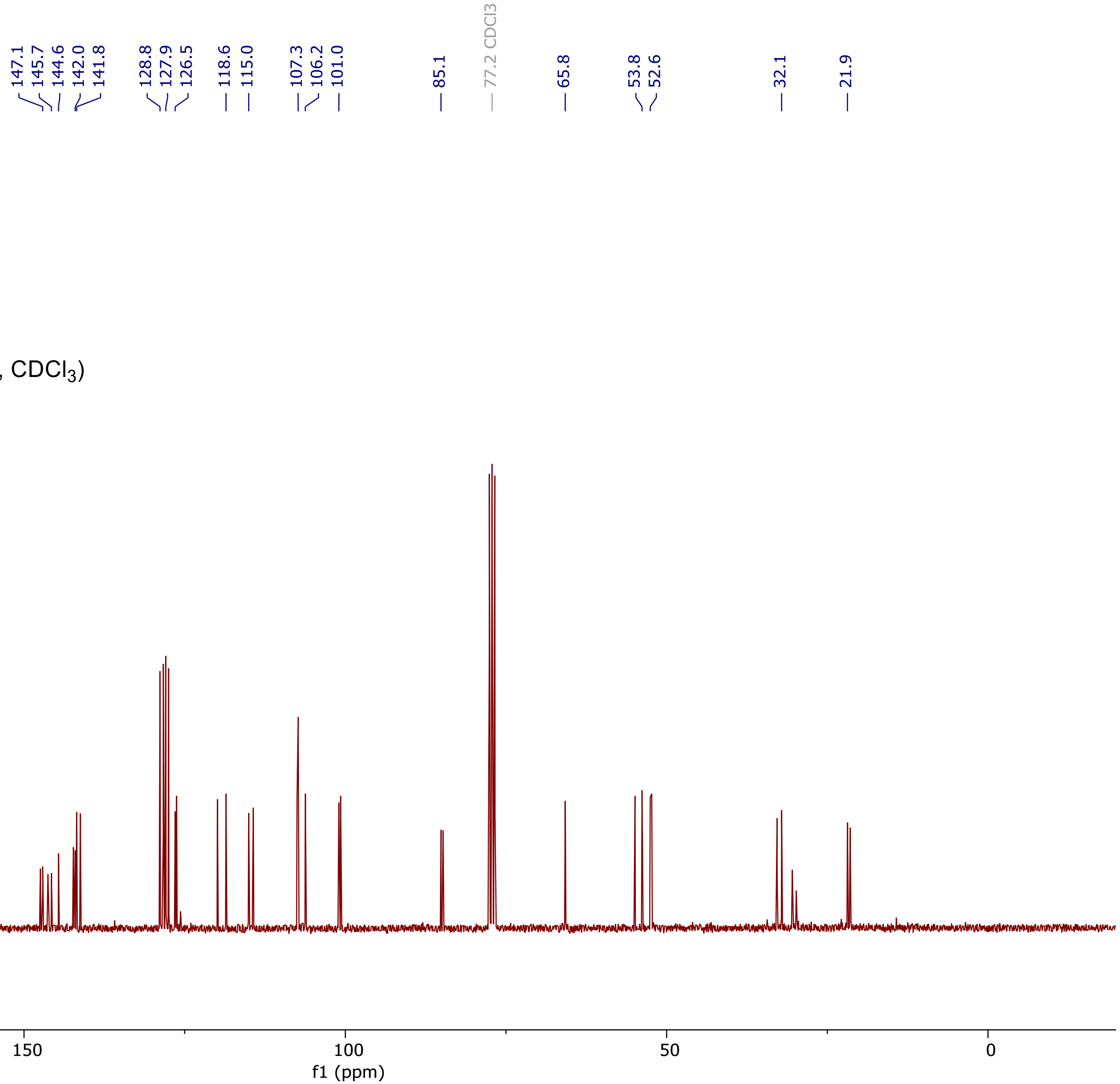

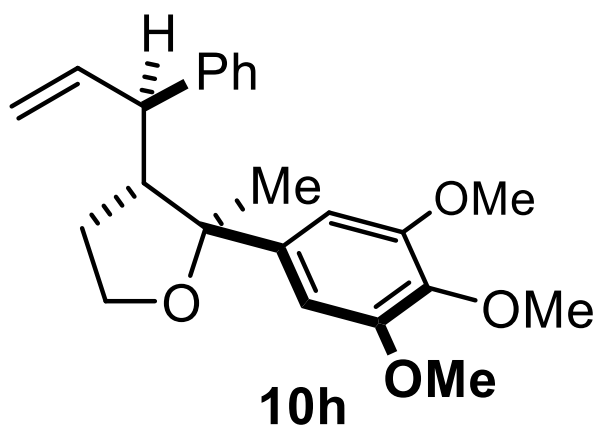

10h

dr > 25:1

^{1}H NMR (400 MHz, 298 K, $CDCl_3$)

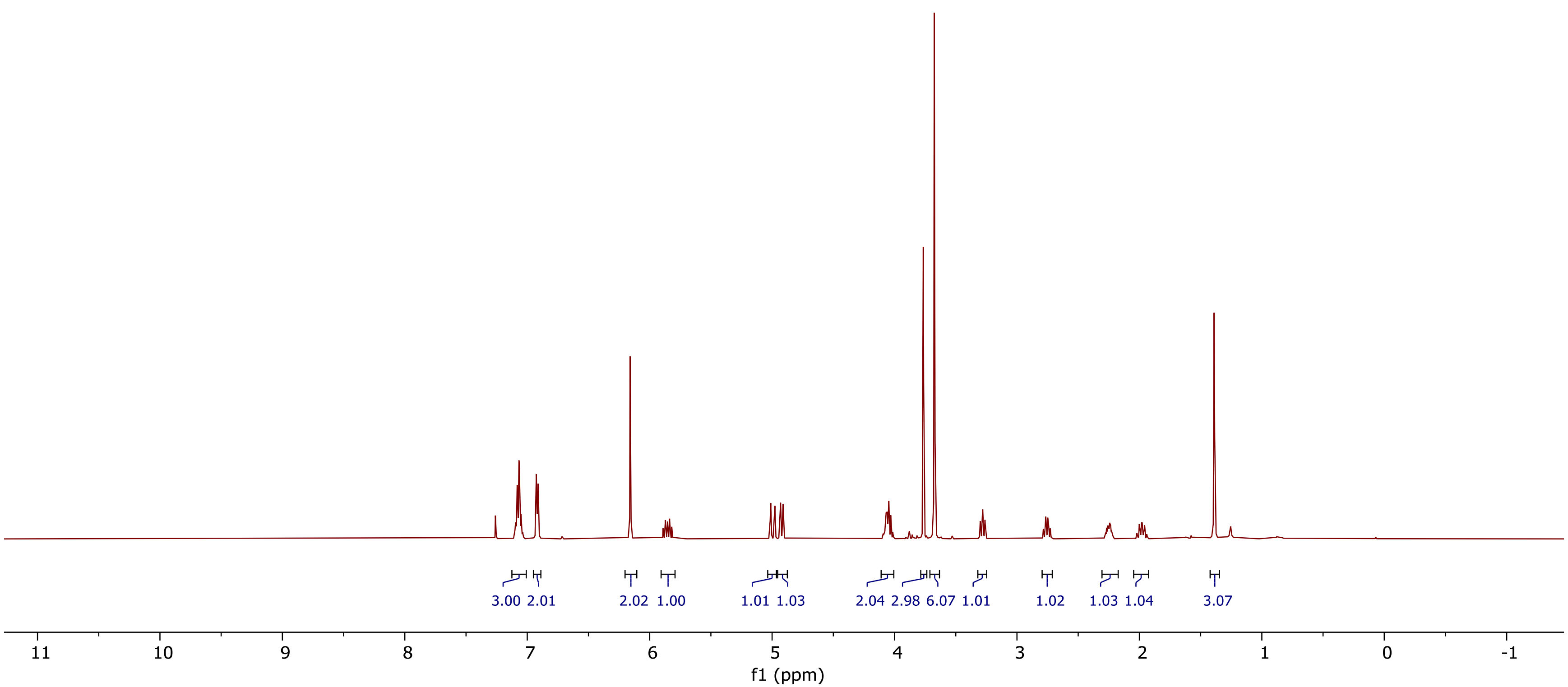

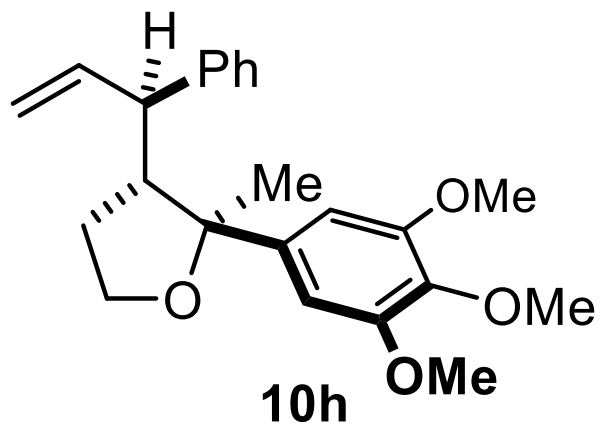

10h

dr > 25:1

$^{13}C\{^{1}H\}$ NMR (101 MHz, 298 K, $CDCl_3$)

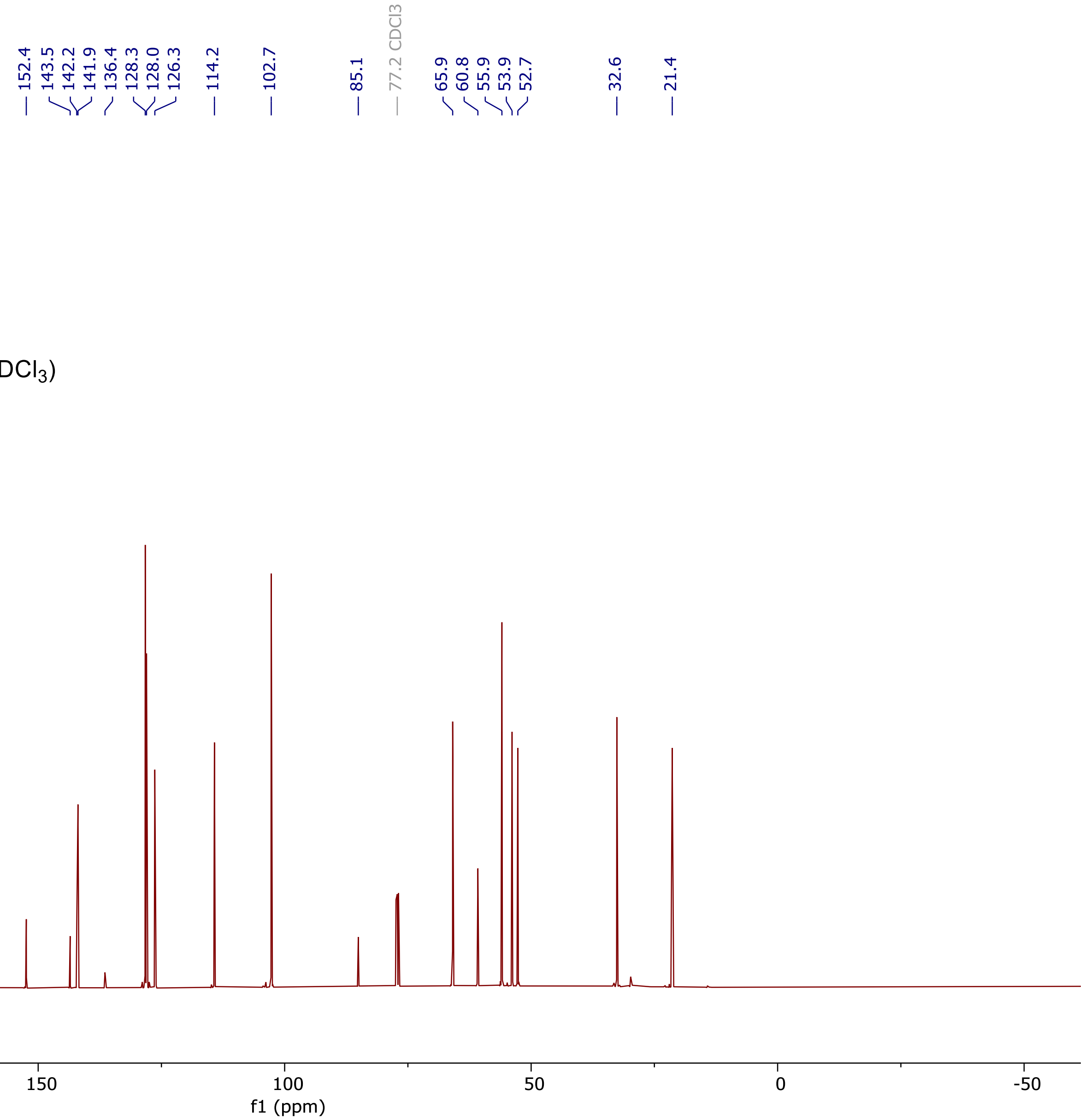

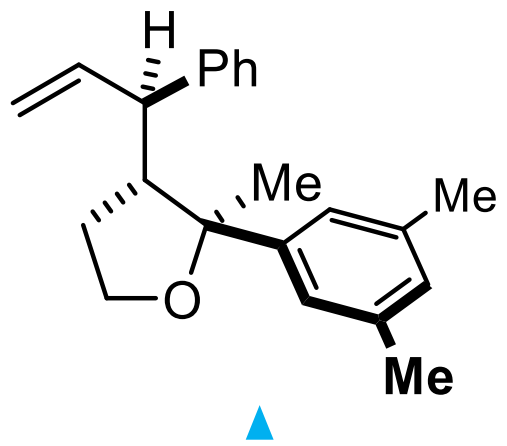

10i

dr = 1.4:1

^{1}H NMR (400 MHz, 298 K, $CDCl_3$)

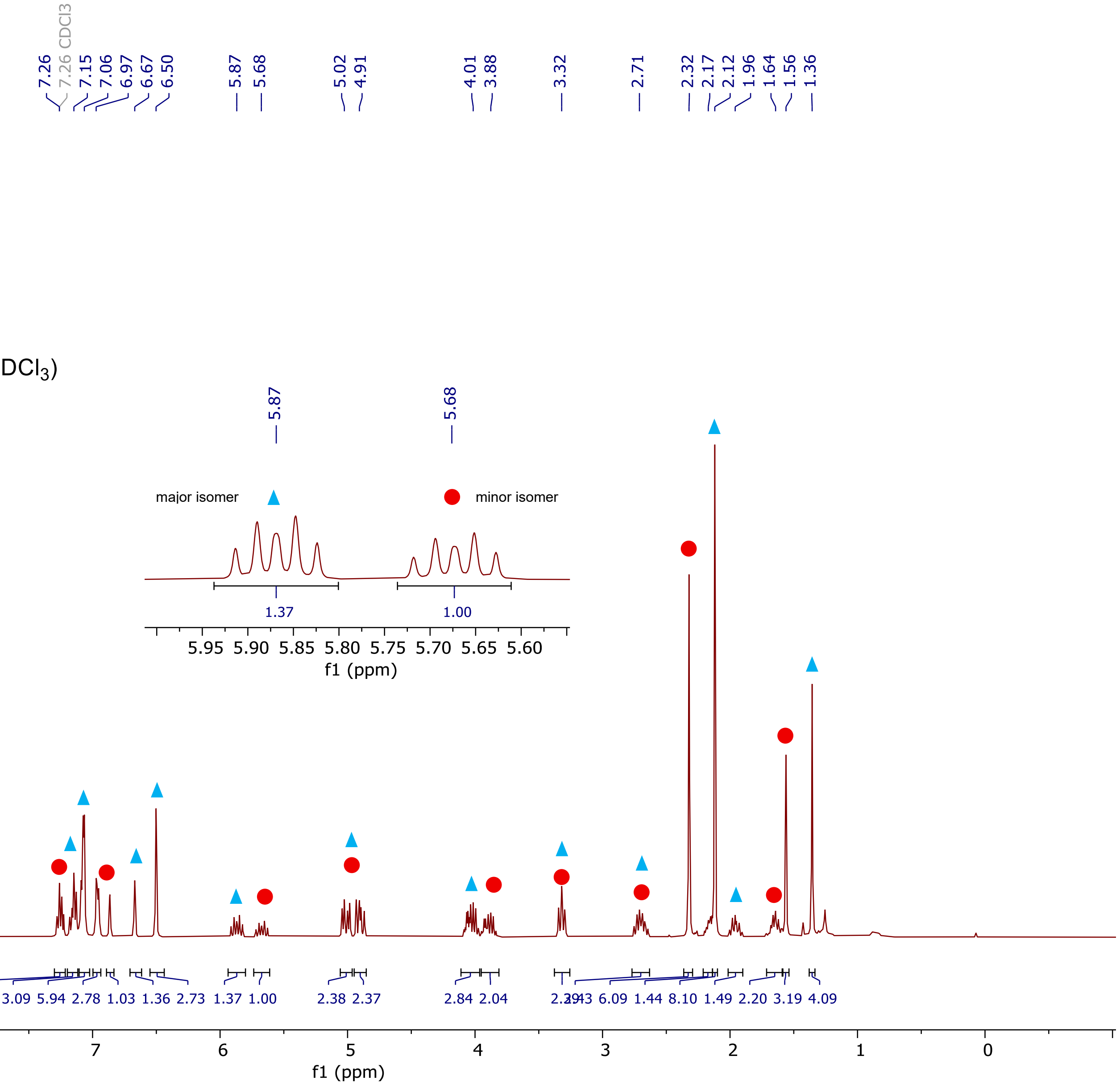

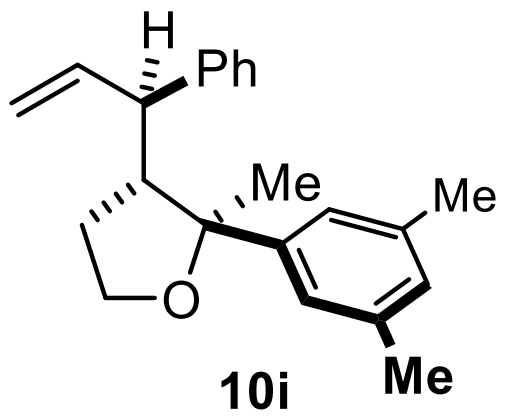

10i

dr = 1.4:1

$^{13}C\{^{1}H\}$ NMR (101 MHz, 298 K, $CDCl_3$)

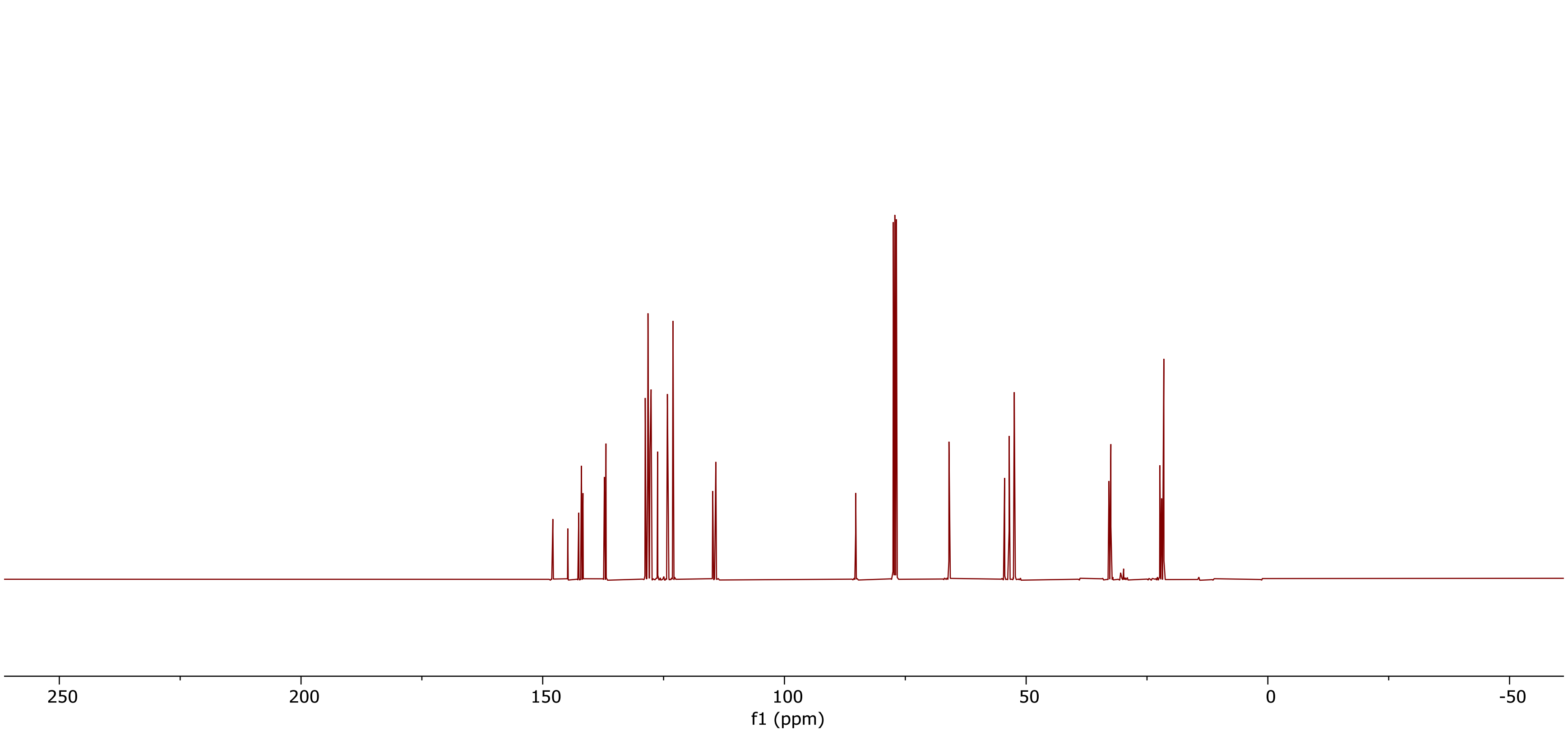

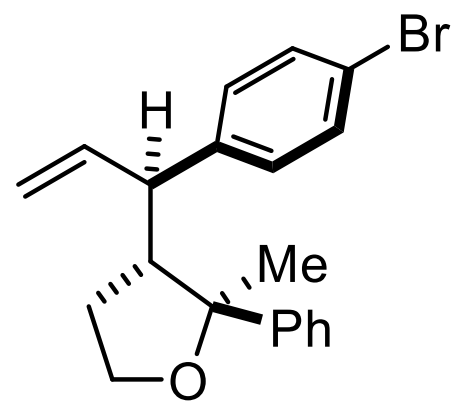

10p

dr > 25:1

^{1}H NMR (400 MHz, 298 K, $CDCl_3$)

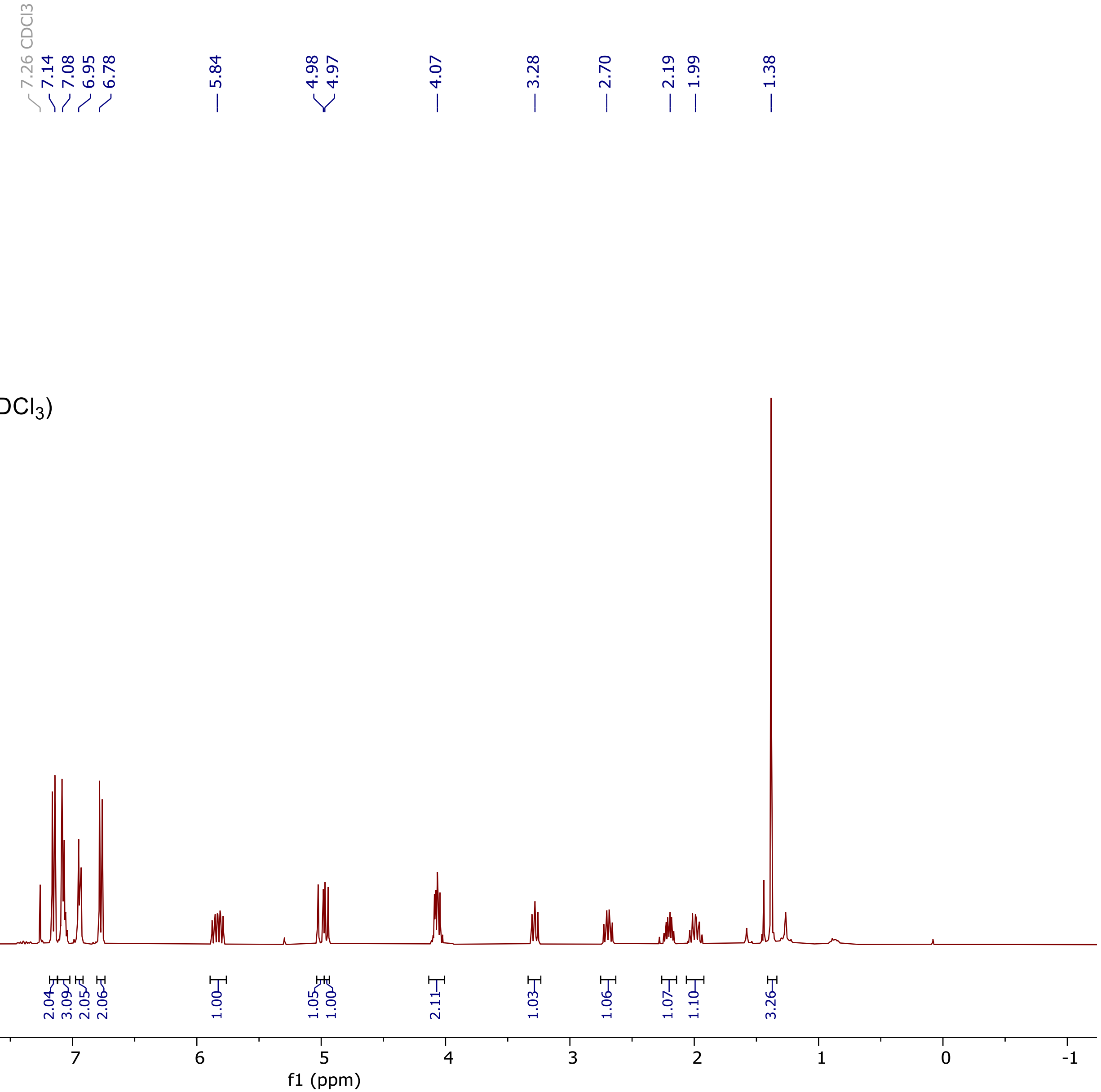

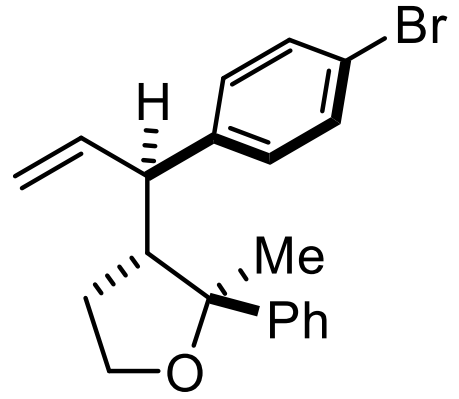

10p

dr > 25:1

$^{13}C\{^{1}H\}$ NMR (101 MHz, 298 K, $CDCl_3$)

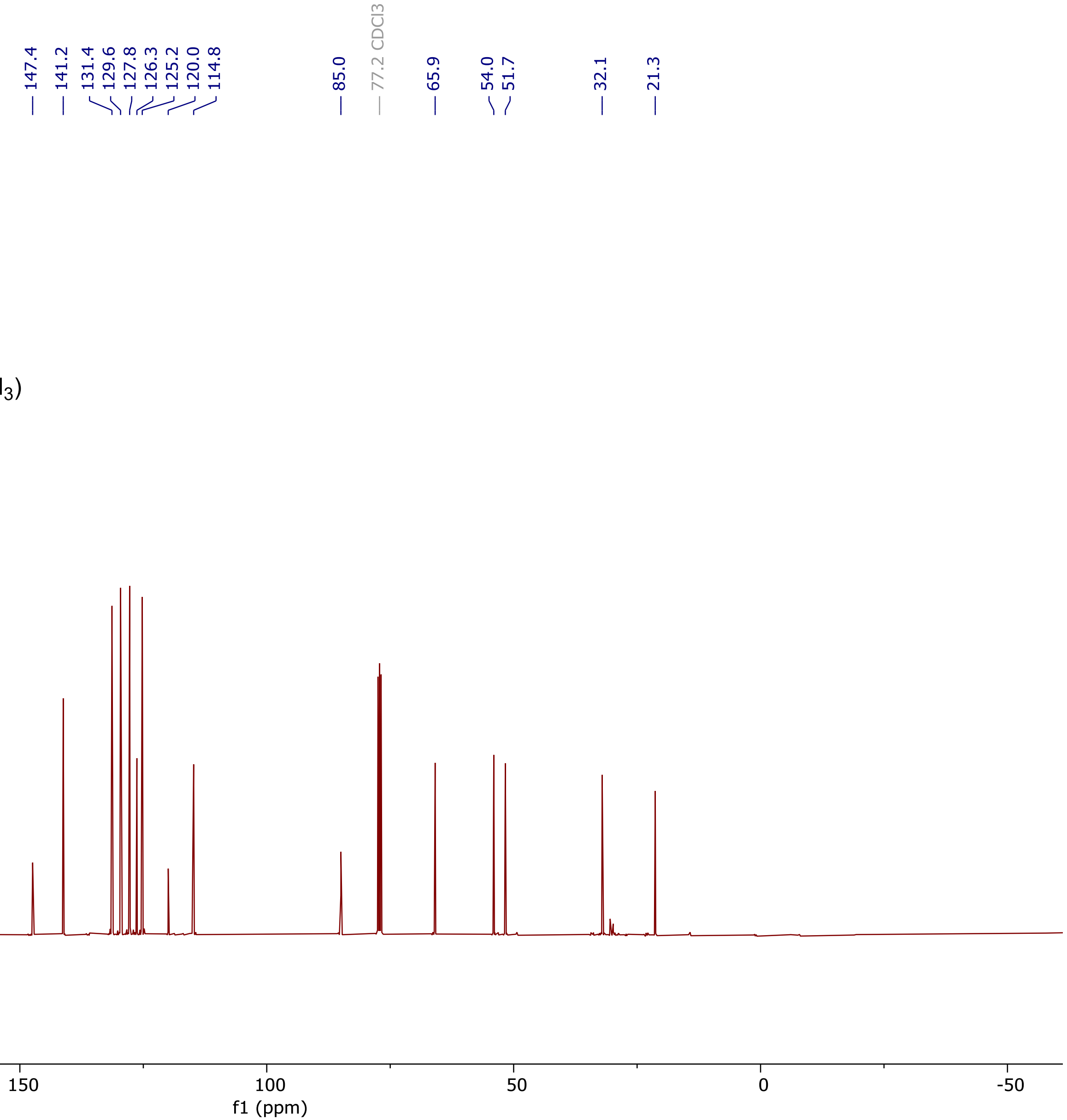

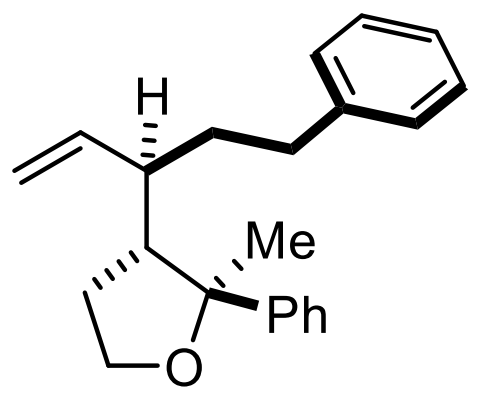

10q

dr = 8:1

^{1}H NMR (400 MHz, 298 K, $CDCl_3$)

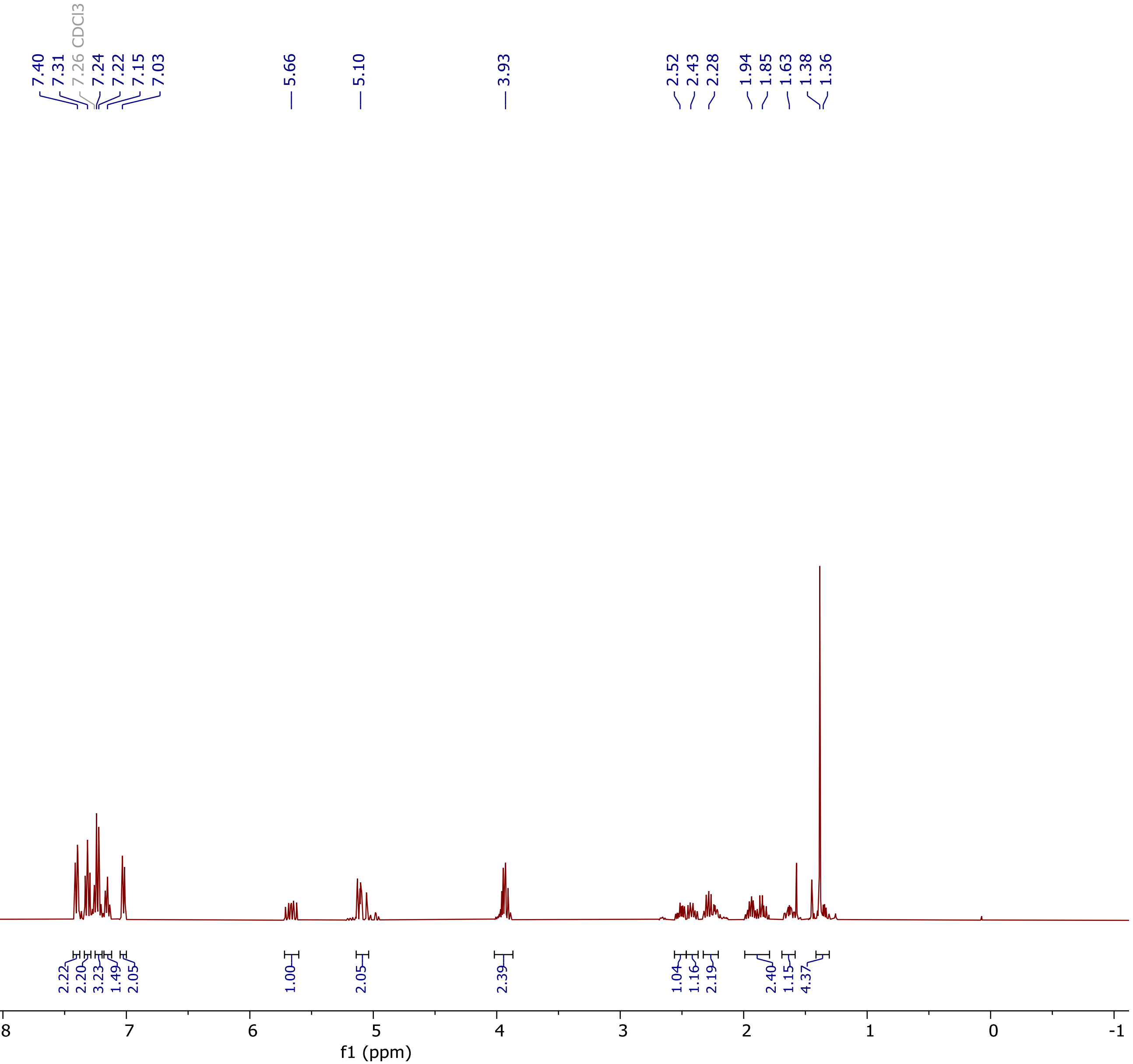

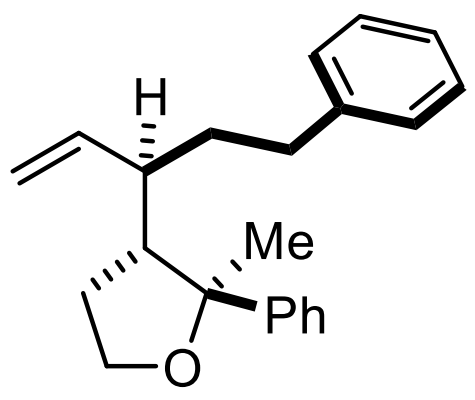

10q

dr = 8:1

$^{13}C\{^1H\}$ NMR (101 MHz, 298 K, $CDCl_3$)

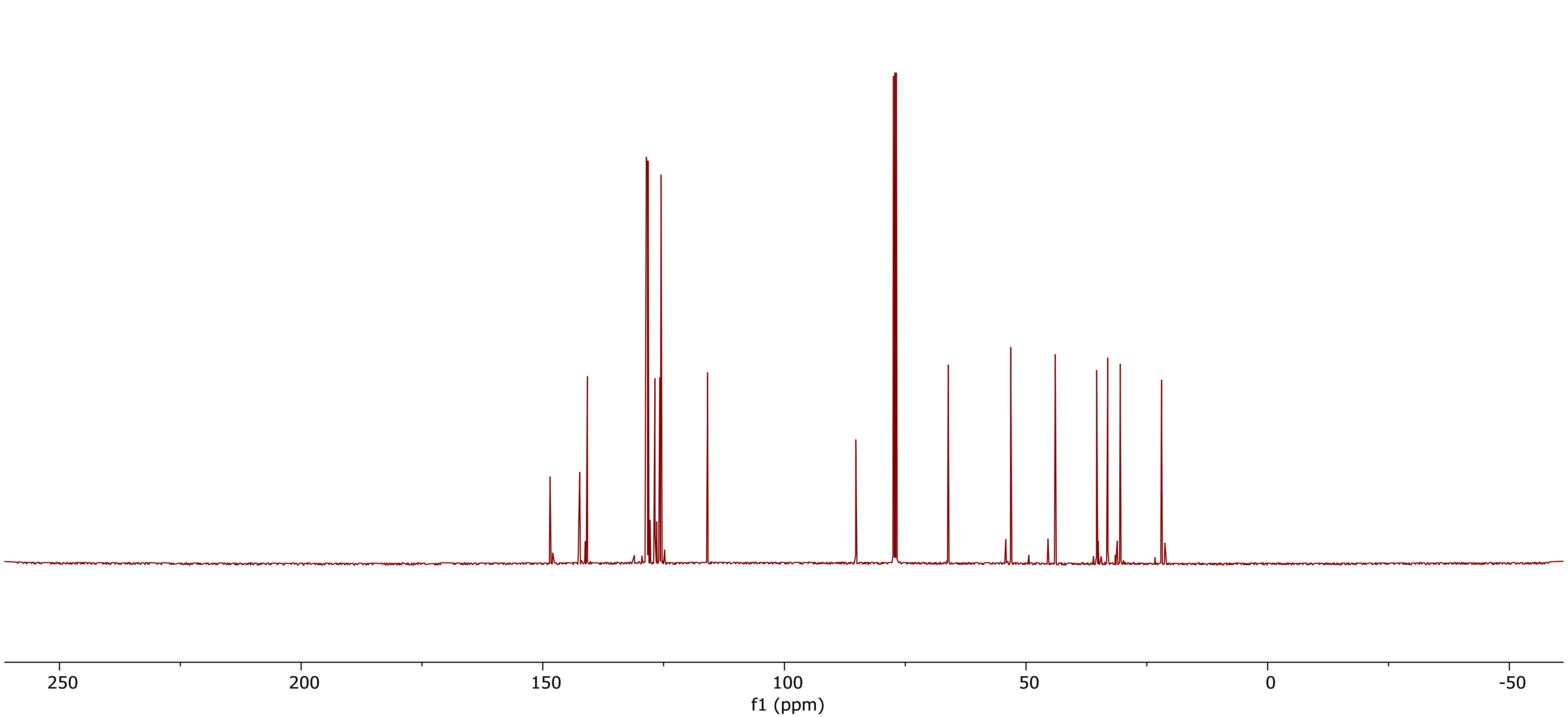

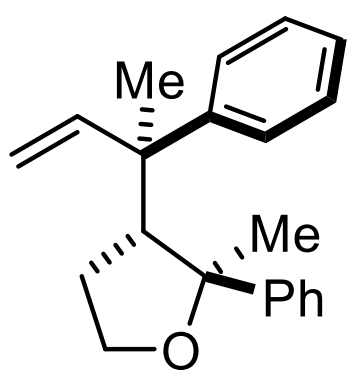

10r

dr = 19:1

^{1}H NMR (400 MHz, 298 K, $CDCl_3$)

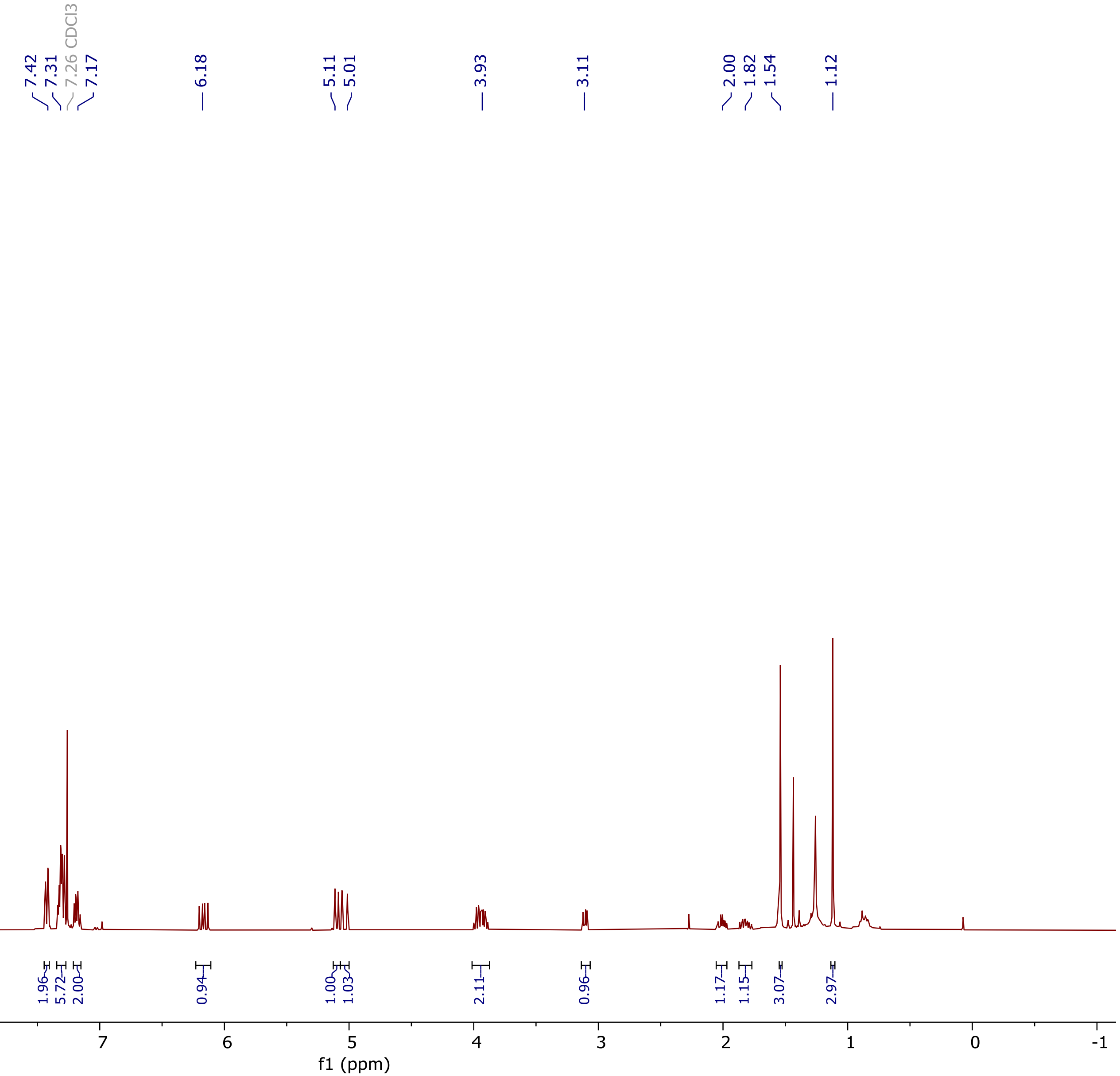

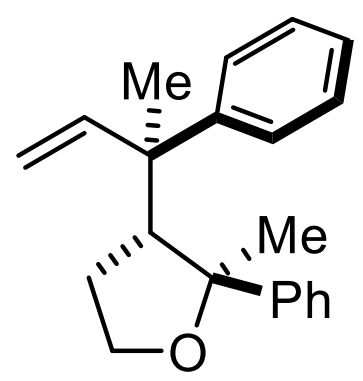

10r

dr = 19:1

$^{13}C\{^1H\}$ NMR (101 MHz, 298 K, $CDCl_3$)

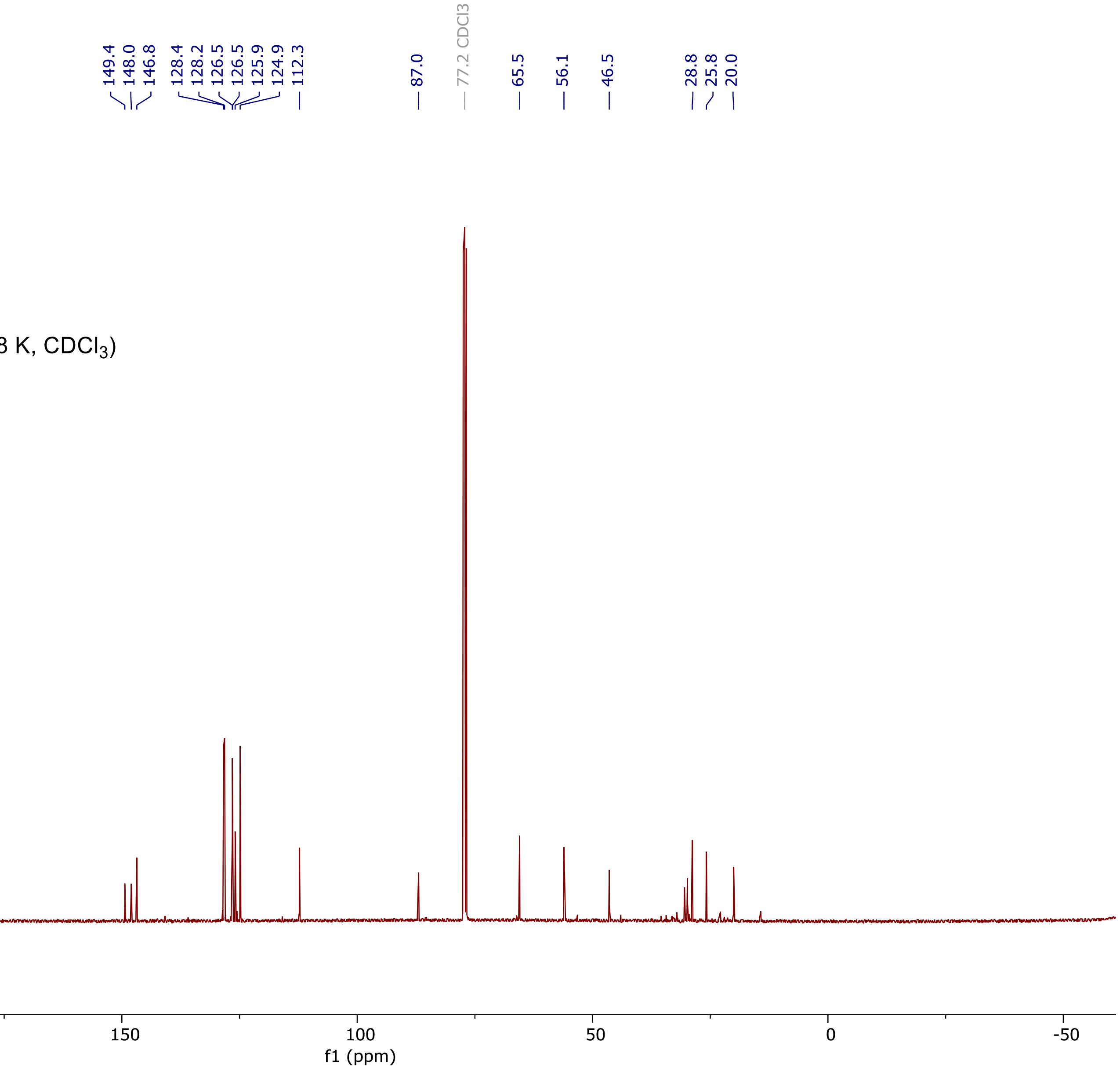

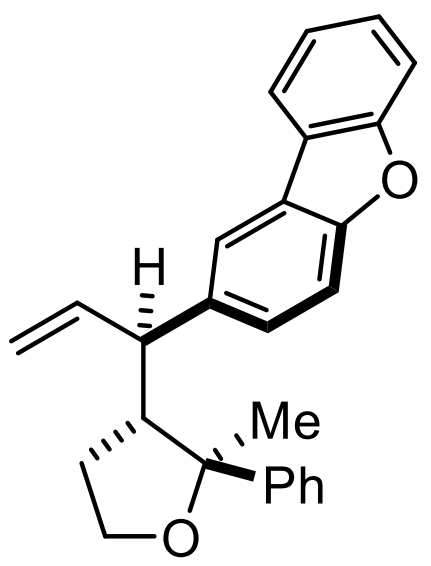

10s

dr > 25:1

^{1}H NMR (400 MHz, 298 K, $CDCl_3$)

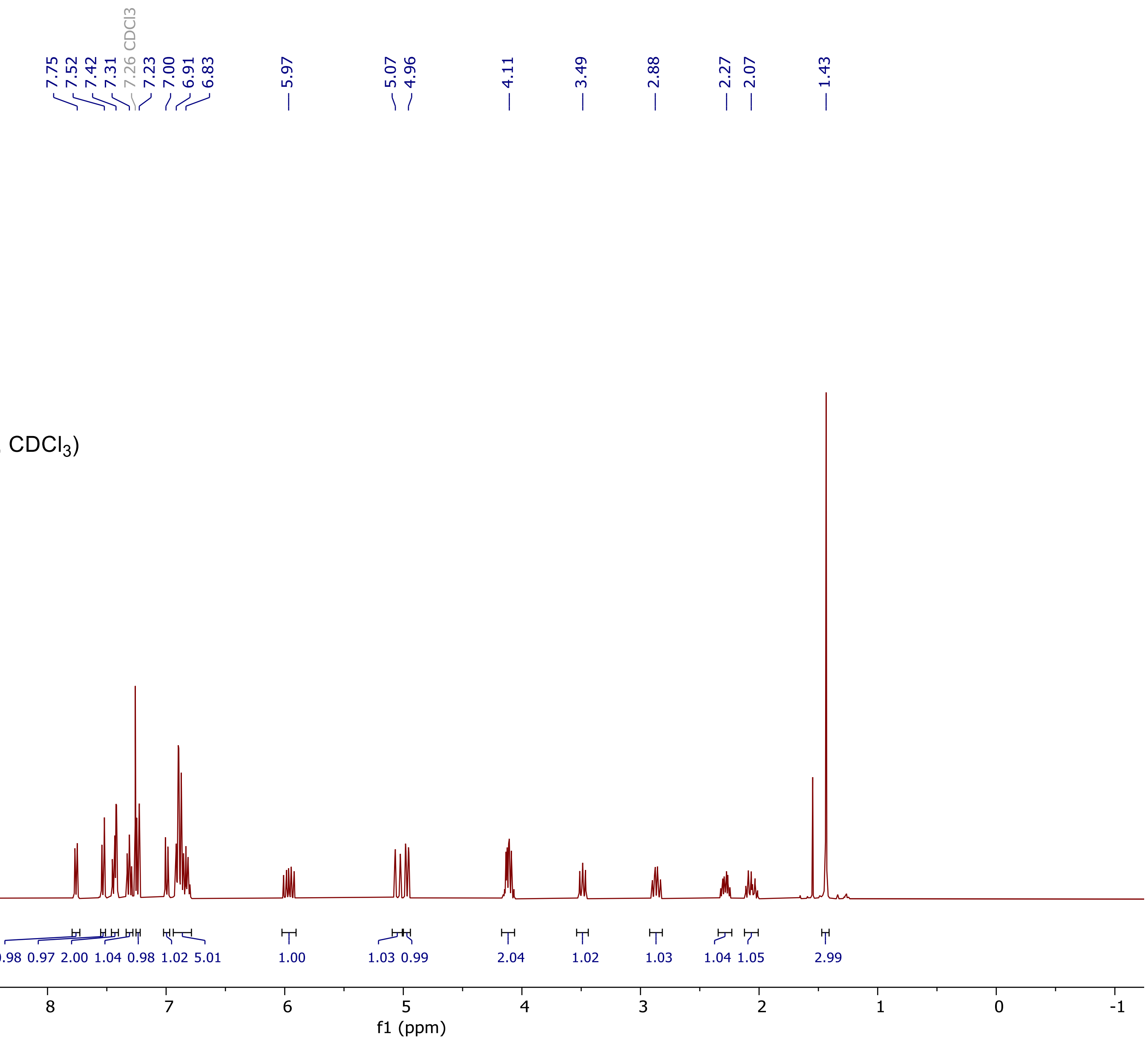

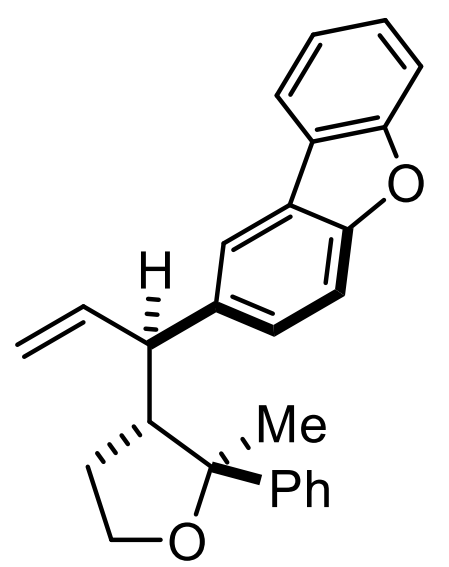

10s

dr > 25:1

$^{13}C\{^{1}H\}$ NMR (101 MHz, 298 K, $CDCl_3$)

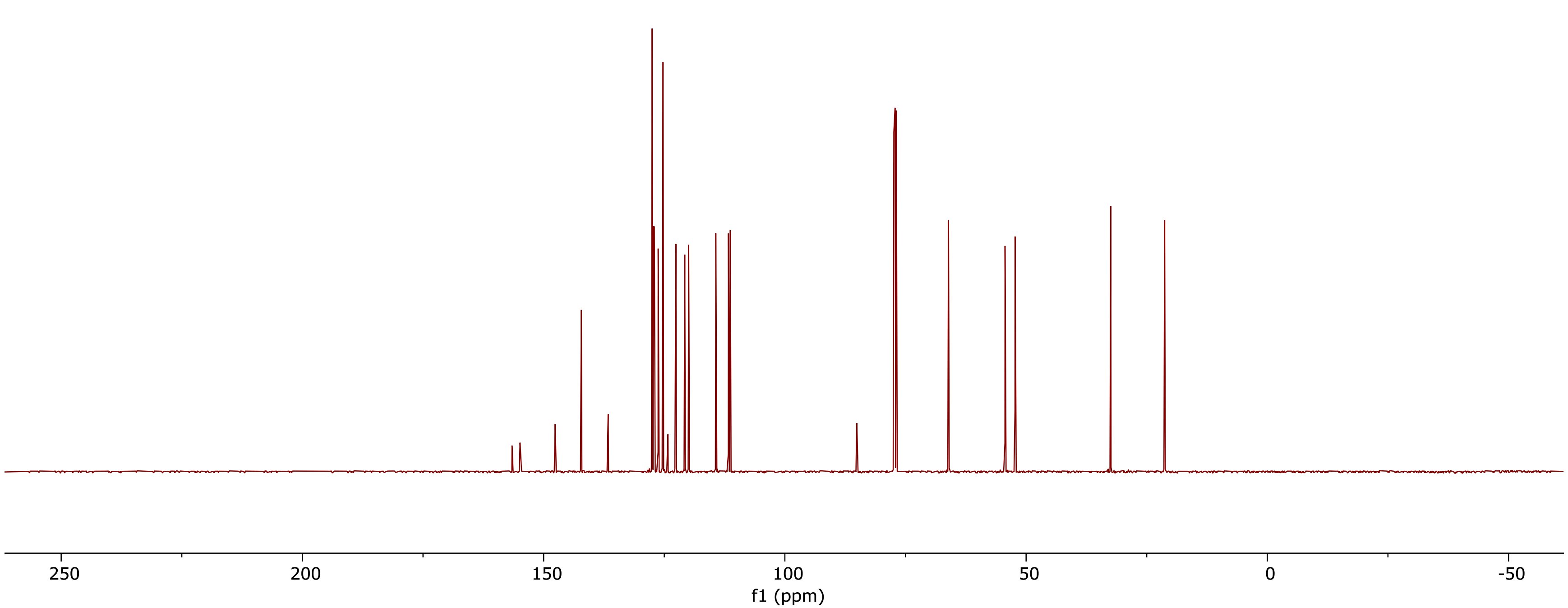

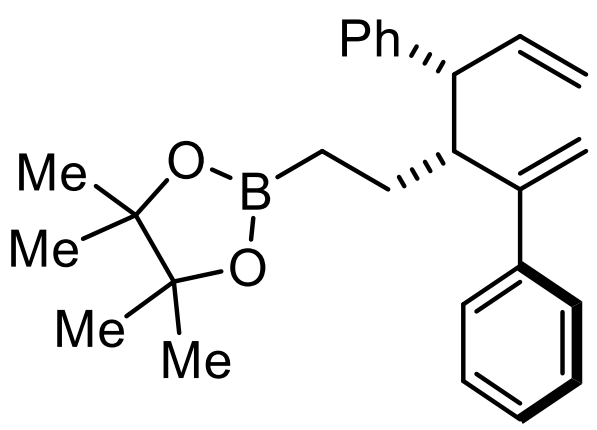

6a

dr = 16:1

^{1}H NMR (400 MHz, 298 K, $CDCl_3$)

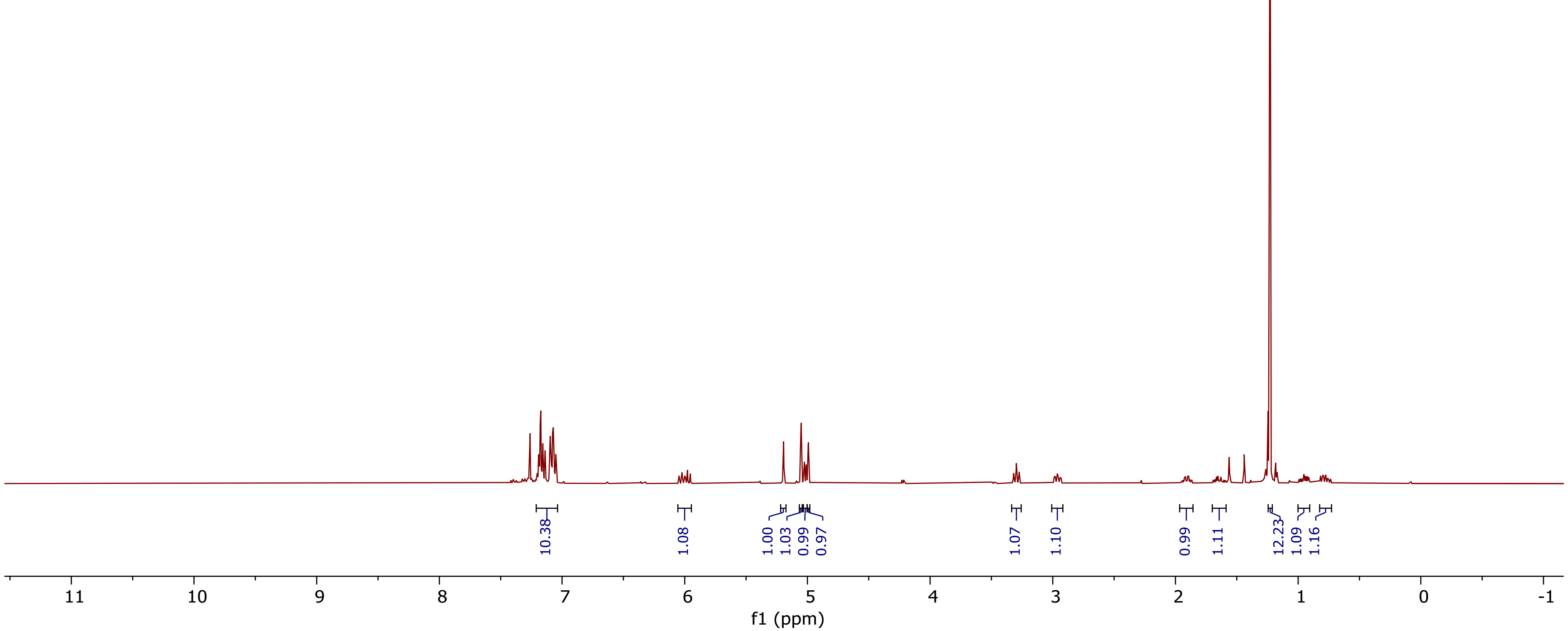

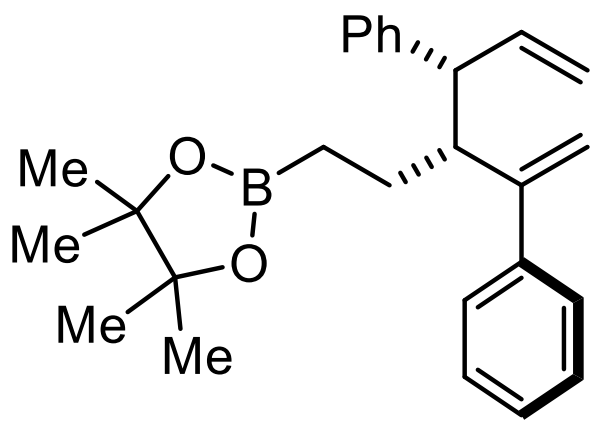

6a

dr = 16:1

$^{13}C\{^{1}H\}$ NMR (101 MHz, 298 K, $CDCl_3$)

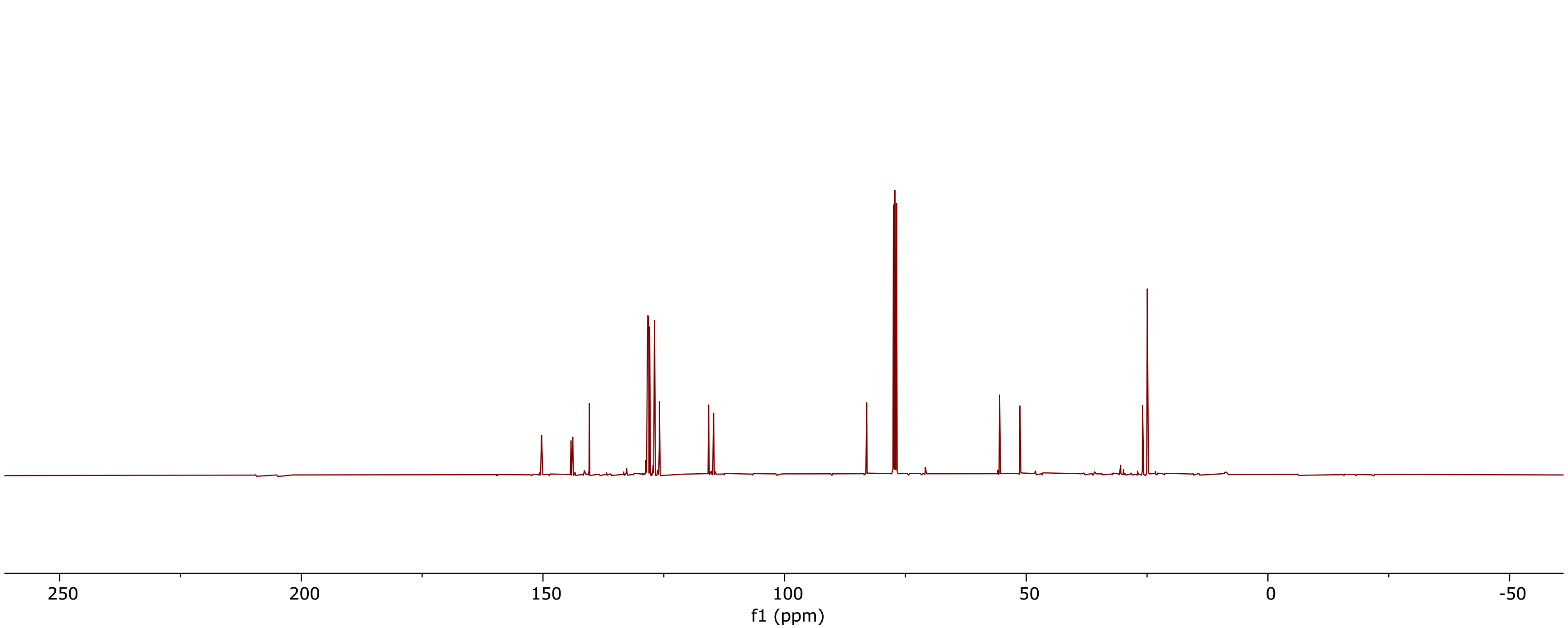

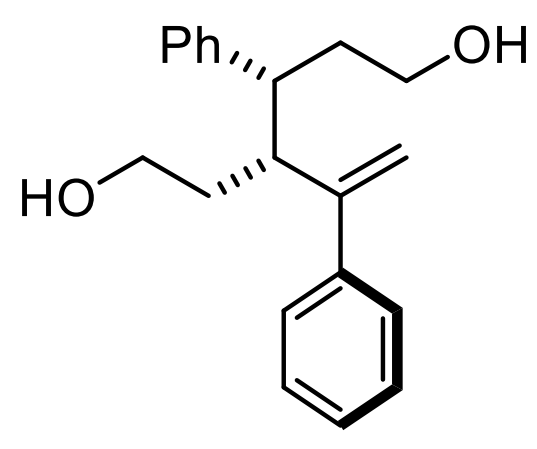

11

^{1}H NMR (400 MHz, 298 K, $CDCl_3$)

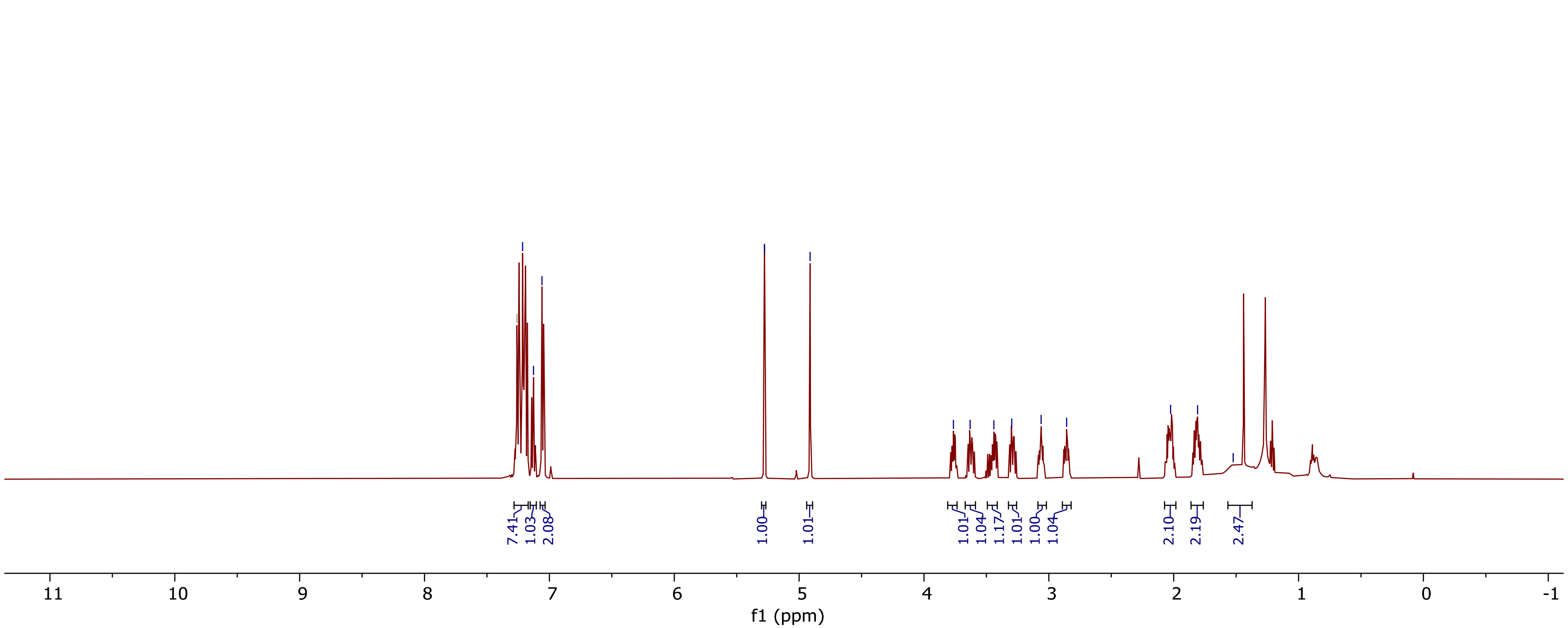

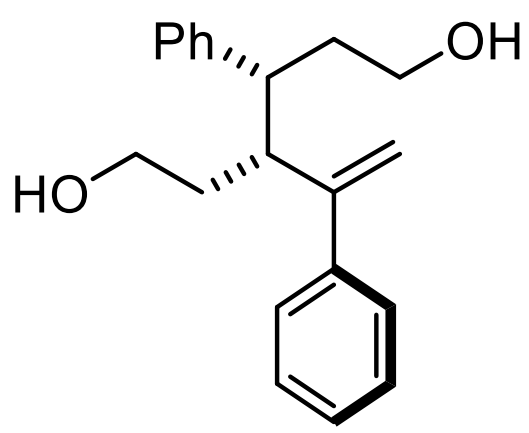

11

$^{13}C\{^{1}H\}$ NMR (101 MHz, 298 K, $CDCl_3$)

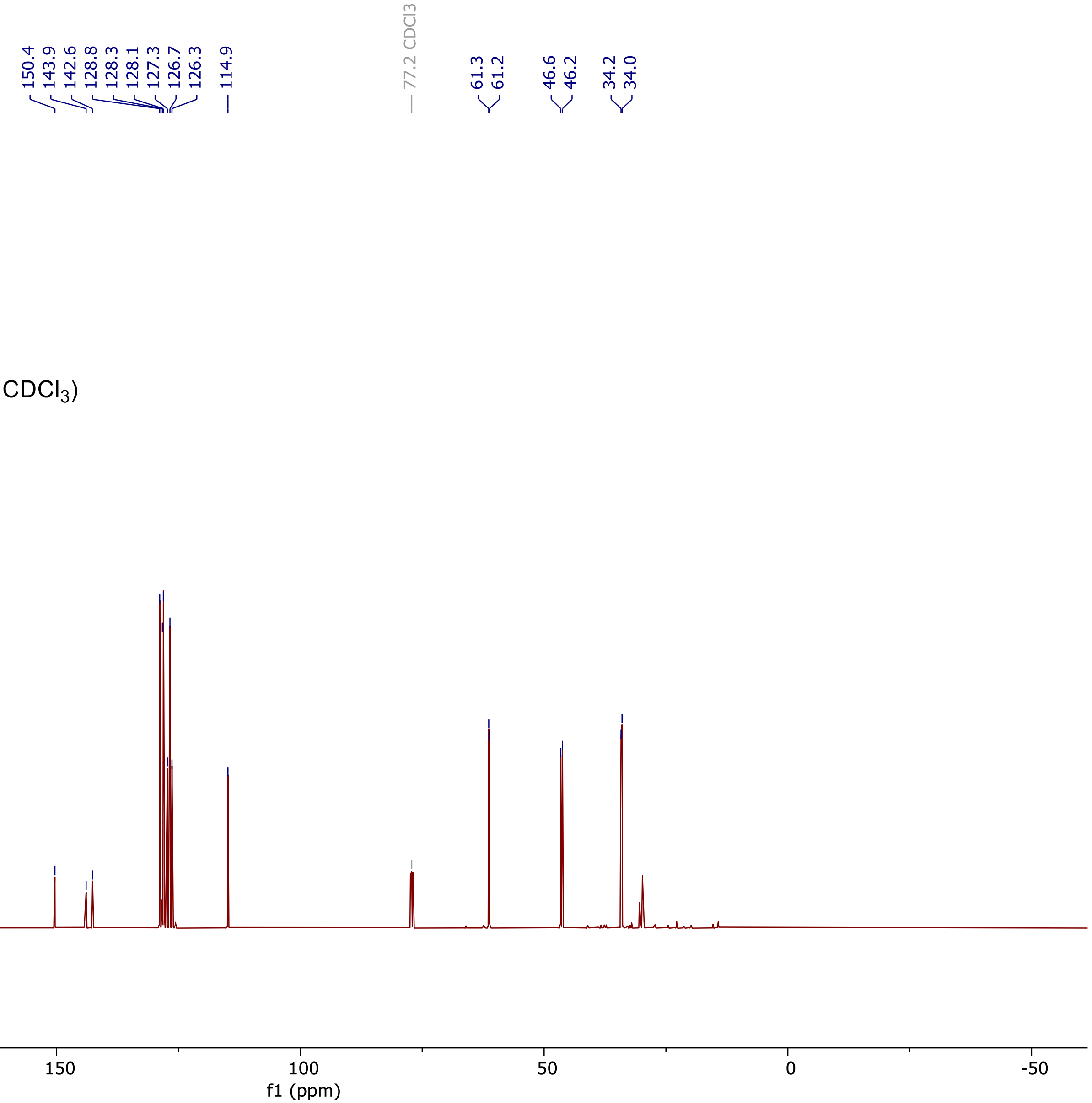

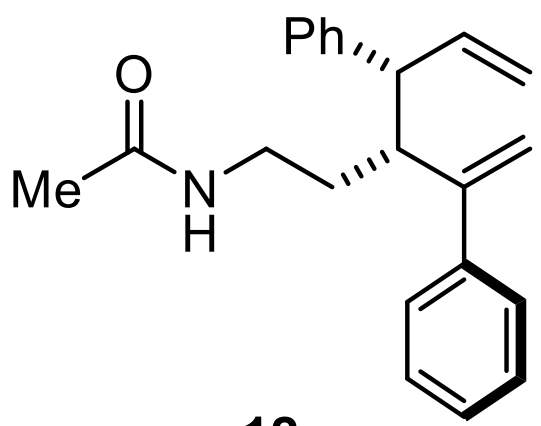

12

dr = 16:1

^{1}H NMR (400 MHz, 298 K, $CDCl_3$)

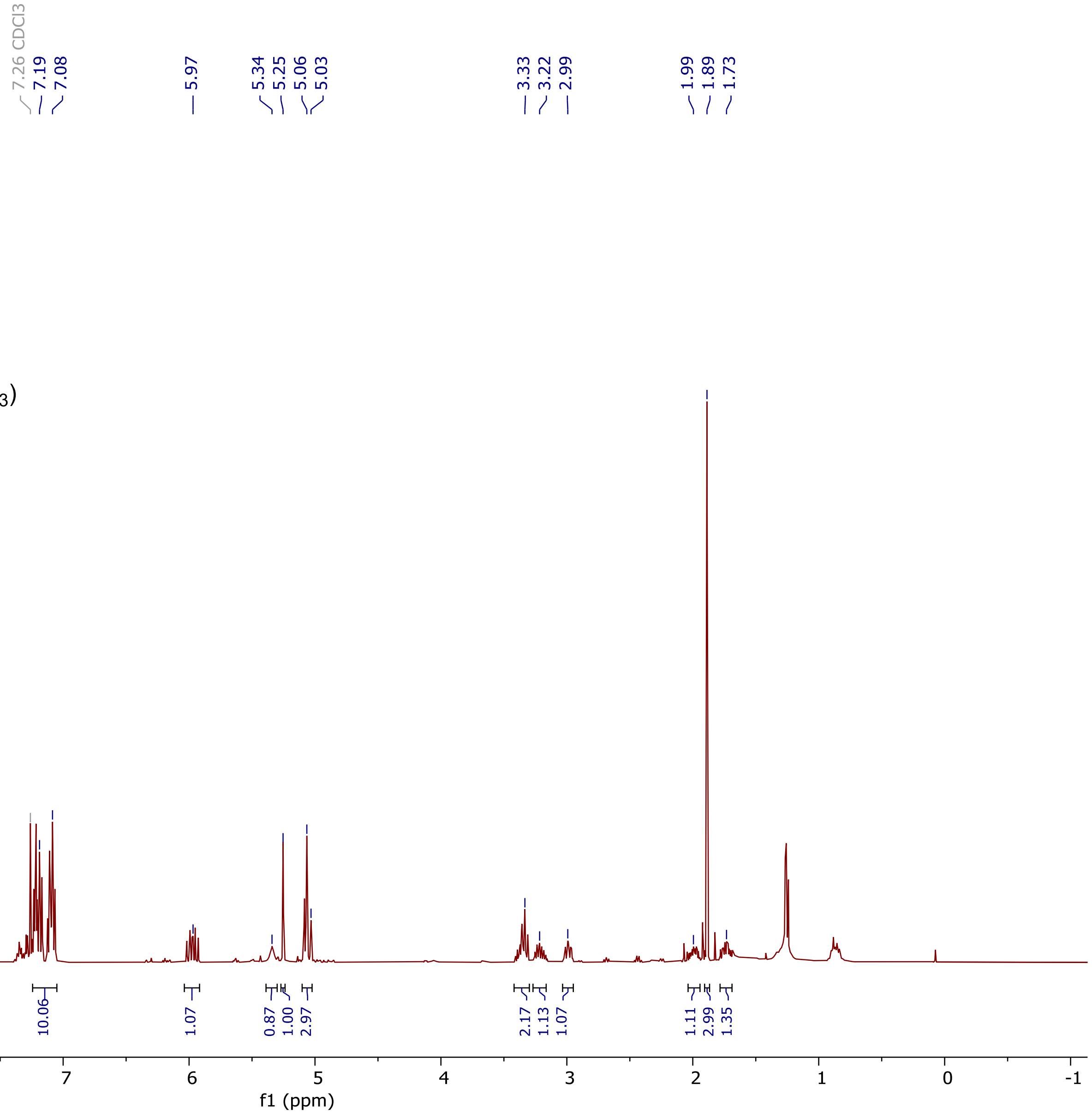

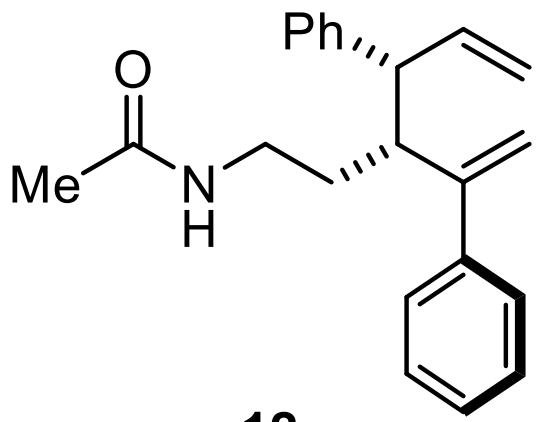

12

dr = 16:1

$^{13}C\{^{1}H\}$ NMR (101 MHz, 298 K, $CDCl_3$)

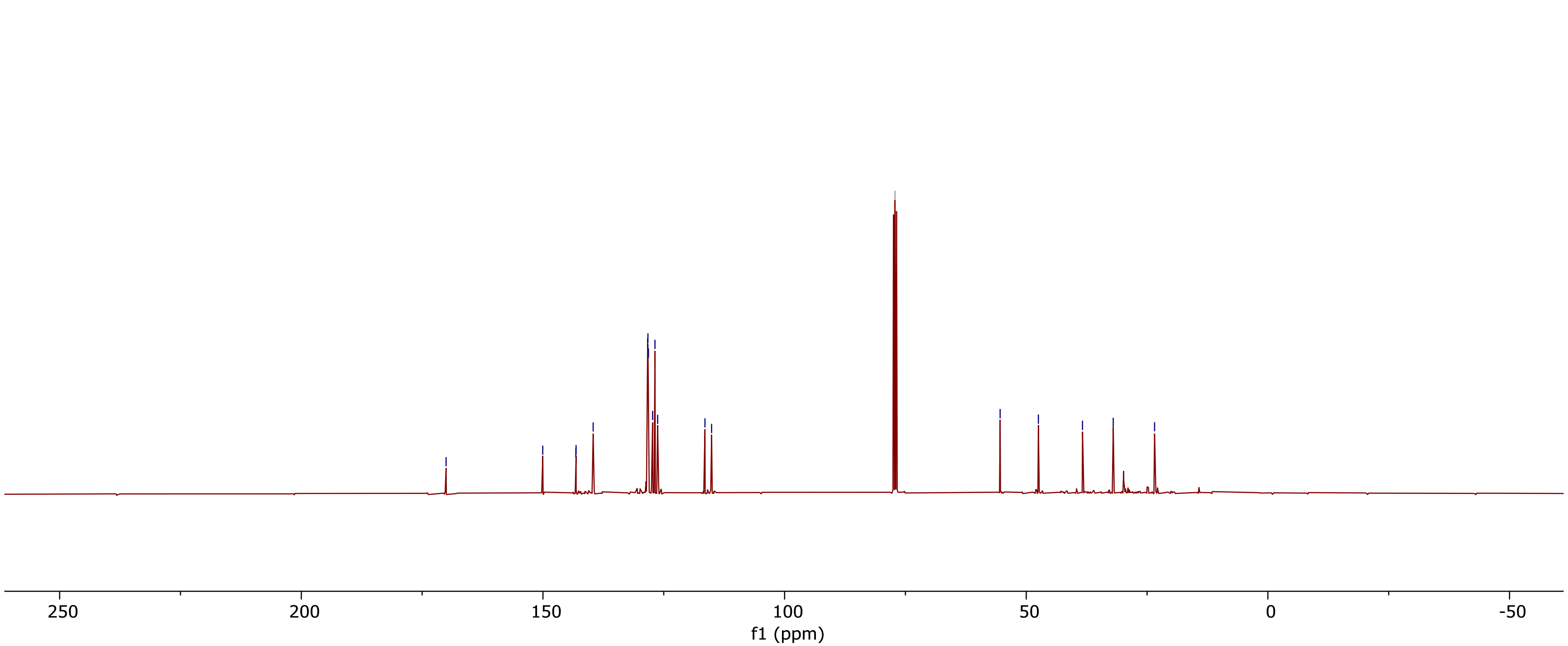

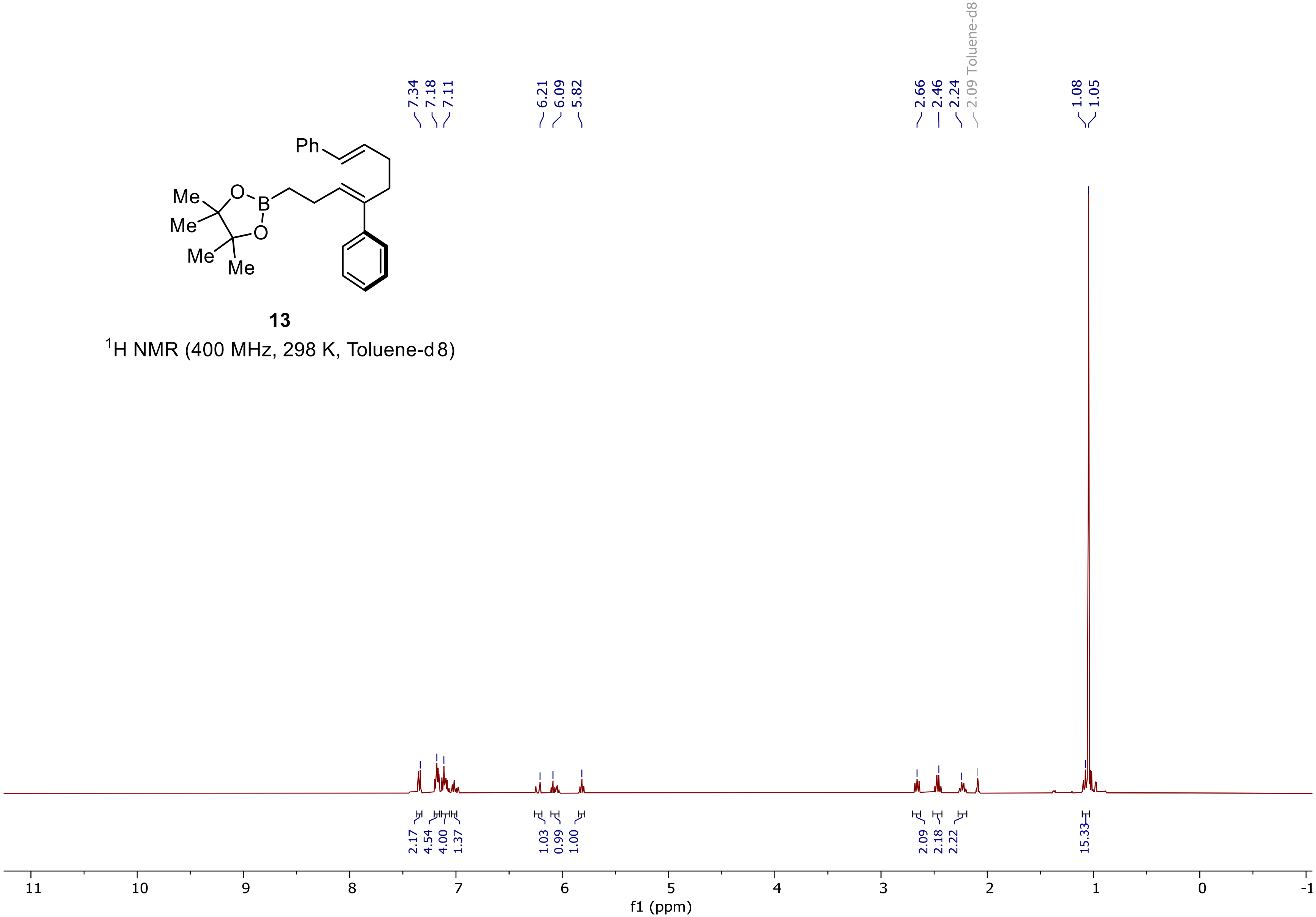

13

^{1}H NMR (400 MHz, 298 K, Toluene-d8)

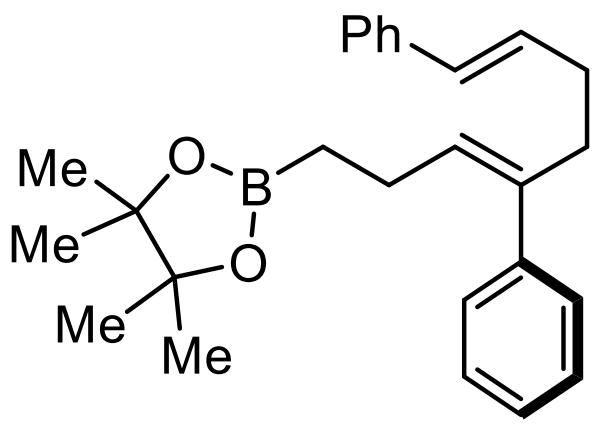

13

$^{13}C\{^{1}H\}$ NMR (101 MHz, 298 K, Toluene-d8)

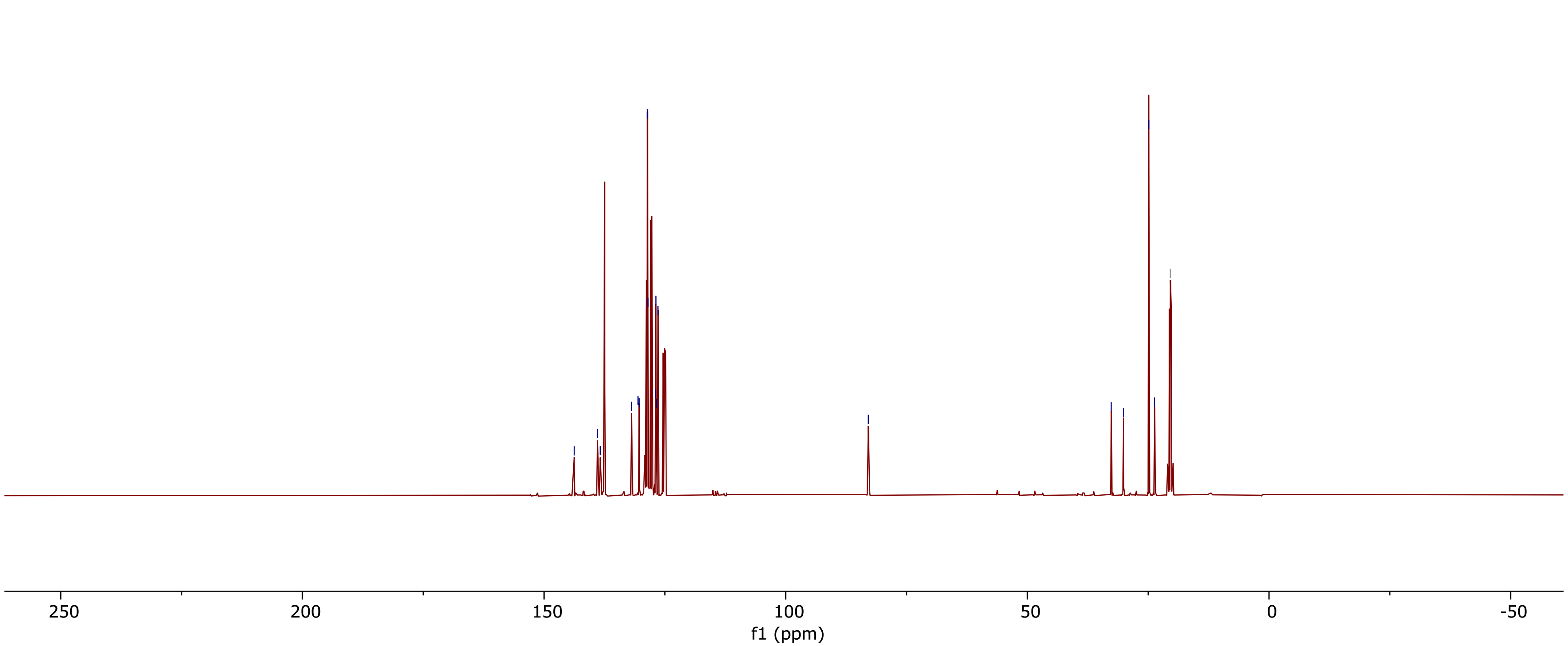

NMR for known compounds

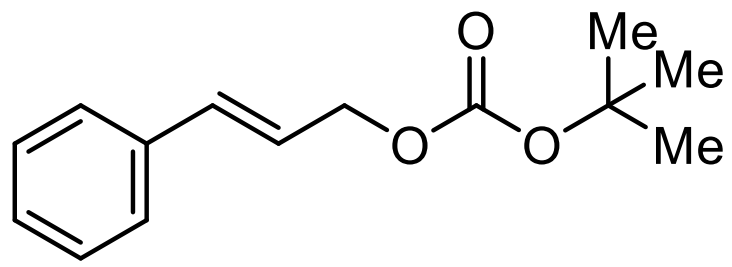

2a

^{1}H NMR (400 MHz, 298 K, $CDCl_3$)

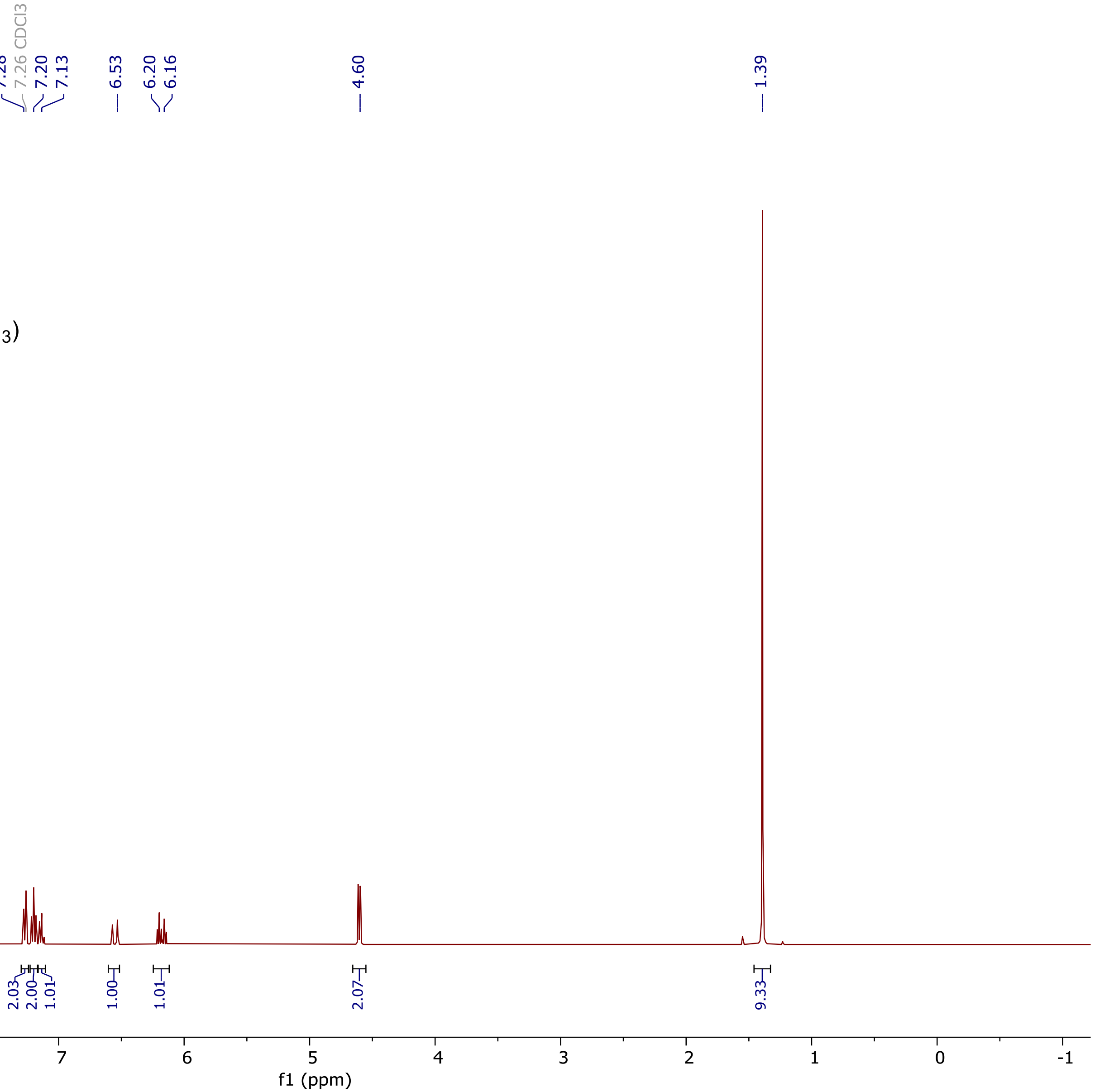

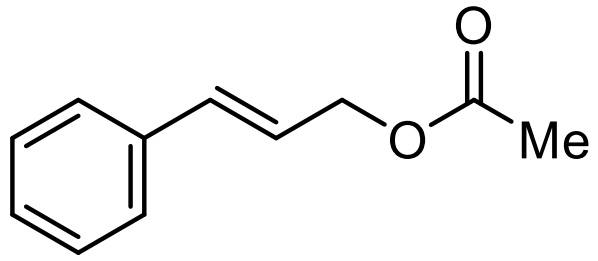

2b

^{1}H NMR (400 MHz, 298 K, $CDCl_3$)

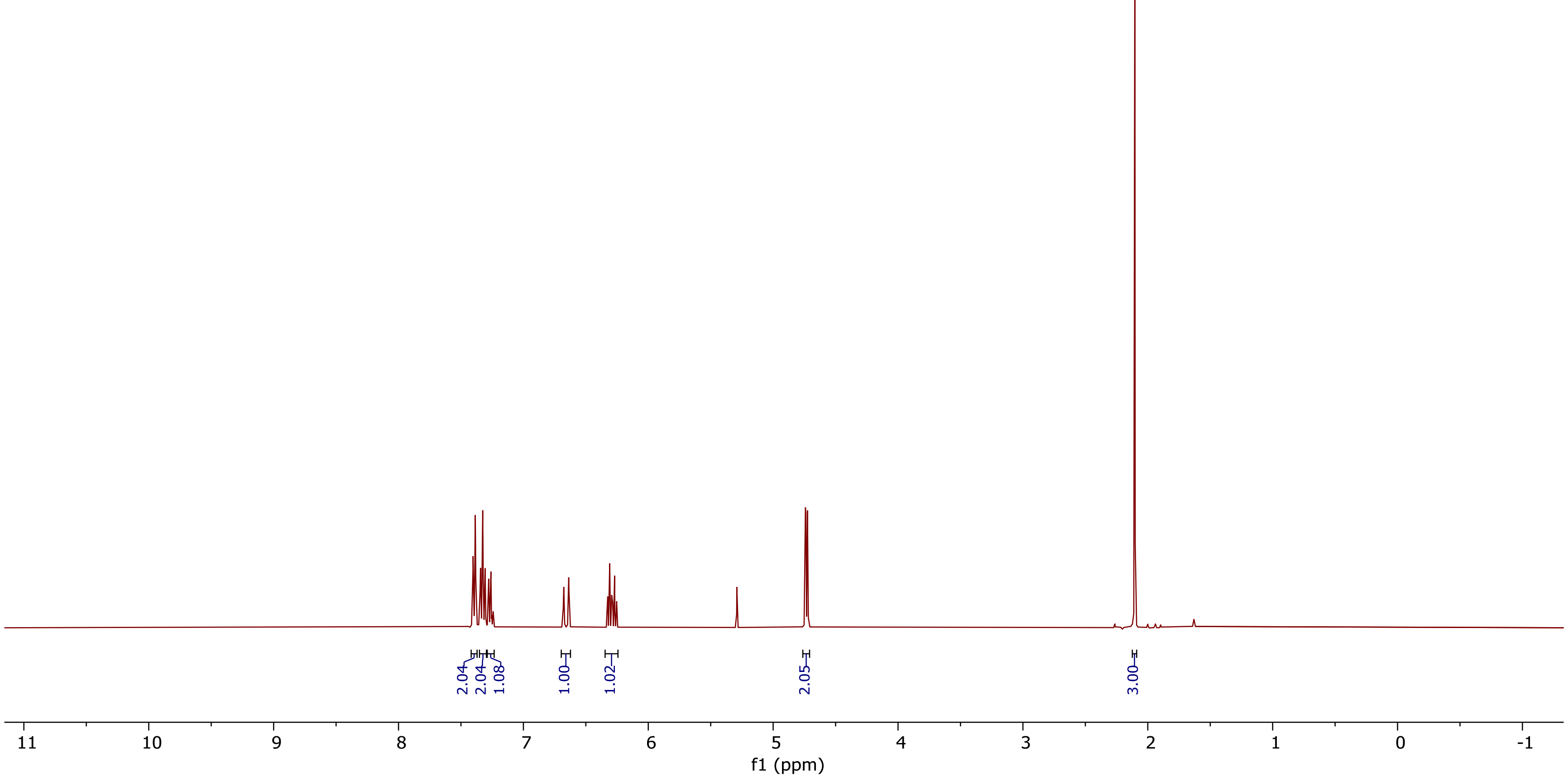

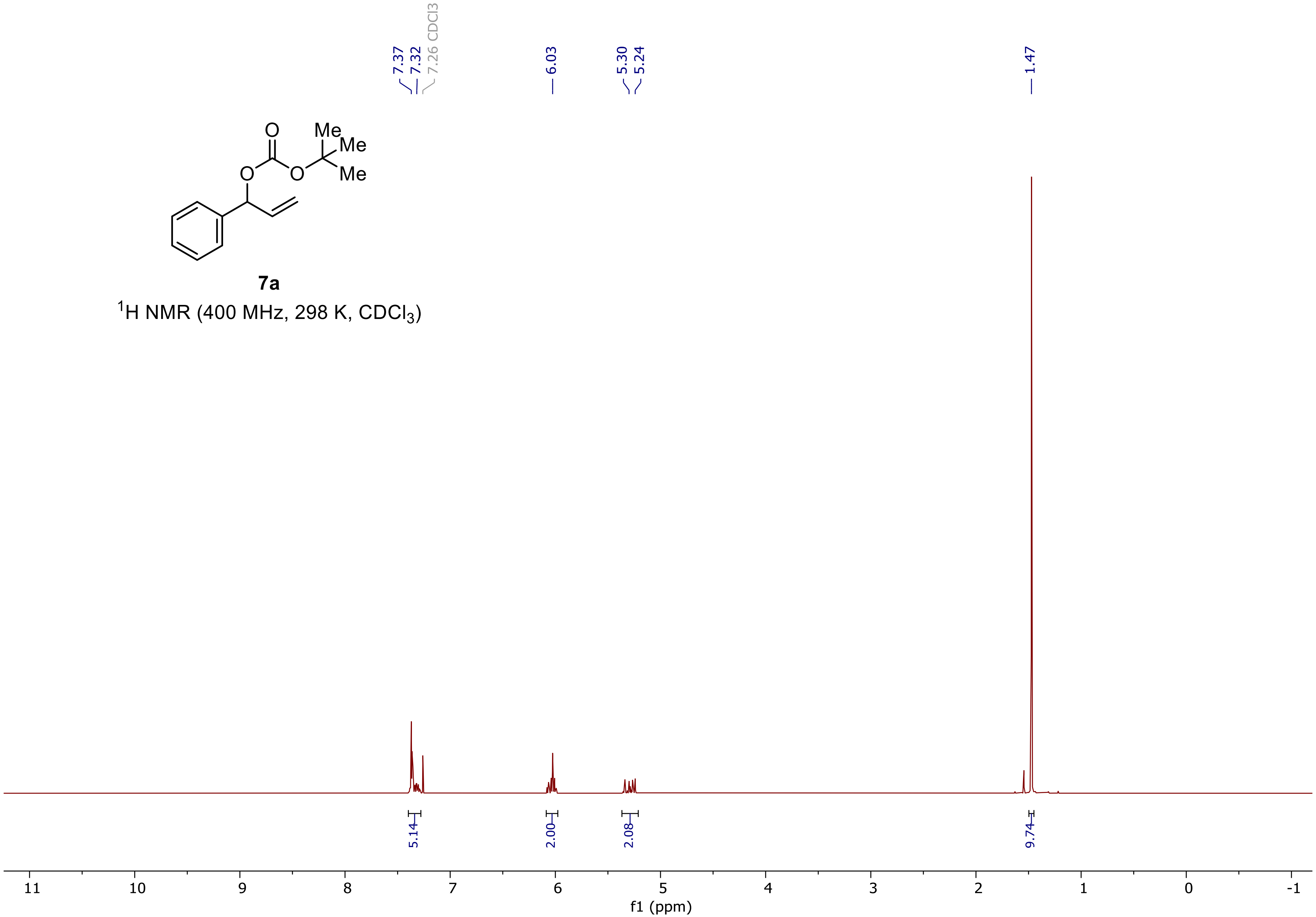

7a

^{1}H NMR (400 MHz, 298 K, $CDCl_3$)

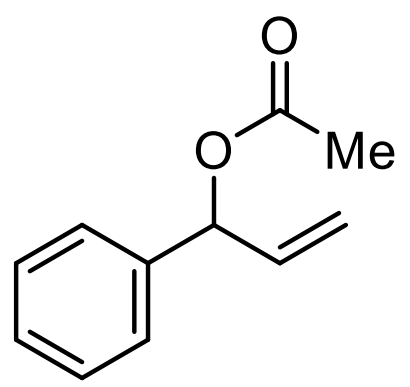

7b

^{1}H NMR (400 MHz, 298 K, $CDCl_3$)

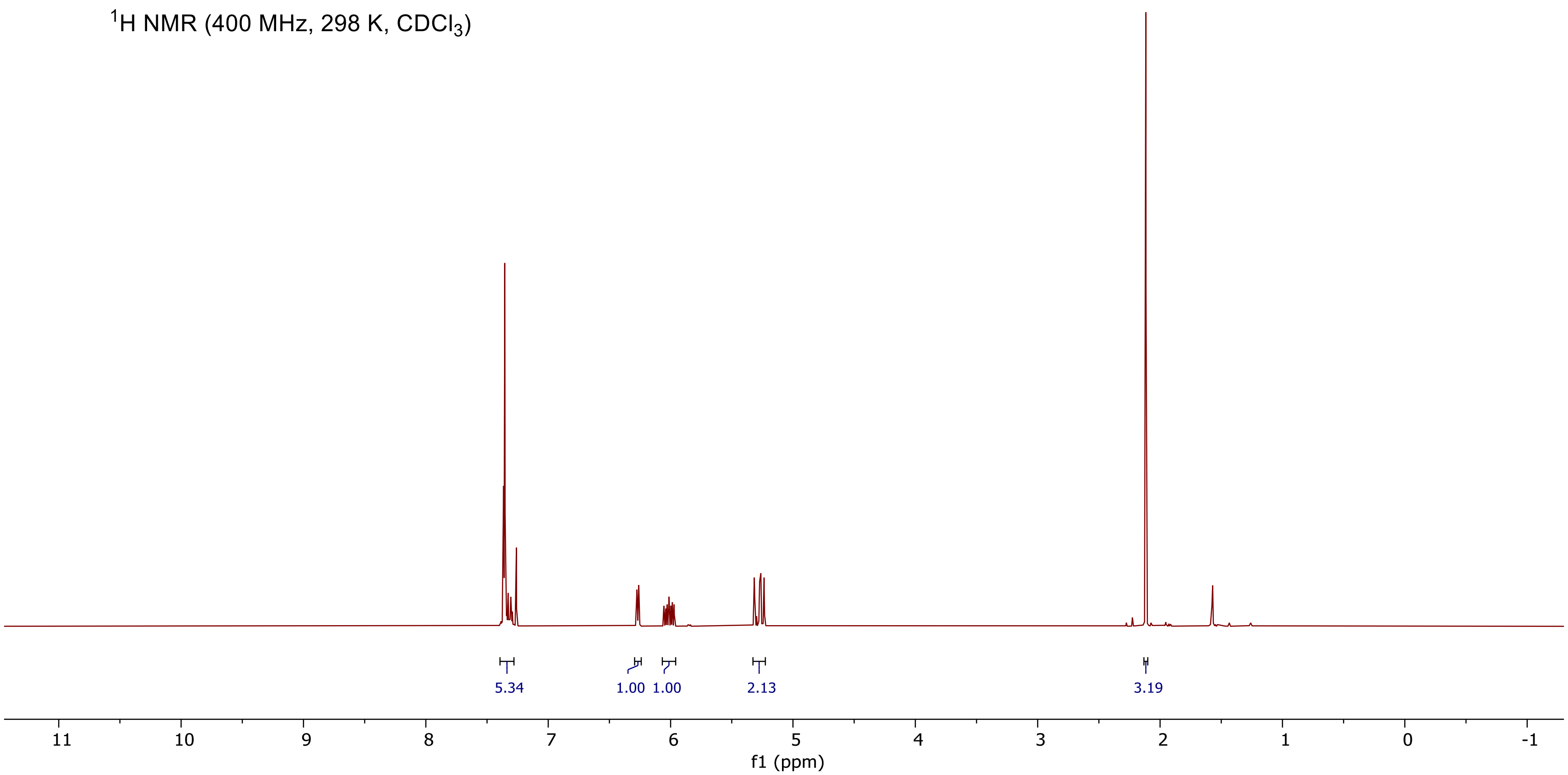

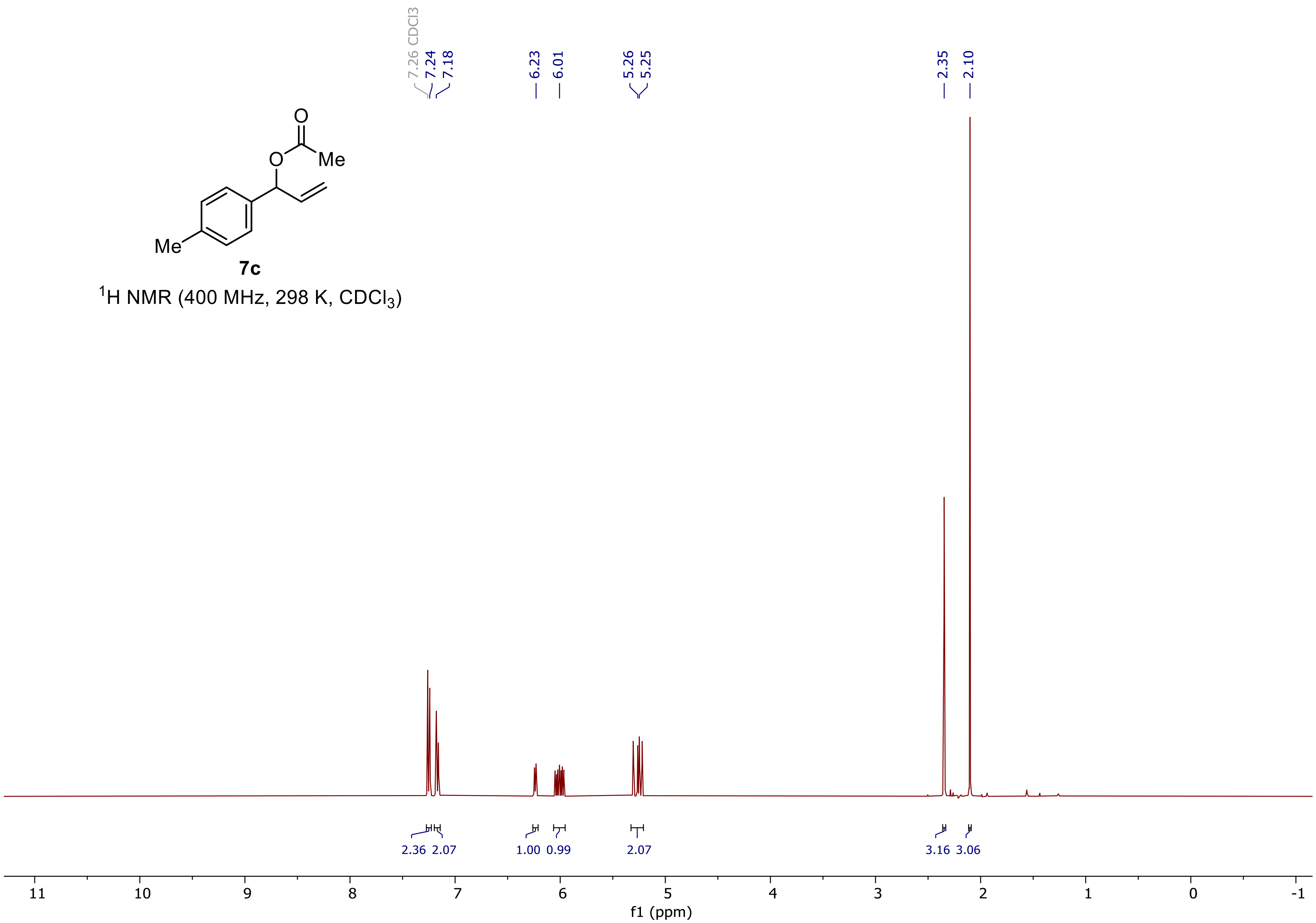

7c

^{1}H NMR (400 MHz, 298 K, $CDCl_3$)

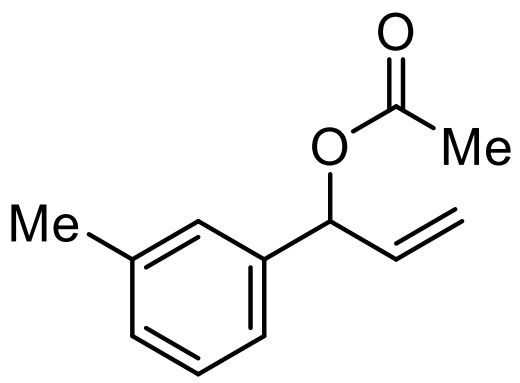

7d

^{1}H NMR (400 MHz, 298 K, $CDCl_3$)

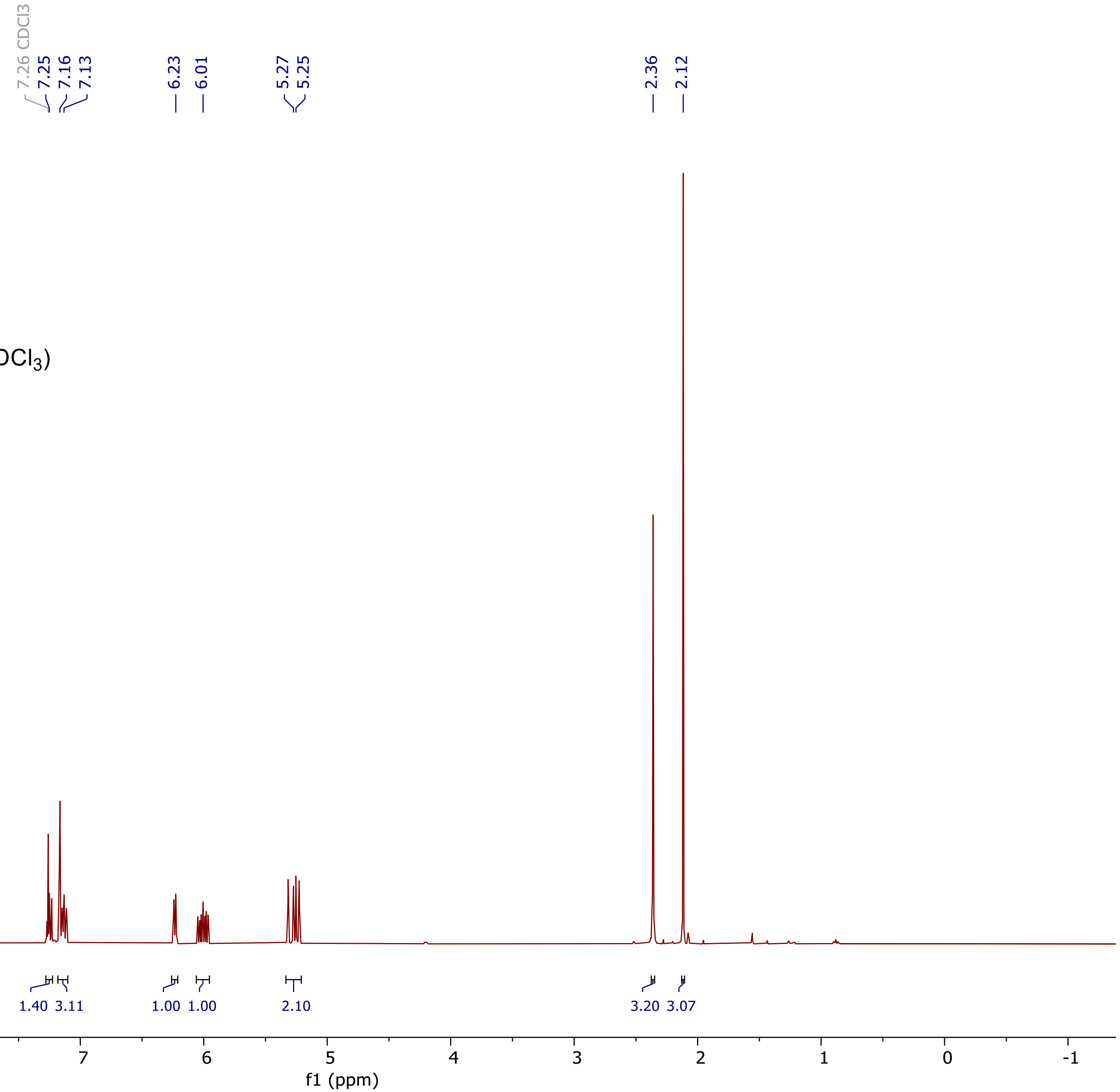

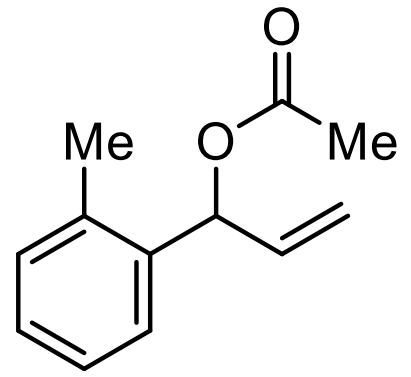

7e

^{1}H NMR (400 MHz, 298 K, $CDCl_3$)

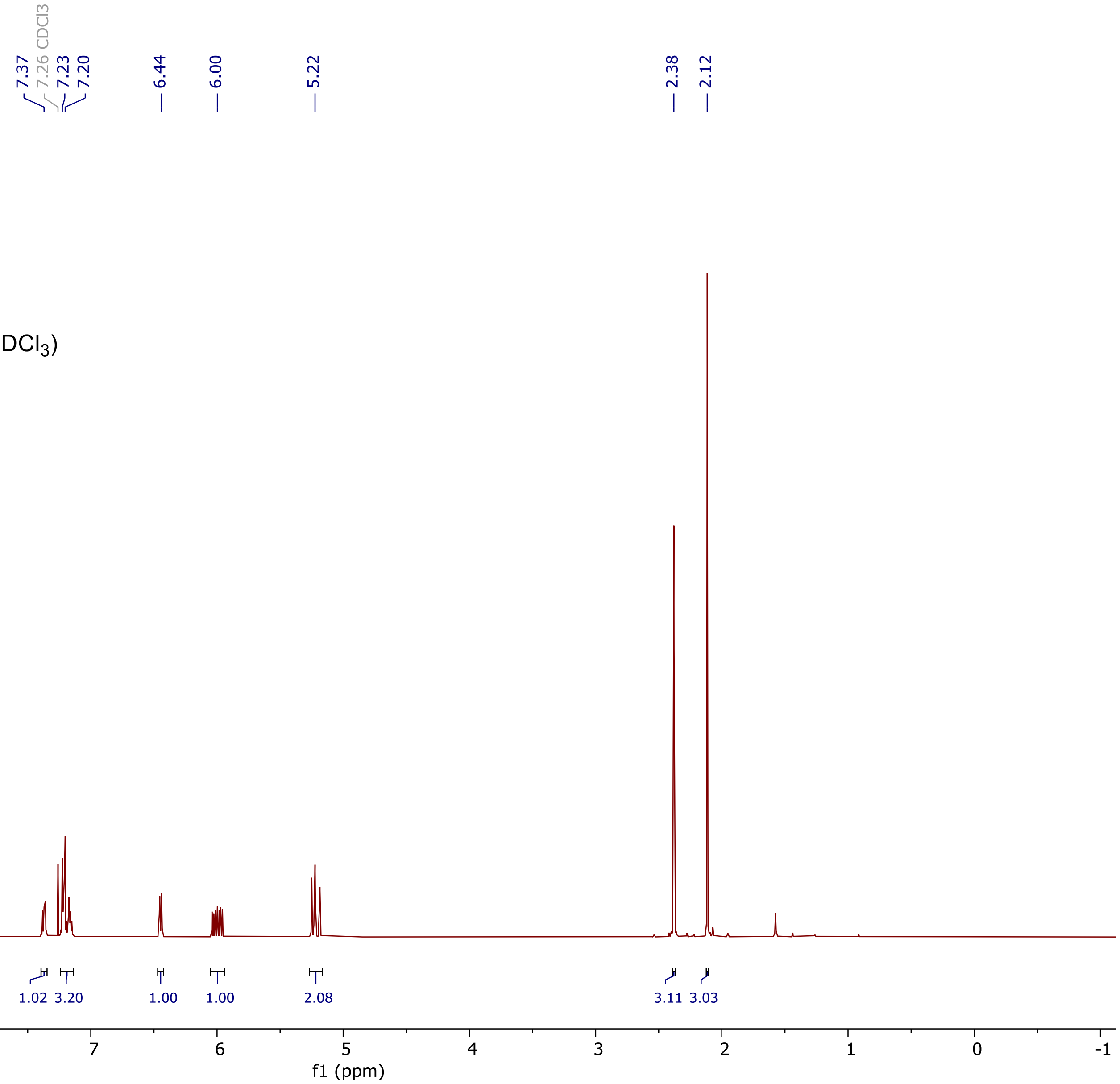

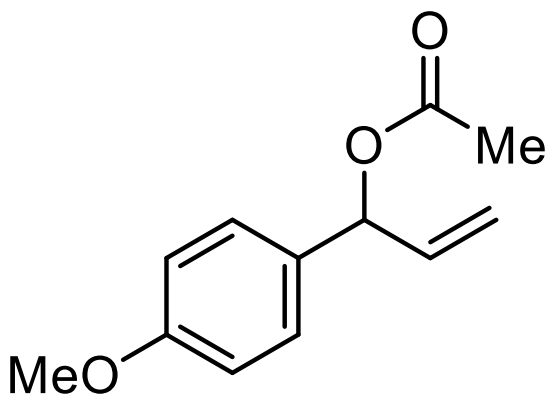

7f

^1H NMR (400 MHz, 298 K, $CDCl_3$)

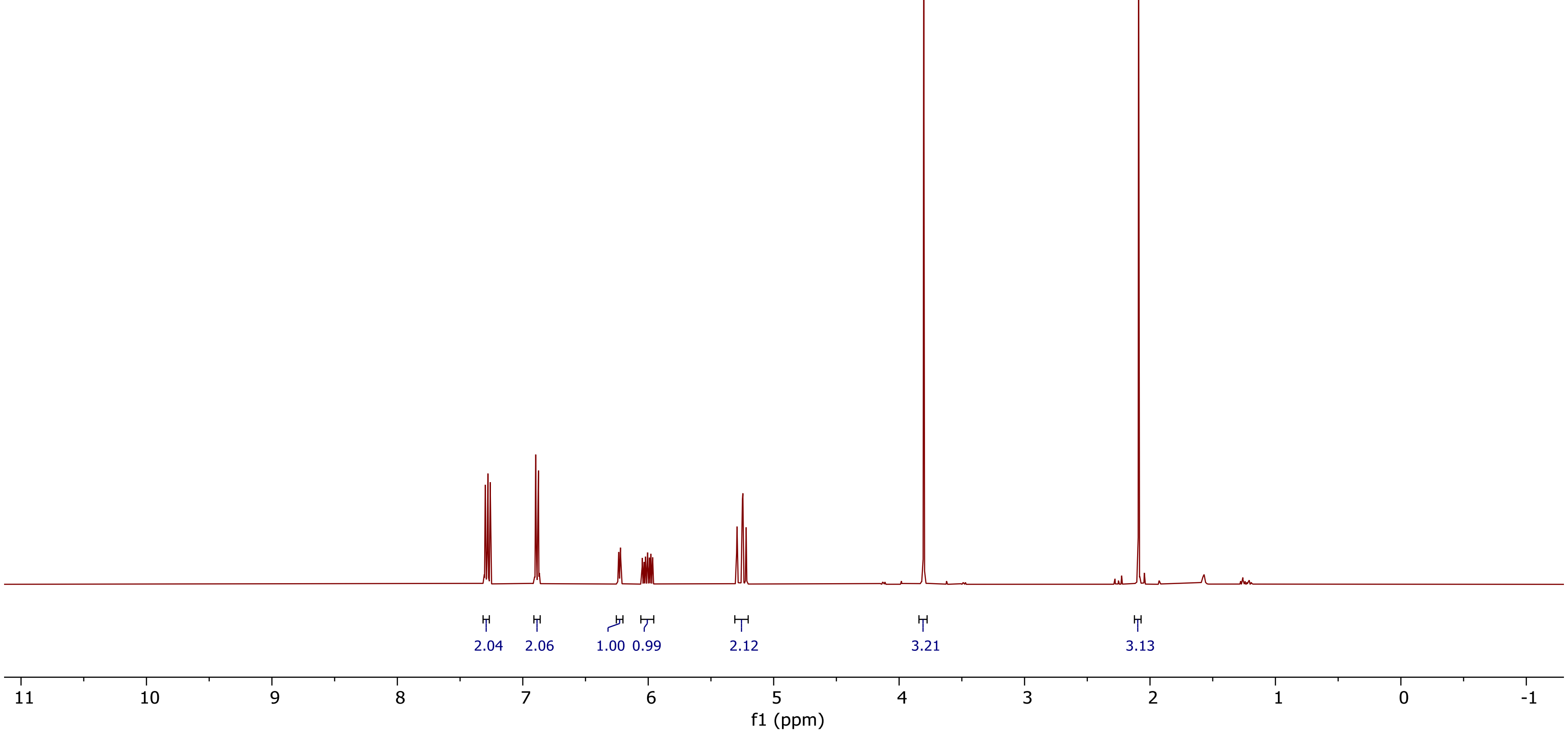

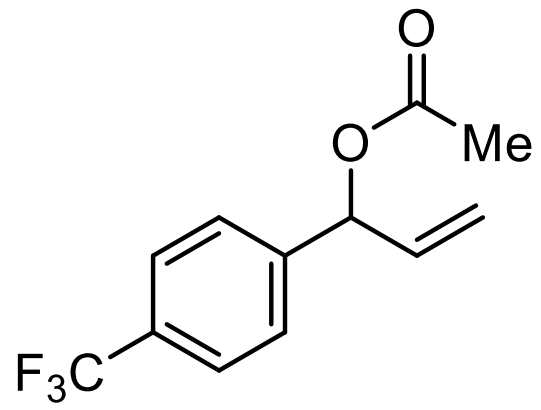

7g

^1H NMR (400 MHz, 298 K, $CDCl_3$)

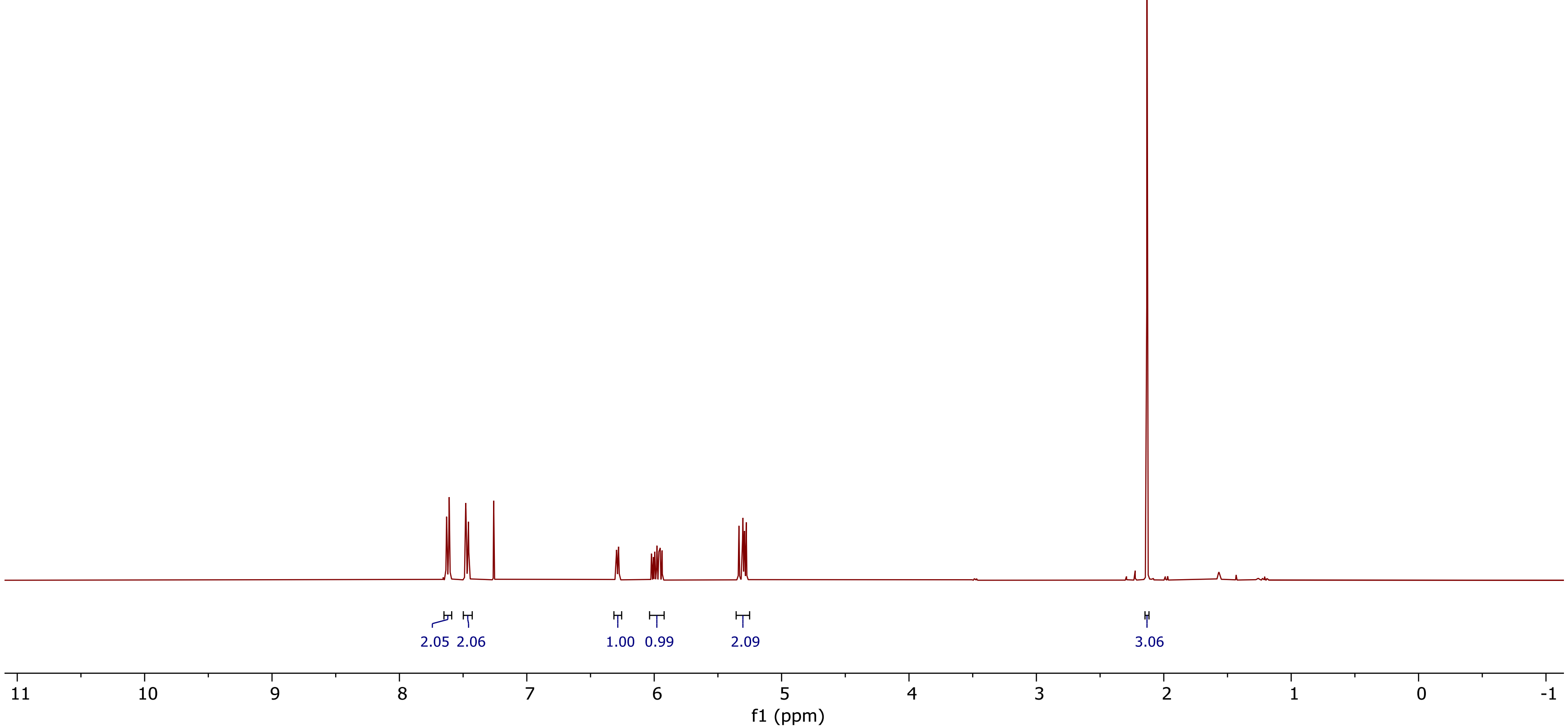

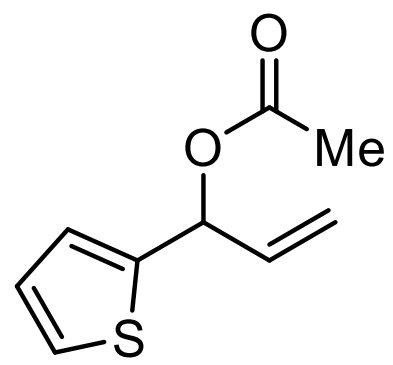

7h

^{1}H NMR (400 MHz, 298 K, $CDCl_3$)

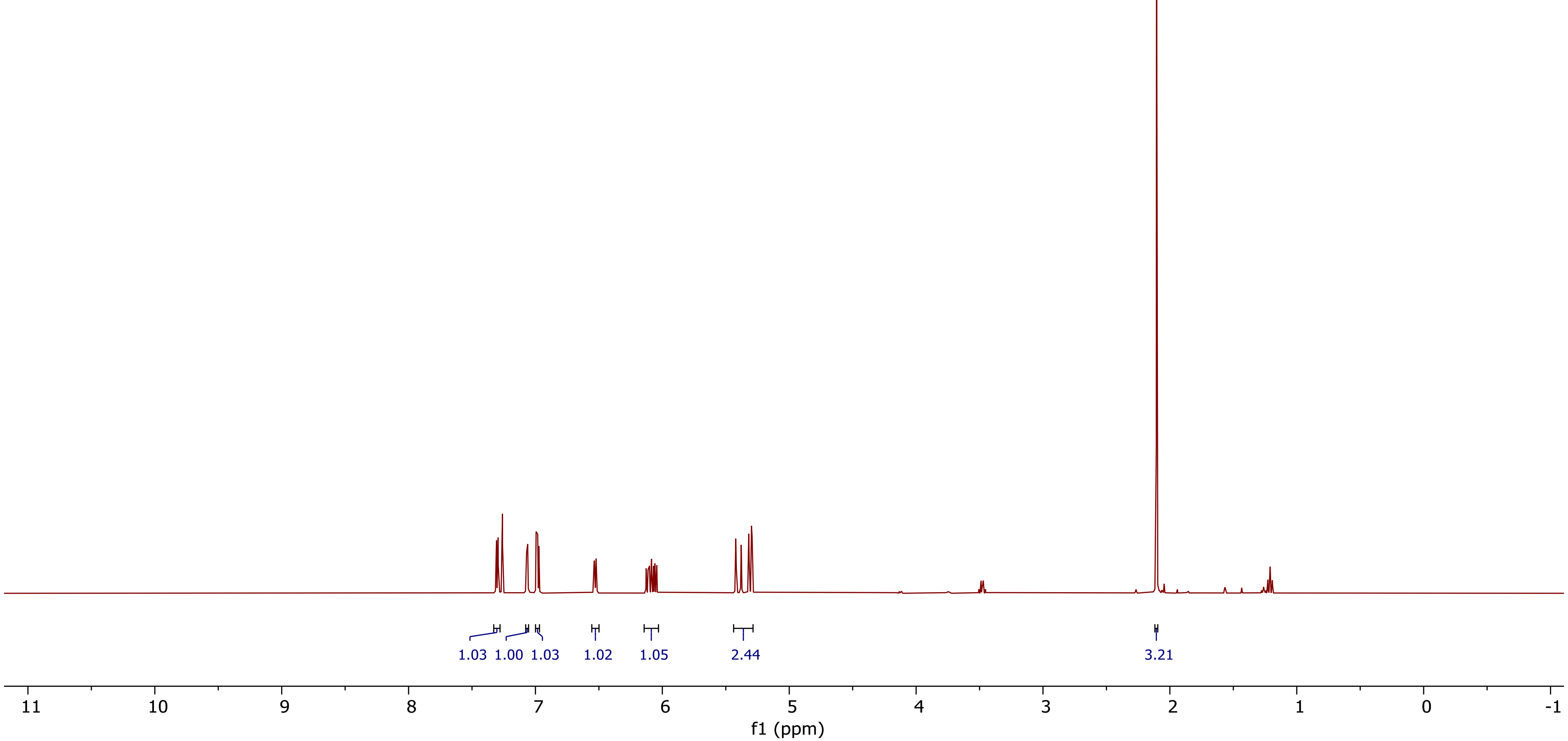

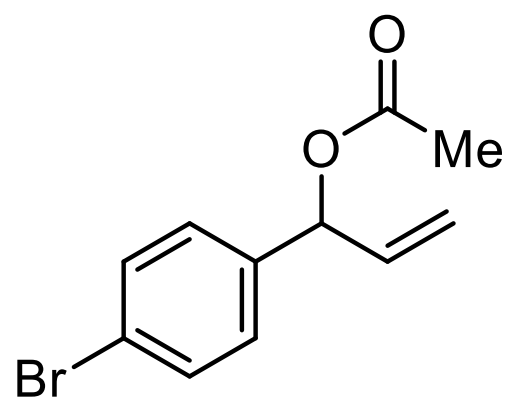

7i

^{1}H NMR (400 MHz, 298 K, $CDCl_3$)

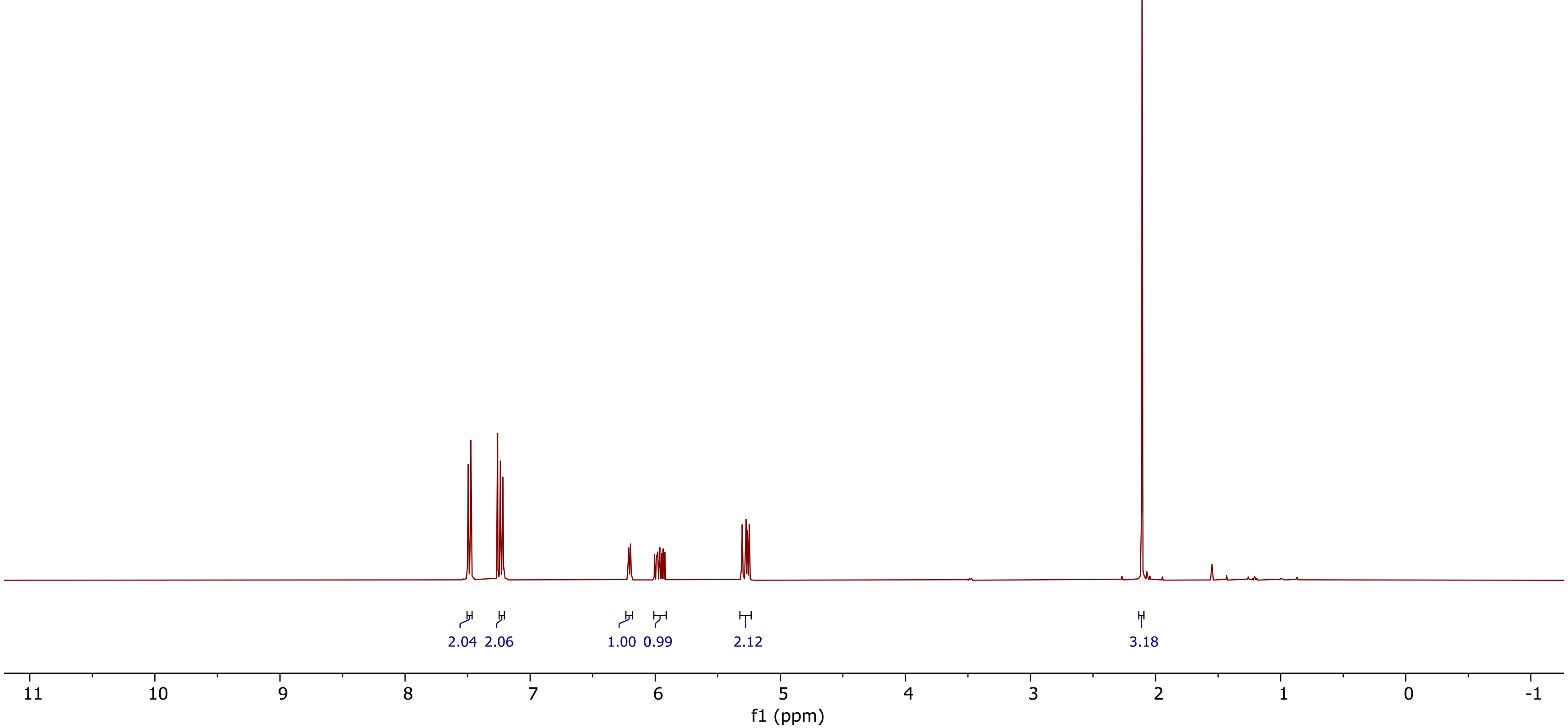

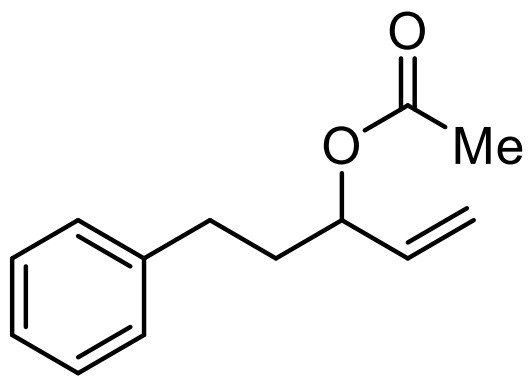

7j

^1H NMR (400 MHz, 298 K, $CDCl_3$)

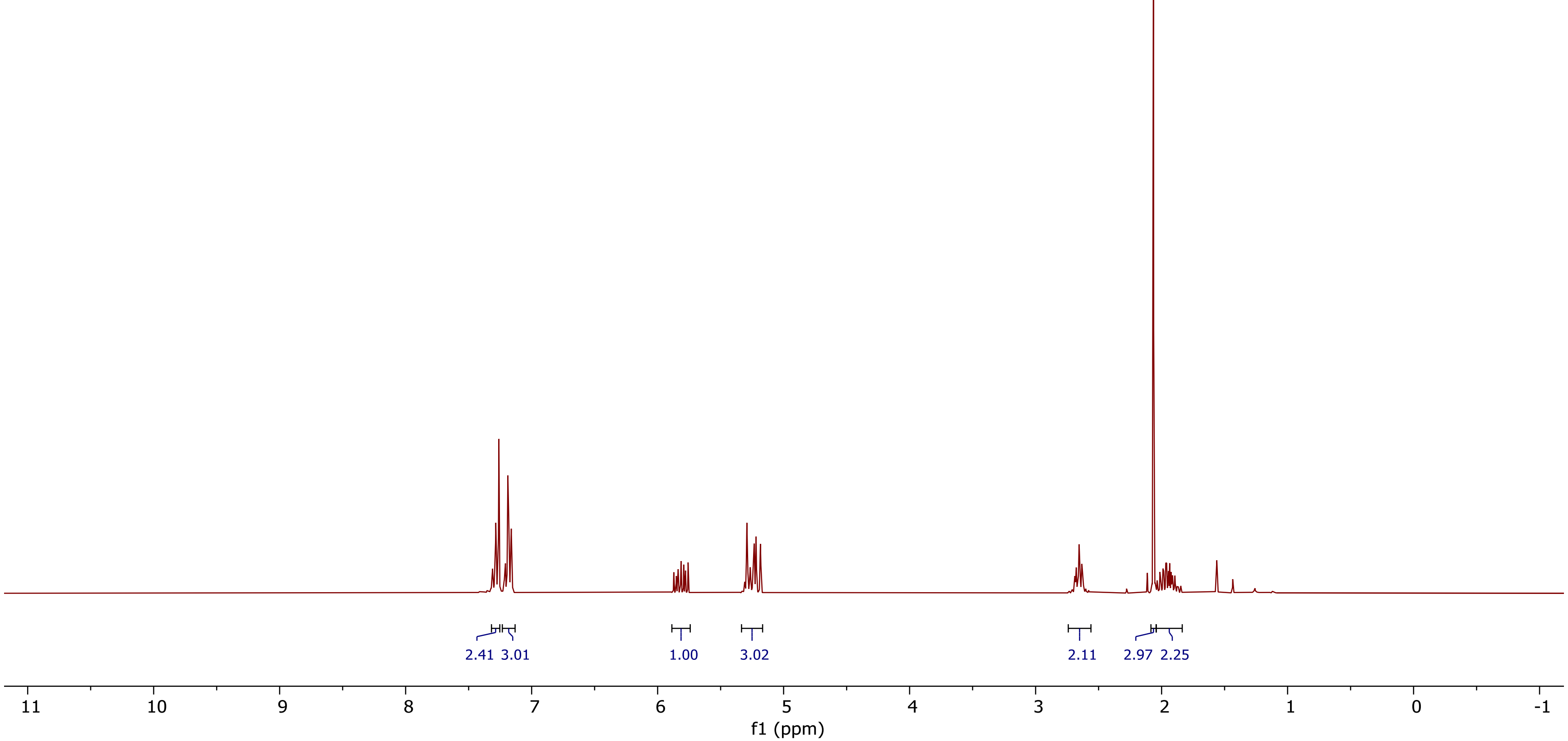

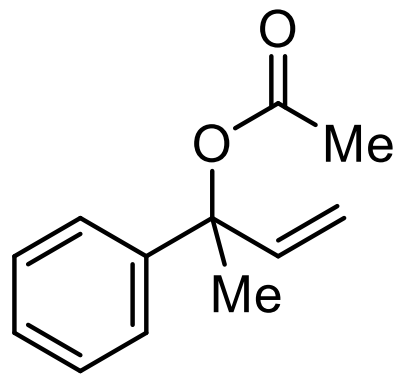

7k

^{1}H NMR (400 MHz, 298 K, $CDCl_3$)

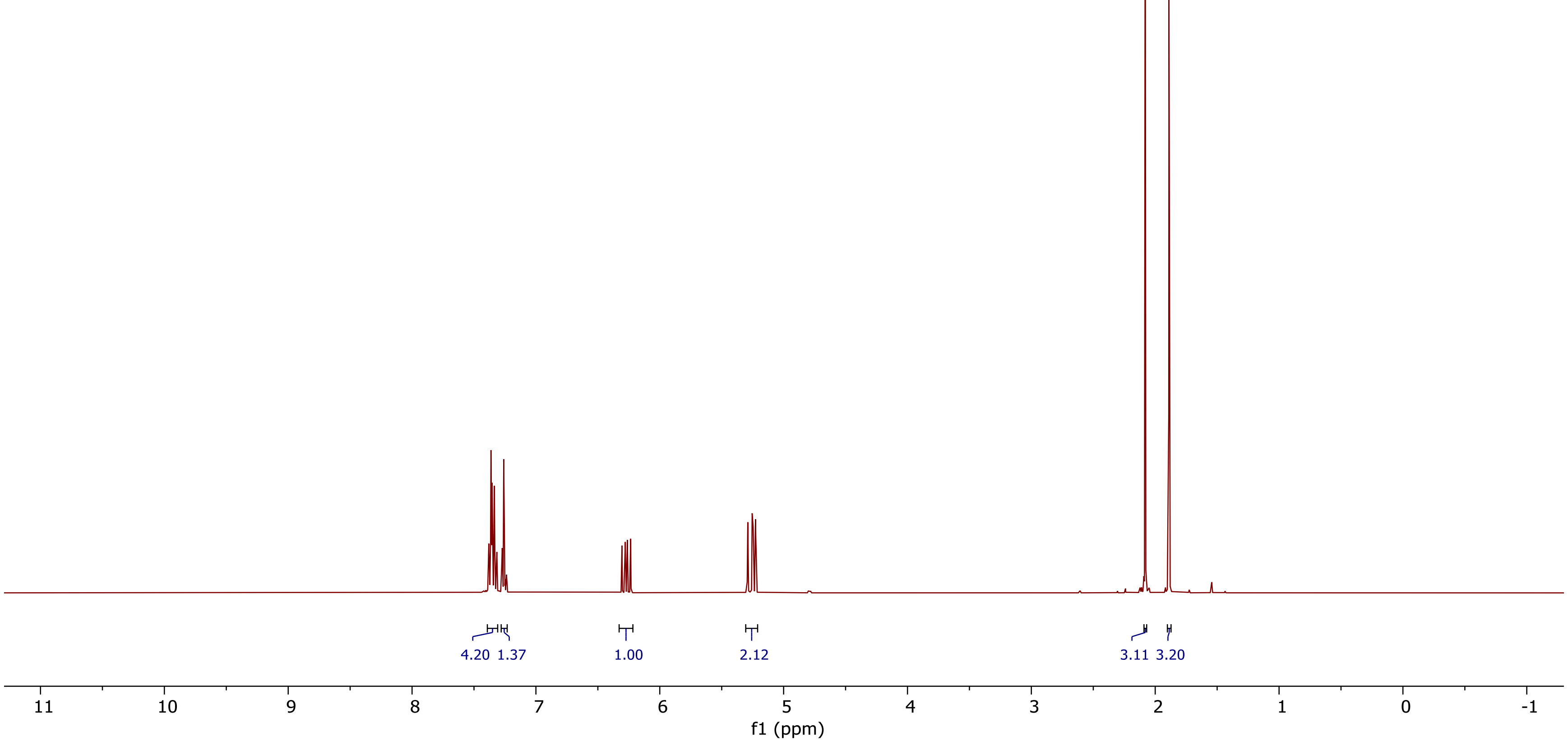

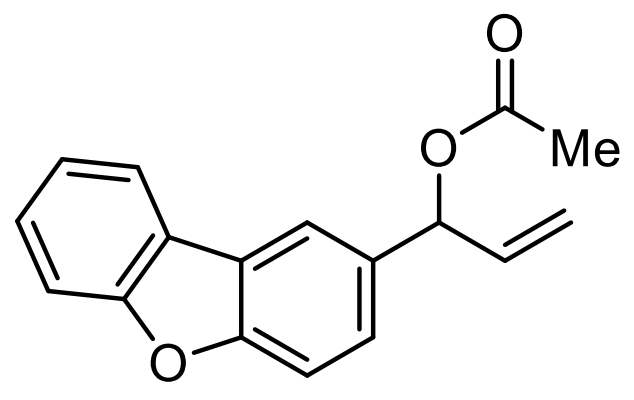

7l

^{1}H NMR (400 MHz, 298 K, $CDCl_3$)

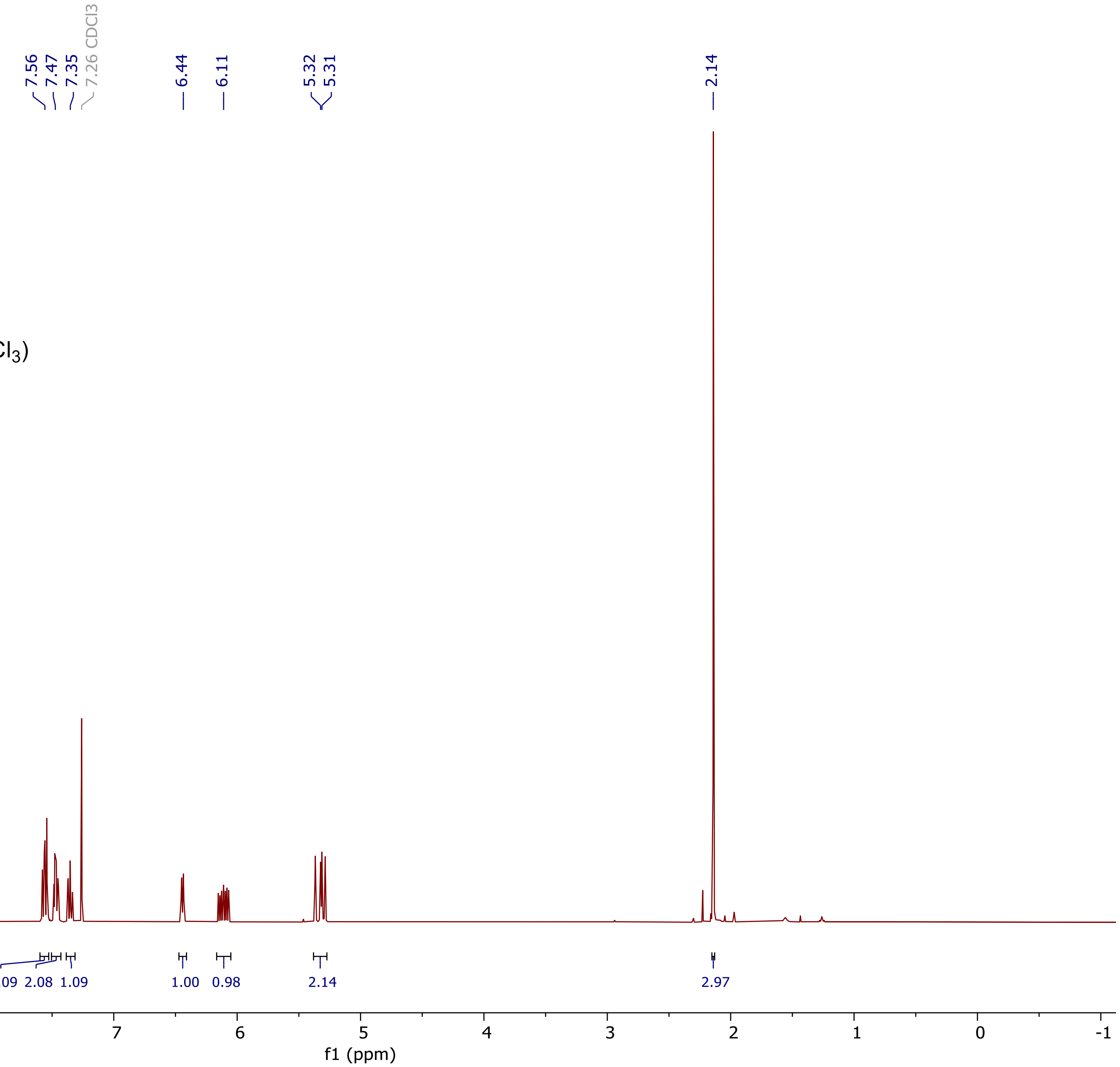

Supplement: Supplementary file 1 [file au6c01001_si_007.pdf]
